# The impact of facility-based transitional care programs on function and discharge destination for older adults with cognitive impairment: a systematic review

**DOI:** 10.1186/s12877-022-03537-y

**Published:** 2022-11-14

**Authors:** Alexia Cumal, Tracey J. F. Colella, Martine T. Puts, Poonam Sehgal, Sheryl Robertson, Katherine S. McGilton

**Affiliations:** 1 KITE Research Institute, Toronto Rehabilitation Institute – University Health Network, Bickle Centre, 550 University Avenue, Suite 236B, Toronto, ON M5G 2A2 Canada; 2 Lawrence S. Bloomberg Faculty of Nursing, University of Toronto, 155 College Street, Suite 130, Toronto, ON M5T 1P8 Canada; 3 KITE Research Institute, Toronto Rehabilitation Institute – University Health Network, Cardiovascular Prevention & Rehabilitation Program, 347 Rumsey Road, Suite 250, Toronto, ON M4G 1R7 Canada; 4 Home and Community Care Support Services Central East, 100 Consilium Place Suite 801, Scarborough, ON M1H 3E3 Canada

**Keywords:** Transitional care programs, Aged, Cognitive impairment, Functional status, Discharge destination, Systematic review

## Abstract

**Background:**

Older adults with cognitive impairment are frequently hospitalized and discharged to facility-based transitional care programs (TCPs). However, it is unknown whether TCPs are effective in improving their functional status and promoting discharge home rather than to long-term care. The aims of this systematic review were to examine the effectiveness of facility-based TCPs on functional status, patient and health services outcomes for older adults (≥ 65 years) with cognitive impairment and to determine what proportion post TCP are discharged home compared to long-term care.

**Methods:**

The Joanna Briggs Institute Critical Appraisal Manual for Evidence Synthesis was used to guide the methodology for this review. The protocol was published in PROSPERO (registration number CRD42021257870). MEDLINE, CINAHL, PsycINFO, the Cochrane Library, and EMBASE databases, and ClinicalTrials.gov and the World Health Organization Trials Registry were searched for English publications. Studies that met the following criteria were included: community-dwelling older adults ≥ 65 years who participated in facility-based TCPs and included functional status and/or discharge destination outcomes. Studies with participants from nursing homes and involved rehabilitation programs or transitional care in the home or in acute care, were excluded. Risk of bias was assessed using the Joanna Briggs Institute Critical Appraisal Checklists. Results are in narrative form.

**Results:**

Twenty-two studies (18 cohort and four cross sectional studies) involving 4,013,935 participants met inclusion criteria. The quality of the studies was mostly moderate to good. Improvement in activities of daily living (ADLs) was reported in eight of 13 studies. Between 24.4%-68% of participants were discharged home, 20–43.9% were hospitalized, and 4.1–40% transitioned to long-term care. Review limitations included the inability to perform meta-analysis due to heterogeneity of outcome measurement tools, measurement times, and patient populations.

**Conclusions:**

Facility-based TCPs are associated with improvements in ADLs and generally result in a greater percentage of participants with cognitive impairment going home rather than to long-term care. However, gains in function were not as great as for those without cognitive impairment. Future research should employ consistent outcome measurement tools to facilitate meta-analyses. The level of evidence is level III-2 according to the National Health and Medical Research Council for cohort and cross-sectional studies.

**Supplementary Information:**

The online version contains supplementary material available at 10.1186/s12877-022-03537-y.

## Background

As a result of the growing aging population there is a greater urgency to establish and maintain effective health care systems and programs. According to the World Health Organization, the proportion of adults over the age of 60 globally will increase from 12 to 22% between 2015 and 2050 [1]. Moreover, the number of people with dementia will almost double, from 50 million people worldwide in the year 2020 to 82 million in 2030, and 152 million in 2050 [2]. Cognitive impairment (CI), which can include dementia, delirium, and unspecified CI [3, 4], has a global prevalence of 5.1–37.5% among older adults aged 60–69 years, with a median of 20.1% [5]. Given the prevalence of CI in older adults and the growing number of people with dementia, there is an increasing demand for health care services that effectively meet their needs and facilitate positive health outcomes.

Systematic reviews have shown that older adults with CI have poorer health outcomes, including a higher risk for hospitalization [6], and increased risk for functional decline when hospitalized [7], and a higher risk for discharge to institutional long-term care post hospitalization [8], compared to those without CI. Moreover, recent reviews have shown that CI is associated with an increased length of hospital stay [9] and delayed discharge [10], which is problematic as these factors are associated with increased mortality, depression, and a decline in mobility and activities of daily living (ADLs) [11]. Therefore, these reviews highlight the need for specialized programs to help older adults with CI achieve positive outcomes such as improvement in functional status and discharge home.

After the acute issue is treated, some older adults remain in hospital longer due to the lack of community supports [12] or as the result of additional functional decline [13]. Thus, facility-based transitional care programs (TCPs) are one possible solution to facilitate discharge for these individuals. In this review, a facility-based TCP is defined as a post-acute program or unit within a facility which provides short-term, restorative care [14, 15] to older adults. Restorative care involves transitioning from providing full care to older adults to providing assistance to older adults, in order to maintain or improve functional abilities [16]. In terms of intensity, restorative care can involve two or more activities such as walking, mobility, and dressing for at least 15 min a day, six days a week [17]. Restorative care differs from inpatient rehabilitation programs in terms of therapy intensity, as inpatient rehabilitation programs are often high intensity, are typically 4–6 weeks in length, involve daily medical and nursing care, and 30–60 min physical and occupational therapy up to 5 times per week [18]. Throughout the literature, facility-based TCPs may be called by different names. In the United States, they may be called subacute care, post acute care, and skilled nursing facilities [14]. They are called intermediate care models in the United Kingdom, transition care programs in Australia, and transitional care programs in Canada [14]. These programs will hereafter be referred to as TCPs in this paper.

A recent scoping review found that TCPs admit older adult patients both with and without CI [14]. Moreover, functional status was the most common patient outcome, while discharge destination was a frequently used health services outcome [14]. Meta-analyses have shown that TCPs can significantly improve an older adult’s ability to perform ADLs, resulting in 80% of participants being discharged home [19], and a significant reduction in hospital readmission rates [20]. However, there are no reviews to date that have determined the impact of TCPs on functional status and discharge destination outcomes for older adults with CI. Given the growing aging population and increasing number of older adults with CI who are most at risk to decline functionally, it is critical that a review be undertaken to inform the creation, modification, and maintenance of effective TCPs for this population.

The review questions were: 1) What is the effectiveness of facility-based TCPs on functional status, patient and health services outcomes for older adults (≥ 65 years) with CI? 2) What proportion of older adults with CI at the end of the TCP are discharged home compared to long-term care?

## Methods

The Joanna Briggs Institute Critical Appraisal Manual for Evidence Synthesis (April 2021) [21] was used to guide the methodology for this systematic review and the results are reported according to the Preferred Reporting Items for Systematic Reviews and Meta-Analyses (PRISMA) 2020 checklist [22]. The review protocol was published in PROSPERO (https://www.crd.york.ac.uk/prospero/; registration number CRD42021257870).

### Search strategy

Comprehensive, systematic searches of OVID MEDLINE, CINAHL, PsycINFO, the Cochrane Library, and EMBASE databases were completed on July 15, 2021, from inception to present. The searches were updated on July 9, 2022. The search strategy was developed and refined by AC in consultation with KSM, TJFC, MTP, and a library information sciences expert (MM).

The three key search terms were: 1) transitional care programs; 2) older adults; and 3) cognitive impairment. In this review, cognitive impairment includes dementia, delirium, and non-specified cognitive impairment, as differentiating between them can be challenging [23]. Long-term care includes long-term care homes, nursing homes, and care homes [24]. Reference lists of included studies and reviews were also hand searched for relevant articles. The full search strategies and search results for each database can be found in Additional file 1.

Registries of ongoing trials from ClinicalTrials.gov and the 17 primary registries on the World Health Organization website [25], were searched independently by two reviewers (SR and AC or NZ). See Additional file 2 for registry search strategies, results and the dates the registries were last searched. Grey literature was not included in this review.

### Study selection

Pilot testing of the search strategy was completed by two independent reviewers (AC, PS, SR). Titles and abstracts were screened by two independent reviewers (AC, PS, SR, CW); full texts of studies were also screened by two independent reviewers (AC, SR, PS, NZ). Disagreements were resolved by discussion and consensus with a third reviewer (the other of AC, PS, SR, SW, or NZ). Covidence systematic review software [26] was used to manage and record data decisions.

Studies were eligible for inclusion if the following criteria were met: 1) included community-dwelling older adults (mean age ≥ 65 years) with CI (dementia, delirium, and/or CI) who were hospitalized and then admitted to a facility-based TCP; 2) TCPs were delivered in skilled nursing facilities, nursing homes, subacute and post acute units in hospitals, geriatric intermediate care facilities, and convalescent care [14, 15]; 3) included functional status and/or discharge destination as outcomes, with functional status defined as the ability to perform activities needed in daily life [27], measured using a validated tool, such as the Barthel Index, and discharge destinations including home, long-term care, and hospital; 4) published as a full length manuscript in a peer-reviewed journal; 5) designated as primary and secondary interventional studies (RCTs, quasi-experimental), primary and secondary observational studies (prospective cohort, retrospective cohort, cross-sectional, and case–control), and mixed-methods studies if there was quantitative data on functional status and/or discharge destination; 6) published in English.

The exclusion criteria were: 1) reviews, case studies, dissertations, conference proceedings, editorials, and qualitative studies; 2) mean age of participants < 65 years old; 3) participants living in a long-term care facility prior to hospitalization and TCP admission; 4) participants who were at the end of life (< 6 months prognosis) [28]; 5) rehabilitation programs; 6) transitional care provided in the home; 7) transitional care services provided only in acute care.

### Data extraction

**Fig. 1:**
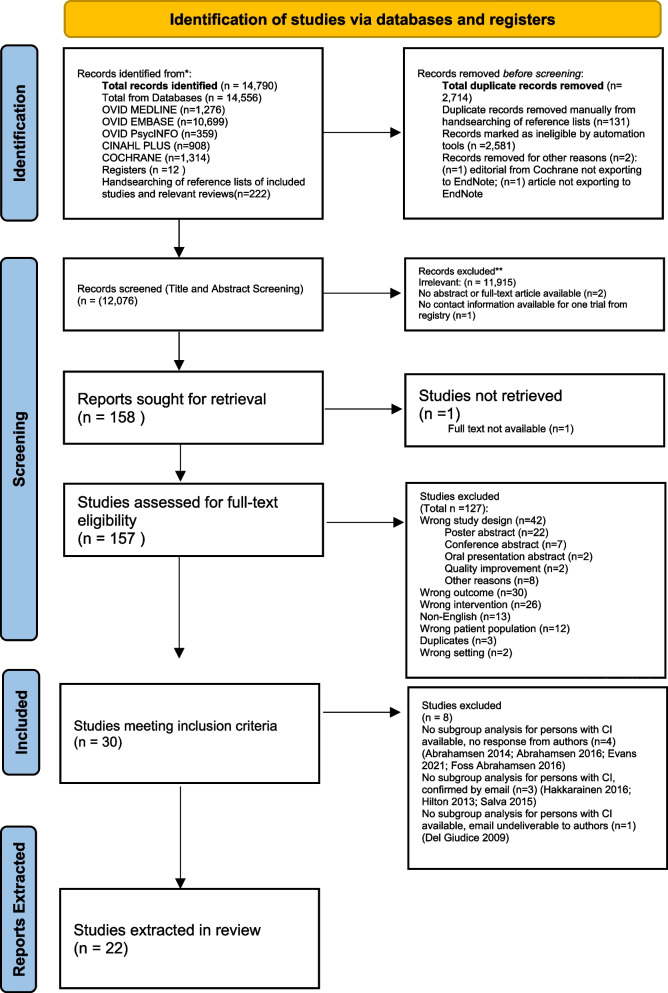
PRISMA flow diagram

Data were independently extracted by two reviewers (AC and SR, PS, CI, NZ, or TC) using a pre-piloted extraction form created with Microsoft Excel 2019. Information about the study design and methodology, TCP characteristics (staff complement, description of TCP services, inclusion and exclusion criteria), participant characteristics, and all outcome measures were extracted from the articles. The outcomes were reported according to the classification of outcomes as outlined in McGilton et al. [14]. The primary outcomes were functional status and discharge destination post TCP. The secondary outcomes were divided into patient outcomes, such as mortality, and health services outcomes, such as rehospitalization [14]. Disagreements between individual judgments were resolved by discussion and consensus. Authors [29–48] were contacted to ascertain any required information that was missing or unclear and data provided directly by the authors [29, 30, 47] was included in this review (See PRISMA diagram, Fig. 1). Information that was not in the study was reported as ‘NR’ (not reported). Extracted data for this review can be found in Additional file 4.

### Risk of bias assessment

The Joanna Briggs Institute Critical Appraisal Checklists [49] were used to assess the risk of bias in the included studies. The checklists were completed by two independent reviewers (AC, KSM, PS, TJFC) and disagreements were resolved by discussion and consensus. No studies were excluded on the basis of quality. Fair quality was assigned to studies if less than or equal to 50% of the checklist items were given a rating of yes, moderate quality if 51–80% of items were given a rating of yes, and good quality if greater than 80% of items were given a rating of yes, based on the rating system used by Benenson et al. [50].

### Synthesis of results

Results were synthesized in narrative form, using tables and figures according to outcome measure. A meta-analysis was not performed due to heterogeneity in the outcome measurement tools and data measurement times. There was also heterogeneity in the patient populations; although all participants had some form of CI, some studies focused on specific populations, such as veterans or older adults with heart failure or traumatic brain injury.

## Results

Figure 1 shows the PRISMA flow diagram which outlines the study selection process. The search of the databases yielded 14,556 articles, the search of the trial registers yielded 12 articles, and hand searches of reference lists of included studies yielded 222 articles, with 2,714 duplicates in total. After completing title and abstract and full text screening, 30 studies met the inclusion criteria. Among the 30 studies that included older adults with CI, only 22 performed a subgroup analysis or had separate data for older adults with CI. Therefore, data was extracted from the 22 studies which reported information on a total of 22 TCPs and 4,013,935 study participants.

### Risk of bias assessment

The majority (*n *= 21) of studies were of good or moderate quality (Additional file 3. Tables S1 and S2). Thirteen studies (59%) had good quality [38, 42, 46–48, 51–58], eight (36%) had moderate quality [30, 44, 45, 59–63], and one (5%) had fair quality [29]. The main issues that lowered study quality were the absence of strategies to address incomplete follow up in 13 studies (59%) [29, 30, 38, 42, 44–46, 57–60, 62, 63] and incomplete follow up or lack of description and exploration of reasons for loss to follow up in seven studies (32%) [29, 30, 44, 45, 60, 62, 63]. No randomized controlled trials met criteria for inclusion in the review. As well, there were four cross-sectional studies [30, 47, 53, 56]. Thus, although the studies had no obvious limitations, the review only included observational studies which allow only for the determination of association and not causality. The level of evidence is level III-2 according to National Health and Medical Research Council (NHMRC) for cohort and cross-sectional studies [64].

### Characteristics of included studies

Among the included studies, 14 (64%) were completed in the United States [42, 44–48, 51, 52, 54, 55, 57, 58, 62, 63], two (9%) in Australia [38, 53], and one (5%) each in Hong Kong [30], Italy [59], Japan [56], Norway [60], Singapore [61], and Taiwan [29]. Eighteen of the 22 articles (82%) were cohort studies, with 13 (59%) being retrospective cohort studies [38, 42, 44, 46, 48, 51, 52, 55, 57–59, 61, 62] and five (23%) prospective cohort studies [29, 30, 45, 60, 63]. There were also three (14%) cross-sectional studies [47, 53, 54] and one (5%) retrospective study design for data from a cross-sectional survey [56]. There were no RCTs or quasi-experimental studies among the included articles. Study characteristics are highlighted in Additional file 3: Table S3.

### Characteristics of the TCPs

There were a variety of settings where the TCPs were conducted, with skilled nursing facilities being the most common (*n* = 9) [42, 44, 45, 51, 52, 54, 58, 62, 63]. Other settings included nursing homes (*n *= 7) [38, 46–48, 55, 57, 60]; a subacute ward in a hospital (*n* = 2) [59, 61]; a community hospital-based post-acute care unit (*n* = 1) [29]; a post-acute convalescence unit (*n* = 1) [30]; a transition care facility (*n* = 1) [53] and geriatric intermediate care facilities (*n* = 1) [56].

Among the eight studies [29, 30, 38, 53, 56, 59–61] that reported on staff complement in TCPs, there were eight studies which involved nurses [30, 38, 47, 53, 56, 59–61]. Six studies each involved physiotherapists [29, 30, 38, 53, 56, 60] and occupational therapists [29, 30, 38, 53, 56, 60] and three studies each involved physicians [56, 59, 60], geriatricians [59–61], social workers [38, 53, 56] and personal care workers/aides [30, 47, 53]. Two studies each included case managers [29, 38], speech therapists [53, 56], and dieticians/nutritionist [29, 53]. One study included both care coordinators and allied health specialists [61] and one study involved a podiatrist [53]. Services provided in the TCPs were reported in ten out of the 20 studies [29, 30, 38, 46, 52, 56, 57, 59–61] and most involved therapies to improve physical function [29, 30, 38, 56, 57, 60]. Services included customised low-intensity therapies to increase physical, cognitive, and psychosocial function [38]; a physical reablement program through daily physiotherapy and occupational therapy sessions, exercise and ADL assistance, nutrition consultation, medication reconciliation, social worker visit on admission and as needed [29]; non-pharmacologic and pharmacological approaches to patients with dementia and challenging neuropsychiatric symptoms [61]; nursing care [46, 56, 57, 59] or treatment [60] or post-acute care [52] and rehabilitation [46, 52, 56, 57, 60]; 2 h daily, 5 days per week of mobility and ADL training [30]; and 180 min of direct nursing care [59]. Details on TCP characteristics are presented in Additional file 3: Table S3.

### Participant characteristics

Among the 22 studies, the mean age of participants ranged from 68.0 [47] to 84.6 [54] years. The percentage of females in the studies ranged from 0% [29] to 96.9% [47]. In terms of ethnicity, the majority were White, ranging from 71.7% [47] to 89% [63] of participants. The Charlson Comorbidity Index score was reported in seven studies [30, 45, 47, 51, 55, 61, 63] and ranged from 1.6 [35] to 3.0 [51].

Eight studies reported outcomes for older adults with dementia [29, 38, 48, 51, 52, 54, 56, 61]; six studies for older adults with CI [44, 46, 53, 58, 60, 62]; three studies for older adults with CI and dementia [30, 47, 57]; two studies for older adults with delirium [42, 45]; two studies for older adults with delirium and dementia [55]; and one study for older adults with delirium and subsyndromal delirium [63]. Delirium was most often measured using the Confusion Assessment Method [45, 55]. Dementia was measured most frequently through the Minimum Data Set admission assessment [45, 47, 55], by the International Classification of Disease coding [42, 56], or through medical records [30, 44]. CI was often defined using the Cognitive Function Scale [44, 46, 62]. Participant characteristics, including tools used to identify CI, are detailed in Table 1. The majority of participants had dementia [38, 42, 54, 56, 61] or mild CI [44, 46, 58, 62], however, the stage of dementia was not specified in the included studies.

**Table 1:** Participant Characteristics

Author Year	Number of patients	How CI was defined	Age (years) mean ± SD	Females n (%)	Ethnicity %	Cognitive Status Score Mean ± SD	Charlson Comorbidity Index Score mean ± SD
Abrahamsen 2016 [60]	Number of patients with an MMSE < 24: 206 (29% of total participants)	CI defined using the Norwegian version of the MMSE (score range 0–30, score < 24 is a sign of cognitive impairment)	Mean age of total sample 85, min–max (70–102)	Of total sample: *n* = 656 (68)	NR	Median MMSE of total sample: 26, min–max (8–30)	Of total sample: > 5 diagnoses, n(%) 567(62)
Bardenheier 2021 [54]	Total number of persons with ADRD: *n* = 2,134,798	ADRD was identified by the (CCW) flag in the Medicare beneficiary summary file	January 1 to September 30, 2015: mean age 84.6	January 1 to September 30, 2015: 141,475(64.9)	January 1 to September 30, 2015 Black % 8.6 Other race % 4.6	NR	NR
Burke 2021 [51]	Total number with dementia: 830,524 (34.3%) With dementia, used in matched cohort: 513,424 (34.3%)	Identification of dementia determined via coding in datasets (MDS, MBSF, MedPAR)	82.8 (8.1)	320,611 (62.4%)	White: 446,090 (86.9%) Black: 46,606 (9.1%) Asian: 5976 (1.2%) Other: 5059 (1.0%) Hispanic: 7951 (1.5%)	CFS: Cognitively intact: 260,736 (50.8%) Mild impairment: 180,667 (35.2%) Moderate impairment: 61,041 (11.9%) Severe impairment: 10,980 (2.1%)	3.0 (2.4)
Cations 2020 [38]	Individuals in residential care settings with dementia: *n* = 10,701 (25.4%)	Dementia was determined from the aged care eligibility assessment and dispensing of medications prescribed for Alzheimer’s disease in the 6 months before hospitalization	For all participants in residential TCP: 83.2(7.3)	For all participants in residential TCP: 25,999(61.7)	NR	NR	For all participants in residential TCP: Comorbidities (median, IQR) 0: 1948 (4.6) 1–4: 15,336 (36.4) 5–9: 21,971 (51.1) 10 + : 9210 (6.90)
Chong 2012 [61]	Persons with dementia and behavioural disturbances: *n* = 31 (16.9%)	Dementia not defined	For all participants: 81.1 (± 8.1)	For all participants: 100 (54.6)	For all participants: Chinese 83.6%; Malay 8.2%; Indian 7.1%; Others 1.2%	NR	For all participants: Modified CCI 1.6 ± 1.3; Severity of illness score index 2.0 ± 0.7
Downer 2022 [46]	Mild CI: *n* = 120,830 Moderate to severe CI: *n* = 74,183	Cognitive status was categorized as none, mild, and moderate or severe impairment according to the Cognitive Function Scale	For total sample: n(%) Age 66–70: 73,721 (12.0); Age 71–75: 99,050 (16.1); Age 76–80: 116,526 (18.9); Age 81–85: 124,364 (20.2); Age ≥ 86: 202,412 (32.9)	For total sample: 394,629 (64.1)	For total sample: White 86.0% Black 7.1% Hispanic 3.6% Other 3.3%	For total sample: CI: n (%) None 421,060 (68.3) Mild 120,830 (19.6) Moderate to severe 74,183 (12.0)	NR
Hang 2021 [19]	CI: *n* = 73	CI measured using Mini Mental State Examination (MMSE), scored ≤ 23/30 at admission	For all participants: 84.2 ± 8.3	For all participants: 103 (60.9)	NR	MMSE Discharged Home n(%) vs Other n(%) Yes 21 (42.9), 66 (63.5) *p* = 0.016*	For all participants: NR
Intrator 2021 [47]	With dementia: *n* = 1091 No/mild CI: *n* = 9808 Moderate/high CI: *n* = 4979	Dementia status from the MDS assessment at the start of the CLC episode, MDS item I4800; Cognitive function status from MDS items B0700, C0100, C0500, C1000	With dementia: 77.99 (10.53) No/mild CI: 68.00 (10.87) Moderate/high CI: 72.79 (11.68)	With dementia: 1.51% No/mild CI: 95.30% Moderate/high CI: 96.91%	American Indian or Alaska Native With dementia: 0.53%; No/mild CI: 0.86%; Moderate/high CI: 1.12% Asian: With dementia: 0.27% No/mild CI: 0.33% Moderate/high CI: 0.25% Black / African American: With dementia:: 21.26%; No/mild CI: 18.70%; Moderate/high CI: 20.69% Hispanic or Latino: With dementia: 5.67%; No/mild CI: 4.02%; Moderate/high CI: 4.25% Native Hawaiian or Other Pacific Islander: With dementia: 0.27%; No/mild CI: 0.49%; Moderate/high CI: 0.52% White: With dementia: 71.74%; No/mild CI: 75.52%; Moderate/high CI: 73.00% Unknown: With dementia: 0.27%; No/mild CI: 0.09%; Moderate/high CI: 0.15%	No/mild CI: *n* = 9808 Moderate/high CI: *n* = 4979	With dementia: 1.66 (SD 0.31) No/mild CI: 1.73 (SD 0.20) Moderate/high CI: 1.70 (SD 0.24)
Kosar 2017 [55]	Participants with delirium: *n* = 242,121	Delirium identified using the CAM criteria in the MDS resident assessment; Dementia status from the MDS admission assessment	83.2 (8.1)	141,451 (58)	non-white race 16%	CFS Score n (%): mild impairment 45,240 (19); moderate impairment 132,759 (55); severe impairment 43,844 (18) dementia n (%): 133,496 (55)	CCI score: 2.6 (2.0)
Lee 2008 [58]	Number of participants with CI not reported	CI measured by 7-category MDS CPS	For all participants: 82.34 ± 7.7 (range 65–102)	For all participants: 67%	NR	For all participants: 1.68(1.7) range 0–6	NR
Lee 2011 [29]	Participants with dementia: *n* = 139	Dementia defined as MMSE < 14 with education years < 6 years; MMSE < 24 with education years ≥ 6 years	82.6 ± 5.9	0 (0%) (All participants were male)	NR	MMSE score 5.9 ± 3.7	NR
Lei 2022 [48]	Veterans with dementia in PAC *n* = 8317	Dementia identified via ICD-9 coding	Aged 66–74: 16.9% Aged 75–84: 42.4% Aged 85 + : 40.7%	166 (2%)	Non-Hispanic white 76.7%	NR	NR
Loomer 2019 [44]	Participants with mild CI *n* = 45,064; Moderate CI *n* = 28,979; Severe CI *n* = 4117; Total number of participants with CI: *n* = 78,160	CI defined using the CFS in the MDS v.3.0; Alzheimer’s disease/dementia identified if it was an admission diagnosis	Ages 65–74 n (%) mild CI 8976 (19.9); moderate CI 3976(13.7); severe CI 728 (17.7); ages 75–84 n (%) mild CI 15,246 (33.8); moderate CI 9358 (32.3); severe CI 1395 (33.8); ages 85–90 n (%) mild CI 9485 (21.1); moderate CI 7147 (24.7); severe CI 750 (18.2); 90 + years n (%) mild CI 8598 (19.1); moderate CI 7241(25.0); severe CI 778 (18.9)	NR	NR	Alzheimer’s Disease/Dementia n (%) Among participants with mild CI 10,941 (24.3) Among participants with moderate CI 17,191 (59.3) Among participants with severe CI 2670 (64.9)	NR
Lueckel 2018 [62]	n (%) Participants with mild CI *n *= 22,043 (25); Moderate CI: *n* = 20,282 (23); Severe CI: *n* = 5144 (6)	CI defined using the CFS	For all participants: 83.9(7.5)	For all participants: 55,418(63)	For all participants: Nonwhite race (9)	CFS Score, *n* (%) 2: Mild impairment 22,043 (25); 3: Moderate impairment 20,282 (23); 4: Severe impairment 5,144 (6)	For all participants: Deyo-Charlson Comorbidity Index, mean (SD) 1.2 (1.4)
Madrigal 2021 [42]	Participants with delirium: *n* = 882	Delirium determined using the MDS 3.0 CAM; dementia determined via ICD-9 coding	81.0(9.3) *P* < 0.001	*n* = 31(3.5)	n (%) White: 710 (80.5) Black: 138(15.6) Hispanic 145(1.6) Other: 20(2.3)	Dementia, n (%): 525 (59.5)	Elixhauser comorbidity index, mean (SD) 4.3(2.8)
Marcantonio 2003 [45]	Participants with delirium symptoms: *n* = 126	Delirium assessed in the MDS; dementia assessed in MDS from the list of MDS-based comorbidities	Participants with delirium symptoms: 79 ± 8	Participants with delirium symptoms: 77(61)	Participants with delirium symptoms: Caucasian, 111 (88)	Participants with delirium symptoms: Dementia diagnosis (n (%)): 18 (14)	Participants with delirium symptoms: 2.1 ± 1.0
Marcantonio 2005 [63]	Participants with delirium: *n* = 188; Participants with subsyndromal delirium: *n* = 246; Total number of persons with CI: *n* = 434	Participants classified as having delirium if they met full CAM criteria; classified as having subsyndromal delirium if they have one or more CAM criteria	Participants with delirium: 83.3 ± 7.4 Participants with subsyndromal delirium: 82.5 ± 7.7	Participants with delirium: 127 (68) Participants with subsyndromal delirium: 167 (68)	Participants with delirium: White, not Hispanic: 146 (87) Participants with subsyndromal delirium: White, not Hispanic: 143 (89)	Participants with delirium: MMSE score 12.7 ± 7.0; MDAS score: 12.6 ± 4.4 Participants with subsyndromal delirium: MMSE score 18.8 ± 6.1; MDAS score: 7.1 ± 3.1	Participants with delirium: CCI score: 1.2 ± 1.2 Participants with subsyndromal delirium: CCI score: 1.4 ± 1.3
Mazzola 2022 [59]	With dementia: *n* = 98 With delirium: *n* = 58	Dementia via history of pre-existing dementia and using the MMSE. Delirium was assessed with the 4AT test	For whole sample: 78.2 (11.6)	For whole sample: 202 (49.7%)	NR	Mini-Mental State Examination Mean (SD) 21.3 (7.5)	For whole sample: 3.0 (1.9)
Miu 2016 [30]	Community-dwelling participants with CI: *n* = 78; with dementia: *n* = 31	CI was determined using the MMSE; delirium via the CAM-CR; dementia identified through medical records	Community-dwelling participants with cognitive impairment: 83.9 ± 6.5; with dementia: 84.2 ± 6.6	Community-dwelling participants with cognitive impairment: 42(54); with dementia: 19(61)	NR	NR	Community-dwelling participants with CI: mean CCI = 2.36 ± 1.55; with dementia: mean CCI = 2.52 ± 1.23
Nakanishi 2016 [56]	Participants who had dementia: *n* = 2483	Dementia diagnosis was determined through the ICD-10	For total sample, based on discharge destination: home: 84 ± 8.3 hospital: 85.2 ± 8.2 Facility: 84.1 ± 8.2 Death: 88.6 ± 7.4	For total sample, based on discharge destination: sex, male, n (%) home: 763(26.3) hospital: 1453 (32.8) facility: 538 (26.0) death: 193 (32.6)	NR	Cognitive impairment^a^ (range 1–6), mean ± SD For total sample, Based on discharge destination: home: 2.6 ± 1.4 hospital: 3.0 ± 1.1 facility: 2.8 ± 1.2 death: 3.2 ± 1.0	NR
Simning 2022 [52]	With dementia: *n* = 10,426	Dementia determined through SNF admission MDS and ICD-9 codes	Discharged home: 81.7 ± 8.5 Not discharged home: 83.0 ± 89.0	Discharged home: 66.40% Not discharged home: 60.90%	Discharged home: White: 84.10% Not discharged home: White: 79.3%	Discharged home: % with dementia: 16.20% Not discharged home % with dementia: 31.9%	Discharged home: Number of diagnoses: 6 ± 3.7 Not discharged home: Number of diagnoses: 5.9 ± 3.6
Wysocki 2015 [57]	Moderately impaired (19.3%) *n* = 171,152 Severely impaired (9.7%) *n* = 86,019 Dementia 12.9%) *n* = 114,396; Any signs of delirium: = 25,717	CI defined using the CPS; Dementia determined if participant had a diagnosis of dementia; Signs of delirium were based on the CAM items	Mean age of total sample: 77.4 ± 12.3 NR for patients with CI or dementia	For total sample: 64.4% NR for patients with CI or dementia	Race, not white for total sample 15.8% NR for patients with CI or dementia	For whole sample: % with Dementia: 12.9; % with moderately impaired cognition for whole sample: 19.3; % with severely impaired cognition for whole sample: 2.9; % with Alzheimer’s disease for whole sample: 2.9	NR

Results are for persons with CI (CI, dementia, delirium) only, unless otherwise stated

*CI* Cognitive impairment, *N* Number, *ADRD* Alzheimer's Disease and related dementias, *MMSE* Mini-Mental State Examination, *CCI* Charlson Comorbidity Index, *MDAS* Memorial Delirium Assessment Scale, *IQR* Interquartile range, *NR* Not reported, *CLC *Community Living Center, *CFS* Cognitive Function Scale, *CI* Cognitive impairment, *CCW* Chronic Condition Data Warehouse, *CAM* Confusion Assessment Method, *MDS* Minimum Data Set, *ICD-9* International Classification of Disease, Ninth Revision, *CAM-CR* Chinese version of the CAM, *ICD-10* International Classification of Disease, Tenth Revision, *SPMSQ* Short portable mental status questionnaire, *CPS* Cognitive Performance Scale, *MBSF* Master Beneficiary Summary File, *SD* Standard deviation

^a^ Cognitive impairment scale not specified, however, authors report that it demonstrates consistency with scores on the MMSE and Hasegawa Dementia Scale-Revised

### Research question 1: effectiveness of TCPs on functional status, patient and health services outcomes

#### Performance of ADLs

Thirteen studies assessed the impact of TCPs on functional status [29, 30, 38, 42, 44–46, 51, 53, 55, 57, 58, 62], see Table 2. Functional status was primarily measured as performance of ADLs, with the Minimum Data Set ADL score (*n* = 8) being the most commonly used tool [42, 44–46, 55, 57, 58, 62]. Performance of ADLs was measured at multiple time points, with assessment most often at admission, discharge, and at 1-month. For functional status outcomes, only those reported from admission to discharge, or first time point are reported below, but follow up time points are found in Table 2.

**Table 2:** Change in Functional Status

Author Year	Outcome (Explanation of Scoring)	Measurements
Burke 2021 [51]	Improvement in functional status as measured by the **BI**	**At baseline:** With dementia: BI: 21.3 (15.6) **At discharge:** With dementia: Yes improvement: 145,838 (28.4%); No improvement: 233,592 (45.5%); Missing: 133,994 (26.1%) Without dementia: Yes improvement: 156,950 (30.6%); No improvement: 210,436 (41.0%); Missing: 146,038 (28.4%)
Cations 2020 [38]	**mBI** Adjusted odds ratio for individuals with dementia in residential TCPs (95% confidence interval) (multinomial regression analysis assessing factors associated with improved and worsened mBI scores from entry to exit of TCP. Improved = moved up one category (e.g. from ‘severe dependence’ to ‘mild dependence’ in 10-item mBI). Worsened = moved down one category	**At discharge:** Improved (*n* = 9236) aOR = 0.70 (0.66–0.75) Worsened (*n* = 9588) aOR = 0.69 (0.65–0.73)
Downer 2022 [46]	**MDS** The difference in self-care function between admission and discharge was calculated; a change score greater than zero indicates functional improvement	**At admission:** Mild CI (vs no CI) -0.71 *p* < -0.001; Moderate to severe (vs. no CI) -2.31 *p* < -0.001 **At discharge:** Mild CI (vs no CI) -1.88 *p* < -0.001.; Moderate to severe (vs. no CI) -4.98 *p* < -0.001 **Difference:** Mild CI (vs no CI) -1.51 *p* < -0.001; Moderate to severe (vs. no CI) -3.78 *p* < -0.001
Hang 2021 [19]	**mBI** (score 0 to 100—higher scores indicate better ADL performance). Measured at admission and discharge from TCP to determine changes in functional ability that occurred during TCP	**At baseline:** Age 60–79 CI: 40.2 (32.5–47.8); No CI: 52 (42.5–61.5) Age ≥ 80: CI: 50.1(45.6–54.9); No CI: 55.1 (47.9–62.4) **At discharge:** Age 60–79: CI: 52.8 (44.7–60.8) Mean difference 12.6 (6.11–19.1) *p* < 0.001; No CI: 71.1 (60.9–81.3) Mean difference 19.1 (10.9–27.4) *p* < < 0.001 Age ≥ 80: CI: 64.5 (59.6–69.4) Mean difference 14.2 (10.3–18.1) *p* < 0.001 No CI: 68.5 (60.3–76.6) Mean difference 13.3 (6.63–20.0) *p* < 0.001
Kosar 2017 [55]	Functional Improvement (**ADL self-performance items in the MDS**) Difference between composite score at admission and score at first discharge assessment within 30 days was calculated. Functional improvement was indicated by a positive difference. Functional improvement = at least a one-point improvement in composite ADL score	**At discharge:** 51.9% of patients with delirium but no dementia had functional improvement, compared to 60.9% of persons with no dementia and no delirium RR 0.89 (0.87, 0.90) 46.2% of patients with delirium and dementia had functional improvement RR 0.87 (0.86, 0.88), compared to 53.3% of patients who had dementia but no delirium RR 0.87 (0.86, 0.88)
Lee 2008 [58]	**7-item MDS-ADL Scale** (Regression analysis was used to examine the association between physical function, as measured by the MDS-ADL scale, and admission factors, with CPS as the covariate); 7 Item MDL-ADL scale rates ADL performance scores from independent (0) to total dependence (4), with a range of scores from 0 to 28	**At 3 months,** estimate is 0.20, 95% confidence interval (0.13–0.28), z score is 5.34 *p* < 0.0001 **At 6 months,** estimate is 0.36, 95% confidence interval (0.27–0.45), z score = 7.90 *p* < 0.0001 **At 9 months,** estimate is 0.34, 95% confidence interval (0.24–0.44), z score = 6.70 *p* < 0.0001 **At 12 months,** estimate is 0.37, 95% confidence interval (0.26–0.48), z score = 6.88 *p* < 0.0001
Lee 2011 [29]	**BI** Improvement is shown by an increase in score from admission to 4 weeks after services	**At baseline:** With dementia: 24.0 ± 29.0; No dementia: 47.1 ± 33.6 **At 4 weeks:** Participants with dementia: 42.6 ± 29.4 *p* < 0.001; No dementia: 66.2 ± 32.9 *p* < 0.001
	**IADL**	**At baseline:** With dementia: 0.5 ± 1.3; No dementia: 1.8 ± 2.4 **At 4 weeks:** Participants with dementia: 1.0 ± 1.7 *p* < 0.001; No dementia: 3.0 ± 2.8, *P* < 0.001
Loomer 2019 [44]	Improvement in self-care and mobility (difference between admission and discharge scores for self-care and mobility came from Section GG of the **MDS 3.0 v1.14.1**) Improvement in composite self-care and composite mobility scores was calculated by subtracting participants’ admission score from their discharge score. The percentage of participants whose composite scores stayed the same or improved were calculated Expected self-care and mobility (a dichotomous variable to determine residents whose scores were the same or higher than expected	**At discharge:** Percentage of residents whose observed self-care and mobility performance improved or stayed the same between admission and discharge: **Composite self-care score:** mild CI 92.1 (*p* < 0.001, ref: intact cognition), moderate CI 87.2 (*p* < 0.001, ref: mild CI), severe CI 84.3 (*p* < 0.001, ref: moderate CI), no CI 95.5 **Composite mobility score:** mild CI 94.8 (*p* < 0.001), moderate CI 91.5 (*p* < 0.001), severe CI 87.6 (*p* < 0.001), no CI 97.0 Percentage of residents whose observed self-care and mobility scores at discharge are the same or higher than their expected discharge score: **Composite self-care:** mild CI 55.8 (*p* < 0.001, ref: intact cognition), moderate CI 51.0 (*p* < 0.001, ref: mild CI), severe CI 45.4 (*p* < 0.001; ref: moderate CI), no CI 63.3 **Composite mobility score:** mild CI 53.9 (*p* < 0.001, ref: intact cognition), moderate CI 48.5 (*p* < 0.001, ref: mild CI), severe CI 44.6 (*p* < 0.001, ref: moderate CI), no CI 62.3
Lueckel 2018 [62]	**MDS ADL**—Change in score between admission and discharge The ADL score ranges from 0 (no impairment) to 28 (total dependence). It is considered an improvement in function if their discharge ADL score was less than their admission score	**At discharge:** Among residents with cognitive impairment, 57.4% had functional improvement at discharge compared with 68.8% without impairment (RR 0.86, 99% CI 0.83, 0.88) (discharged within 30 days)
Madrigal 2021 [42]	**The MDS 3.0 ADL assessment** For MDS, scores are 0–28, with higher scores indicating lower functional ability. Functional recovery = ADL scores at admission minus ADL scores at 30 days Functional recovery scores were categorized into 3 groups: Functional improvement (score > 0); no change in function (score = 0); worse functional performance: (score < 0)	**At baseline:** with delirium: mean (SD) ADL score on SNF admission 18.3 (4.7) *p* < 0.001, SMD 0.44; no delirium: mean (SD) ADL score on SNF admission 16.1 (5.2) **At discharge:** ADL score change from admission to follow up assessment, mean (SD) Delirium: 0.6 (2.9) *p* < 0.001; no delirium: 1.8 (3.6) *p* < 0.001 **At 1 month:** Follow up assessment at 30 days unless unavailable, then 15-, 45-, 60- or 90- day assessment was used. Categorical ADL score change from admission to follow-up, No. (%): **worse functional performance:** delirium 1991 (21.7) *p* < 0.001; no delirium: 2821(14.4) *p* < 0.001 **no change**: delirium 364(41.3); *p* < 0.001; no delirium:6655(33.9)* p* < 0.001 **improved functional performance:** delirium: 327 (37.1) *p* < 0.001; no delirium: 10,137(51.7) *p* < 0.001
Marcantonio 2003 [45]	Linear regression model to measure the association of persistent delirium symptoms and functional recovery as determined by the **MDS ADL score**; β for ADL change: positive change indicates worsening of ADL function	**At baseline:** Participants with delirium symptoms: 23 ± 9 *p* < 0.01 **At 1 or 2 weeks:** Participants with persistent delirium: β for ADL change = 3.6, 95% CI [2.2,5.0]
	Linear regression model to measure association of persistent delirium symptoms and functional recovery as determined by **MDS IADL score**	**At baseline:** Participants with delirium symptoms: 21 ± 6 *p* < 0.01 **At 1 or 2 weeks:** Participants with persistent delirium: β for IADL change = 2.6, 95% confidence interval [1.4, 3.6]
Miu 2016 [30]	**mBI** (Higher scores indicate greater independence in performance of activities of daily living). A gain of mBI score from admission to discharge indicates improvement	**At baseline:** Community-dwelling participants with CI: 47.1 ± 29.5; with dementia 35.7 ± 28.6; No CI: 60.6 ± 29.5 **At discharge:** Community-dwelling participants with CI:50.8 ± 27.7; with dementia: 46.2 ± 27.1; with no CI: 71.5 ± 27.2 **At 1 month:** Community-dwelling participants with CI: 48.9 ± 30.9; with dementia: 42.4 ± 30.7; with no CI: 67.8 ± 28.8
Wysocki 2015 [57]	**MDS 3.0**^a^** ADL change** (linear regression coefficient –Regression result predicting ADL Improvement); ADL change calculated as admission score minus discharge score, and so positive scores mean improvement, negative scores indicate decline. A negative coefficient estimate shows less improvement in ADLs	**At discharge:** Coefficient estimates (standard error): Moderately impaired: -0.761 (0.011) *p* < 0.001; Severely impaired: -1.698 (0.016) *p* < 0.001; Dementia: -0.416 (0.013) *p* < 0.001; Any signs of delirium: -0.7333(0.026) *p* < 0.001

*NA* Not applicable, *BI* Barthel Index, *SD* Standard deviation, *IADL* Instrumental activities of daily living, *CI* Confidence interval, *mBI* modified Barthel Index, *aOR* adjusted odds ratio. Score is mean ± SD unless stated otherwise, *CPS* Cognitive Performance Scale, *BI* Barthel Index

^a^ MDS 3.0 scale ranges from 0 to 28, with higher scores indicating greater impairment. ADL change was calculated as the admission score minus the discharge score, and so positive scores indicate improvement and negative scores indicate decline

Improvement in functional status was reported in eight studies for older adults with CI [29, 30, 42, 44, 51, 53, 55, 62]; however, overall, a greater percentage of participants without CI experienced functional improvement compared to those with CI. Improvement in performance of ADLs was reported in 28.4% [51] to 53.3% [55] with dementia, 46.2% with dementia and delirium [55], 51.9% with delirium [55], and 57.4% of participants with CI [62], compared with 30.6% [51] to 68.8% [62] of participants without CI. Moreover, gains in functional status scores were smaller for older adults with CI [30], dementia [29, 53], and delirium [42], compared to those without CI**.** Furthermore, poor functional status post TCP was reported in four studies [38, 45, 57, 58] and having CI was associated with significantly less improvement in one study [46].

#### Patient outcomes

Other patient outcomes were assessed in six studies [29, 45, 54, 55, 62, 63], with mortality (*n* = 5) [30, 54, 55, 62, 63] being the most common (Additional file 3: Table S4). Three-month mortality ranged from 8.2% [54] to 33.7% [55] for participants with CI, compared to a range from 5.7% [27] to 12.8% [55] for those with no CI. Six-month mortality rate for older adults with delirium was 25.0% and 18.3% for those with subsyndromal delirium, compared to only 5.7% for those without delirium [63]. Furthermore, 1-year mortality for older adults with CI ranged from 38.8% [62] to 49.1% [55], compared to a range from 24.4% [55] to 26.2% for those without CI [62]. There were improvements between admission and at four weeks in the Mini-Mental State Examination, Geriatric Depression Scale, and Mini Nutritional Assessment scores in older adults with dementia, however, those without CI had greater improvements in the Geriatric Depression Scale than those with CI [29].

#### Health services outcomes

Health services outcomes were measured in five studies [29, 30, 54, 55, 62] (Additional file 3. Table S5), with mean length of TCP stay being most commonly evaluated [29, 30, 62]. Mean TCP length of stay for older adults with CI ranged from 28.6 days [29] to 37.2 days [30], compared to a range from 27.5 days [62] to 31.7 days for older adults without CI [30]. Between 13.4 and 16.4% of participants with dementia were re-hospitalized within 30 days [54], while 17.2% of older adults with delirium and dementia, 26.4% of older adults with delirium but no dementia [55], and between 13.8% and 16.8% of patients without dementia [54] were re-hospitalized. Between 24.6% [54] and 38.7% [30] of participants with dementia and 34.3% of older adults with CI [30] were re-hospitalized within 90 days [54], compared to between 22.3% [30] and 27.2% [54] of older adults with no CI.

### Research question 2: proportion of older adults discharged home and to LTC

**Fig. 2:**
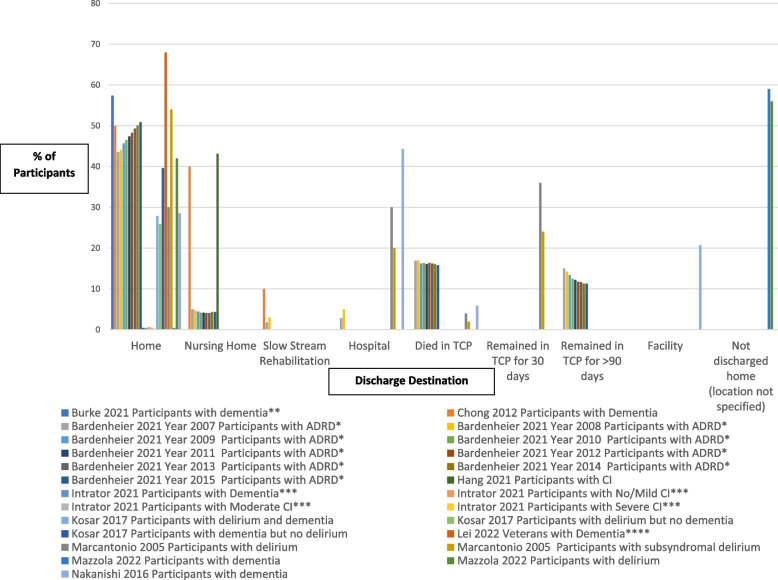
Percentage of participants with CI discharged by destination Legend for Fig. 2: ADRD = Alzheimer’s Disease and Related Dementias; CI = Cognitive impairment; TCP = Transitional Care Program; * = Outcome is Successful Discharge (defined as being discharged alive from a skilled nursing facility (SNF) to the community within 90 days of SNF admission without subsequent inpatient healthcare utilization for 30 continuous days; ** = Outcome is community discharge rate (metric used on Nursing Home Compare is the rate of beneficiaries who are able to leave the SNF by 100 days after hospital discharge and remain in the community (i.e., alive and outside the hospital and nursing home) for at least 30 days after SNF discharge; *** = Outcome is Successful Discharge (discharge to community within 100 days of a nursing home admission, defined as: Discharge to the community within 100 days (allowing for interim discharges from Community Living Center  to hospital if the Minimum Data Set noted that return was anticipated, observation stays, and emergency room use), and no unplanned admissions to a hospital, a nursing home or observation stay, and not dying within 30 days following discharge; **** = Outcome is Successful Discharge to the community (During the 30 subsequent days the veteran did not die, was not readmitted to a hospital for an unplanned inpatient stay, and was not admitted to a nursing home): No * indicates that it is the percentage of older adults with CI discharged home, and does not specify that it needs to have been a “successful” discharge as defined in the 4 studies with a *

Eleven studies assessed discharge destination [38, 47, 51–56, 60, 61, 63]. The most common discharge destination was home, followed by hospital, and then nursing home (Fig. 2). The percentage of participants with any form of CI discharged home ranged from 24.4% [56] to 68% [48]; to hospital ranged from 20% [63] to 43.9% [56]; and to long-term care ranged from 4.1% [27] to 40% [35]. In comparison, for participants without CI, between 55.1% [55] and 73% [63] were discharged home, 13% were discharged to hospital [63], and 2.7% to 3.5% [54] were discharged to long-term care. Moreover, participants with dementia in facility-based TCPs were less likely to be discharged to home (adjusted odds ratio (aOR) 0.53 [28] and aOR 0.4 [52]) compared to participants without dementia. Finally, participants with CI were less likely to be discharged home (odds ratio (OR) 0.46), more likely to be discharged to the nursing home or be deceased after two months (OR 2.95), and more likely to transfer to another TCP after two months (OR 1.96), compared to those without CI [60].

Beyond the percentage of participants discharged home, four studies [47, 48, 51, 54] specified the percentage of participants who had a successful community discharge, that is, they were discharged from TCP to the community within 90–100 days of TCP admission [47, 51, 54] and, within 30 days of discharge from TCP, they were not hospitalized [47, 48, 51, 54], were not admitted to a nursing home [47, 48, 51], and did not die [47, 48, 51]. Between 24.6% [47] and 68.0% [48] of older adults with any form of CI, compared to 58.1% [47] and 62.9% [54] of older adults with no CI had a successful community discharge. Furthermore, only one study [51] looked at both successful discharge (57.4% of older adults with dementia) and functional decline. Improvement in functional status was found in 28.4% of participants with dementia, while 45.5% had no improvement, and 26.1% had missing data [51].

### Quality of studies

Although the majority of the studies were rated moderate to good quality, the heterogeneity of the outcome measures, measurement times, and patient populations as well as study designs in the included studies, in addition to the lack of RCTs in this review precluded meaningful meta-analysis. Furthermore, as all the studies included in this review were observational, there is a risk of bias due to lack of randomization. Therefore, only determination of associations was possible.

## Discussion

The results of this systematic review reveal that TCPs help improve outcomes for older adults with and without CI [29, 30, 42, 44, 51, 53–55, 62]. However, a greater percentage of participants without CI had improvements in ADLs and better patient and health services outcomes compared to those with CI. In terms of discharge destination, older adults with CI were more often discharged home than to long-term care, however, a greater percentage of participants without CI were discharged home [38, 45, 47, 54, 55, 60]. There was also a wide range in the percentage of older adults with CI who had a successful discharge home [47, 48, 51, 54].

A meta-analysis by Hang et al. [19] on community-dwelling older adults in TCPs found a significant improvement in modified Barthel Index functional score between admission and discharge (pooled mean difference of 17.65 points (95% confidence interval [5.68, 29.62], *p* = 0.004). However, Hang et al.’s meta-analysis did not focus on community-dwelling older adults with CI; instead, they focused on community-dwelling older adults in general. In this review, community-dwelling older adults with CI in TCPs also had an improvement in ADLs which was reported in eight of 12 studies. However, the study by Miu, Chan, & Kok [30] used the modified Barthel Index and found a smaller increase in functional score for those with dementia than that reported in Hang et al. [19]. Similarly, overall, functional improvement found in this present review was smaller for older adults with CI than for those without CI.

Although participants with CI had less functional improvement in TCPs than those without CI, it is likely that having older adults with CI who remain in hospitals once their acute medical condition is treated is not ideal. A previous review by Hartley et al. and an article by Pedone and colleagues demonstrated that having CI on hospital admission is a risk factor for functional decline [7, 65]. Therefore, the improvements in functional status in TCPs indicate that these settings may be a better option for older adults with CI, rather than remaining in acute care where there is the risk of functional decline.

The meta-analysis by Hang and colleagues found that 80% of older adult participants in TCPs were discharged home [19]; however, this is a stark difference from the 25.9–68% of older adults with CI discharged home in the current review. Prior research on hospitalized older adults who have CI found that living alone and having responsive behaviours (e.g., verbal or physical behaviours related to care provision) at admission were negatively associated with discharge home [66]. Therefore, behavioural and psychological symptoms may influence discharge outcomes [66]. Thus, the lower percentage of participants with dementia being discharged home from TCPs may be due a variety of factors; future research to determine the facilitators and barriers to being discharged home is needed. In terms of discharge to long-term care, a review by Fogg and colleagues found that between 8.3–22.4% of hospitalized patients with CI (mild CI, CI, dementia) compared to 3.5–19.4% with no CI (*p* = 0.001), transitioned to nursing homes post TCP [9], slightly less than what was found in the present review (4.1–40%). Moreover, these reviews highlight the need for specialized interventions to increase the percentage of older adults with CI who can be discharged to their home.

Furthermore, given the role of TCPs in improving safety of transitions, there is a need to consider the difference between promoting increased discharge home and promoting successful discharge home. Discharged home means that the older adults are not discharged to a different facility such as long-term care. Successful discharge was defined slightly differently by each of the four studies; it means that, within 30 days of discharge to home, the older adult avoids re-hospitalization [47, 48, 51, 54], admission to nursing home [47, 48, 51], and death [47, 48, 51]. Moreover, adverse events such as falls [67], functional decline [68], and medication-related adverse events [69] can all contribute to re-hospitalization risk. Given the percentage of older adults with CI who were re-hospitalized post TCP [48, 51, 54, 55] as well as the wide range for the percentage of older adults with CI who had a successful discharge home [47, 48, 51, 54], there is a need for interventions to promote safe, successful transitions to the home that reduces the risk of adverse events. Indeed, Toles and colleagues’ study involving persons with dementia, their care partners, TCP staff, and home health nurses found that transitions from TCPs to home involve several important and unique care needs [70]. These included care planning specific to the needs of persons with dementia; the need to prepare care partners to manage dementia symptoms at home; difficulty connecting care partners and older adults with dementia to community supports; and the need for support for care partners to address their own needs [70]. Other considerations to reduce adverse events that can result in re-hospitalization include medication management [69], addressing information needs of care partners, such as providing instructions on how to transfer the older adults in and out of a wheelchair, and scheduled post-TCP medical follow-up appointments [71].

This present review also demonstrates that various health care professionals are involved in the different TCP models of care. One model which has resulted in positive functional status and patient outcomes included an interprofessional team that focused on a reablement approach [29]. A reablement approach in older adults with dementia involves maintaining function for as long as possible, regaining lost function when it is possible to do so, and adapting when lost function cannot be regained [72]. In Lee et al.’s prospective cohort study, a TCP with a physical reablement program consisting of a comprehensive geriatric assessment, ADL training, exercises, and care plans with functional goals resulted in improvements in all patient outcome measures, including functional status, instrumental ADLs, and cognitive function for older adults with dementia [29]. However, discharge destination was not an outcome assessed in this study. The reablement approach could be adopted by TCPs and tested for the impact on both functional status and discharge destination in future studies. This model could also be compared and evaluated with other models in order to determine best practices for this population.

### Implications for practice, policy, and future research

This review provided supportive evidence regarding the impact of TCPs on improvements in ADLs, patient and health services outcomes, and the greater percentage of discharges home than to long-term care for older adults with CI. However, practitioners and policymakers should take into consideration the level of evidence from this review, given the lack of RCTs and quasi-experimental studies.

#### Practice

In practice, health care teams can consider TCPs as possible discharge destinations for older adults with CI who are not yet ready to be discharged home. Given that participants with CI gained smaller improvements in ADLs, it is critical to identify patients with any form of CI, so that additional or specialized resources, such as recreational therapy, behavioural supports, or Geriatric Psychiatry, can be allocated to help improve their outcomes. Moreover, in order to improve the safety of transitions, TCPs should consider including informational support to care partners on dementia care, connecting care partners and older adults with CI with community resources, and providing support for the needs of care partners.

#### Policy

Given the findings of improved ADLs in older adults, TCPs may be better settings than acute care for this population and as such should be transferred to these settings as soon as they are medically stable. Thus, policymakers involved in the creation or modification of future TCPs should ensure timely access to TCPs for persons with CI. Policymakers should also consider the rate of successful discharges for older adults with CI as a quality measure for TCPs.

#### Research

Although this review showed that there were improvements in ADLs for older adults with CI associated with TCPs, causality cannot be implied due to the lack of RCT evidence.

Thus, there is a need for RCTs to be conducted to compare TCPs for older adults with CI with usual care, and to assess whether improvements in functional status translate into an increase in the percentage of older adults with CI who are discharged home. Second, there is a need to develop and test reablement interventions in TCPs that focus on maintaining and improving functional status in older adults with CI; a reablement program may be one solution [29]. Third, further studies are required to assess and measure other health outcomes such as complex functioning required to perform IADLs, in addition to the performance of ADLs, since living in the community requires more than just physical capabilities [73]. Fourth, future studies should utilize standardized functional status measurement tools among older adults with CI in TCPs in order to facilitate meta-analyses. Fifth, studies should include both discharge destination and rate of successful discharge to community as outcome measures, to demonstrate effectiveness of TCPs. Finally, there is a need for quantitative and qualitative studies to determine the factors, such as social supports and resources, barriers, and facilitators, that can have an impact on discharge destination for this population, and for intervention studies to address the barriers.

### Strengths and limitations

Strengths of this review include registering and following a PROSPERO protocol and having studies that were of moderate to good quality. As well, there were large sample sizes in the included studies, increasing the confidence placed in the results of the review. In addition, the search strategy was developed in consultation with a library information sciences expert, promoting comprehensiveness. Furthermore, the time frame for the study was from inception to present, thereby promoting the inclusion of all applicable studies. A limitation of the review was that only studies reported in English were included, which may limit generalizability of the findings. Additional research studies may have been missed due to the exclusion of non-English language documents. Another limitation is that there are differences between the TCPs in different countries; SNFs in the US have differences compared to transition care programs in Australia and transitional care programs in Canada. As well, a limitation was the variability in outcome measurement tools and outcome assessment times, as well as patient populations, which prevented meta-analysis.

## Conclusions

This systematic review showed that overall facility-based TCPs are associated with improvements in ADLs, and a larger percentage of older adults with CI were discharged home compared to long-term care. However, functional status and discharge destination outcomes for older adults with CI were worse than for those without CI. There is a need for RCTs to determine the effectiveness of TCPs in improving functional status and other patient outcomes and a specific call to understand interventions to increase the percentage of older adults with CI who are discharged home.

## Supplementary Information

**Additional file 1. **Full Database Search Strategies and Search Results.

**Additional file 2. **Registry Search Strategies, Results and the Dates the Registries were Last Searched.

**Additional file 3: Table S1. **Risk of Bias Assessment for Cohort Studies; **Table S2.** Risk of Bias Assessment for Cross-Sectional Studies; **Table S3. **Characteristics of Included Studies; **Table S4.** Patient Outcomes; **Table S5.** Health Services Outcomes.

**Additional file 4. **Extracted Data.

## Data Availability

All data generated or analysed during this study are included in this published article [and its supplementary information files].

## Abbreviations

CI: Cognitive impairment
TCP: Transitional Care Program
RCT: Randomized controlled trial
ADLs: Activities of daily living
PRISMA: Preferred Reporting Items for Systematic Reviews and Meta-Analyses
aOR: Adjusted odds ratio
OR: Odds ratio
