# Correction: The impact of facility-based transitional care programs on function and discharge destination for older adults with cognitive impairment: a systematic review

**DOI:** 10.1186/s12877-023-03812-6

**Published:** 2023-03-30

**Authors:** Alexia Cumal, Tracey J. F. Colella, Martine T. Puts, Poonam Sehgal, Sheryl Robertson, Katherine S. McGilton

**Affiliations:** 1 KITE Research Institute, Toronto Rehabilitation Institute – University Health Network, Bickle Centre, 550 University Avenue, Suite 236B, Toronto, ON M5G 2A2 Canada; 2 Lawrence S. Bloomberg Faculty of Nursing, University of Toronto, 155 College Street, Suite 130, Toronto, ON M5T 1P8 Canada; 3 KITE Research Institute, Toronto Rehabilitation Institute – University Health Network, Cardiovascular Prevention & Rehabilitation Program, 347 Rumsey Road, Suite 250, Toronto, ON M4G 1R7 Canada; 4 Home and Community Care Support Services Central East, 100 Consilium Place Suite 801, Scarborough, ON M1H 3E3 Canada

**Correction: BMC Geriatrics 22, 854 (2022)**

**https://doi.org/10.1186/s12877-022-03537-y**

After publication of this article [1], the authors reported that in Table 1 and Fig. 2 some errors occurred. Updated versions of Table 1 and Fig. 2 are shown below.

**Table 1:** Participant Characteristics

Author Year	Number of patients	How CI was defined	Age (years) mean +/- SD	Females n (%)	Ethnicity %	Cognitive Status Score Mean +/- SD	Charlson Comorbidity Index Score mean +/- SD
Abrahamsen 2016	Number of patients with an MMSE <24: 206 (29% of total participants)	CI defined using the Norwegian version of the MMSE (score range 0-30, score <24 is a sign of cognitive impairment)	Mean age of total sample 85, min-max (70-102)	Of total sample: n=656 (68)	NR	Median MMSE of total sample: 26, min-max (8-30)-	Of total sample: >5 diagnoses, n(%) 567 (62)
Bardenheier 2021	Total number of persons with ADRD: n=2134798	ADRD was identified by the (CCW) flag in the Medicare beneficiary summary file	January 1 to September 30, 2015: mean age 84.6	January 1 to September 30, 2015: 141475 (64.9)	January 1 to September 30, 2015 Black % 8.6 Other race % 4.6	NR	NR
Burke 2021	Total number with dementia: 830,524 (34.3%) With dementia, used in matched cohort: 513,424 (34.3%)	Identification of dementia determined via coding in datasets (MDS, MBSF, MedPAR)	82.8 (8.1)	320,611 (62.4%)	White: 446,090 (86.9%) Black: 46,606 (9.1%) Asian: 5976 (1.2%) Other: 5059 (1.0%) Hispanic: 7951 (1.5%)	CFS: Cognitively intact: 260,736 (50.8%) Mild impairment: 180,667 (35.2%) Moderate impairment: 61,041 (11.9%) Severe impairment: 10,980 (2.1%)	3.0 (2.4)
Cations 2020	Individuals in residential care settings with dementia: n=10701 (25.4%)	Dementia was determined from the aged care eligibility assessment and dispensing of medications prescribed for Alzheimer’s disease in the 6 months before hospitalization	For all participants in residential TCP: 83.2 (7.3)	For all participants in residential TCP: 25999 (61.7)	NR	NR	For all participants in residential TCP: Comorbidities (median, IQR) 0: 1948 (4.6) 1-4: 15336 (36.4) 5-9: 21971 (51.1) 10+:9210 (6.90)
Chong 2012	Persons with dementia and behavioural disturbances: n=31 (16.9%)	Dementia not defined	For all participants: 81.1 (+/-8.1)	For all participants: 100 (54.6)	For all participants: Chinese 83.6%; Malay 8.2%; Indian 7.1%; Others 1.2%	NR	For all participants: Modified CCI 1.6 ± 1.3; Severity of illness score index 2.0 ± 0.7
Downer 2022	Mild CI: n=120830 Moderate to severe CI: n=74183	Cognitive status was categorized as none, mild, and moderate or severe impairment according to the Cognitive Function Scale	For total sample: n(%) Age 66-70: 73721 (12.0); Age 71-75: 99050 (16.1); Age 76-80: 116526 (18.9); Age 81-85: 124364 (20.2); Age ≥86: 202412 (32.9)	For total sample: 394629 (64.1)	For total sample: White 86.0% Black 7.1% Hispanic 3.6% Other 3.3%	For total sample: CI: n (%) None 421060 (68.3) Mild 120830 (19.6) Moderate to severe 74183 (12.0)	NR
Hang 2021	CI: n=73	CI measured using Mini Mental State Examination (MMSE), scored ≤ 23/30 at admission	For all participants: 84.2+/-8.3	For all participants: 103 (60.9)	NR	MMSE Discharged Home n(%) vs Other n(%) Yes 21 (42.9), 66 (63.5) p=0.016*	For all participants: NR
Intrator 2021	With dementia: n=1091 No/mild CI: n=9808 Moderate/high CI: n=4979	Dementia status from the MDS assessment at the start of the CLC episode, MDS item I4800; Cognitive function status from MDS items B0700, C0100, C0500, C1000	With dementia: 77.99 (10.53) No/mild CI: 68.00 (10.87) Moderate/high CI: 72.79 (11.68)	With dementia: 1.7% With dementia and No/Mild CI: 2.0% With dementia and moderate/high CI: 1.7% With no dementia: 4.3% With no dementia and No/Mild CI: 4.8% With no dementia and moderate/high CI: 3.4% %Female among Veterans in cohort with no/mild CI: 4.7% %Female among Veterans in cohort with moderate/high CI: 3.0% Overall %Female in this cohort was 4.1%	American Indian or Alaska Native With dementia: 0.53%; No/mild CI: 0.86%; Moderate/high CI: 1.12% Asian: With dementia: 0.27% No/mild CI: 0.33% Moderate/high CI: 0.25% Black / African American: With dementia: : 21.26%; No/mild CI: 18.70%; Moderate/high CI: 20.69% Hispanic or Latino: With dementia: 5.67%; No/mild CI: 4.02%; Moderate/high CI: 4.25% Native Hawaiian or Other Pacific Islander: With dementia: 0.27%; No/mild CI: 0.49%; Moderate/high CI: 0.52% White: With dementia: 71.74%; No/mild CI: 75.52%; Moderate/high CI: 73.00% Unknown: With dementia: 0.27%; No/mild CI: 0.09%; Moderate/high CI: 0.15%	No/mild CI: n=9808 Moderate/high CI: n=4979	With dementia: 1.66 (SD 0.31) No/mild CI: 1.73 (SD 0.20) Moderate/high CI: 1.70 (SD 0.24)
Kosar 2017	Participants with delirium: n=242121	Delirium identified using the CAM criteria in the MDS resident assessment; Dementia status from the MDS admission assessment	83.2 (8.1)	141451 (58)	non-white race 16%	CFS Score n (%): mild impairment 45240 (19); moderate impairment 132759 (55); severe impairment 43844 (18) dementia n (%): 133496 (55)	CCI score: 2.6 (2.0)
Lee 2008	Number of participants with CI not reported	CI measured by 7-category MDS CPS	For all participants: 82.34±7.7 (range 65-102)	For all participants: 67%	NR	For all participants: 1.68 (1.7) range 0-6	NR
Lee 2011	Participants with dementia: n=139	Dementia defined as MMSE<14 with education years<6 years; MMSE<24 with education years ≥6 years	82.6±5.9	0 (0%) (All participants were male)	NR	MMSE score 5.9±3.7	NR
Lei 2022	Veterans with dementia in PAC n=8317	Dementia identified via ICD-9 coding	Aged 66-74: 16.9% Aged 75-84: 42.4% Aged 85+: 40.7%	166 (2%)	Non-Hispanic white 76.7%	NR	NR
Loomer 2019	Participants with mild CI n=45064; Moderate CI n=28979; Severe CI n=4117; Total number of participants with CI: n=78160	CI defined using the CFS in the MDS v.3.0; Alzheimer’s disease/dementia identified if it was an admission diagnosis	Ages 65-74 n (%) mild CI 8976 (19.9); moderate CI 3976 (13.7); severe CI 728 (17.7); ages 75-84 n (%) mild CI 15246 (33.8); moderate CI 9358 (32.3); severe CI 1395 (33.8); ages 85-90 n (%) mild CI 9485 (21.1); moderate CI 7147 (24.7); severe CI 750 (18.2); 90+ years n (%) mild CI 8598 (19.1); moderate CI 7241 (25.0); severe CI 778 (18.9)	NR	NR	Alzheimer’s Disease/Dementia n (%) Among participants with mild CI 10941 (24.3) Among participants with moderate CI 17191 (59.3) Among participants with severe CI 2670 (64.9)	NR
Lueckel 2018	n (%) Participants with mild CI n=22043 (25); Moderate CI: n=20282 (23); Severe CI: n=5144 (6)	CI defined using the CFS	For all participants: 83.9 (7.5)	For all participants: 55418 (63)	For all participants: Nonwhite race (9)	CFS Score, *n* (%) 2: Mild impairment 22043 (25); 3: Moderate impairment 20282 (23); 4: Severe impairment 5,144 (6)	For all participants: Deyo-Charlson Comorbidity Index, mean (SD) 1.2 (1.4)
Madrigal 2021	Participants with delirium: n=882	Delirium determined using the MDS 3.0 CAM; dementia determined via ICD-9 coding	81.0 (9.3) P<0.001	n=31 (3.5)	n (%) White: 710 (80.5) Black: 138 (15.6) Hispanic 145 (1.6) Other: 20 (2.3)	Dementia, n (%): 525 (59.5)	Elixhauser comorbidity index, mean (SD) 4.3 (2.8)
Marcantonio 2003	Participants with delirium symptoms: n=126	Delirium assessed in the MDS; dementia assessed in MDS from the list of MDS-based comorbidities	Participants with delirium symptoms: 79 +/-8	Participants with delirium symptoms: 77 (61)	Participants with delirium symptoms: Caucasian, 111 (88)	Participants with delirium symptoms: Dementia diagnosis (n (%)): 18 (14)	Participants with delirium symptoms: 2.1 +/- 1.0
Marcantonio 2005	Participants with delirium: n=188; Participants with subsyndromal delirium: n=246; Total number of persons with CI: n=434	Participants classified as having delirium if they met full CAM criteria; classified as having subsyndromal delirium if they have one or more CAM criteria	Participants with delirium: 83.3±7.4 Participants with subsyndromal delirium: 82.5±7.7	Participants with delirium: 127 (68) Participants with subsyndromal delirium: 167 (68)	Participants with delirium: White, not Hispanic: 146 (87) Participants with subsyndromal delirium: White, not Hispanic: 143 (89)	Participants with delirium: MMSE score 12.7 +/-7.0; MDAS score: 12.6+/-4.4 Participants with subsyndromal delirium: MMSE score 18.8 +/- 6.1; MDAS score: 7.1+/-3.1	Participants with delirium: CCI score: 1.2+/- 1.2 Participants with subsyndromal delirium: CCI score: 1.4+/-1.3
Mazzola 2022	With dementia: n=98 With delirium: n=58	Dementia via history of pre-existing dementia and using the MMSE. Delirium was assessed with the 4AT test.	For whole sample: 78.2 (11.6)	For whole sample: 202 (49.7%)	NR	Mini-Mental State Examination Mean (SD) 21.3 (7.5)	For whole sample: 3.0 (1.9)
Miu 2016	Community-dwelling participants with CI: n=78; with dementia: n=31	CI was determined using the MMSE; delirium via the CAM-CR; dementia identified through medical records	Community-dwelling participants with cognitive impairment: 83.9±6.5; with dementia: 84.2±6.6	Community-dwelling participants with cognitive impairment: 42 (54); with dementia: 19 (61)	NR	NR	Community-dwelling participants with CI: mean CCI =2.36±1.55; with dementia: mean CCI = 2.52±1.23
Nakanishi 2016	Participants who had dementia: n=2483	Dementia diagnosis was determined through the ICD-10	For total sample, based on discharge destination: home: 84±8.3 hospital: 85.2 ±8.2 Facility: 84.1 ±8.2 Death: 88.6±7.4	For total sample, based on discharge destination: sex, male, n (%) home: 763 (26.3) hospital: 1453 (32.8) facility: 538 (26.0) death: 193 (32.6)	NR	Cognitive impairment^ǂ^ (range 1-6), mean±SD For total sample, Based on discharge destination: home: 2.6 ±1.4 hospital: 3.0 ±1.1 facility: 2.8 ±1.2 death: 3.2 ±1.0	NR
Simning 2022	With dementia: n=10426	Dementia determined through SNF admission MDS and ICD-9 codes	Discharged home: 81.7± 8.5 Not discharged home: 83.0± 89.0	Discharged home: 66.40% Not discharged home: 60.90%	Discharged home: White: 84.10% Not discharged home: White: 79.3%	Discharged home: % with dementia: 16.20% Not discharged home % with dementia: 31.9%	Discharged home: Number of diagnoses: 6±3.7 Not discharged home: Number of diagnoses: 5.9±3.6
Wysocki 2015	Moderately impaired (19.3%) n= 171,152 Severely impaired (9.7%) n=86019 Dementia 12.9%) n=114396; Any signs of delirium: =25717	CI defined using the CPS; Dementia determined if participant had a diagnosis of dementia; Signs of delirium were based on the CAM items	Mean age of total sample: 77.4+/-12.3 NR for patients with CI or dementia	For total sample: 64.4% NR for patients with CI or dementia	Race, not white for total sample 15.8% NR for patients with CI or dementia	For whole sample: % with Dementia: 12.9; % with moderately impaired cognition for whole sample: 19.3; % with severely impaired cognition for whole sample: 2.9; % with Alzheimer’s disease for whole sample: 2.9	NR

*CI* cognitive impairment, *N* number, *ADRD* Alzheimer’s Disease and related dementias, *MMSE* Mini-Mental State Examination, *CCI* Charlson Comorbidity Index, *MDAS* Memorial Delirium Assessment Scale, ^*ǂ*^ Cognitive impairment scale not specified, however, authors report that it demonstrates consistency with scores on the MMSE and Hasegawa Dementia Scale-Revised, *IQR* Interquartile range, *NR* Not reported, *CFS* Cognitive Function Scale, *CI* cognitive impairment, *CCW* Chronic Condition Data Warehouse, *CAM* Confusion Assessment Method, *MDS* Minimum Data Set, *ICD-9* International Classification of Disease, Ninth Revision, *CAM-CR* Chinese version of the CAM, *ICD-10* International Classification of Disease, Tenth Revision, *SPMSQ* short portable mental status questionnaire, *CPS* Cognitive Performance Scale, *MBSF* Master Beneficiary Summary File, *SD* standard deviation

Results are for persons with CI (CI, dementia, delirium) only, unless otherwise stated

**Fig. 2:**
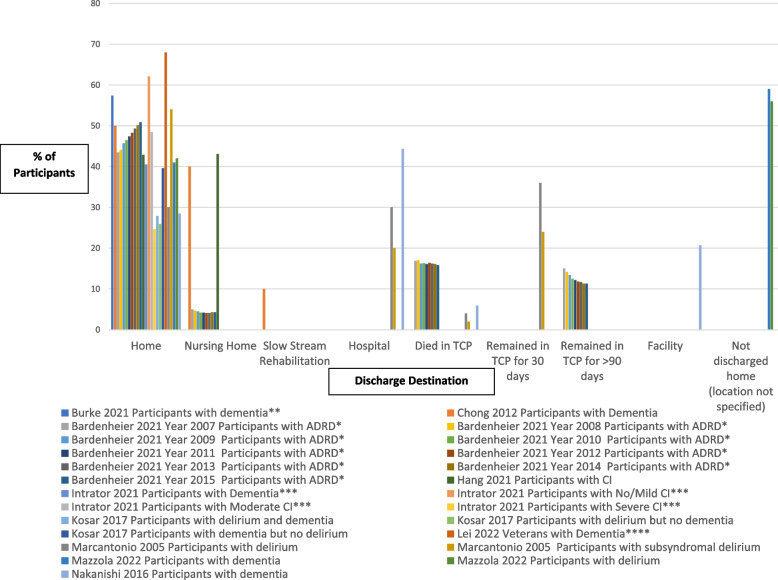
Percentage of participants with CI discharged by destination. ADRD=Alzheimer’s Disease and Related Dementias; CI=Cognitive impairment; TCP=Transitional Care Program; * = Outcome is Successful Discharge (defned as being discharged alive from a skilled nursing facility (SNF) to the community within 90 days of SNF admission without subsequent inpatient healthcare utilization for 30 continuous days; ** = Outcome is community discharge rate (metric used on Nursing Home Compare is the rate of benefciaries who are able to leave the SNF by 100 days after hospital discharge and remain in the community (i.e.,alive and outside the hospital and nursing home) for at least 30 days after SNF discharge; *** = Outcome is Successful Discharge (discharge to community within 100 days of a nursing home admission, defned as: Discharge to the community within 100 days (allowing for interim discharges from Community Living Center to hospital if the Minimum Data Set noted that return was anticipated, observation stays, and emergency room use), and no unplanned admissions to a hospital, a nursing home or observation stay, and not dying within 30 days following discharge; **** = Outcome is Successful Discharge to the community (During the 30 subsequent days the veteran did not die, was not readmitted to a hospital for an unplanned inpatient stay, and was not admitted to a nursing home): No * indicates that it is the percentage of older adults with CI discharged home, and does not specify that it needs to have been a “successful” discharge as defned in the 4 studies with a *

The original article [1] has been corrected.
