# Supplementary material for: The impact of facility-based transitional care programs on function and discharge destination for older adults with cognitive impairment: a systematic review
Source: BMC Geriatr. 2022 Nov 14;22:854. doi: 10.1186/s12877-022-03537-y (PMC9661763; doi:10.1186/s12877-022-03537-y)
Supplement: Supplementary file 1 — Additional file 1. Full Database Search Strategies and Search Results. [file 12877_2022_3537_MOESM1_ESM.pdf]

Additional file 1: Full Database Search Strategies and Search Results

OVID Medline Search Strategy

Ovid MEDLINE: Epub Ahead of Print, In-Process & Other Non-Indexed Citations, Ovid MEDLINE® Daily and Ovid MEDLINE® 1946-Present

Search done on: July 15, 2021

Search dates: Inception to present (July 14, 2021)

1. (Transition* adj3 (care* or unit* or bed* or program* or ward* or setting* or facilit* or service* or model* or centre* or center*)).tw,kf.
2. Transitional care/
3. (Intermediate adj3 (unit* or care or bed* or program* or ward* or setting* or facilit* or service* or model* or centre* or center*)).tw,kf.
4. Intermediate care facilities/
5. ((Subacute or sub-acute) adj3 (unit* or care or bed* or program* or ward* or setting* or facilit* or service* or model* or centre* or center*)).tw,kf.
6. Subacute care/
7. ((Postacute or post-acute) adj3 (unit* or care or bed* or program* or ward* or setting* or facilit* or service* or model* or centre* or center*)).tw,kf.
8. ((Post acute) adj3 (unit* or care or bed* or program* or ward* or setting* or facilit* or service* or model* or centre* or center*)).tw,kf.
9. ((Skilled Nursing) adj3 (unit* or bed* or program* or ward* or setting* or facilit* or service* or model* or centre* or center*)).tw,kf.
10. Skilled nursing facilities/
11. (Restor* adj3 (unit* or care or bed* or program* or ward* or setting* or facilit* or service* or model* or centre* or center*)).tw,kf.
12. (Convalesc* adj3 (unit* or care or bed* or program* or ward* or setting* or facilit* or service* or model* or centre* or center* or home* or hospital*)).tw,kf.
13. Convalescence/
14. or/1-13
15. (Old* or aged or aging).tw,kf.
16. (Centenarian* or nonagenarian* or octogenarian* or geriatr* or gerontol* or senescen* or septuagenarian* or pensioner* or senile).tw,kf.
17. Senior*.tw,kf.
18. Elder*.tw,kf.
19. Aged/
20. "Aged, 80 and over"/
21. or/15-20
22. (Cognit* adj3 impair*).tw,kf.
23. (Mild neurocognitive disorder*).tw,kf.
24. Cognitive dysfunction/
25. (Major neurocognitive disorder*).tw,kf.

26. Dement*.tw,kf.
27. Dementia/
28. Alzheimer disease/
29. Alzheimer*.tw,kf.
30. AIDS Dementia Complex/
31. Dementia, Vascular/
32. Dementia, Multi-Infarct/
33. Lewy body disease/
34. Deliri*.tw,kf.
35. Delirium/
36. or/22-35
37. 14 and 21 and 36
    Result: 1102 articles

OVID Embase Search Strategy

Embase Classic+Embase 1947 to 2021 July 14

Search done on: July 15, 2021

Search dates: Inception to present (July 14, 2021)

1. (Transition* adj3 (care* or unit* or bed* or program* or ward* or setting* or facilit* or service* or model* or centre* or center*)).tw,kw.
2. Transitional care/
3. (Intermediate adj3 (unit* or care or bed* or program* or ward* or setting* or facilit* or service* or model* or centre* or center*)).tw,kw.
4. Nursing home/
5. ((Subacute or sub-acute) adj3 (unit* or care or bed* or program* or ward* or setting* or facilit* or service* or model* or centre* or center*)).tw,kw.
6. Subacute care/
7. ((Postacute or post-acute) adj3 (unit* or care or bed* or program* or ward* or setting* or facilit* or service* or model* or centre* or center*)).tw,kw.
8. ((Post acute) adj3 (unit* or care or bed* or program* or ward* or setting* or facilit* or service* or model* or centre* or center*)).tw,kw.
9. ((Skilled Nursing) adj3 (unit* or bed* or program* or ward* or setting* or facilit* or service* or model* or centre* or center*)).tw,kw.
10. (Restor* adj3 (unit* or care or bed* or program* or ward* or setting* or facilit* or service* or model* or centre* or center*)).tw,kw.
11. (Convalesc* adj3 (unit* or care or bed* or program* or ward* or setting* or facilit* or service* or model* or centre* or center* or home* or hospital*)).tw,kw.
12. Convalescence/
13. or/1-12
14. (Old* or aged or aging).tw,kw.
15. (Centenarian* or nonagenarian* or octogenarian* or geriatr* or gerontol* or senescen* or septuagenarian* or pensioner* or senile).tw,kw.
16. Senior*.tw,kw.
17. Elder*.tw,kw.
18. Aged/
19. Very Elderly/
20. or/14-19
21. (Cognit* adj3 impair*).tw,kw.
22. (Mild neurocognitive disorder*).tw,kw.
23. Cognitive deficit/
24. (Major neurocognitive disorder*).tw,kw.
25. Dement*.tw,kw.
26. Dementia/
27. Alzheimer disease/

28. Alzheimer*.tw,kw.
29. Frontotemporal dementia/
30. Frontal variant frontotemporal dementia/
31. HIV associated dementia/
32. "Mixed depression and dementia"/
33. Multiinfarct dementia/
34. Pick presenile dementia/
35. Presenile dementia/
36. Senile dementia/
37. Diffuse Lewy body disease/
38. Deliri*.tw,kw.
39. Delirium/
40. or/21-39
41. 13 and 20 and 40
    Result: 9635 articles

OVID APA PsycInfo Search Strategy

APA PsycInfo 1806 to July Week 1 2021

Search done on: July 15, 2021

Search dates: Inception to present (July 14, 2021)

1. (Transition* adj3 (care* or unit* or bed* or program* or ward* or setting* or facilit* or service* or model* or centre* or center*)).tw
2. (Intermediate adj3 (unit* or care or bed* or program* or ward* or setting* or facilit* or service* or model* or centre* or center*)).tw
3. ((Subacute or sub-acute) adj3 (unit* or care or bed* or program* or ward* or setting* or facilit* or service* or model* or centre* or center*)).tw
4. ((Postacute or post-acute) adj3 (unit* or care or bed* or program* or ward* or setting* or facilit* or service* or model* or centre* or center*)).tw
5. ((Post acute) adj3 (unit* or care or bed* or program* or ward* or setting* or facilit* or service* or model* or centre* or center*)).tw
6. ((Skilled Nursing) adj3 (unit* or bed* or program* or ward* or setting* or facilit* or service* or model* or centre* or center*)).tw
7. (Restor* adj3 (unit* or care or bed* or program* or ward* or setting* or facilit* or service* or model* or centre* or center*)).tw
8. (Convalesc* adj3 (unit* or care or bed* or program* or ward* or setting* or facilit* or service* or model* or centre* or center* or home* or hospital*)).tw
9. or/1-8
10. (Old* or aged or aging).tw
11. (Centenarian* or nonagenarian* or octogenarian* or geriatr* or gerontol* or senescen* or septuagenarian* or pensioner* or senile).tw
12. Senior*.tw
13. Elder*.tw
14. Geriatric patients/
15. or/10-14
16. (Cognit* adj3 impair*).tw
17. (Mild neurocognitive disorder*).tw
18. Cognitive Impairment/
19. Mild Cognitive Impairment/
20. (Major neurocognitive disorder*).tw
21. Dement*.tw
22. Dementia/
23. "Alzheimer's Disease"/
24. Alzheimer*.tw
25. AIDS Dementia Complex/
26. Dementia with Lewy Bodies/
27. Presenile Dementia/
28. Senile Dementia/

29. Semantic Dementia/
30. Vascular Dementia/
31. Deliri*.tw
32. Delirium/
33. or/16-32
34. 9 and 15 and 33

Result: 334 articles

CINAHL Plus Search Strategy

CINAHL Plus with Full Text

Search done on: July 15, 2021

Search dates: Inception to present (July 14, 2021)

1. TI ((Transition* N3 (care* or unit* or bed* or program* or ward* or setting* or facilit* or service* or model* or centre* or center*))) OR AB ((Transition* N3 (care* or unit* or bed* or program* or ward* or setting* or facilit* or service* or model* or centre* or center*)))
2. MH Transitional Care
3. TI ((Intermediate N3 (unit* or care or bed* or program* or ward* or setting* or facilit* or service* or model* or centre* or center*))) OR AB ((Intermediate N3 (unit* or care or bed* or program* or ward* or setting* or facilit* or service* or model* or centre* or center*)))
4. TI (((Subacute or sub-acute) N3 (unit* or care or bed* or program* or ward* or setting* or facilit* or service* or model* or centre* or center*))) OR AB (((Subacute or sub-acute) N3 (unit* or care or bed* or program* or ward* or setting* or facilit* or service* or model* or centre* or center*)))
5. MH Subacute care
6. TI (((Postacute or post-acute) N3 (unit* or care or bed* or program* or ward* or setting* or facilit* or service* or model* or centre* or center*))) OR AB (((Postacute or post-acute) N3 (unit* or care or bed* or program* or ward* or setting* or facilit* or service* or model* or centre* or center*)))
7. TI (((Post acute) N3 (unit* or care or bed* or program* or ward* or setting* or facilit* or service* or model* or centre* or center*))) OR AB (((Post acute) N3 (unit* or care or bed* or program* or ward* or setting* or facilit* or service* or model* or centre* or center*)))
8. TI (((Skilled Nursing) N3 (unit* or bed* or program* or ward* or setting* or facilit* or service* or model* or centre* or center*))) OR AB (((Skilled Nursing) N3 (unit* or bed* or program* or ward* or setting* or facilit* or service* or model* or centre* or center*)))
9. MH Skilled Nursing Facilities
10. TI ((Restor* N3 (unit* or care or bed* or program* or ward* or setting* or facilit* or service* or model* or centre* or center*))) OR AB ((Restor* N3 (unit* or care or bed* or program* or ward* or setting* or facilit* or service* or model* or centre* or center*)))
11. TI ((Convalesc* N3 (unit* or care or bed* or program* or ward* or setting* or facilit* or service* or model* or centre* or center* or home* or hospital*))) OR AB ((Convalesc* N3 (unit* or care or bed* or program* or ward* or setting* or facilit* or service* or model* or centre* or center* or home* or hospital*)))
12. S1 OR S2 OR S3 OR S4 OR S5 OR S6 OR S7 OR S8 OR S9 OR S10 OR S11
13. TI ((Old* or aged or aging)) OR AB ((Old* or aged or aging))
14. TI ((Centenarian* or nonagenarian* or octogenarian* or geriatr* or gerontol* or senescen* or septuagenarian* or pensioner* or senile)) OR AB ((Centenarian* or nonagenarian* or

octogenarian* or geriatr* or gerontol* or senescen* or septuagenarian* or pensioner* or senile))

15. TI Senior* OR AB Senior*
16. TI Elder* OR AB Elder*
17. MH Aged
18. MH "Aged, 80 and over"
19. S13 OR S14 OR S15 OR S16 OR S17 OR S18
20. TI (Cognit* N3 impair*) OR AB (Cognit* N3 impair*)
21. TI (Mild neurocognitive disorder*) OR AB (Mild neurocognitive disorder*)
22. MH "Mild Cognitive Impairment"
23. TI (Major neurocognitive disorder*) OR AB (Major neurocognitive disorder*)
24. TI Dement* OR AB Dement*
25. MH Dementia
26. MH "Alzheimer’s disease"
27. TI Alzheimer* OR AB Alzheimer*
28. MH "AIDS Dementia Complex"
29. MH "Dementia, Vascular"
30. MH "Dementia, Multi-Infarct"
31. MH "Lewy Body Disease"
32. MH "Dementia, Presenile"
33. MH "Dementia, Senile"
34. TI Deliri* OR AB Deliri*
35. MH Delirium
36. S20 OR S21 OR S22 OR S23 OR S24 OR S25 OR S26 OR S27 OR S28 OR S29 OR S30 OR S31 OR S32 OR S33 OR S34 OR S35
37. S12 AND S19 AND S36
    Result: 825 articles

Cochrane Search Strategy

Cochrane Central Register of Controlled Trials

Issue 6 of 12, June 2021

Search done on: July 15, 2021

Search dates: Inception to present (July 14, 2021)

1. (Transition* NEAR/3 (care* or unit* or bed* or program* or ward* or setting* or facilit* or service* or model* or centre* or center*)):ti,ab,kw
2. [mh ^"Transitional care"]
3. (Intermediate NEAR/3 (unit* or care or bed* or program* or ward* or setting* or facilit* or service* or model* or centre* or center*)):ti,ab,kw
4. [mh ^"Intermediate care facilities"]
5. ((Subacute or sub-acute) NEAR/3 (unit* or care or bed* or program* or ward* or setting* or facilit* or service* or model* or centre* or center*)):ti,ab,kw
6. [mh ^"Subacute care"]
7. ((Postacute or post-acute) NEAR/3 (unit* or care or bed* or program* or ward* or setting* or facilit* or service* or model* or centre* or center*)):ti,ab,kw
8. ((Post acute) NEAR/3 (unit* or care or bed* or program* or ward* or setting* or facilit* or service* or model* or centre* or center*)):ti,ab,kw
9. ((Skilled Nursing) NEAR/3 (unit* or bed* or program* or ward* or setting* or facilit* or service* or model* or centre* or center*)):ti,ab,kw
10. [mh ^"Skilled nursing facilities"]
11. (Restor* NEAR/3 (unit* or care or bed* or program* or ward* or setting* or facilit* or service* or model* or centre* or center*)):ti,ab,kw
12. (Convalesc* NEAR/3 (unit* or care or bed* or program* or ward* or setting* or facilit* or service* or model* or centre* or center* or home* or hospital*)):ti,ab,kw
13. [mh ^"Convalescence"]
14. <u>{or #1-#13}</u>
15. (Old* or aged or aging):ti,ab,kw
16. (Centenarian* or nonagenarian* or octogenarian* or geriatr* or gerontol* or senescen* or septuagenarian* or pensioner* or senile):ti,ab,kw
17. Senior*:ti,ab,kw
18. Elder*:ti,ab,kw
19. [mh ^"Aged"]
20. [mh ^"Aged, 80 and over"]
21. <u>{or #15-#20}</u>
22. (Cognit* NEAR/3impair*):ti,ab,kw
23. (Mild neurocognitive disorder*):ti,ab,kw
24. [mh ^"Cognitive dysfunction"]
25. (Major neurocognitive disorder*):ti,ab,kw
26. Dement*:ti,ab,kw

27. [mh ^"Dementia"]
28. [mh ^"Alzheimer disease"]
29. Alzheimer*:ti,ab,kw
30. [mh ^"AIDS Dementia Complex"]
31. [mh ^"Dementia, Vascular"]
32. [mh ^"Dementia, Multi-Infarct"]
33. [mh ^"Lewy body disease"]
34. Deliri*:ti,ab,kw
35. [mh ^"Delirium"]
36. {or #22-#35}
37. #14 and #21 and #36
    Result: 1193 articles

▼ **Search History** (37) | View Saved

# ▲	Searches	Results	Type	Actions	Annotations
1	(Transition* adj3 (care* or unit* or bed* or program* or ward* or setting* or facilit* or service* or model* or centre* or center*)).tw,kf.	21273	Advanced	Display Results | More	
2	Transitional care/	946	Advanced	Display Results | More	
3	(Intermediate adj3 (unit* or care or bed* or program* or ward* or setting* or facilit* or service* or model* or centre* or center*)).tw,kf.	4941	Advanced	Display Results | More	
4	Intermediate care facilities/	709	Advanced	Display Results | More	
5	((Subacute or sub-acute) adj3 (unit* or care or bed* or program* or ward* or setting* or facilit* or service* or model* or centre* or center*)).tw,kf.	1607	Advanced	Display Results | More	
6	Subacute care/	1226	Advanced	Display Results | More	
7	((Postacute or post-acute) adj3 (unit* or care or bed* or program* or ward* or setting* or facilit* or service* or model* or centre* or center*)).tw,kf.	2470	Advanced	Display Results | More	
8	(Post acute adj3 (unit* or care or bed* or program* or ward* or setting* or facilit* or service* or model* or centre* or center*)).tw,kf.	1590	Advanced	Display Results | More	
9	(Skilled Nursing adj3 (unit* or bed* or program* or ward* or setting* or facilit* or service* or model* or centre* or center*)).tw,kf.	3298	Advanced	Display Results | More	
10	Skilled nursing facilities/	4684	Advanced	Display Results | More	
11	(Restor* adj3 (unit* or care or bed* or program* or ward* or setting* or facilit* or service* or model* or centre* or center*)).tw,kf.	4853	Advanced	Display Results | More	
12	(Convalesc* adj3 (unit* or care or bed* or program* or ward* or setting* or facilit* or service* or model* or centre* or center* or home* or hospital*)).tw,kf.	1170	Advanced	Display Results | More	
13	Convalescence/	3769	Advanced	Display Results | More	
14	or/1-13	45862	Advanced	Display Results | More	
15	(Old* or aged or aging).tw,kf.	2146884	Advanced	Display Results | More	
16	(Centenarian* or nonagenarian* or octogenarian* or geriatr* or gerontol* or senescen* or septuagenarian* or pensioner* or senile).tw,kf.	143696	Advanced	Display Results | More	
17	Senior*.tw,kf.	44525	Advanced	Display Results | More	
18	Elder*.tw,kf.	277924	Advanced	Display Results | More	
19	Aged/	3227978	Advanced	Display Results | More	
20	"Aged, 80 and over"/	968934	Advanced	Display Results | More	
21	or/15-20	4979344	Advanced	Display Results | More	
22	(Cognit* adj3 impair*).tw,kf.	87569	Advanced	Display Results | More	
23	Mild neurocognitive disorder*.tw,kf.	196	Advanced	Display Results | More	
24	Cognitive dysfunction/	23446	Advanced	Display Results | More	

Contract

	#	Searches	Results	Type	Actions	
☐	25	Major neurocognitive disorder*.tw,kf.	195	Advanced	Display Results | More	
☐	26	Dement*.tw,kf.	126201	Advanced	Display Results | More	
☐	27	Dementia/	54314	Advanced	Display Results | More	
☐	28	Alzheimer disease/	100955	Advanced	Display Results | More	
☐	29	Alzheimer*.tw,kf.	161199	Advanced	Display Results | More	
☐	30	AIDS Dementia Complex/	3841	Advanced	Display Results | More	
☐	31	Dementia, Vascular/	5152	Advanced	Display Results | More	
☐	32	Dementia, Multi-Infarct/	1089	Advanced	Display Results | More	
☐	33	Lewy body disease/	3599	Advanced	Display Results | More	
☐	34	Deliri*.tw,kf.	17817	Advanced	Display Results | More	
☐	35	Delirium/	10213	Advanced	Display Results | More	
☐	36	or/22-35	331515	Advanced	Display Results | More	
☐	37	14 and 21 and 36	1102	Advanced	Display Results | More	

Save | Remove | Combine with: AND | OR

Save All | Edit | Create RSS | View Saved | Email All Search History | Copy Search History Link | Copy Search History Details

Advanced Search | Basic Search | Find Citation | Search Tools | Search Fields | Multi-Field Search

1 Resource selected | Hide | Change

Ovid MEDLINE: Epub Ahead of Print, In-Process & Other Non-Indexed Citations, Ovid MEDLINE® Daily and Ovid MEDLINE® 1946-Present

Enter keyword or phrase (* or $ for truncation)

◉ **Keyword** ○ Author ○ Title ○ Journal

Search | Expand Term Finder

Limits *(expand)* ☐ Include Multimedia ☑ Map Term to Subject Heading

Options

To search Open Access content on Ovid, go to Basic Search.

Print | Email | Export | + My Projects | Keep Selected

☐ All | Range | Clear | 100 Per Page | 1 | Go | Next ›

Search Information

You searched:

14 and 21 and 36

Search terms used:

80
aged
aged,
and
over
aging
aids
dementia
complex
alzheimer
disease
alzheimer*
bed*
care
care*
centenarian*
center*
centre*
cognit*
cognitive

☐ 1.

Abstract Reference
Complete Reference

Find Similar
Find Citing Articles

Get it UTL

**Unique Identifier**	32816014
**Title**	**Stress, Burden, and Well-Being in Dementia and Nondementia Caregivers: Insights From the Caregiving Transitions Study.**
**Title Comment**	Erratum in: Gerontologist. 2021 Jul 13;61(5):804; PMID: 33415329 [https://www-ncbi-nlm-nih-gov.myaccess.library.utoronto.ca/pub...]
**Source**	Gerontologist. 61(5):670-679, 2021 Jul 13.
**Authors**	Sheehan OC; Haley WE; Howard VJ; Huang J; Rhodes JD; Roth DL
**Authors Full Name**	Sheehan, Orla C; Haley, William E; Howard, Virginia J; Huang, Jin; Rhodes, J David; Roth, David L.
**Publication Type**	Journal Article.

Cite | + My Projects | + Annotate

dysfunction
convalesc*
convalescence
deliri*
delirium
dement*
dementia,
multi-infarct
vascular
elder*
facilit*
geriatr*
gerontol*
home*
hospital*
impair*
intermediate
facilities
lewy
body
major
neurocognitive
disorder*
mild
model*
nonagenarian*
octogenarian*
old*
pensioner*
post
acute
post-acute
postacute
program*
restor*
senescen*
senile
senior*
septuagenarian*
service*
setting*
skilled
nursing
sub-acute
subacute
transition*
transitional
unit*
ward*

Search Returned:
1102 text results

Sort By:

-

Customize Display

Filter By

Add to Search History

Selected Only (0)

Years

All Years
Current year
Past 3 years
Past 5 years

2.

**Unique Identifier**	32776123
**Title**	**Participation in a Substance Misuse Intervention in Post-acute Care Is Associated With More Optimal Rehabilitation Outcomes.**
**Source**	Gerontologist. 61(5):787-796, 2021 Jul 13.
**Authors**	Cimarolli VR; Burack O; Minahan J; Hennessa A; Stone R; Shi X
**Authors Full Name**	Cimarolli, Verena R; Burack, Orah; Minahan, Jillian; Hennessa, Alexandra; Stone, Robyn; Shi, Xiaomei.
**Publication Type**	Journal Article.

Abstract Reference
Complete Reference
Find Similar
Find Citing Articles
Get it UTL

Cite + My Projects + Annotate

3.

**Unique Identifier**	34250881
**Title**	**A Scoping Review of Care Trajectories across Multiple Settings for Persons with Dementia.**
**Source**	Canadian Journal on Aging. 1-25, 2021 Jul 12.
**Authors**	Kosteniuk JG; Morgan DG; Elliot V; Froehlich Chow A; Bayly M; Watson E; Osman M; Acan Osman B; O'Connell ME; Kirk A; Stewart N; Cammer A; Innes A
**Authors Full Name**	Kosteniuk, Julie G; Morgan, Debra G; Elliot, Valerie; Froehlich Chow, Amanda; Bayly, Melanie; Watson, Erin; Osman, Meric; Acan Osman, Beliz; O'Connell, Megan E; Kirk, Andrew; Stewart, Norma; Cammer, Allison; Innes, Anthea.
**Publication Type**	Journal Article.

Abstract Reference
Complete Reference
Find Similar
Find Citing Articles
Get it UTL

Cite + My Projects + Annotate

4.

**Unique Identifier**	34237256
**Title**	**Association Between Cognitive Status and Falls With and Without Injury During a Skilled Nursing Facility Short Stay.**
**Source**	Journal of the American Medical Directors Association. 2021 Jul 05.
**Authors**	Tzeng HM; Downer B; Haas A; Ottenbacher KJ
**Authors Full Name**	Tzeng, Huey-Ming; Downer, Brian; Haas, Allen; Ottenbacher, Kenneth J.
**Publication Type**	Journal Article.

Abstract Reference
Complete Reference
Find Similar
Find Citing Articles
Get it UTL

Cite + My Projects + Annotate

5.

**Unique Identifier**	34212470
**Title**	**Toward the development of a vibrant, super-aged society: The**

Abstract Reference
Complete Reference
Find Similar

- ▶ Specific Year Range
- ▶ Subject
- ▶ Author
- ▶ Journal
- ▶ Publication Type

▼ My Projects

+ New Project

No projects available.

future of medicine and society in Japan.

Find Citing Articles

Full Text

Source Geriatrics & gerontology international. 2021 Jul 01.

Authors Iijima K; Arai H; Akishita M; Endo T; Ogasawara K; Kashihara N; Hayashi YK; Yumura W; Yokode M; Ouchi Y

Authors Full Name Iijima, Katsuya; Arai, Hidenori; Akishita, Masahiro; Endo, Tamao; Ogasawara, Kouetsu; Kashihara, Naoki; Hayashi, Yukiko K; Yumura, Wako; Yokode, Masayuki; Ouchi, Yasuyoshi.

Publication Type Journal Article.

Cite + My Projects + Annotate

6.

Abstract Reference
Complete Reference

Unique Identifier 33543243

Title Age and frailty are independently associated with increased COVID-19 mortality and increased care needs in survivors: results of an international multi-centre study.

Find Similar
Find Citing Articles

Source Age & Ageing. 2021 Feb 05.

Authors Geriatric Medicine Research Collaborative

Authors Full Name Geriatric Medicine Research Collaborative.

Publication Type Journal Article.

Cite + My Projects + Annotate

7.

Abstract Reference
Complete Reference

Unique Identifier 33481990

Title Follow-up services for delirium after COVID-19-where now?.

Find Similar
Find Citing Articles

Get it UTL

Source Age & Ageing. 2021 Jan 22.

Authors Rahman S; Byatt K

Authors Full Name Rahman, Shibley; Byatt, Kit.

Publication Type Journal Article.

Cite + My Projects + Annotate

8.

Abstract Reference
Complete Reference

Unique Identifier 34137032

Title Trajectories of functional performance recovery after inpatient geriatric rehabilitation: an observational study.

Find Similar
Find Citing Articles

Get it UTL

Source Medical Journal of Australia. 2021 Jun 16.

Authors Soh CH; Reijnierse EM; Tuttle C; Marston C; Goonan R; Lim WK; Maier AB

Authors Full Name Soh, Cheng Hwee; Reijnierse, Esmee M; Tuttle, Camilla; Marston, Celia; Goonan, Rose; Lim, Wen Kwang; Maier, Andrea B.

Publication Type | Journal Article.

Cite | + My Projects | + Annotate

9.

Unique Identifier 34108416

Title High Occurrence of Post Intensive Care Syndrome Identified in Surgical ICU Survivors After Implementation of a Multidisciplinary Clinic.

Source The Journal of Trauma and Acute Care Surgery. 2021 Apr 08.

Authors Bottom-Tanzer SF; Poyant JO; Louzada MT; Ahmed SE; Boudouvas A; Poon E; Hojman HM; Bugaev N; Johnson BP; Van Kirk A; Daniel E; Emoff C; Mahoney EJ

Authors Full Name Bottom-Tanzer, Samantha F; Poyant, Janelle O; Louzada, Maria T; Ahmed, Sana E; Boudouvas, Abbey; Poon, Eileen; Hojman, Horacio M; Bugaev, Nikolay; Johnson, Benjamin P; Van Kirk, Annalisa; Daniel, Eryn; Emoff, Caroline; Mahoney, Eric J.

Publication Type Journal Article.

Ovid Full Text
Abstract Reference
Complete Reference

Find Similar
Find Citing Articles

Article as PDF (1273KB) | Cite | + My Projects | + Annotate

10.

Unique Identifier 34109693

Title Analysis of discharge documentation for older adults living with dementia: A cohort study.

Source Journal of Clinical Nursing. 2021 Jun 09.

Authors Parker KJ; Phillips JL; Luckett T; Agar M; Ferguson C; Hickman LD

Authors Full Name Parker, Kirsten J; Phillips, Jane L; Luckett, Tim; Agar, Meera; Ferguson, Caleb; Hickman, Louise D.

Publication Type Journal Article.

Abstract Reference
Complete Reference

Find Similar
Find Citing Articles

Full Text

Cite | + My Projects | + Annotate

11.

Unique Identifier 34059366

Title Effect of Dementia on Outcomes After Surgically Treated Hip Fracture in Older Adults.

Source Journal of Arthroplasty. 2021 Apr 30.

Authors Jorissen RN; Inacio MC; Cations M; Lang C; Caughey GE; Crotty M

Authors Full Name Jorissen, Robert N; Inacio, Maria C; Cations, Monica; Lang, Catherine; Caughey, Gillian E; Crotty, Maria.

Publication Type Journal Article.

Abstract Reference
Complete Reference

Find Similar
Find Citing Articles

Get it UTL

Cite + My Projects + Annotate

12.

Abstract Reference
Complete Reference

Unique Identifier 34047895

Title Ageing- and dementia-friendly design: theory and evidence from cognitive psychology, neuropsychology and environmental psychology can contribute to design guidelines that minimise spatial disorientation.

Find Similar
Find Citing Articles

Get it UTL

Source Cognitive Processing. 2021 May 28.

Authors Wiener JM; Pazzaglia F

Authors Full Name Wiener, Jan M; Pazzaglia, Francesca.

Publication Type Journal Article.

Cite + My Projects + Annotate

13.

Abstract Reference
Complete Reference

Unique Identifier 33984248

Title Functional Outcomes, Goals, and Goal Attainment Amongst Chronically Critically Ill Long-Term Acute Care Hospital Patients.

Find Similar
Find Citing Articles

Get it UTL

Source Annals of the American Thoracic Society. 2021 May 13.

Authors Dubin R; Veith JM; Grippi MA; McPeake J; Harhay MO; Mikkelsen ME

Authors Full Name Dubin, Randy; Veith, Joshua M; Grippi, Michael A; McPeake, Joanne; Harhay, Michael O; Mikkelsen, Mark E.

Publication Type Journal Article.

Cite + My Projects + Annotate

14.

Abstract Reference
Complete Reference

Unique Identifier 33476569

Title Mobility and Self-Care are Associated With Discharge to Community After Home Health for People With Dementia.

Find Similar
Find Citing Articles

Get it UTL

Source Journal of the American Medical Directors Association. 2021 Jan 19.

Authors Knox S; Downer B; Haas A; Ottenbacher KJ

Authors Full Name Knox, Sara; Downer, Brian; Haas, Allen; Ottenbacher, Kenneth J.

Publication Type Journal Article.

Cite + My Projects + Annotate

15.

Abstract Reference
Complete Reference

Unique Identifier 32730727

**Title**	**Hospitalization and discharge routes of elderly hip fracture patients with and without dementia: a nationwide cross-sectional exploratory study using the Japanese Diagnostic Procedure Combination database.**	Find Similar<br>Find Citing Articles<br><br>Full Text
**Source**	Disability & Rehabilitation. 1-7, 2020 Jul 30.	
**Authors**	Mine Y; Muramatsu K; Fushimi K; Matsuda S	
**Authors Full Name**	Mine, Yuko; Muramatsu, Keiji; Fushimi, Kiyohide; Matsuda, Shinya.	
**Publication Type**	Journal Article.	

Cite + My Projects + Annotate

16.

**Unique Identifier**	33783631	Abstract Reference<br>Complete Reference
**Title**	**Appropriateness of the post-operative rehabilitation of low energy hip fractures in elderly in comparison with the AAOS appropriate use criteria at a level one trauma center.**	Find Similar<br>Find Citing Articles
**Source**	European journal of orthopaedic surgery & traumatologie. 2021 Mar 30.	Get it UTL
**Authors**	Adam M; Alkaramany E; Alhamoud A; Derbas J; Murshid A; Alhaneedi GA	
**Authors Full Name**	Adam, Mohammed; Alkaramany, Eslam; Alhamoud, Abduljabbar; Derbas, Jawad; Murshid, Abdullah; Alhaneedi, Ghalib Ahmed.	
**Publication Type**	Journal Article.	

Cite + My Projects + Annotate

17.

**Unique Identifier**	33271123	Abstract Reference<br>Complete Reference
**Title**	**Cross-Cultural Adaptation and Validation of the Italian Version of the Observational Scale of Level of Arousal.**	Find Similar<br>Find Citing Articles
**Source**	Journal of the American Medical Directors Association. 2020 Nov 30.	Get it UTL
**Authors**	Martella LA; Carmisciano L; Giannotti C; Signori A; Pontremoli R; Giusti M; Gualco E; Beccati V; Marengoni A; Nencioni A; Monacelli F	
**Authors Full Name**	Martella, Lucia Anna; Carmisciano, Luca; Giannotti, Chiara; Signori, Alessio; Pontremoli, Roberto; Giusti, Massimo; Gualco, Elisa; Beccati, Valentina; Marengoni, Alessandra; Nencioni, Alessio; Monacelli, Fiammetta.	
**Publication Type**	Journal Article.	

Cite + My Projects + Annotate

18.

**Unique Identifier**	33320184	Abstract Reference<br>Complete Reference
**Title**	**Individual heterogeneity in the probability of hospitalization, skilled nursing facility admission and mortality.**	Find Similar<br>Find Citing Articles

**Source**	Journals of Gerontology Series A-Biological Sciences & Medical Sciences. 2020 Dec 15.	Get it UTL
**Authors**	McAvay GJ; Vander Wyk B; Allore H	
**Authors Full Name**	McAvay, Gail J; Vander Wyk, Brent; Allore, Heather.	
**Publication Type**	Journal Article.	

Cite + My Projects + Annotate

☐ 19.

**Unique Identifier**	33283698	Abstract Reference<br>Complete Reference
**Title**	**The prevalence rates and sequelae of delirium at age older than 90 years.**	Find Similar<br>Find Citing Articles
**Source**	Palliative & Supportive Care. 1-6, 2020 Dec 07.	Get it UTL
**Authors**	Gehrke S; Bode L; Seiler A; Ernst J; von Kanel R; Boettger S	
**Authors Full Name**	Gehrke, Samuel; Bode, Leonie; Seiler, Annina; Ernst, Jutta; von Kanel, Roland; Boettger, Soenke.	
**Publication Type**	Journal Article.	

Cite + My Projects + Annotate

☐ 20.

**Unique Identifier**	33246842	Abstract Reference<br>Complete Reference
**Title**	**Health Profiles, Health Services Use, and Transition to Dementia in Inpatients With Late-Life Depression and Other Mental Illnesses.**	Find Similar<br>Find Citing Articles
**Source**	Journal of the American Medical Directors Association. 2020 Nov 15.	Get it UTL
**Authors**	Reppermund S; Heintze T; Srasuebkul P; Trollor JN	
**Authors Full Name**	Reppermund, Simone; Heintze, Theresa; Srasuebkul, Preeyaporn; Trollor, Julian N.	
**Publication Type**	Journal Article.	

Cite + My Projects + Annotate

☐ 21.

**Unique Identifier**	33071094	Abstract Reference<br>Complete Reference
**Title**	**Predictors of return visits to the emergency department among different age groups of older adults.**	Find Similar<br>Find Citing Articles
**Source**	American Journal of Emergency Medicine. 2020 Jul 22.	Full Text
**Authors**	Oliveira J E Silva L; Jeffery MM; Campbell RL; Mullan AF; Takahashi PY; Bellolio F	
**Authors Full Name**	Oliveira J E Silva, Lucas; Jeffery, Molly M; Campbell, Ronna L; Mullan, Aidan F; Takahashi, Paul Y; Bellolio, Fernanda.	

**Publication Type**	Journal Article.

Cite + My Projects + Annotate

22.

Abstract Reference
Complete Reference

**Unique Identifier**	33021670
**Title**	**Increasing Life-Space Mobility in community-dwelling older persons with cognitive impairment following rehabilitation: A randomized controlled trial.**
**Source**	Journals of Gerontology Series A-Biological Sciences & Medical Sciences. 2020 Oct 06.
**Authors**	Ullrich P; Werner C; Bongartz M; Eckert T; Abel B; Schonstein A; Kiss R; Hauer K
**Authors Full Name**	Ullrich, Phoebe; Werner, Christian; Bongartz, Martin; Eckert, Tobias; Abel, Bastian; Schonstein, Anton; Kiss, Rainer; Hauer, Klaus.
**Publication Type**	Journal Article.

Find Similar
Find Citing Articles
Get it UTL

Cite + My Projects + Annotate

23.

Abstract Reference
Complete Reference

**Unique Identifier**	32839751
**Title**	**Labile PT-INR in a Covid-19 Patient Under Long-term Vitamin K Antagonist Therapy: a Case Report.**
**Source**	SN Comprehensive Clinical Medicine. 1-3, 2020 Aug 19.
**Authors**	Trevisan C; Miconi L; Barbierato E; Marinaro G; Targhetta S; D'agata M; Rinaldi D
**Authors Full Name**	Trevisan, Caterina; Miconi, Lorella; Barbierato, Emanuele; Marinaro, Giuseppe; Targhetta, Stefano; D'agata, Mario; Rinaldi, Daniela.
**Publication Type**	Journal Article.

Find Similar
Find Citing Articles
Get it UTL

Cite + My Projects + Annotate

24.

Abstract Reference
Complete Reference

**Unique Identifier**	32431132
**Title**	**Changing needs in advanced dementia.**
**Source**	Nursing Older People. 2020 May 20.
**Authors**	Brown M; Tolson D; Ritchie L
**Authors Full Name**	Brown, Margaret; Tolson, Debbie; Ritchie, Louise.
**Publication Type**	Journal Article.

Find Similar
Find Citing Articles
Get it UTL

Cite + My Projects + Annotate

25.

Abstract Reference
Complete Reference

**Unique Identifier**	31697390
**Title**	**Disparities in Dementia Occurrence and Rate of Cognitive Decline Between African American and White Long-Term Care Residents.**
**Source**	Research in Gerontological Nursing. 1-7, 2019 Nov 06.
**Authors**	Mace RA; Mansbach WE
**Authors Full Name**	Mace, Ryan A; Mansbach, William E.
**Publication Type**	Journal Article.

Find Similar
Find Citing Articles

Get it UTL

Cite + My Projects + Annotate

26.

Abstract Reference
Complete Reference

**Unique Identifier**	29751966
**Title**	**Outcomes in nursing home patients with traumatic brain injury.**
**Source**	Surgery. 2018 May 09.
**Authors**	Lueckel SN; Kosar CM; Teno JM; Monaghan SF; Heffernan DS; Cioffi WG; Thomas KS
**Authors Full Name**	Lueckel, Stephanie N; Kosar, Cyrus M; Teno, Joan M; Monaghan, Sean F; Heffernan, Daithi S; Cioffi, William G; Thomas, Kali S.
**Publication Type**	Journal Article.

Find Similar
Find Citing Articles

Get it UTL

Cite + My Projects + Annotate

27.

Abstract Reference
Complete Reference

**Unique Identifier**	30942985
**Book Title**	Emergency Department Interventions for Older Adults
**Source**	Department of Veterans Affairs (US). VA Evidence-based Synthesis Program Reports 2018 06.
**Authors**	Hughes JM; Freiermuth CE; Williams JW Jr.; Ragsdale L; Eucker S; Goldstein K; Rodriguez R; Fulton J; Hastings SN; Shepherd-Banigan M; Ramos K; Tabriz AA; Gordon AM; Gierisch JM; Kosinski A; McDuffie J; Van Noord M
**Authors Full Name**	Hughes, Jaime M.; Freiermuth, Caroline E.; Williams, John W. Jr.; Ragsdale, Luna; Eucker, Stephanie; Goldstein, Karen; Rodriguez, Rachel; Fulton, Jessica; Hastings, S. Nicole; Shepherd-Banigan, Megan; Ramos, Katherine; Tabriz, Amir Alishahi; Gordon, Adelaide M.; Gierisch, Jennifer M.; Kosinski, Andrzej; McDuffie, Jennifer; Van Noord, Megan.
**Publication Type**	Review.

Full Text

Cite + My Projects + Annotate

28.

Abstract Reference
Complete Reference

Unique Identifier 26207079

Title **Planning and Decision Making for Care Transitions.**

Source Annual Review of Gerontology & Geriatrics. 31(1):111-142, 2011.

Authors Sorensen S; Mak W; Pinquart M

Authors Full Name Sorensen, Silvia; Mak, Wingyun; Pinquart, Martin.

Publication Type Journal Article.

Find Similar
Find Citing Articles

Get it UTL

Cite + My Projects + Annotate

29.

Abstract Reference
Complete Reference

Unique Identifier 34245586

Title **Participation of persons with dementia and their caregivers in research. [Review]**

Source Journal of the American Geriatrics Society. 69(7):1784-1792, 2021 Jul.

Authors Frank L; Jennings LA; Petersen RC; Majid T; Gilmore-Bykovskyi A; Schicker L; Karlawish J

Authors Full Name Frank, Lori; Jennings, Lee A; Petersen, Ronald C; Majid, Tabassum; Gilmore-Bykovskyi, Andrea; Schicker, Lonni; Karlawish, Jason.

Publication Type Journal Article. Review.

Find Similar
Find Citing Articles

Full Text

Cite + My Projects + Annotate

30.

Abstract Reference
Complete Reference

Unique Identifier 34081627

Title ***Cistanche deserticola* polysaccharides alleviate cognitive decline in aging model mice by restoring the gut microbiota-brain axis.**

Source Aging. 13(11):15320-15335, 2021 06 03.

Authors Gao Y; Li B; Liu H; Tian Y; Gu C; Du X; Bu R; Gao J; Liu Y; Li G

Authors Full Name Gao, Yuan; Li, Bing; Liu, Hong; Tian, Yajuan; Gu, Chao; Du, Xiaoli; Bu, Ren; Gao, Jie; Liu, Yang; Li, Gang.

Publication Type Journal Article. Research Support, Non-U.S. Gov't.

Find Similar
Find Citing Articles

Full Text

Cite + My Projects + Annotate

31.

Ovid Full Text
Abstract Reference
Complete Reference

Unique Identifier 34131093

Title **Executive Function Moderates Functional Outcomes of Engagement Strategies During Rehabilitation in Older Adults.**

Source American Journal of Physical Medicine & Rehabilitation. 100(7):635-

Find Similar
Find Citing Articles

642, 2021 Jul 01.

Authors Ercal B; Rodebaugh TL; Bland MD; Barco P; Lenard E; Lang CE; Miller JP; Yingling M; Lenze EJ

Authors Full Name Ercal, Baris; Rodebaugh, Thomas L; Bland, Marghuretta D; Barco, Peggy; Lenard, Emily; Lang, Catherine E; Miller, J Philip; Yingling, Michael; Lenze, Eric J.

Publication Type Journal Article.

Article as PDF (1801KB) Cite + My Projects + Annotate

32.

Abstract Reference
Complete Reference

Unique Identifier 33290190

Title **Skeletal Muscle Index's Impact on Discharge Disposition After Head and Neck Cancer Free Flap Reconstruction.**

Find Similar
Find Citing Articles

Source Otolaryngology - Head & Neck Surgery. 165(1):59-68, 2021 Jul.

Get it UTL

Authors Jones AJ; Campiti VJ; Alwani M; Novinger LJ; Bonetto A; Sim MW; Yesensky JA; Moore MG; Mantravadi AV

Authors Full Name Jones, Alexander Joseph; Campiti, Vincent Joseph; Alwani, Mohamedkazim; Novinger, Leah J; Bonetto, Andrea; Sim, Michael W; Yesensky, Jessica A; Moore, Michael G; Mantravadi, Avinash V.

Publication Type Journal Article.

Cite + My Projects + Annotate

33.

Abstract Reference
Complete Reference

Unique Identifier 32558919

Title **In-hospital interventions for reducing readmissions to acute care for adults aged 65 and over: An umbrella review.**

Find Similar
Find Citing Articles

Source International Journal for Quality in Health Care. 32(7):414-430, 2020 Sep 23.

Full Text

Authors Conroy T; Heuzenroeder L; Feo R

Authors Full Name Conroy, Tiffany; Heuzenroeder, Louise; Feo, Rebecca.

Publication Type Journal Article.

Cite + My Projects + Annotate

34.

Abstract Reference
Complete Reference

Unique Identifier 33270824

Title **The impact of dementia on aged care service transitions in the last five years of life.**

Find Similar
Find Citing Articles

Source Age & Ageing. 50(4):1159-1165, 2021 Jun 28.

Get it UTL

Authors Welberry HJ; Jorm LR; Barbieri S; Hsu B; Brodaty H

Authors Full Welberry, Heidi J; Jorm, Louisa R; Barbieri, Sebastiano; Hsu,

**Name**	Benjumin; Brodaty, Henry.
**Publication Type**	Journal Article.

Cite + My Projects + Annotate

35.

Abstract Reference
Complete Reference

**Unique Identifier**	33814428
**Title**	The Characteristics of Social Network Structure in Later Life in Relation to Incidence of Mild Cognitive Impairment and Conversion to Probable Dementia.
**Source**	Journal of Alzheimer's Disease. 81(2):699-710, 2021.
**Authors**	Zhang Y; Natale G; Clouston S
**Authors Full Name**	Zhang, Yun; Natale, Ginny; Clouston, Sean.
**Publication Type**	Journal Article.

Find Similar
Find Citing Articles
Get it UTL

Cite + My Projects + Annotate

36.

Ovid Full Text
Abstract Reference
Complete Reference

**Unique Identifier**	33196587
**Title**	What Factors Predict Adverse Discharge Disposition in Patients Older Than 60 Years Undergoing Lower-extremity Surgery? The Adverse Discharge in Older Patients after Lower-extremity Surgery (ADELES) Risk Score.
**Title Comment**	Comment in: Clin Orthop Relat Res. 2021 Mar 1;479(3):558-560; PMID: 33201023 [https://www-ncbi-nlm-nih-gov.myaccess.library.utoronto.ca/pub...]
**Source**	Clinical Orthopaedics & Related Research. 479(3):546-547, 2021 03 01.
**Authors**	Schaefer MS; Hammer M; Platzbecker K; Santer P; Grabitz SD; Murugappan KR; Houle T; Barnett S; Rodriguez EK; Eikermann M
**Authors Full Name**	Schaefer, Maximilian S; Hammer, Maximilian; Platzbecker, Katharina; Santer, Peter; Grabitz, Stephanie D; Murugappan, Kadhiresan R; Houle, Tim; Barnett, Sheila; Rodriguez, Edward K; Eikermann, Matthias.
**Publication Type**	Journal Article.

Find Similar
Find Citing Articles

Cite + My Projects + Annotate

37.

Ovid Full Text
Abstract Reference
Complete Reference

**Unique Identifier**	33528173
**Title**	Successful Community Discharge Among Older Adults With Traumatic Brain Injury in Skilled Nursing Facilities.
**Source**	Journal of Head Trauma Rehabilitation. 36(3):E186-E198, 2021 May-Jun 01.

Find Similar
Find Citing Articles

**Authors**	Evans E; Gutman R; Resnik L; Zonfrillo MR; Lueckel SN; Kumar RG; DeVone F; Dams-O'Connor K; Thomas KS
**Authors Full Name**	Evans, Emily; Gutman, Roee; Resnik, Linda; Zonfrillo, Mark R; Lueckel, Stephanie N; Kumar, Raj G; DeVone, Frank; Dams-O'Connor, Kristen; Thomas, Kali S.
**Publication Type**	Journal Article.

Article as PDF (473KB) Cite + My Projects + Annotate

38.

Abstract Reference
Complete Reference

Find Similar
Find Citing Articles

Get it UTL

**Unique Identifier**	32820702
**Title**	**Interventions to optimise transitional care coordination for older people living with dementia and concomitant multimorbidity and their caregivers: A systematic review.**
**Source**	Contemporary Nurse. 56(5-6):505-533, 2020 Oct-Dec.
**Authors**	Parker KJ; Hickman LD; Phillips JL; Ferguson C
**Authors Full Name**	Parker, Kirsten J; Hickman, Louise D; Phillips, Jane L; Ferguson, Caleb.
**Publication Type**	Journal Article.

Cite + My Projects + Annotate

39.

Abstract Reference
Complete Reference

Find Similar
Find Citing Articles

Get it UTL

**Unique Identifier**	33813146
**Title**	**Validation of the Delirium Diagnostic Tool-Provisional (DDT-Pro) in a skilled nursing facility and comparison to the 4 'A's test (4AT).**
**Source**	General Hospital Psychiatry. 70:116-123, 2021 May-Jun.
**Authors**	Sepulveda E; Bermudez E; Gonzalez D; Cotino P; Vinuelas E; Palma J; Ciutat M; Grau I; Vilella E; Trzepacz PT; Franco JG
**Authors Full Name**	Sepulveda, Esteban; Bermudez, Ester; Gonzalez, Dulce; Cotino, Paula; Vinuelas, Eva; Palma, Jose; Ciutat, Marta; Grau, Imma; Vilella, Elisabet; Trzepacz, Paula T; Franco, Jose G.
**Publication Type**	Journal Article.

Cite + My Projects + Annotate

40.

Abstract Reference
Complete Reference

Find Similar
Find Citing Articles

Full Text

**Unique Identifier**	32911278
**Title**	**Delirium in elderly patients: Prospective prevalence across hospital services.**
**Source**	General Hospital Psychiatry. 67:19-25, 2020 Nov - Dec.
**Authors**	Fuchs S; Bode L; Ernst J; Marquetand J; von Kanel R; Bottger S
**Authors Full Name**	Fuchs, Simon; Bode, Leonie; Ernst, Jutta; Marquetand, Justus; von Kanel, Roland; Bottger, Sonke.

Publication Type Journal Article.

Cite + My Projects + Annotate

41.

Abstract Reference
Complete Reference

Unique Identifier 33300605

Title **Successful Discharge to Community Gap of FFS Medicare Beneficiaries With and Without ADRD Narrowed.**

Source Journal of the American Geriatrics Society. 69(4):972-978, 2021 Apr.

Authors Bardenheier BH; Rahman M; Kosar C; Werner RM; Mor V

Authors Full Name Bardenheier, Barbara H; Rahman, Momotazur; Kosar, Cyrus; Werner, Rachel M; Mor, Vincent.

Publication Type Journal Article.

Find Similar
Find Citing Articles

Full Text

Cite + My Projects + Annotate

42.

Abstract Reference
Complete Reference

Unique Identifier 33582120

Title **Cerebrovascular damage after midlife transient hypertension in non-transgenic and Alzheimer's disease rats.**

Source Brain Research. 1758:147369, 2021 May 01.

Authors Lai AY; Joo IL; Trivedi AU; Dorr A; Hill ME; Stefanovic B; McLaurin J

Authors Full Name Lai, Aaron Y; Joo, Illsung L; Trivedi, Arunachala U; Dorr, Adrienne; Hill, Mary E; Stefanovic, Bojana; McLaurin, JoAnne.

Publication Type Journal Article.

Find Similar
Find Citing Articles

Full Text

Cite + My Projects + Annotate

43.

Abstract Reference
Complete Reference

Unique Identifier 33769114

Title **Challenges to Admitting Residents: Perspectives from Rural Nursing Home Administrators and Staff.**

Source Inquiry. 58:469580211005191, 2021 Jan-Dec.

Authors Henning-Smith C; Cross D; Rahman A

Authors Full Name Henning-Smith, Carrie; Cross, Dori; Rahman, Adrita.

Publication Type Journal Article.

Find Similar
Find Citing Articles

Get it UTL

Cite + My Projects + Annotate

44.

Abstract Reference

Complete Reference

Unique Identifier 30841745

Title **Caregivers of older adults with dementia and multiple chronic conditions: Exploring their experiences with significant changes.**

Find Similar
Find Citing Articles

Get it UTL

Source Dementia. 19(8):2601-2620, 2020 Nov.

Authors Ploeg J; Northwood M; Duggleby W; McAiney CA; Chambers T; Peacock S; Fisher K; Ghosh S; Markle-Reid M; Swindle J; Williams A; Triscott JA

Authors Full Name Ploeg, Jenny; Northwood, Melissa; Duggleby, Wendy; McAiney, Carrie A; Chambers, Tracey; Peacock, Shelley; Fisher, Kathryn; Ghosh, Sunita; Markle-Reid, Maureen; Swindle, Jennifer; Williams, Allison; Triscott, Jean Ac.

Publication Type Journal Article.

Cite + My Projects + Annotate

45.

Abstract Reference
Complete Reference

Unique Identifier 32255521

Title **Does Alzheimer's Disease and Related Dementias Modify Delirium Severity and Hospital Outcomes?.**

Find Similar
Find Citing Articles

Full Text

Source Journal of the American Geriatrics Society. 68(8):1722-1730, 2020 08.

Authors Hshieh TT; Fong TG; Schmitt EM; Marcantonio ER; Xu G; Gou YR; Travison TG; Metzger ED; Jones RN; Inouye SK; BASIL Study Group

Authors Full Name Hshieh, Tammy T; Fong, Tamara G; Schmitt, Eva M; Marcantonio, Edward R; Xu, Guoquan; Gou, Yun R; Travison, Thomas G; Metzger, Eran D; Jones, Richard N; Inouye, Sharon K; BASIL Study Group.

Publication Type Journal Article. Research Support, N.I.H., Extramural. Research Support, Non-U.S. Gov't.

Cite + My Projects + Annotate

46.

Abstract Reference
Complete Reference

Unique Identifier 32343401

Title **Implementation of Post-Acute Rehabilitation at Home: A Skilled Nursing Facility-Substitutive Model.**

Find Similar
Find Citing Articles

Full Text

Title Comment Comment in: J Am Geriatr Soc. 2020 Jul;68(7):1400-1401; PMID: 32343361 [https://www-ncbi-nlm-nih-gov.myaccess.library.utoronto.ca/pub...]

Source Journal of the American Geriatrics Society. 68(7):1584-1593, 2020 07.

Authors Augustine MR; Davenport C; Ornstein KA; Cuan M; Saenger P; Lubetsky S; Federman A; DeCherrie LV; Leff B; Siu AL

Authors Full Name Augustine, Matthew R; Davenport, Claire; Ornstein, Katherine A; Cuan, Mitchell; Saenger, Pamela; Lubetsky, Sara; Federman, Alex; DeCherrie, Linda V; Leff, Bruce; Siu, Albert L.

Publication Type Journal Article. Research Support, N.I.H., Extramural. Research Support, U.S. Gov't, Non-P.H.S.. Research Support, Non-U.S. Gov't.

Cite + My Projects + Annotate

47.

Unique Identifier	31169053
Title	**Influence of Cognition on Length of Stay and Rehospitalization in Older Veterans Admitted for Post-Acute Care.**
Source	Journal of Applied Gerontology. 39(6):609-617, 2020 06.
Authors	Stelmokas J; Rochette AD; Hogikyan R; Kitchen Andren KA; Reckow J; Sciaky A; Bieliauskas L; Alexander NB
Authors Full Name	Stelmokas, Julija; Rochette, Amber D; Hogikyan, Robert; Kitchen Andren, Katherine A; Reckow, Jaclyn; Sciaky, Alexandra; Bieliauskas, Linas; Alexander, Neil B.
Publication Type	Journal Article. Research Support, N.I.H., Extramural. Research Support, U.S. Gov't, Non-P.H.S..

Abstract Reference
Complete Reference
Find Similar
Find Citing Articles
Get it UTL

Cite + My Projects + Annotate

48.

Unique Identifier	32297239
Title	**The financial and social costs of delirium. [Review]**
Source	European Geriatric Medicine. 11(1):105-112, 2020 02.
Authors	Caplan GA; Teodorczuk A; Streatfeild J; Agar MR
Authors Full Name	Caplan, Gideon A; Teodorczuk, Andrew; Streatfeild, Jared; Agar, Meera R.
Publication Type	Journal Article. Review. Research Support, Non-U.S. Gov't.

Abstract Reference
Complete Reference
Find Similar
Find Citing Articles
Get it UTL

Cite + My Projects + Annotate

49.

Unique Identifier	34209409
Title	**Chronology of COVID-19 Symptoms in Very Old Patients: Study of a Hospital Outbreak.**
Source	Journal of Clinical Medicine. 10(13), 2021 Jun 30.
Authors	Lafuente-Lafuente C; Nghiem QD; Keravec H; Oukbir-Ferrag S; Magri M; Oquendo B; Donadio C; Rainone A; Belmin J
Authors Full Name	Lafuente-Lafuente, Carmelo; Nghiem, Quoc Duy; Keravec, Heloise; Oukbir-Ferrag, Sihem; Magri, Maurizio; Oquendo, Bruno; Donadio, Cristiano; Rainone, Antonio; Belmin, Joel.
Publication Type	Journal Article.

Abstract Reference
Complete Reference
Find Similar
Find Citing Articles
Full Text

Cite + My Projects + Annotate

50.

Unique Identifier	34064001
Title	**Profiling Delirium Progression in Elderly Patients via Continuous-Time Markov Multi-State Transition Models.**

Abstract Reference
Complete Reference
Find Similar
Find Citing Articles

**Source**	Journal of Personalized Medicine. 11(6), 2021 May 21.
**Authors**	Ocagli H; Azzolina D; Soltanmohammadi R; Aliyari R; Bottigliengo D; Acar AS; Stivanello L; Degan M; Baldi I; Lorenzoni G; Gregori D
**Authors Full Name**	Ocagli, Honoria; Azzolina, Danila; Soltanmohammadi, Rozita; Aliyari, Roqaye; Bottigliengo, Daniele; Acar, Aslihan Senturk; Stivanello, Lucia; Degan, Mario; Baldi, Ileana; Lorenzoni, Giulia; Gregori, Dario.
**Publication Type**	Journal Article.

Get it UTL

Cite + My Projects + Annotate

51.

Abstract Reference
Complete Reference
Find Similar
Find Citing Articles
Full Text

**Unique Identifier**	34082836
**Title**	**Involving frail older patients in identifying outcome measures for transitional care-a feasibility study.**
**Source**	Research Involvement & Engagement. 7(1):36, 2021 Jun 03.
**Authors**	Hansen TK; Jensen AL; Damsgaard EM; Rubak TMM; Jensen MEJ; Gregersen M
**Authors Full Name**	Hansen, Troels Kjaerskov; Jensen, Annesofie Lunde; Damsgaard, Else Marie; Rubak, Tone Maria Morck; Jensen, Mikkel Erik Juul; Gregersen, Merete.
**Publication Type**	Journal Article.

Cite + My Projects + Annotate

52.

Abstract Reference
Complete Reference
Find Similar
Find Citing Articles
Full Text

**Unique Identifier**	33241125
**Title**	**Social Vulnerability and Medical Complexity Among Medicare Beneficiaries Receiving Home Health Without Prior Hospitalization.**
**Source**	Innovation in Aging. 4(6):igaa049, 2020.
**Authors**	Burgdorf JG; Mroz TM; Wolff JL
**Authors Full Name**	Burgdorf, Julia G; Mroz, Tracy M; Wolff, Jennifer L.
**Publication Type**	Journal Article.

Cite + My Projects + Annotate

53.

Abstract Reference
Complete Reference
Find Similar
Find Citing Articles
Get it UTL

**Unique Identifier**	33994991
**Title**	**The Potential Role of Protein Kinase R as a Regulator of Age-Related Neurodegeneration. [Review]**
**Source**	Frontiers in aging neuroscience. 13:638208, 2021.
**Authors**	Martinez NW; Gomez FE; Matus S
**Authors Full**	Martinez, Nicolas W; Gomez, Felipe E; Matus, Soledad.

Name

Publication Type: Journal Article. Review.

Cite | + My Projects | + Annotate

54.

Abstract Reference
Complete Reference

Unique Identifier: 33953612

Title: **Managing Polypharmacy in Older Adults with Cancer Across Different Healthcare Settings. [Review]**

Source: Drug Healthcare & Patient Safety. 13:101-116, 2021.

Authors: Whitman A; Erdeljac P; Jones C; Pillarella N; Nightingale G

Authors Full Name: Whitman, Andrew; Erdeljac, Paige; Jones, Caroline; Pillarella, Nicole; Nightingale, Ginah.

Publication Type: Journal Article. Review.

Find Similar
Find Citing Articles

Get it UTL

Cite | + My Projects | + Annotate

55.

Abstract Reference
Complete Reference

Unique Identifier: 33935693

Title: **Case Report: The Complexities of Managing Medications and the Importance of Deprescribing Anticholinergics in Older Adults.**

Source: Frontiers in Pharmacology. 12:584667, 2021.

Authors: Elliott T; Eckmann L; Moga DC

Authors Full Name: Elliott, Taylor; Eckmann, Lynne; Moga, Daniela C.

Publication Type: Case Reports.

Find Similar
Find Citing Articles

Get it UTL

Cite | + My Projects | + Annotate

56.

Abstract Reference
Complete Reference

Unique Identifier: 31449295

Title: **Risk Factors and Outcomes Associated With Treatment of Asymptomatic Bacteriuria in Hospitalized Patients.**

Source: JAMA Internal Medicine. 179(11):1519-1527, 2019 Nov 01.

Authors: Petty LA; Vaughn VM; Flanders SA; Malani AN; Conlon A; Kaye KS; Thyagarajan R; Osterholzer D; Nielsen D; Eschenauer GA; Bloemers S; McLaughlin E; Gandhi TN

Authors Full Name: Petty, Lindsay A; Vaughn, Valerie M; Flanders, Scott A; Malani, Anurag N; Conlon, Anna; Kaye, Keith S; Thyagarajan, Rama; Osterholzer, Danielle; Nielsen, Daniel; Eschenauer, Gregory A; Bloemers, Sarah; McLaughlin, Elizabeth; Gandhi, Tejal N.

Publication Type: Journal Article.

Find Similar
Find Citing Articles

Get it UTL

Cite + My Projects + Annotate

57.

Abstract Reference
Complete Reference

Unique Identifier	33816752
Title	**Association of cognitive impairment severity with potentially avoidable readmissions: A retrospective cohort study of 8897 older patients.**
Source	Alzheimer's & Dementia : Diagnosis, Assessment & Disease Monitoring. 13(1):e12147, 2021.
Authors	Mitsutake S; Ishizaki T; Tsuchiya-Ito R; Furuta K; Hatakeyama A; Sugiyama M; Toba K; Ito H
Authors Full Name	Mitsutake, Seigo; Ishizaki, Tatsuro; Tsuchiya-Ito, Rumiko; Furuta, Ko; Hatakeyama, Akira; Sugiyama, Mika; Toba, Kenji; Ito, Hideki.
Publication Type	Journal Article.

Find Similar
Find Citing Articles

Full Text

Cite + My Projects + Annotate

58.

Abstract Reference
Complete Reference

Unique Identifier	33520321
Title	**Development of Neuroleptic Malignant Syndrome in a Patient with Lewy Body Dementia after Intramuscular Administration of Paliperidone.**
Source	Case Reports in Neurological Medicine Print. 2021:8879333, 2021.
Authors	Yeung HM; Schmitz S; Kvantaliani N; Martin C
Authors Full Name	Yeung, Ho-Man; Schmitz, Sarah; Kvantaliani, Nino; Martin, Christina.
Publication Type	Case Reports.

Find Similar
Find Citing Articles

Get it UTL

Cite + My Projects + Annotate

59.

Abstract Reference
Complete Reference

Unique Identifier	33415288
Title	**Transition Experiences of Caregivers of Older Adults With Dementia and Multiple Chronic Conditions: An Interpretive Description Study.**
Source	SAGE Open Nursing. 6:2377960820934290, 2020 Jan-Dec.
Authors	Lam A; Ploeg J; Carroll SL; Duggleby W; McAiney C; Julian P
Authors Full Name	Lam, Annie; Ploeg, Jenny; Carroll, Sandra L; Duggleby, Wendy; McAiney, Carrie; Julian, Patricia.
Publication Type	Journal Article.

Find Similar
Find Citing Articles

Get it UTL

Cite + My Projects + Annotate

60.

**Unique Identifier**	33392535	Abstract Reference<br>Complete Reference
**Title**	**Association of dementia diagnosis with urinary tract infection in the emergency department.**	Find Similar<br>Find Citing Articles
**Source**	Journal of the American College of Emergency Physicians open. 1(6):1291-1296, 2020 Dec.	Full Text
**Authors**	Yourman LC; Kent TJ; Israni JS; Ko KJ; Lesser A	
**Authors Full Name**	Yourman, Lindsey C; Kent, Tyler J; Israni, Juhi S; Ko, Kelly J; Lesser, Adriane.	
**Publication Type**	Journal Article.	

Cite + My Projects + Annotate

61.

**Unique Identifier**	33354590	Abstract Reference<br>Complete Reference
**Title**	**Revisiting the Role of Physicians in Assisted Living and Residential Care Settings.**	Find Similar<br>Find Citing Articles
**Source**	Gerontology & Geriatric Medicine. 6:2333721420979840, 2020 Jan-Dec.	Get it UTL
**Authors**	Dys S; Smith L; Tunalilar O; Carder P	
**Authors Full Name**	Dys, Sarah; Smith, Lindsey; Tunalilar, Ozcan; Carder, Paula.	
**Publication Type**	Journal Article.	

Cite + My Projects + Annotate

62.

**Unique Identifier**	32904844	Abstract Reference<br>Complete Reference
**Title**	**Promoting physical activity in geriatric patients with cognitive impairment after discharge from ward-rehabilitation: a feasibility study.**	Find Similar<br>Find Citing Articles
**Source**	European Journal of Ageing. 17(3):309-320, 2020 Sep.	Get it UTL
**Authors**	Eckert T; Bongartz M; Ullrich P; Abel B; Christian W; Kiss R; Hauer K	
**Authors Full Name**	Eckert, Tobias; Bongartz, Martin; Ullrich, Phoebe; Abel, Bastian; Christian, Werner; Kiss, Rainer; Hauer, Klaus.	
**Publication Type**	Journal Article.	

Cite + My Projects + Annotate

63.

**Unique Identifier**	32885187	Abstract Reference<br>Complete Reference
**Title**	**Study protocol for IMAGE: implementing multidisciplinary assessments for geriatric patients in an emergency department**	Find Similar<br>Find Citing Articles

observation unit, a hybrid effectiveness/implementation study using the Consolidated Framework for Implementation Research.

Get it UTL

Source	Implementation Science Communications. 1:28, 2020.
Authors	Southerland LT; Stephens JA; Carpenter CR; Mion LC; Moffatt-Bruce SD; Zachman A; Hill M; Caterino JM
Authors Full Name	Southerland, Lauren T; Stephens, Julie A; Carpenter, Christopher R; Mion, Lorraine C; Moffatt-Bruce, Susan D; Zachman, Angela; Hill, Michael; Caterino, Jeffrey M.
Publication Type	Journal Article.

Cite + My Projects + Annotate

64.

Abstract Reference
Complete Reference

Unique Identifier	32848345
Title	Challenges and Strategies for Managing Diabetes in the Elderly in Long-Term Care Settings.
Source	Diabetes Spectrum. 33(3):236-245, 2020 Aug.
Authors	Pandya N; Hames E; Sandhu S
Authors Full Name	Pandya, Naushira; Hames, Elizabeth; Sandhu, Sukhman.
Publication Type	Journal Article.

Find Similar
Find Citing Articles

Full Text

Cite + My Projects + Annotate

65.

Abstract Reference
Complete Reference

Unique Identifier	32565686
Title	The potential role of pomegranate and its nano-formulations on cerebral neurons in aluminum chloride induced Alzheimer rat model.
Source	Saudi Journal of Biological Sciences. 27(7):1710-1716, 2020 Jul.
Authors	Almuhayawi MS; Ramadan WS; Harakeh S; Al Jaouni SK; Bharali DJ; Mousa SA; Almuhayawi SM
Authors Full Name	Almuhayawi, Mohammed S; Ramadan, Wafaa S; Harakeh, Steve; Al Jaouni, Soad K; Bharali, Dhruba J; Mousa, Shaker A; Almuhayawi, Saad M.
Publication Type	Journal Article.

Find Similar
Find Citing Articles

Full Text

Cite + My Projects + Annotate

66.

Abstract Reference
Complete Reference

Unique Identifier	32140409
Title	Invasive pulmonary aspergillosis in a patient with cirrhosis.
Source	IDCases. 19:e00722, 2020.
Authors	Clark HL; Valencia HE; Findeis-Hosey JJ; Georas SN

Find Similar
Find Citing Articles

Get it UTL

Authors Full Name Clark, Heather L; Valencia, Hugo E; Findeis-Hosey, Jennifer J; Georas, Steve N.

Publication Type Journal Article.

Cite + My Projects + Annotate

67.

Abstract Reference
Complete Reference

Unique Identifier 34209673

Title *C9orf72* Intermediate Repeats Confer Genetic Risk for Severe COVID-19 Pneumonia Independently of Age.

Find Similar
Find Citing Articles

Source International Journal of Molecular Sciences. 22(13), 2021 Jun 29.

Full Text

Authors Zanella I; Zacchi E; Piva S; Filosto M; Beligni G; Alaverdian D; Amitrano S; Fava F; Baldassarri M; Frullanti E; Meloni I; Renieri A; Gen-Covid Multicenter Study; Gevacoba Study Group; Castelli F; Quiros-Roldan E

Authors Full Name Zanella, Isabella; Zacchi, Eliana; Piva, Simone; Filosto, Massimiliano; Beligni, Giada; Alaverdian, Diana; Amitrano, Sara; Fava, Francesca; Baldassarri, Margherita; Frullanti, Elisa; Meloni, Ilaria; Renieri, Alessandra; Gen-Covid Multicenter Study; Gevacoba Study Group; Castelli, Francesco; Quiros-Roldan, Eugenia.

Publication Type Journal Article.

Cite + My Projects + Annotate

68.

Abstract Reference
Complete Reference

Unique Identifier 34187380

Title Exploring life-space in the nursing home. An observational longitudinal study.

Find Similar
Find Citing Articles

Source BMC Geriatrics. 21(1):396, 2021 06 29.

Full Text

Authors Sverdrup K; Bergh S; Selbaek G; Benth JS; Husebo B; Roen IM; Thingstad P; Tangen GG

Authors Full Name Sverdrup, Karen; Bergh, Sverre; Selbaek, Geir; Benth, Jurate Saltyte; Husebo, Bettina; Roen, Irene Mari; Thingstad, Pernille; Tangen, Gro Gujord.

Publication Type Journal Article. Observational Study. Research Support, Non-U.S. Gov't.

Cite + My Projects + Annotate

69.

Abstract Reference
Complete Reference

Unique Identifier 33579284

Title Characteristics and mortality rates among patients requiring intermediate care: a national cohort study using linked databases.

Find Similar
Find Citing Articles

Source BMC Medicine. 19(1):48, 2021 02 12.

Full Text

Authors Evans CJ; Potts L; Dalrymple U; Pring A; Verne J; Higginson IJ; Gao

W; SPACE

Authors Full Name Evans, Catherine J; Potts, Laura; Dalrymple, Ursula; Pring, Andrew; Verne, Julia; Higginson, Irene J; Gao, Wei; SPACE.

Publication Type Journal Article. Research Support, Non-U.S. Gov't.

Cite + My Projects + Annotate

70.

Abstract Reference
Complete Reference

Unique Identifier 33739444

Title Management and outcomes of a COVID-19 outbreak in a nursing home with predominantly Black residents.

Find Similar
Find Citing Articles

Source Journal of the American Geriatrics Society. 69(5):1155-1165, 2021 05.

Full Text

Authors Beiting KJ; Huisingh-Scheetz M; Walker J; Graupner J; Martinchek M; Thompson K; Levine S; Gleason LJ

Authors Full Name Beiting, Kimberly J; Huisingh-Scheetz, Megan; Walker, Jacob; Graupner, Jeffrey; Martinchek, Michelle; Thompson, Katherine; Levine, Stacie; Gleason, Lauren J.

Publication Type Journal Article. Observational Study. Research Support, N.I.H., Extramural. Research Support, U.S. Gov't, P.H.S..

Cite + My Projects + Annotate

71.

Abstract Reference
Complete Reference

Unique Identifier 33524341

Title Two European Examples of Acute Geriatric Units Located Outside of a General Hospital for Older Adults With Exacerbated Chronic Conditions.

Find Similar
Find Citing Articles

Source Journal of the American Medical Directors Association. 22(6):1228-1234, 2021 06.

Get it UTL

Authors Ribbink ME; Gual N; MacNeil-Vroomen JL; Ars Ricart J; Buurman BM; Inzitari M; AGCH-Study Group

Authors Full Name Ribbink, Marthe E; Gual, Neus; MacNeil-Vroomen, Janet L; Ars Ricart, Joan; Buurman, Bianca M; Inzitari, Marco; AGCH-Study Group.

Publication Type Journal Article. Research Support, Non-U.S. Gov't.

Cite + My Projects + Annotate

72.

Abstract Reference
Complete Reference

Unique Identifier 33121870

Title Receipt of Timely Primary Care Services Following Post-Acute Skilled Nursing Facility Care.

Find Similar
Find Citing Articles

Source Journal of the American Medical Directors Association. 22(3):701-705.e1, 2021 03.

Get it UTL

Authors Simning A; Orth J; Caprio TV; Li Y; Wang J; Temkin-Greener H

Authors Full Name Simning, Adam; Orth, Jessica; Caprio, Thomas V; Li, Yue; Wang, Jinjiao; Temkin-Greener, Helena.

**Publication Type**	Journal Article. Research Support, N.I.H., Extramural. Research Support, Non-U.S. Gov't.

Cite + My Projects + Annotate

73.

Abstract Reference
Complete Reference

**Unique Identifier**	33082097
**Title**	**Using Standardized Tools to Characterize Adult Day Program Populations: Implications for Future Research and Clinical Practice.**
**Source**	Journal of the American Medical Directors Association. 22(5):1096-1100.e1, 2021 05.
**Authors**	Dharmakulaseelan L; Berall A; Santiago AT; Gardner S; Aleong R; Edelstein B; Karuza J; Blake C; Crawford S; Naglie G
**Authors Full Name**	Dharmakulaseelan, Laavanya; Berall, Anna; Santiago, Anna Theresa; Gardner, Sandra; Aleong, Rosanne; Edelstein, Beatrise; Karuza, Jurgis; Blake, Catherine; Crawford, Steve; Naglie, Gary.
**Publication Type**	Journal Article. Research Support, Non-U.S. Gov't.

Find Similar
Find Citing Articles
Get it UTL

Cite + My Projects + Annotate

74.

Abstract Reference
Complete Reference

**Unique Identifier**	32943341
**Title**	**Care Transitions to the Community from Veterans Affairs Nursing Homes: Experiences of Social Connection and Disconnection.**
**Source**	Journal of the American Medical Directors Association. 22(3):682-688, 2021 03.
**Authors**	Simons KV; Bower ES; Gillespie SM; Mills WL
**Authors Full Name**	Simons, Kelsey V; Bower, Emily S; Gillespie, Suzanne M; Mills, Whitney L.
**Publication Type**	Journal Article. Research Support, U.S. Gov't, Non-P.H.S..

Find Similar
Find Citing Articles
Get it UTL

Cite + My Projects + Annotate

75.

Abstract Reference
Complete Reference

**Unique Identifier**	32472324
**Title**	**Medication-related hospital admissions and readmissions in older patients: an overview of literature. [Review]**
**Source**	International Journal of Clinical Pharmacy. 42(5):1243-1251, 2020 Oct.
**Authors**	Linkens AEMJH; Milosevic V; van der Kuy PHM; Damen-Hendriks VH; Mestres Gonzalvo C; Hurkens KPGM
**Authors Full Name**	Linkens, A E M J H; Milosevic, V; van der Kuy, P H M; Damen-Hendriks, V H; Mestres Gonzalvo, C; Hurkens, K P G M.
**Publication Type**	Journal Article. Review.

Find Similar
Find Citing Articles
Get it UTL

Cite + My Projects + Annotate

76.

Abstract Reference
Complete Reference

Find Similar
Find Citing Articles

Full Text

Unique Identifier 31898134

Title Interventions to Prevent or Delay Long-Term Nursing Home Placement for Adults with Impairments-a Systematic Review of Reviews. [Review]

Source Journal of General Internal Medicine. 35(7):2118-2129, 2020 07.

Authors Duan-Porter W; Ullman K; Rosebush C; McKenzie L; Ensrud KE; Ratner E; Greer N; Shippee T; Gaugler JE; Wilt TJ

Authors Full Name Duan-Porter, Wei; Ullman, Kristen; Rosebush, Christina; McKenzie, Lauren; Ensrud, Kristine E; Ratner, Edward; Greer, Nancy; Shippee, Tetyana; Gaugler, Joseph E; Wilt, Timothy J.

Publication Type Journal Article. Research Support, U.S. Gov't, Non-P.H.S.. Review.

Cite + My Projects + Annotate

77.

Abstract Reference
Complete Reference

Find Similar
Find Citing Articles

Unique Identifier 33308926

Title Association of Therapy Time and Cognitive Recovery in Stroke Patients in Post-Acute Rehabilitation.

Title Comment Comment in: J Am Med Dir Assoc. 2021 Jun;22(6):1327-1328; PMID: 33640313 [https://www-ncbi-nlm-nih-gov.myaccess.library.utoronto.ca/pub...]
Comment in: J Am Med Dir Assoc. 2021 Jun;22(6):1328-1329; PMID: 33785311 [https://www-ncbi-nlm-nih-gov.myaccess.library.utoronto.ca/pub...]

Source Journal of the American Medical Directors Association. 22(2):453-458.e3, 2021 02.

Authors Cogan AM; Weaver JA; Davidson LF; Khromouchkine N; Mallinson T

Authors Full Name Cogan, Alison M; Weaver, Jennifer A; Davidson, Leslie F; Khromouchkine, Nikolai; Mallinson, Trudy.

Publication Type Journal Article. Observational Study. Research Support, U.S. Gov't, Non-P.H.S..

Cite + My Projects + Annotate

78.

Abstract Reference
Complete Reference

Find Similar
Find Citing Articles

Get it UTL

Unique Identifier 32948474

Title Initiation of Psycholeptic Medication During Hospitalization With Recommendation for Discontinuation After Discharge.

Source Journal of the American Medical Directors Association. 22(1):96-100.e5, 2021 01.

Authors Conti F; Consonni D; Damanti S; Nobili A; Pasina L; Mannucci PM; Cesari M; Rossi PD

Authors Full Conti, Federica; Consonni, Dario; Damanti, Sarah; Nobili, Alessandro;

**Name**	Pasina, Luca; Mannucci, Pier Mannuccio; Cesari, Matteo; Rossi, Paolo Dionigi.
**Publication Type**	Journal Article.

Cite + My Projects + Annotate

79.

Abstract Reference
Complete Reference

**Unique Identifier**	33951161
**Title**	**Age and frailty are independently associated with increased COVID-19 mortality and increased care needs in survivors: results of an international multi-centre study.**
**Source**	Age & Ageing. 50(3):617-630, 2021 05 05.
**Authors**	Geriatric Medicine Research Collaborative; Covid Collaborative; Welch C
**Authors Full Name**	Geriatric Medicine Research Collaborative; Covid Collaborative; Welch, Carly.
**Publication Type**	Journal Article. Multicenter Study.

Find Similar
Find Citing Articles
Get it UTL

Cite + My Projects + Annotate

80.

Abstract Reference
Complete Reference

**Unique Identifier**	33951153
**Title**	**Follow-up services for delirium after COVID-19-where now?.**
**Source**	Age & Ageing. 50(3):601-604, 2021 05 05.
**Authors**	Rahman S; Byatt K
**Authors Full Name**	Rahman, Shibley; Byatt, Kit.
**Publication Type**	Journal Article.

Find Similar
Find Citing Articles
Get it UTL

Cite + My Projects + Annotate

81.

Abstract Reference
Complete Reference

**Unique Identifier**	32482500
**Title**	**Development of a screening tool to identify patients likely to benefit from clinical pharmacist review in a home-based primary care population.**
**Source**	Journal of the American Pharmacists Association: JAPhA. 60(5):750-756, 2020 Sep - Oct.
**Authors**	Stewart AE; Lovato JF; Zimmer R; Stewart AP; Hinely MT; Yang M
**Authors Full Name**	Stewart, Amy E; Lovato, James F; Zimmer, Rachel; Stewart, Alyssa P; Hinely, Molly T; Yang, Mia.
**Publication Type**	Journal Article. Research Support, U.S. Gov't, P.H.S..

Find Similar
Find Citing Articles
Get it UTL

Cite + My Projects + Annotate

82.

Abstract Reference
Complete Reference

Unique Identifier	33739444
Title	**Management and outcomes of a COVID-19 outbreak in a nursing home with predominantly Black residents.**
Source	Journal of the American Geriatrics Society. 69(5):1155-1165, 2021 05.
Authors	Beiting KJ; Huisingh-Scheetz M; Walker J; Graupner J; Martinchek M; Thompson K; Levine S; Gleason LJ
Authors Full Name	Beiting, Kimberly J; Huisingh-Scheetz, Megan; Walker, Jacob; Graupner, Jeffrey; Martinchek, Michelle; Thompson, Katherine; Levine, Stacie; Gleason, Lauren J.
Publication Type	Journal Article. Observational Study. Research Support, N.I.H., Extramural. Research Support, U.S. Gov't, P.H.S..

Find Similar
Find Citing Articles

Full Text

Cite + My Projects + Annotate

83.

Abstract Reference
Complete Reference

Unique Identifier	32741644
Title	**Quality of Post-Acute Care in Skilled Nursing Facilities That Disproportionately Serve Hispanics With Dementia.**
Source	Journal of the American Medical Directors Association. 21(11):1705-1711.e3, 2020 11.
Authors	Rivera-Hernandez M; Fabius CD; Fashaw S; Downer B; Kumar A; Panagiotou OA; Epstein-Lubow G
Authors Full Name	Rivera-Hernandez, Maricruz; Fabius, Chanee D; Fashaw, Shekinah; Downer, Brian; Kumar, Amit; Panagiotou, Orestis A; Epstein-Lubow, Gary.
Publication Type	Journal Article. Research Support, N.I.H., Extramural.

Find Similar
Find Citing Articles

Get it UTL

Cite + My Projects + Annotate

84.

Abstract Reference
Complete Reference

Unique Identifier	31879184
Title	**Association Between Home Health Services and Facility Admission in Older Adults With and Without Alzheimer's Disease.**
Source	Journal of the American Medical Directors Association. 21(5):627-633.e9, 2020 05.
Authors	Wang J; Caprio TV; Simning A; Shang J; Conwell Y; Yu F; Li Y
Authors Full Name	Wang, Jinjiao; Caprio, Thomas V; Simning, Adam; Shang, Jingjing; Conwell, Yeates; Yu, Fang; Li, Yue.
Publication Type	Journal Article.

Find Similar
Find Citing Articles

Get it UTL

Cite + My Projects + Annotate

85.

Abstract Reference
Complete Reference

Unique Identifier: 33025500

Title: **Atypical clinical presentation of COVID-19 infection in residents of a long-term care facility.**

Source: European Geriatric Medicine. 11(6):1085-1088, 2020 12.

Authors: Blain H; Rolland Y; Benetos A; Giacosa N; Albrand M; Miot S; Bousquet J

Authors Full Name: Blain, Hubert; Rolland, Yves; Benetos, Athanase; Giacosa, Nadia; Albrand, Mylene; Miot, Stephanie; Bousquet, Jean.

Publication Type: Journal Article. Observational Study.

Find Similar
Find Citing Articles
Get it UTL

Cite + My Projects + Annotate

86.

Abstract Reference
Complete Reference

Unique Identifier: 32990494

Title: **Transitioning to Long-Term Care: Family Caregiver Experiences of Dementia, Communities, and Counseling.**

Title Comment: Erratum in: J Aging Health. 2021 Jun-Jul;33(5-6):NP1; PMID: 33983060 [https://www-ncbi-nlm-nih-gov.myaccess.library.utoronto.ca/pub...]

Source: Journal of Aging & Health. 33(1-2):133-146, 2021 01.

Authors: Zmora R; Statz TL; Birkeland RW; McCarron HR; Finlay JM; Rosebush CE; Gaugler JE

Authors Full Name: Zmora, Rachel; Statz, Tamara L; Birkeland, Robyn W; McCarron, Hayley R; Finlay, Jessica M; Rosebush, Christina E; Gaugler, Joseph E.

Publication Type: Journal Article. Randomized Controlled Trial. Research Support, N.I.H., Extramural.

Find Similar
Find Citing Articles
Get it UTL

Cite + My Projects + Annotate

87.

Abstract Reference
Complete Reference

Unique Identifier: 32962491

Title: **Change in Social Engagement among Incident Caregivers and Controls: Findings from the Caregiving Transitions Study.**

Source: Journal of Aging & Health. 33(1-2):114-124, 2021 01.

Authors: Liu C; Fabius CD; Howard VJ; Haley WE; Roth DL

Authors Full Name: Liu, Chelsea; Fabius, Chanee D; Howard, Virginia J; Haley, William E; Roth, David L.

Publication Type: Journal Article. Research Support, N.I.H., Extramural.

Find Similar
Find Citing Articles
Get it UTL

Cite + My Projects + Annotate

88.

Abstract Reference

Unique Identifier	32183993	Complete Reference
Title	**Development of delirium: Association with old age, severe burns, and intensive care.**	Find Similar<br>Find Citing Articles
Source	Burns. 46(4):797-803, 2020 06.	
Authors	Abdelrahman I; Vieweg R; Irschik S; Steinvall I; Sjoberg F; Elmasry M	Full Text
Authors Full Name	Abdelrahman, Islam; Vieweg, Rosa; Irschik, Stefan; Steinvall, Ingrid; Sjoberg, Folke; Elmasry, Moustafa.	
Publication Type	Journal Article. Research Support, Non-U.S. Gov't.	

Cite + My Projects + Annotate

89.

		Abstract Reference<br>Complete Reference
Unique Identifier	32955961	
Title	**Top Ten Tips Palliative Care Clinicians Should Know About Cognitive Impairment and Institutional Care.**	Find Similar<br>Find Citing Articles
Source	Journal of Palliative Medicine. 23(11):1525-1531, 2020 11.	
Authors	Schlogl M; Riese F; Little MO; Blum D; Jox RJ; O'Neill L; Pautex S; Piers R; Way D; Jones CA	Get it UTL
Authors Full Name	Schlogl, Mathias; Riese, Florian; Little, Milta O; Blum, David; Jox, Ralf J; O'Neill, Lynn; Pautex, Sophie; Piers, Ruth; Way, Deborah; Jones, Christopher A.	
Publication Type	Journal Article.	

Cite + My Projects + Annotate

90.

		Abstract Reference<br>Complete Reference
Unique Identifier	34079240	
Title	**Dementia Enlightened?! A Systematic Literature Review of the Influence of Indoor Environmental Light on the Health of Older Persons with Dementia in Long-Term Care Facilities.**	Find Similar<br>Find Citing Articles
Source	Clinical Interventions In Aging. 16:909-937, 2021.	Get it UTL
Authors	Goudriaan I; van Boekel LC; Verbiest MEA; van Hoof J; Luijkx KG	
Authors Full Name	Goudriaan, Ingrid; van Boekel, Leonieke C; Verbiest, Marjolein E A; van Hoof, Joost; Luijkx, Katrien G.	
Publication Type	Journal Article. Systematic Review.	

Cite + My Projects + Annotate

91.

		Abstract Reference<br>Complete Reference
Unique Identifier	32816865	
Title	**A pharmacist-led pilot using a performance dashboard to improve psychotropic medication use in a skilled nursing facility.**	Find Similar<br>Find Citing Articles

Source	BMJ Open Quality. 9(3), 2020 08.
Authors	Bell K; Hartmann C; Baughman AW
Authors Full Name	Bell, Kristin; Hartmann, Christine; Baughman, Amy Wisteria.
Publication Type	Journal Article.

Get it UTL

Cite + My Projects + Annotate

92.

Abstract Reference
Complete Reference

Unique Identifier	32594461
Title	**Cognitive status as a robust predictor of repeat falls in older Veterans in post-acute care.**
Source	Aging-Clinical & Experimental Research. 33(6):1677-1682, 2021 Jun.
Authors	Rochette AD; Alexander NB; Cigolle CT; Hogikyan R; Phillips K; Khan FA; Stelmokas J
Authors Full Name	Rochette, Amber D; Alexander, Neil B; Cigolle, Christine T; Hogikyan, Robert; Phillips, Kristin; Khan, Fareeha A; Stelmokas, Julija.
Publication Type	Journal Article.

Find Similar
Find Citing Articles

Get it UTL

Cite + My Projects + Annotate

93.

Abstract Reference
Complete Reference

Unique Identifier	32753058
Title	**Are there changes in medical specialist contacts after transition to a nursing home? an analysis of German claims data.**
Source	BMC Health Services Research. 20(1):716, 2020 Aug 04.
Authors	Spreckelsen O; Schmiemann G; Freitag MH; Fassmer AM; Engel B; Hoffmann F
Authors Full Name	Spreckelsen, Ove; Schmiemann, Guido; Freitag, Michael H; Fassmer, Alexander M; Engel, Bettina; Hoffmann, Falk.
Publication Type	Journal Article.

Find Similar
Find Citing Articles

Full Text

Cite + My Projects + Annotate

94.

Abstract Reference
Complete Reference

Unique Identifier	33188132
Title	**Cumulative health deficits, *APOE* genotype, and risk for later-life mild cognitive impairment and dementia.**
Source	Journal of Neurology, Neurosurgery & Psychiatry. 92(2):136-142, 2021 02.
Authors	Ward DD; Wallace LMK; Rockwood K
Authors Full Name	Ward, David D; Wallace, Lindsay M K; Rockwood, Kenneth.
Publication	Journal Article. Research Support, N.I.H., Extramural. Research

Find Similar
Find Citing Articles

Get it UTL

Type Support, Non-U.S. Gov't.

Cite + My Projects + Annotate

95.

Abstract Reference
Complete Reference

Find Similar
Find Citing Articles

Get it UTL

**Unique Identifier**	32362165
**Title**	**The Transition From Spousal Caregiver to Widowhood: Quantitative Findings of a Mixed-Methods Study.**
**Source**	Journal of the American Psychiatric Nurses Association. 26(6):527-541, 2020 Nov/Dec.
**Authors**	Groh CJ; Saunders MM
**Authors Full Name**	Groh, Carla J; Saunders, Mitzi M.
**Publication Type**	Journal Article. Research Support, N.I.H., Extramural.

Cite + My Projects + Annotate

96.

Abstract Reference
Complete Reference

Find Similar
Find Citing Articles

Full Text

**Unique Identifier**	32965034
**Title**	**Association of Positive Delirium Screening with Incident Dementia in Skilled Nursing Facilities.**
**Source**	Journal of the American Geriatrics Society. 68(12):2931-2936, 2020 12.
**Authors**	Briesacher BA; Koethe B; Olivieri-Mui B; Saczynski JS; Fick DM; Devlin JW; Marcantonio ER
**Authors Full Name**	Briesacher, Becky A; Koethe, Benjamin; Olivieri-Mui, Brianne; Saczynski, Jane S; Fick, Donna Marie; Devlin, John W; Marcantonio, Edward R.
**Publication Type**	Journal Article. Research Support, N.I.H., Extramural.

Cite + My Projects + Annotate

97.

Abstract Reference
Complete Reference

Find Similar
Find Citing Articles

Full Text

**Unique Identifier**	34030672
**Title**	**Facility and resident characteristics associated with variation in nursing home transfers: evidence from the OPTIMISTIC demonstration project.**
**Source**	BMC Health Services Research. 21(1):492, 2021 May 24.
**Authors**	Blackburn J; Balio CP; Carnahan JL; Fowler NR; Hickman SE; Sachs GA; Tu W; Unroe KT
**Authors Full Name**	Blackburn, Justin; Balio, Casey P; Carnahan, Jennifer L; Fowler, Nicole R; Hickman, Susan E; Sachs, Greg A; Tu, Wanzhu; Unroe, Kathleen T.
**Publication Type**	Journal Article.

Cite + My Projects + Annotate

98.

Unique Identifier 31941400

Title Relationship of Medicare-Medicaid Dual Eligibility and Dementia With Unplanned Facility Admissions Among Medicare Home Health Care Recipients.

Source Journal of Aging & Health. 32(9):1178-1187, 2020 10.

Authors Wang J; Caprio TV; Temkin-Greener H; Cai X; Simning A; Li Y

Authors Full Name Wang, Jinjiao; Caprio, Thomas V; Temkin-Greener, Helena; Cai, Xueya; Simning, Adam; Li, Yue.

Publication Type Journal Article.

Abstract Reference
Complete Reference

Find Similar
Find Citing Articles

Get it UTL

Cite | + My Projects | + Annotate

99.

Unique Identifier 32552857

Title The OPTIMIZE patient- and family-centered, primary care-based deprescribing intervention for older adults with dementia or mild cognitive impairment and multiple chronic conditions: study protocol for a pragmatic cluster randomized controlled trial.

Title Comment Comment in: J Gen Intern Med. 2021 Apr;36(4):1122; PMID: 33432430 [https://www-ncbi-nlm-nih-gov.myaccess.library.utoronto.ca/pub...]

Source Trials [Electronic Resource]. 21(1):542, 2020 Jun 18.

Authors Bayliss EA; Shetterly SM; Drace ML; Norton J; Green AR; Reeve E; Weffald LA; Wright L; Maciejewski ML; Sheehan OC; Wolff JL; Gleason KS; Kraus C; Maiyani M; Du Vall M; Boyd CM

Authors Full Name Bayliss, E A; Shetterly, S M; Drace, M L; Norton, J; Green, A R; Reeve, E; Weffald, L A; Wright, L; Maciejewski, M L; Sheehan, O C; Wolff, J L; Gleason, K S; Kraus, C; Maiyani, M; Du Vall, M; Boyd, C M.

Publication Type Clinical Trial Protocol. Journal Article.

Abstract Reference
Complete Reference

Find Similar
Find Citing Articles

Full Text

Cite | + My Projects | + Annotate

100.

Unique Identifier 32430843

Title Molecular Mechanisms of ER Stress and UPR in the Pathogenesis of Alzheimer's Disease. [Review]

Source Molecular Neurobiology. 57(7):2902-2919, 2020 Jul.

Authors Uddin MS; Tewari D; Sharma G; Kabir MT; Barreto GE; Bin-Jumah MN; Perveen A; Abdel-Daim MM; Ashraf GM

Authors Full Name Uddin, Md Sahab; Tewari, Devesh; Sharma, Gaurav; Kabir, Md Tanvir; Barreto, George E; Bin-Jumah, May N; Perveen, Asma; Abdel-Daim, Mohamed M; Ashraf, Ghulam Md.

Publication Type Journal Article. Review.

Abstract Reference
Complete Reference

Find Similar
Find Citing Articles

Get it UTL

Cite + My Projects + Annotate

All Range Clear 100 Per Page 1 Go Next ›

Print Email Export + My Projects Keep Selected

English Français Italiano Deutsch 日本語 繁體中文 Español 简体中文
한국어

About Us Contact Us Privacy Policy Terms of Use

© 2021 Ovid Technologies, Inc. All rights reserved. OvidUI_04.14.00.086, SourceID 90004a473d2e16b9a99bd726b1500dc38f7baefd

Search History/Alerts

Print Search History | Retrieve Searches | Retrieve Alerts | Save Searches / Alerts

Select / deselect all | Search with AND | Search with OR | Delete Searches | Re

Search ID#	Search Terms	Search Options	Actions
S37	S12 AND S19 AND S36	**Search modes** - Boolean/Phrase	**View Results** (825) **View Details**
S36	S20 OR S21 OR S22 OR S23 OR S24 OR S25 OR S26 OR S27 OR S28 OR S29 OR S30 OR S31 OR S32 OR S33 OR S34 OR S35	**Search modes** - Boolean/Phrase	**View Results** (123,798) **View Det**
S35	MH Delirium	**Search modes** - Boolean/Phrase	**View Results** (7,173) **View Detail**
S34	TI Deliri* OR AB Deliri*	**Search modes** - Boolean/Phrase	**View Results** (8,939) **View Detail**
S33	MH "Dementia, Senile"	**Search modes** - Boolean/Phrase	**View Results** (1,804) **View Detail**
S32	MH "Dementia, Presenile"	**Search modes** - Boolean/Phrase	**View Results** (112) **View Details**
S31	MH "Lewy Body Disease"	**Search modes** - Boolean/Phrase	**View Results** (1,180) **View Detail**
S30	MH "Dementia, Multi-Infarct"	**Search modes** - Boolean/Phrase	**View Results** (308) **View Details**
S29	MH "Dementia, Vascular"	**Search modes** - Boolean/Phrase	**View Results** (1,241) **View Detail**
S28	MH "AIDS Dementia Complex"	**Search modes** - Boolean/Phrase	**View Results** (552) **View Details**
S27	TI Alzheimer* OR AB Alzheimer*	**Search modes** - Boolean/Phrase	**View Results** (36,462) **View Deta**
S26	MH "Alzheimer's disease"	**Search modes** - Boolean/Phrase	**View Results** (34,050) **View Deta**
S25	MH Dementia	**Search modes** - Boolean/Phrase	**View Results** (42,577) **View Deta**
S24	TI Dement* OR AB Dement*	**Search modes** - Boolean/Phrase	**View Results** (57,225) **View Deta**
S23	TI (Major neurocognitive disorder*) OR AB (Major neurocognitive disorder*)	**Search modes** - Boolean/Phrase	**View Results** (86) **View Details**
S22	MH "Mild Cognitive Impairment"	**Search modes** - Boolean/Phrase	**View Results** (198) **View Details**
S21	TI (Mild neurocognitive disorder*) OR AB (Mild neurocognitive disorder*)	**Search modes** - Boolean/Phrase	**View Results** (63) **View Details**
S20	TI (Cognit* N3 impair*) OR AB (Cognit* N3 impair*)	**Search modes** - Boolean/Phrase	**View Results** (31,011) **View Deta**
S19	S13 OR S14 OR S15 OR S16 OR S17 OR S18	**Search modes** - Boolean/Phrase	**View Results** (1,220,037) **View D**
S18	MH "Aged, 80 and over"	**Search modes** - Boolean/Phrase	**View Results** (311,070) **View Det**
S17	MH Aged	**Search modes** - Boolean/Phrase	**View Results** (855,367) **View Det**
S16	TI Elder* OR AB Elder*	**Search modes** - Boolean/Phrase	**View Results** (106,097) **View Det**
S15	TI Senior* OR AB Senior*	**Search modes** - Boolean/Phrase	**View Results** (26,845) **View Deta**

☐	S14	TI ((Centenarian* or nonagenarian* or octogenarian* or geriatr* or gerontol* or senescen* or septuagenarian* or pensioner* or senile)) OR AB ((Centenarian* or nonagenarian* or octogenarian* or geriatr* or gerontol* or senescen* or septuagenarian* or pensioner* or senile))	**Search modes** - Boolean/Phrase	**View Results** (40,949)	**View Deta**
☐	S13	TI ((Old* or aged or aging)) OR AB ((Old* or aged or aging))	**Search modes** - Boolean/Phrase	**View Results** (488,060)	**View Det**
☐	S12	S1 OR S2 OR S3 OR S4 OR S5 OR S6 OR S7 OR S8 OR S9 OR S10 OR S11	**Search modes** - Boolean/Phrase	**View Results** (26,769)	**View Deta**
☐	S11	TI ((Convalesc* N3 (unit* or care or bed* or program* or ward* or setting* or facilit* or service* or model* or centre* or center* or home* or hospital*))) OR AB ((Convalesc* N3 (unit* or care or bed* or program* or ward* or setting* or facilit* or service* or model* or centre* or center* or home* or hospital*)))	**Search modes** - Boolean/Phrase	**View Results** (332)	**View Details**
☐	S10	TI ((Restor* N3 (unit* or care or bed* or program* or ward* or setting* or facilit* or service* or model* or centre* or center*))) OR AB ((Restor* N3 (unit* or care or bed* or program* or ward* or setting* or facilit* or service* or model* or centre* or center*)))	**Search modes** - Boolean/Phrase	**View Results** (1,776)	**View Detail**
☐	S9	MH Skilled Nursing Facilities	**Search modes** - Boolean/Phrase	**View Results** (4,513)	**View Detail**
☐	S8	TI (((Skilled Nursing) N3 (unit* or bed* or program* or ward* or setting* or facilit* or service* or model* or centre* or center*))) OR AB (((Skilled Nursing) N3 (unit* or bed* or program* or ward* or setting* or facilit* or service* or model* or centre* or center*)))	**Search modes** - Boolean/Phrase	**View Results** (2,846)	**View Detail**
☐	S7	TI (((Post acute) N3 (unit* or care or bed* or program* or ward* or setting* or facilit* or service* or model* or centre* or center*))) OR AB (((Post acute) N3 (unit* or care or bed* or program* or ward* or setting* or facilit* or service* or model* or centre* or center*)))	**Search modes** - Boolean/Phrase	**View Results** (1,550)	**View Detail**
☐	S6	TI (((Postacute or post-acute) N3 (unit* or care or bed* or program* or ward* or setting* or facilit* or service* or model* or centre* or center*))) OR AB (((Postacute or post-acute) N3 (unit* or care or bed* or program* or ward* or setting* or facilit* or service* or model* or centre* or center*)))	**Search modes** - Boolean/Phrase	**View Results** (2,326)	**View Detail**
☐	S5	MH Subacute care	**Search modes** - Boolean/Phrase	**View Results** (1,788)	**View Detail**
☐	S4		**Search modes** - Boolean/Phrase		

		TI (((Subacute or sub-acute) N3 (unit* or care or bed* or program* or ward* or setting* or facilit* or service* or model* or centre* or center*))) OR AB (((Subacute or sub-acute) N3 (unit* or care or bed* or program* or ward* or setting* or facilit* or service* or model* or centre* or center*)))		View Results (1,092) | View Detail
	S3	TI ((Intermediate N3 (unit* or care or bed* or program* or ward* or setting* or facilit* or service* or model* or centre* or center*))) OR AB ((Intermediate N3 (unit* or care or bed* or program* or ward* or setting* or facilit* or service* or model* or centre* or center*)))	**Search modes** - Boolean/Phrase	View Results (1,821) | View Detail
	S2	MH Transitional Care	**Search modes** - Boolean/Phrase	View Results (2,172) | View Detail
	S1	TI ((Transition* N3 (care* or unit* or bed* or program* or ward* or setting* or facilit* or service* or model* or centre* or center*))) OR AB ((Transition* N3 (care* or unit* or bed* or program* or ward* or setting* or facilit* or service* or model* or centre* or center*)))	**Search modes** - Boolean/Phrase	View Results (13,279) | View Deta

1. The impact of **dementia** on **aged care** service **transitions** in the last five years of life.

(includes abstract) Welberry, Heidi J; Jorm, Louisa R; Barbieri, Sebastiano; Hsu, Benjumin; Brodaty, Henry; Age & **Ageing**, Jul2021; 50(4): 1159-1165. 7p. (Article) ISSN: 0002-0729

2. Participation of persons with **dementia** and their **caregivers** in research.

(includes abstract) Frank, Lori; Jennings, Lee A.; Petersen, Ronald C.; Majid, Tabassum; Gilmore-Bykovskyi, Andrea; Schicker, Lonni; Karlawish, Jason; Journal of the American Geriatrics Society, Jul2021; 69(7): 1784-1792. 9p. (Article) ISSN: 0002-8614

3. Corrigendum...Zmora, R., Statz, T. L., Birkeland, R. W., McCarron, H. R., Finlay, J. M., Rosebush, C. E., and Gaugler, J. E. (2021). **Transitioning** to Long-Term **Care**: Family **Caregiver** Experiences of **Dementia**, Communities, and Counseling. Journal of **Aging** and Health, 33(1-2), 133-146.

(includes abstract) *In:* Journal of **Aging** & Health; Jun/Jul2021; v.33. n.5/6, NP1-NP1. 1p. (Correction Notice - corrected article) ISSN: 0898-2643

Subjects: Dementia Patients; **Transitional Care**; Family; **Caregiver** Attitudes; Professional-Family Relations

4. Patient-Centered **Care**: Transforming the Health **Care** System in Vietnam With Support of Digital Health Technology.

(includes abstract) Dang, Thu Ha; Nguyen, Tuan Anh; Van, Minh Hoang; Santin, Olinda; Tran, Oanh Mai Thi; Schofield, Penelope; Hoang Van, Minh; Journal of Medical Internet Research, Jun2021; 23(6): N.PAG-N.PAG. 1p. (journal article) ISSN: 1438-8871 PMID: NLM34085939

5. Two European Examples of **Acute Geriatric** Units Located Outside of a General **Hospital** for **Older** Adults With Exacerbated Chronic Conditions.

(includes abstract) Ribbink, Marthe E.; Gual, Neus; MacNeil-Vroomen, Janet L.; Ars Ricart, Joan; Buurman, Bianca M.; Inzitari, Marco; Journal of the American Medical Directors Association, Jun2021; 22(6): 1228-1234. 7p. (Article - research) ISSN: 1525-8610

Subjects: Chronic Disease Therapy; **Gerontologic Care**; **Subacute Care**; **Hospital** Units; Health **Services** Accessibility; Patient Admission; Treatment Outcomes; **Aged**: 65+ years; **Aged**, 80 & over

6. **Facility** and resident characteristics associated with variation in **nursing home** transfers: evidence from the OPTIMISTIC demonstration project.

(includes abstract) Blackburn, Justin; Balio, Casey P.; Carnahan, Jennifer L.; Fowler, Nicole R.; Hickman, Susan E.; Sachs, Greg A.; Tu, Wanzhu; Unroe, Kathleen T.; BMC Health **Services** Research, 5/24/2021; 21(1): 1-12. 12p. (journal article) ISSN: 1472-6963 PMID: NLM34030672

Subjects: Nursing Homes; Medicare; **Aged**: 65+ years; **Aged**, 80 & over

7. Follow-up **services** for **delirium** after COVID-19—where now?

(includes abstract) Rahman, Shibley; Byatt, Kit; Age & **Ageing**, May2021; 50(3): 601-604. 4p. (Article) ISSN: 0002-0729

Subjects: After **Care**; Mental Health **Services**; **Delirium** Psychosocial Factors; COVID-19 Symptoms; **Aged**: 65+ years

8. Age and frailty are independently associated with increased COVID-19 mortality and increased **care** needs in survivors: results of an international multi-**centre** study.

(includes abstract) Collaborative, **Geriatric** Medicine Research; Collaborative, Covid; Welch, Carly; Age & **Ageing**, May2021; 50(3): 617-630. 14p. (Article - research, tables/charts) ISSN: 0002-0729

Subjects: COVID-19 Mortality; Frailty Syndrome; Age Factors; Survivors; **Transitional Care**; Health **Services** Needs and Demand; Risk Assessment; Adult: 19-44 years; Middle **Aged**: 45-64 years; **Aged**: 65+ years; **Aged**, 80 & over; Female; Male

9. Risk factors of readmission after **geriatric hospital care**: An interRAI-based cohort study in Finland.

(includes abstract) Kerminen, Hanna M.; Jäntti, Pirkko O.; Valvanne, Jaakko N.A.; Huhtala, Heini S.A.; Jämsen, Esa R.K.; Archives of Gerontology & Geriatrics, May2021; 94 N.PAG-N.PAG. 1p. (Article - research) ISSN: 0167-4943

Subjects: Readmission

10. Validation of the **Delirium** Diagnostic Tool-Provisional (DDT-Pro) in a **skilled nursing facility** and comparison to the 4 'A's test (4AT).

(includes abstract) Sepúlveda, Esteban; Bermúdez, Ester; González, Dulce; Cotino, Paula; Viñuelas, Eva; Palma, José; Ciutat, Marta; Grau, Imma; Vilella, Elisabet; Trzepacz, Paula T.; Franco, José G.; General **Hospital** Psychiatry, May2021; 70 116-123. 8p. (Article - research) ISSN: 0163-8343

Subjects: Skilled Nursing Facilities; **Delirium** Diagnosis; Clinical Assessment Tools Evaluation; Instrument Validation; Predictive Value of Tests; **Aged**: 65+ years

11. Successful Community Discharge Among **Older** Adults With Traumatic Brain Injury in **Skilled Nursing Facilities**.

(includes abstract) Evans, Emily; Gutman, Roee; Resnik, Linda; Zonfrillo, Mark R.; Lueckel, Stephanie N.; Kumar, Raj G.; DeVone, Frank; Dams-O'Connor, Kristen; Thomas, Kali S.; Journal of Head Trauma Rehabilitation, May/Jun2021; 36(3): E186-E198. 13p. (Article - research, tables/charts) ISSN: 0885-9701

Subjects: Severity of Injury Adverse Effects; Functional Status In **Old** Age; Patient Discharge In **Old** Age; Community Living In **Old** Age; **Skilled Nursing Facilities**; **Aged**, Hospitalized; Brain Injuries Rehabilitation; **Aged**, 80 & over; **Aged**: 65+ years; Male; Female

12. Management and outcomes of a COVID-19 outbreak in a **nursing home** with predominantly Black residents.

(includes abstract) Beiting, Kimberly J.; Huisingh-Scheetz, Megan; Walker, Jacob; Graupner, Jeffrey; Martinchek, Michelle; Thompson, Katherine; Levine, Stacie; Gleason, Lauren J.; Journal of the American Geriatrics Society, May2021; 69(5): 1155-1165. 11p. (Article - algorithm, critical path, research, tables/charts) ISSN: 0002-8614

Subjects: COVID-19 Drug Therapy; COVID-19 Therapy; Critical Path; Black Persons; **Skilled Nursing Facilities**; Urban Areas; Treatment Outcomes Evaluation; **Aged**: 65+ years

13. Using Standardized Tools to Characterize Adult Day **Program** Populations: Implic[illegible]ons for Future Research and Clinical Practice.

(includes abstract) Dharmakulaseelan, Laavanya; Berall, Anna; Santiago, Anna Theresa; Gardner, Sandra; Aleong, Rosanne; Edelstein, Beatrise; Karuza, Jurgis; Blake, Catherine; Crawford, Steve; Naglie, Gary; Journal of the American Medical Directors Association, May2021; 22(5): 1096-1096. 1p. (Article - research) ISSN: 1525-8610

Subjects: Day **Care** In Adulthood; Data Collection Methods; Physician Attitudes; Self Report; Adult: 19-44 years; **Aged**: 65+ years; **Aged**, 80 & over; Female; Male

14. **Older** Adults with Mental Illness or **Dementia** Struggle with the **Skilled Nursing Facility**-to-**Home Transition**.

Simning, Adam; Orth, Jessica; Temkin-Greener, Helena; Li, Yue; Simons, Kelsey; Conwell, Yeates; *In:* American Journal of **Geriatric** Psychiatry; 2021 Supplement; v.29. n.4, S132-S132. 1p. (Abstract) ISSN: 1064-7481

15. Factors Associated With Discharge Destination in Community-Dwelling Adults Ad[illegible]tted to **Acute** General Medical Units.

(includes abstract) D'Souza, Aruska N.; Granger, Catherine L.; Patrick, Cameron J.; Kay, Jacqueline E.; Said, Catherine M.; Journal of **Geriatric** Physical Therapy, Apr-Jun2021; 44(2): 94-100. 7p. (Article - research, tables/charts) ISSN: 1539-8412

Subjects: Community Living In **Old** Age; Patient Discharge Psychosocial Factors; **Acute Care**; **Hospital** Units; Patient Admission Psychosocial Factors; **Aged**: 65+ years; **Aged**, 80 & over; Male; Female

16. Successful Discharge to Community Gap of FFS Medicare Beneficiaries With and Without ADRD Narrowed.

(includes abstract) Bardenheier, Barbara H.; Rahman, Momotazur; Kosar, Cyrus; Werner, Rachel M.; Mor, Vincent; Journal of the American Geriatrics Society, Apr2021; 69(4): 972-978. 7p. (Article - research, tables/charts) ISSN: 0002-8614

Subjects: **Alzheimer's** Disease; **Dementia**; Medicare; Patient Discharge; **Skilled Nursing Facilities**; Fee for Service Plans; Billing and Claims; Middle **Aged**: 45-64 years; **Aged**: 65+ years; Female; Male

17. Redefining **geriatric** trauma: 55 is the new 65.

(includes abstract) Fakhry, Samir M.; Morse, Jennifer L.; Garland, Jeneva M.; Wilson, Nina Y.; Shen, Yan; Wyse, Ransom J.; Watts, Dorraine D.; Journal of Trauma & **Acute Care** Surgery, Apr2021; 90(4): 738-743. 6p. (journal article - research) ISSN: 2163-0755 PMID: NLM33740785

Subjects: Wounds, Penetrating Mortality; Wounds, Nonpenetrating Mortality; Middle **Aged**: 45-64 years; **Aged**, 80 & over; Adult: 19-44 years; **Aged**: 65+ years; Female; Male

18. Understanding **transitional care** programs for **older** adults who experience delayed discharge: a scoping review.

(includes abstract) McGilton, Katherine S.; Vellani, Shirin; Krassikova, Alexandra; Robertson, Sheryl; Irwin, Constance; Cumal, Alexia; Bethell, Jennifer; Burr, Elaine; Keatings, Margaret; McKay, Sandra; Nichol, Kathryn; Puts, Martine; Singh, Anita; Sidani, Souraya; BMC Geriatrics, 3/29/2021; 21(1): 1-18. 18p. (journal article - research) ISSN: 1471-2318 PMID: NLM33781222

Subjects: Aged: 65+ years; Female

19. Challenges to Admitting Residents: Perspectives from Rural **Nursing Home** Administrators and Staff.

(includes abstract) Henning-Smith, Carrie; Cross, Dori; Rahman, Adrita; Inquiry (00469580), 3/26/2021; 58 1-8. 8p. (Article - research, tables/charts) ISSN: 0046-9580

Subjects: Nursing Homes Administration; Rural Areas; Administrative Personnel; **Nursing Home** Personnel; Attitude of Health Personnel; Patient Admission

20. **Delirium** and Functional Recovery in Patients Discharged to **Skilled Nursing Facilities** After Hospitalization for Heart Failure.

(includes abstract) Madrigal, Caroline; Kim, Jenny; Jiang, Lan; Lafo, Jacob; Bozzay, Melanie; Primack, Jennifer; Correia, Stephen; Erqou, Sebhat; Wu, Wen-Chih; Rudolph, James L.; JAMA Network Open, 3/16/2021; 4(3): e2037968-e2037968. 1p. (Article - research, tables/charts)

Subjects: Heart Failure; **Skilled Nursing Facilities**; **Delirium**; Recovery; Functional Status; **Aged**: 65+ years; **Aged**, 80 & over; Male; Female

21. What Factors Predict Adverse Discharge Disposition in Patients **Older** Than 60 Years Undergoing Lower-extremity Surgery? The Adverse Discharge in **Older** Patients after Lower-extremity Surgery (ADELES) Risk Score.

(includes abstract) Schaefer, Maximilian S.; Hammer, Maximilian; Platzbecker, Katharina; Santer, Peter; Grabitz, Stephanie D.; Murugappan, Kadhiresan R.; Houle, Tim; Barnett, Sheila; Rodriguez, Edward K.; Eikermann, Matthias; Clinical Orthopaedics & Related Research®, Mar2021; 479(3): 546-557. 12p. (journal article) ISSN: 0009-921X PMID: NLM33196587

22. **Care Transitions** to the Community from Veterans Affairs **Nursing** Homes: Experiences of Social Connection and Disconnection.

(includes abstract) Simons, Kelsey V.; Bower, Emily S.; Gillespie, Suzanne M.; Mills, Whitney L.; Journal of the American Medical Directors Association, Mar2021; 22(3): 682-688. 7p. (Article - research, tables/charts) ISSN: 1525-8610

Subjects: Transitional Care; Veterans Psychosocial Factors; **United** States Department of Veterans Affairs; **Nursing** Homes; Social Behavior

23. Receipt of Timely Primary **Care Services** Following **Post-Acute Skilled Nursing Facility Care**.

(includes abstract) Simning, Adam; Orth, Jessica; Caprio, Thomas V.; Li, Yue; Wang, Jinjiao; Temkin-Greener, Helena; Journal of the American Medical Directors Association, Mar2021; 22(3): 701-701. 1p. (Article - research, tables/charts) ISSN: 1525-8610

Subjects: Primary Health **Care**; **Skilled Nursing Facilities**; Health **Services** Accessibility; **Transitional Care**; Continuity of Patient **Care**; **Subacute Care**; Middle **Aged**: 45-64 years; **Aged**: 65+ years

24. Characteristics and mortality rates among patients requiring **intermediate care**: a national cohort study using linked databases.

(includes abstract) Evans, Catherine J.; Potts, Laura; Dalrymple, Ursula; Pring, Andrew; Verne, Julia; Higginson, Irene J.; Gao, Wei; on behalf of SPACE; Banerjee, Sube; Dawkins, Marsha; Ellis-Smith, Clare; Goodman, Claire; Norton, Christine; Maddocks, Mathew; Seamark, David; SPACE; BMC Medicine, 2/12/2021; 19(1): 1-13. 13p. (journal article - research) ISSN: 1741-7015 PMID: NLM33579284

25. The Frailty In Residential Sector over Time (FIRST) study: methods and baseline cohort description.

(includes abstract) Jadczak, Agathe Daria; Robson, Leonie; Cooper, Tina; Bell, J. Simon; Visvanathan, Renuka; on behalf of the FIRST Study Collaborators; Karnon, Jonathan; Afzali, Hossein Hajiali; Theou, Olga; Yu, Solomon; Milte, Rachel; Inacio, Maria; Ratcliffe, Julie; Wilson, David; Tucker, Graeme; Liau, Shin; Thompson, Mark Q.; FIRST Study Collaborators; BMC Geriatrics, 2/3/2021; 21(1): 1-12. 12p. (journal article - research) ISSN: 1471-2318 PMID: NLM33535968

Subjects: **Aged**: 65+ years; **Aged**, 80 & over; Female; Male

26. Association of Therapy Time and **Cognitive** Recovery in Stroke Patients in **Post-Acute** Rehabilitation.

(includes abstract) Cogan, Alison M.; Weaver, Jennifer A.; Davidson, Leslie F.; Khromouchkine, Nikolai; Mallinson, Trudy; Journal of the American Medical Directors Association, Feb2021; 22(2): 453-453. 1p. (Article - research, tables/charts) ISSN: 1525-8610

Subjects: Treatment Duration; Cognition; Recovery; Stroke Patients; Stroke Rehabilitation; Length of Stay; **Aged**: 65+ years

27. Severe heat stroke complicated by multiple cerebral infarctions: a case report.

(includes abstract) Kamidani, Ryo; Okada, Hideshi; Kitagawa, Yuichiro; Kusuzawa, Keigo; Ichihashi, Masahiro; Kakino, Yoshinori; Oiwa, Hideaki; Yasuda, Ryu; Fukuta, Tetsuya; Yoshiyama, Naomasa; Miyake, Takahito; Okamoto, Haruka; Suzuki, Kodai; Yamada, Noriaki; Doi, Tomoaki; Yoshida, Takahiro; Ushikoshi, Hiroaki; Kumada, Keisuke; Yoshida, Shozo; Ogura, Shinji; Journal of Medical Case Reports, 1/28/2021; 15(1): 1-6. 6p. (journal article - case study) ISSN: 1752-1947 PMID: NLM33504362

Subjects: Heat Stroke Complications; Disseminated Intravascular Coagulation; Heat Stroke Therapy; **Aged**, 80 & over; Female

28. A qualitative systematic review of experiences of persons with **dementia** regarding **transition** to long-term **care**.

(includes abstract) Young, Jessica A; Lind, Christopher; Orange, JB; **Dementia** (14713012), Jan2021; 20(1): 5-27. 23p. (Article - research, systematic review, tables/charts) ISSN: 1471-3012

Subjects: Dementia Patients Psychosocial Factors; Long Term **Care**; **Transitional Care**; Patient Attitudes Evaluation

29. Initiation of Psycholeptic Medication During Hospitalization With Recommendation for Discontinuation After Discharge.

(includes abstract) Conti, Federica; Consonni, Dario; Damanti, Sarah; Nobili, Alessandro; Pasina, Luca; Mannucci, Pier Mannuccio; Cesari, Matteo; Rossi, Paolo Dionigi; Journal of the American Medical Directors Association, Jan2021; 22(1): 96-96. 1p. (Article - research, tables/charts) ISSN: 1525-8610

Subjects: Psychotropic Drugs Administration and Dosage; Hospitalization; Patient Discharge; Medication Compliance; **Dementia**; **Aged**: 65+ years; **Aged**, 80 & over

30. Family Communication in Long-Term **Care** During a Pandemic: Lessons for Enhancing Emotional Experiences.

(includes abstract) Monin, Joan K.; Ali, Talha; Syed, Sumaiyah; Piechota, Amanda; Lepore, Michael; Mourgues, Catalina; Gaugler, Joseph E.; Marottoli, Richard; David, Daniel; American Journal of **Geriatric** Psychiatry, Dec2020; 28(12): 1299-1307. 9p. (journal article) ISSN: 1064-7481 PMID: NLM33004262

Subjects: Long Term **Care** Trends; Long Term **Care**; Disease Outbreaks; Coronavirus Infections Epidemiology; Long Term **Care** Psychosocial Factors; Communication; Family Psychosocial Factors; Attitude of Health Personnel; Pneumonia, Viral Psychosocial Factors; Pneumonia, Viral Epidemiology; Coronavirus Infections Psychosocial Factors; **Aged**: 65+ years; Adult: 19-44 years; Male; Female

31. Association of Positive **Delirium** Screening with Incident **Dementia** in **Skilled Nursing Facilities**.

(includes abstract) Briesacher, Becky A.; Koethe, Benjamin; Olivieri-Mui, Brianne; Saczynski, Jane S.; Fick, Donna Marie; Devlin, John W.; Marcantonio, Edward R.; Journal of the American Geriatrics Society, Dec2020; 68(12): 2931-2936. 6p. (Article - research, tables/charts) ISSN: 0002-8614

Subjects: Skilled Nursing Facilities; **Dementia** Diagnosis; **Delirium** Diagnosis; Health Screening

32. Factors associated with success in **transition care services** among **older** people in Australia.

(includes abstract) Cations, Monica; Lang, Catherine; Crotty, Maria; Wesselingh, Steven; Whitehead, Craig; Inacio, Maria C.; BMC Geriatrics, 11/23/2020; 20(1): N.PAG-N.PAG. 1p. (journal article - research) ISSN: 1471-2318 PMID: NLM33228558

Subjects: Aged: 65+ years; **Aged**, 80 & over; Female

33. **Caregivers** of **older** adults with **dementia** and multiple chronic conditions: Exploring their experiences with significant changes.

(includes abstract) Ploeg, Jenny; Northwood, Melissa; Duggleby, Wendy; McAiney, Carrie A; Chambers, Tracey; Peacock, Shelley; Fisher, Kathryn; Ghosh, Sunita; Markle-Reid, Maureen; Swindle, Jennifer; Williams, Allison; Triscott, Jean AC; **Dementia** (14713012), Nov2021; 19(8): 2601-2620. 20p. (Article - research, tables/charts) ISSN: 1471-3012

Subjects: Dementia In **Old** Age; Chronic Disease In **Old** Age; **Caregivers**; Professional Role; **Caregiver** Attitudes; Coping; **Aged**, 80 & over; Middle **Aged**: 45-64 years; **Aged**: 65+ years; Male; Female

34. **Delirium** in **elderly** patients: Prospective prevalence across **hospital services**.

(includes abstract) Fuchs, Simon; Bode, Leonie; Ernst, Jutta; Marquetand, Justus; von Känel, Roland; Böttger, Sönke; General **Hospital** Psychiatry, Nov2020; 67 19-25. 7p. (Article - research, tables/charts) ISSN: 0163-8343

Subjects: Delirium Epidemiology; Hospitalization; Health **Services** Accessibility; Prevalence; **Aged**: 65+ years

35. Top Ten Tips Palliative **Care** Clinicians Should Know About **Cognitive Impairment** and Institutional **Care**.

(includes abstract) Schlögl, Mathias; Riese, Florian; Little, Milta O.; Blum, David; Jox, Ralf J.; O'Neill, Lynn; Pautex, Sophie; Piers, Ruth; Way, Deborah; Jones, Christopher A.; Journal of Palliative Medicine, Nov2020; 23(11): 1525-1531. 7p. (Article) ISSN: 1096-6218

Subjects: Advance **Care** Planning; Palliative **Care**; Cognition **Disorders**; Institutionalization; **Dementia** Therapy

36. Changing needs in advanced **dementia**.

(includes abstract) Brown, Margaret; Tolson, Debbie; Ritchie, Louise; **Nursing Older** People, Nov2020; 32(4): 14-20. 7p. (Article) ISSN: 1472-0795

Subjects: Dementia Diagnosis; **Dementia** Physiopathology; **Dementia** Patients; **Aged**: 65+ years

37. Making the Case for Centralized **Dementia Care** Through Adaptive Reuse in the Time of COVID-19.

(includes abstract) Roberts, Emily; Carter, Heather Carlile; Inquiry (00469580), 10/30/2020; 1-6. 6p. (Article - editorial, pictorial) ISSN: 0046-9580

Subjects: Hospital Design and Construction; Environmental Sustainability; Community Mental Health **Services**; **Dementia** Therapy; Housing for the **Elderly**; **Gerontologic Care**; COVID-19 Pandemic

38. The impact of **dementia** and language on hospitalizations: a retrospective cohort [illegible] long-term **care** residents.

(includes abstract) Riad, Karine; Webber, Colleen; Batista, Ricardo; Reaume, Michael; Rhodes, Emily; Knight, Braden; Prud'homme, Denis; Tanuseputro, Peter; BMC Geriatrics, 10/8/2020; 20(1): N.PAG-N.PAG. 1p. (journal article - research) ISSN: 1471-2318 PMID: NLM33032528

Subjects: Dementia Therapy; **Dementia** Epidemiology; Long Term **Care**; **Dementia** Diagnosis; **Aged**: 65+ years

39. The license plate test performance in Canadian adolescents with learning disabilit[illegible]s: A preliminary study.

(includes abstract) Harrison, Allyson G.; Armstrong, Irene; Applied Neuropsychology: Child, Oct-Dec2020; 9(4): 360-366. 7p. (journal article) ISSN: 2162-2965 PMID: NLM32286886

40. Interventions to optimise **transitional care** coordination for **older** people living wit[illegible] **dementia** and concomitant multimorbidity and their **caregivers**: A systematic review.

(includes abstract) Parker, Kirsten J.; Hickman, Louise D.; Phillips, Jane L.; Ferguson, Caleb; Contemporary Nurse: A Journal for the Australian **Nursing** Profession, Oct-Dec2020; 56(5/6): 505-533. 29p. (Article - research, systematic review, tables/charts) ISSN: 1037-6178

Subjects: Dementia Therapy; Comorbidity In **Old** Age; **Transitional Care**; **Caregivers**; **Aged**: 65+ years

41. Resident-to-Resident Aggression in Long-Term **Care**: Analysis of Structured and Unstructured Data From the National Violent Death Reporting System, 2003-2016.

(includes abstract) DeBois, Kristen A.; Evans, Shelly D.; Chatfield, Sheryl L.; Journal of Applied Gerontology, Oct2020; 39(10): 1069-1077. 9p. (Article - research, tables/charts) ISSN: 0733-4648

Subjects: Long Term **Care**; **Nursing Home** Patients Psychosocial Factors; Aggression; Patient Abuse Complications; Wounds and Injuries Prevention and Control; **Aged**: 65+ years; **Aged**, 80 & over

42. An Illustration of SARS-CoV-2 Dissemination Within a **Skilled Nursing Facility** U[illegible]ng Heat Maps.

(includes abstract) Blackman, Carolyn; Farber, Sharon; Feifer, Richard A.; Mor, Vincent; White, Elizabeth M.; Journal of the American Geriatrics Society, Oct2020; 68(10): 2174-2178. 5p. (Article - pictorial) ISSN: 0002-8614

Subjects: COVID-19 Pandemic; Severe **Acute** Respiratory Syndrome Transmission; **Skilled Nursing Facilities**; Maps; Disease Transmission

43. Evaluating **Cognitive Impairment** and Its Relation to Function in a Population of Individuals Who Are Homeless.

(includes abstract) Synovec, Caitlin E.; Occupational Therapy in Mental Health, Oct-Dec2020; 36(4): 330-352. 23p. (Article - research, tables/charts) ISSN: 0164-212X

Subjects: Homeless Persons Psychosocial Factors; Cognition **Disorders** Therapy; Mental Health **Services**; Cognition Evaluation; Neuropsychological Tests; Functional Assessment; Occupational Therapy Assessment; Adult: 19-44 years; Middle **Aged**: 45-64 years; **Aged**: 65+ years; Male; Female

44. The Development of a Crosswalk for Functional Measures in **Postacute** Medicare Claims.

(includes abstract) McDonough, Christine M; Carmichael, Donald; Marino, Molly E; Ni, Pengsheng; Tosteson, Anna N A; Bynum, Julie P W; Physical Therapy, Oct2020; 100(10): 1862-1871. 10p. (Article - research, tables/charts) ISSN: 0031-9023

Subjects: **Subacute Care**; Medicare; Billing and Claims; Adult: 19-44 years; Middle **Aged**: 45-64 years; **Aged**: 65+ years; **Aged**, 80 & over; Male; Female

45. **Postacute Care** Utilization in Postsurgical Orthogeriatric Hip Fracture **Care**.

(includes abstract) Arshi, Armin; Iglesias, Brenda C.; Zambrana, Lester E.; Lai, Wilson C.; Zeegen, Erik N.; Sassoon, Adam A; Stavrakis, Alexandra I; Journal of the American Academy of Orthopaedic Surgeons, Sep2020; 28(18): 743-749. 7p. (journal article) ISSN: 1067-151X PMID: NLM31764201

Subjects: Fracture Fixation Statistics and Numerical Data; Postoperative **Care** Statistics and Numerical Data; Hip Fractures Surgery; Patient Attitudes; **Subacute Care** Statistics and Numerical Data; **Aged**, 80 & over; Male; Female

46. **Care Transition** Decisions After a Fall-related Emergency Department Visit: A Qualitative Study of Patients' and **Caregivers'** Experiences.

(includes abstract) Gettel, Cameron J.; Hayes, Kelsey; Shield, Renee R.; Guthrie, Kate M.; Goldberg, Elizabeth M.; Wall, Stephen P.; Academic Emergency Medicine, Sep2020; 27(9): 876-886. 11p. (Article - research, tables/charts) ISSN: 1069-6563

Subjects: Accidental Falls Psychosocial Factors; Emergency **Care**; **Transitional Care** Psychosocial Factors; Patient Attitudes; **Caregiver** Attitudes; **Aged**: 65+ years; **Aged**, 80 & over; Male; Female

47. **Transitions** to family **caregiving**: enrolling incident **caregivers** and matched non-**caregiving** controls from a population-based study.

(includes abstract) Roth, David L.; Haley, William E.; David Rhodes, J.; Sheehan, Orla C.; Huang, Jin; Blinka, Marcela D.; Yuan, Ya; Irvin, Marguerite R.; Jenny, Nancy; Durda, Peter; Cushman, Mary; Walston, Jeremy D.; Howard, Virginia J.; **Aging** Clinical & Experimental Research, Sep2020; 32(9): 1829-1838. 10p. (Article - research, tables/charts) ISSN: 1594-0667

Subjects: **Caregiver** Attitudes; Health **Transition**; Family

48. Community-Based Organizations Help with **Care** Coordination for Patients with **Dementia**: Key is a feedback loop with primary **care**, too.

(includes abstract) AHC MEDIA; Case Management Advisor, Sep2020; 31(9): 1-3. 3p. (Article) ISSN: 1053-5500

Subjects: Dementia Diagnosis; **Dementia** Therapy; Asians Psychosocial Factors; Continuity of Patient **Care**; Community Health **Services**; Primary Health **Care**; **Aged**: 65+ years

49. Permanent **transition** of homecare recipients with **dementia** to **nursing** homes in New York State: Risk factors.

(includes abstract) Young, Yuchi; Papenkov, Maksim; Hsu, Wan-Hsaing; Shahid, Faryal; Kuo, Yen-Hong; **Geriatric Nursing**, Sep2020; 41(5): 553-558. 6p. (Article - research, tables/charts) ISSN: 0197-4572

Subjects: Dementia Risk Factors; **Transitional Care**; **Home** Health **Care**; **Nursing** Homes; **Aged**: 65+ years; **Aged**, 80 & over

50. Frailty Phenotype and Healthcare Costs and Utilization in **Older** Men.

(includes abstract) Ensrud, Kristine E.; Kats, Allyson M.; Schousboe, John T.; Taylor, Brent C.; Vo, Tien N.; Cawthon, Peggy M.; Hoffman, Andrew R.; Langsetmo, Lisa; Journal of the American Geriatrics Society, Sep2020; 68(9): 2034-2042. 9p. (Article - research, tables/charts) ISSN: 0002-8614

Subjects: Frailty Syndrome In **Old** Age; Phenotype In **Old** Age; Health **Care** Costs In **Old** Age; Health Resource Utilization In **Old** Age; **Aged**: 65+ years; Male

EBSCO Connect | Privacy Policy | A/B Testing | Terms of Use | Copyright | Cookie Policy

powered by EBSCO*host*

© 2021 EBSCO Industries, Inc. All rights reserved.

▼ **Search History** (41)

View Saved

# ▲	Searches	Results	Type	Actions	Annotations
1	(Transition* adj3 (care* or unit* or bed* or program* or ward* or setting* or facilit* or service* or model* or centre* or center*)).tw,kw.	30059	Advanced	Display Results | More	
2	Transitional care/	3750	Advanced	Display Results | More	
3	(Intermediate adj3 (unit* or care or bed* or program* or ward* or setting* or facilit* or service* or model* or centre* or center*)).tw,kw.	6651	Advanced	Display Results | More	
4	Nursing home/	57991	Advanced	Display Results | More	
5	((Subacute or sub-acute) adj3 (unit* or care or bed* or program* or ward* or setting* or facilit* or service* or model* or centre* or center*)).tw,kw.	2508	Advanced	Display Results | More	
6	Subacute care/	1192	Advanced	Display Results | More	
7	((Postacute or post-acute) adj3 (unit* or care or bed* or program* or ward* or setting* or facilit* or service* or model* or centre* or center*)).tw,kw.	3616	Advanced	Display Results | More	
8	(Post acute adj3 (unit* or care or bed* or program* or ward* or setting* or facilit* or service* or model* or centre* or center*)).tw,kw.	2625	Advanced	Display Results | More	
9	(Skilled Nursing adj3 (unit* or bed* or program* or ward* or setting* or facilit* or service* or model* or centre* or center*)).tw,kw.	5482	Advanced	Display Results | More	
10	(Restor* adj3 (unit* or care or bed* or program* or ward* or setting* or facilit* or service* or model* or centre* or center*)).tw,kw.	5971	Advanced	Display Results | More	
11	(Convalesc* adj3 (unit* or care or bed* or program* or ward* or setting* or facilit* or service* or model* or centre* or center* or home* or hospital*)).tw,kw.	1691	Advanced	Display Results | More	
12	Convalescence/	56681	Advanced	Display Results | More	
13	or/1-12	163830	Advanced	Display Results | More	
14	(Old* or aged or aging).tw,kw.	3149409	Advanced	Display Results | More	
15	(Centenarian* or nonagenarian* or octogenarian* or geriatr* or gerontol* or senescen* or septuagenarian* or pensioner* or senile).tw,kw.	198542	Advanced	Display Results | More	
16	Senior*.tw,kw.	63456	Advanced	Display Results | More	
17	Elder*.tw,kw.	409385	Advanced	Display Results | More	
18	Aged/	3354014	Advanced	Display Results | More	
19	Very Elderly/	234512	Advanced	Display Results | More	
20	or/14-19	5892521	Advanced	Display Results | More	
21	(Cognit* adj3 impair*).tw,kw.	135437	Advanced	Display Results | More	
22	Mild neurocognitive disorder*.tw,kw.	348	Advanced	Display Results | More	
23	Cognitive deficit/	179332	Advanced	Display Results | More	

Contract

	#	Search	Results	Type	Actions	
☐	24	Major neurocognitive disorder*.tw,kw.	325	Advanced	Display Results | More	
☐	25	Dement*.tw,kw.	188751	Advanced	Display Results | More	
☐	26	Dementia/	127211	Advanced	Display Results | More	
☐	27	Alzheimer disease/	213733	Advanced	Display Results | More	
☐	28	Alzheimer*.tw,kw.	224568	Advanced	Display Results | More	
☐	29	Frontotemporal dementia/	14978	Advanced	Display Results | More	
☐	30	Frontal variant frontotemporal dementia/	2592	Advanced	Display Results | More	
☐	31	HIV associated dementia/	2995	Advanced	Display Results | More	
☐	32	"Mixed depression and dementia"/	139	Advanced	Display Results | More	
☐	33	Multiinfarct dementia/	12918	Advanced	Display Results | More	
☐	34	Pick presenile dementia/	1479	Advanced	Display Results | More	
☐	35	Presenile dementia/	879	Advanced	Display Results | More	
☐	36	Senile dementia/	3330	Advanced	Display Results | More	
☐	37	Diffuse Lewy body disease/	9679	Advanced	Display Results | More	
☐	38	Deliri*.tw,kw.	29387	Advanced	Display Results | More	
☐	39	Delirium/	30971	Advanced	Display Results | More	
☐	40	or/21-39	584550	Advanced	Display Results | More	
☐	41	13 and 20 and 40	9635	Advanced	Display Results | More	

Save | Remove | Combine with: AND | OR

Save All | Edit | Create RSS | View Saved | Email All Search History | Copy Search History Link | Copy Search History Details

Advanced Search | Basic Search | Find Citation | Search Tools | Search Fields | Multi-Field Search

1 Resource selected | Hide | Change

Embase Classic+Embase 1947 to 2021 July 14

Enter keyword or phrase (* or $ for truncation)

◉ **Keyword** ○ Author ○ Title ○ Journal

Search

▸ **Limits** *(expand)* ☐ Include Multimedia ☑ Map Term to Subject Heading

To search Open Access content on Ovid, go to Basic Search.

Options

Print | Email | Export | + My Projects | Keep Selected

Search Information

You searched:

13 and 20 and 40

Search terms used:

aged
aging
alzheimer
disease
alzheimer*
bed*
care
care*

☐ All | Range | Clear | 100 Per Page | 1 | Go | Next ›

☐ 1.

Abstract Reference
Complete Reference

Title **Understanding the Impact of Urinary Incontinence in Persons with Dementia: Development of an Interdisciplinary Service Model.**

Source Advances in Urology. 2021 (no pagination), 2021. Article Number: 9988056. Date of Publication: 2021.

Author Juliebo-Jones P.; Coulthard E.; Mallam E.; Archer H.; Drake M.J.

Publisher Hindawi Limited

Find Similar
Find Citing Articles

Get it UTL

centenarian*
center*
centre*
cognit*
cognitive
deficit
convalesc*
convalescence
deliri*
delirium
dement*
dementia
diffuse
lewy
body
elder*
facilit*
frontal
variant
frontotemporal
geriatr*
gerontol*
hiv
associated
home*
hospital*
impair*
intermediate
major
neurocognitive
disorder*
mild
mixed
depression
and
model*
multiinfarct
nonagenarian*
nursing
home
octogenarian*
old*
pensioner*
pick
presenile
post
acute
post-acute
postacute
program*
restor*
senescen*
senile
senior*
septuagenarian*
service*
setting*
skilled
sub-acute
subacute
transition*
transitional
unit*
very
elderly
ward*

Publication Type Article

Cite + My Projects + Annotate

2.

Title **Participation of persons with dementia and their caregivers in research.**

Source Journal of the American Geriatrics Society. 69 (7) (pp 1784-1792), 2021. Date of Publication: July 2021.

Author Frank L.; Jennings L.A.; Petersen R.C.; Majid T.; Gilmore-Bykovskyi A.; Schicker L.; Karlawish J.

Publisher Blackwell Publishing Inc.

Publication Type Review

Abstract Reference
Complete Reference
Find Similar
Find Citing Articles
Full Text

Cite + My Projects + Annotate

3.

Title **Intervention of companion clowns in a special care unit: a 1-year pilot study.**

Source Aging Clinical and Experimental Research. (no pagination), 2021. Date of Publication: 2021.

Author De Mauleon A.; Lelievre A.; Hermabessiere S.; Rolland Y.

Publisher Springer Science and Business Media Deutschland GmbH

Publication Type Article

Abstract Reference
Complete Reference
Find Similar
Find Citing Articles
Get it UTL

Cite + My Projects + Annotate

4.

Title **Optimal healthcare delivery to care homes in the UK: A realist evaluation of what supports effective working to improve healthcare outcomes.**

Source Age and Ageing. 47 (4) (pp 595-603), 2018. Date of Publication: 01 Jul 2018.

Author Gordon A.L.; Goodman C.; Davies S.L.; Dening T.; Gage H.; Meyer J.; Schneider J.; Bell B.; Jordan J.; Martin F.C.; Iliffe S.; Bowman C.; Gladman J.R.F.; Victor C.; Mayrhofer A.; Handley M.; Zubair M.

Publisher Oxford University Press

Publication Type Article

Abstract Reference
Complete Reference
Find Similar
Find Citing Articles
Get it UTL

Cite + My Projects + Annotate

5.

Abstract Reference
Complete Reference

Search Returned:
9635 text results

Sort By:

-

Customize Display

Filter By

Add to Search History

Selected Only (0)

Years

All Years

Current year

Past 3 years

Past 5 years

Specific Year Range

Subject

Author

Journal

Publication Type

My Projects

+ New Project

No projects available.

Title	**Trajectories of pro re nata (PRN) medication prescribing and administration in long-term care facilities.**
Source	Research in social & administrative pharmacy : RSAP. 17 (8) (pp 1463-1468), 2021. Date of Publication: 01 Aug 2021.
Author	Sharma M.; Wong X.Y.; Bell J.S.; Corlis M.; Hogan M.; Sluggett J.K.
Publisher	NLM (Medline)
Publication Type	Article

Find Similar
Find Citing Articles
Get it UTL

Cite + My Projects + Annotate

6.

Title	**COVID-19 in patients with dementia : Clinical features and predictive factors of mortality in a cohort of 125 patients.**
Source	European Journal of Neurology. Conference: 7th Congress of the European Academy of Neurology. Virtual. 28 (SUPPL 1) (pp 159), 2021. Date of Publication: June 2021.
Author	Agathe V.; Mhanna E.; Lebozec M.; Aveneau C.; Volpe Gillot L.; Paquet C.
Publisher	Blackwell Publishing Ltd
Publication Type	Conference Abstract

Abstract Reference
Complete Reference
Find Similar
Find Citing Articles
Full Text

Cite + My Projects + Annotate

7.

Title	**Remote reminiscence using immersive virtual reality may be efficacious for reducing anxiety in patients with mild cognitive impairment even in covid-19 pandemic: A case report.**
Source	Biological and Pharmaceutical Bulletin. 44 (7) (pp 1019-1023), 2021. Date of Publication: July 2021.
Author	Yahara M.; Niki K.; Ueno K.; Okamoto M.; Okuda T.; Tanaka H.; Naito Y.; Ishii R.; Ueda M.; Ito T.
Publisher	Pharmaceutical Society of Japan
Publication Type	Article

Abstract Reference
Complete Reference
Find Similar
Find Citing Articles
Full Text

Cite + My Projects + Annotate

8.

Title	**Toward the development of a vibrant, super-aged society: The future of medicine and society in Japan.**
Source	Geriatrics and Gerontology International. (no pagination), 2021. Date of Publication: 2021.
Author	Iijima K.; Arai H.; Akishita M.; Endo T.; Ogasawara K.; Kashihara N.; Hayashi Y.K.; Yumura W.; Yokode M.; Ouchi Y.
Publisher	Blackwell Publishing
Publication	Article

Abstract Reference
Complete Reference
Find Similar
Find Citing Articles
Full Text

Type

Cite + My Projects + Annotate

9.

Abstract Reference
Complete Reference

**Title**	**A third of dying patients do not have end-of-life discussions with a physician: A nationwide registry study.**
**Source**	Palliative & supportive care. (pp 1-6), 2021. Date of Publication: 23 Jun 2021.
**Author**	Melin-Johansson C.; Sveen J.; Lovgren M.; Udo C.
**Publisher**	NLM (Medline)
**Publication Type**	Article

Find Similar
Find Citing Articles

Get it UTL

Cite + My Projects + Annotate

10.

Abstract Reference
Complete Reference

**Title**	**The nutritional status of the elderly patient infected with COVID-19: the forgotten risk factor?.**
**Source**	Current Medical Research and Opinion. 37 (4) (pp 549-554), 2021. Date of Publication: 2021.
**Author**	Abadia Otero J.; Briongos Figuero L.S.; Gabella Mattin M.; Usategui Martin I.; Cubero Morais P.; Cuellar Olmedo L.; Inglada Galiana L.; Duenas Gutierrez C.; Carretero Gomez J.; Corral Gudino L.; Miramontes Gonzalez J.P.
**Publisher**	Taylor and Francis Ltd.
**Publication Type**	Article

Find Similar
Find Citing Articles

Full Text

Cite + My Projects + Annotate

11.

Abstract Reference
Complete Reference

**Title**	**Immersive Virtual Reality Reminiscence Reduces Anxiety in the Oldest-Old Without Causing Serious Side Effects: A Single-Center, Pilot, and Randomized Crossover Study.**
**Source**	Frontiers in Human Neuroscience. 14 (no pagination), 2020. Article Number: 598161. Date of Publication: 18 Jan 2021.
**Author**	Niki K.; Yahara M.; Inagaki M.; Takahashi N.; Watanabe A.; Okuda T.; Ueda M.; Iwai D.; Sato K.; Ito T.
**Publisher**	Frontiers Media S.A.
**Publication Type**	Article

Find Similar
Find Citing Articles

Get it UTL

Cite + My Projects + Annotate

12.

Abstract Reference
Complete Reference

Title	**Italian version of the short 10/66 dementia diagnostic schedule: a validation study.**	Find Similar<br>Find Citing Articles<br><br>Full Text
Source	BMJ Open. 11 (6) (no pagination), 2021. Article Number: 045867. Date of Publication: 30 Jun 2021.	
Author	Ibnidris A.; Piumatti G.; Carlevaro F.; Fadda M.; Magno F.; Magistro D.; Albanese E.	
Publisher	BMJ Publishing Group	
Publication Type	Article	

Cite + My Projects + Annotate

13.

		Abstract Reference<br>Complete Reference
Title	**Predictive factors of pulmonary embolism in older patients with sars-cov-2: The octa-covid-19 study.**	
Source	Journal of Clinical Medicine. 10 (13) (no pagination), 2021. Article Number: 2998. Date of Publication: 01 Jul 2021.	Find Similar<br>Find Citing Articles
Author	Quezada-Feijoo M.; Ramos M.; Lozano-Montoya I.; Toro R.; Jaramillo-Hidalgo J.; Fernandez de la Puente E.; Garmendia B.; Carrillo P.; Cristofori G.; Goni Roson S.; Ayala R.; Sarro M.; Gomez-Pavon F.J.	Full Text
Publisher	MDPI AG	
Publication Type	Article	

Cite + My Projects + Annotate

14.

		Abstract Reference<br>Complete Reference
Title	**An integrated multidisciplinary model of COVID-19 recovery care.**	
Source	Irish Journal of Medical Science. 190 (2) (pp 461-468), 2021. Date of Publication: May 2021.	Find Similar<br>Find Citing Articles
Author	O'Brien H.; Tracey M.J.; Ottewill C.; O'Brien M.E.; Morgan R.K.; Costello R.W.; Gunaratnam C.; Ryan D.; McElvaney N.G.; McConkey S.J.; McNally C.; Curley G.F.; MacHale S.; Gillan D.; Pender N.; Barry H.; de Barra E.; Kiernan F.M.; Sulaiman I.; Hurley K.	Get it UTL
Publisher	Springer Science and Business Media Deutschland GmbH	
Publication Type	Article	

Cite + My Projects + Annotate

15.

		Abstract Reference<br>Complete Reference
Title	**Assessing the impact of COVID-19 on the health of geriatric patients: The European GeroCovid Observational Study.**	
Source	European Journal of Internal Medicine. 87 (pp 29-35), 2021. Date of Publication: May 2021.	Find Similar<br>Find Citing Articles
Author	Trevisan C.; Del Signore S.; Fumagalli S.; Gareri P.; Malara A.; Mossello E.; Volpato S.; Monzani F.; Coin A.; Bellelli G.; Zia G.;	Get it UTL

Ranhoff A.H.; Antonelli Incalzi R.

Publisher Elsevier B.V.

Publication Type Article

Cite + My Projects + Annotate

16.

Abstract Reference
Complete Reference

Title **Influence of cognitive function and nurse support on malnutrition risk in nursing home residents.**

Source Nursing open. 8 (4) (pp 1805-1811), 2021. Date of Publication: 01 Jul 2021.

Author Pakai A.; Havasi-Santha E.; Mak E.; Mate O.; Pusztai D.; Fuller N.; Zrinyi M.; Olah A.

Publisher NLM (Medline)

Publication Type Article

Find Similar
Find Citing Articles

Full Text

Cite + My Projects + Annotate

17.

Abstract Reference
Complete Reference

Title **Oral health matters in cognitive impaired aged residents in geriatric care facilities: A cross-sectional survey.**

Source Nursing open. 8 (2) (pp 792-798), 2021. Date of Publication: 01 Mar 2021.

Author Chen L.; Gu L.; Li X.; Chen W.; Zhang L.

Publisher NLM (Medline)

Publication Type Article

Find Similar
Find Citing Articles

Full Text

Cite + My Projects + Annotate

18.

Abstract Reference
Complete Reference

Title **Change in Social Engagement among Incident Caregivers and Controls: Findings from the Caregiving Transitions Study.**

Source Journal of aging and health. 33 (1-2) (pp 114-124), 2021. Date of Publication: 01 Jan 2021.

Author Liu C.; Fabius C.D.; Howard V.J.; Haley W.E.; Roth D.L.

Publisher NLM (Medline)

Publication Type Article

Find Similar
Find Citing Articles

Get it UTL

Cite + My Projects + Annotate

19.

Abstract Reference
Complete Reference

**Title**	**Transitioning to Long-Term Care: Family Caregiver Experiences of Dementia, Communities, and Counseling.**	Find Similar<br>Find Citing Articles<br>Get it UTL
**Source**	Journal of aging and health. 33 (1-2) (pp 133-146), 2021. Date of Publication: 01 Jan 2021.	
**Author**	Zmora R.; Statz T.L.; Birkeland R.W.; McCarron H.R.; Finlay J.M.; Rosebush C.E.; Gaugler J.E.	
**Publisher**	NLM (Medline)	
**Publication Type**	Article	

Cite + My Projects + Annotate

☐ 20.

Abstract Reference
Complete Reference

**Title**	**Mechanical Restraint in Nursing Homes in Brazil: a cross-sectional study.**	Find Similar<br>Find Citing Articles<br>Get it UTL
**Source**	Revista brasileira de enfermagem. 73 3 (Supplement) (pp e20190509), 2020. Date of Publication: 2020.	
**Author**	Delvalle R.; Santana R.F.; Menezes A.K.; Cassiano K.M.; Carvalho A.C.S.; Barros P.F.A.	
**Publisher**	NLM (Medline)	
**Publication Type**	Article	

Cite + My Projects + Annotate

☐ 21.

Abstract Reference
Complete Reference

**Title**	**Psychotropic drug use in Sydney nursing homes.**	
**Source**	Medical Journal of Australia. 163 (2) (pp 70-72), 1995. Date of Publication: 1995.	Find Similar<br>Find Citing Articles
**Author**	Snowdon J.; Vaughan R.; Miller R.; Burgess E.E.; Tremlett P.	
**Publisher**	Australasian Medical Publishing Co. Ltd	Full Text
**Publication Type**	Article	

Cite + My Projects + Annotate

☐ 22.

Complete Reference

**Title**	**Dementia research in the nursing home.**	
**Source**	Hospital and Community Psychiatry. 39 (3) (pp 257-259), 1988. Date of Publication: 1988.	Find Similar<br>Find Citing Articles
**Author**	Abrams R.	Get it UTL
**Publication Type**	Article	

Cite + My Projects + Annotate

23.

Title	**Hydration interventions for older people living in residential and nursing care homes: Overview of the literature.**	Abstract Reference<br>Complete Reference<br>Find Similar<br>Find Citing Articles<br>Full Text
Source	British Medical Bulletin. 131 (1) (pp 71-79), 2019. Date of Publication: 19 Sep 2019.	
Author	Cook G.; Hodgson P.; Thompson J.; Bainbridge L.; Johnson A.; Storey P.	
Publisher	Oxford University Press	
Publication Type	Review	

Cite + My Projects + Annotate

24.

Title	**Chronology of covid-19 symptoms in very old patients: Study of a hospital outbreak.**	Ovid Full Text<br>Abstract Reference<br>Complete Reference<br>Find Similar<br>Find Citing Articles<br>Full Text
Source	Journal of Clinical Medicine. 10 (13) (no pagination), 2021. Article Number: 2962. Date of Publication: 01 Jul 2021.	
Author	Lafuente-Lafuente C.; Nghiem Q.D.; Keravec H.; Oukbir-Ferrag S.; Magri M.; Oquendo B.; Donadio C.; Rainone A.; Belmin J.	
Publisher	MDPI AG	
Publication Type	Article	

Article as PDF (591KB) Cite + My Projects + Annotate

25.

Title	**Functioning and cognition of portuguese older adults attending in residential homes and day centers: A comparative study.**	Abstract Reference<br>Complete Reference<br>Find Similar<br>Find Citing Articles<br>Full Text
Source	International Journal of Environmental Research and Public Health. 18 (13) (no pagination), 2021. Article Number: 7030. Date of Publication: 01 Jul 2021.	
Author	Lopes M.J.; de Pinho L.G.; Fonseca C.; Goes M.; Oliveira H.; Garcia-Alonso J.; Afonso A.	
Publisher	MDPI AG	
Publication Type	Article	

Cite + My Projects + Annotate

26.

Title	**Caffeine Consumption and Behavioral Symptoms in Nursing Home Residents: A Cross-Sectional Analysis.**	Abstract Reference<br>Complete Reference<br>Find Similar<br>Find Citing Articles<br>Get it UTL
Source	Journal of Nutrition, Health and Aging. 25 (1) (pp 100-107), 2021. Date of Publication: January 2021.	
Author	Kromhout (Michelle) M.A.; Rius Ottenheim N.; Putter H.; Numans M.E.; Achterberg W.P.	

Publisher Serdi-Editions

Publication Type Article

Cite + My Projects + Annotate

27.

Abstract Reference
Complete Reference

Title **Texture-Modified Diet for Improving the Management of Oropharyngeal Dysphagia in Nursing Home Residents: An Expert Review.**

Source Journal of Nutrition, Health and Aging. 24 (6) (pp 576-581), 2020. Date of Publication: 01 Jun 2020.

Author Ballesteros-Pomar M.D.; Cherubini A.; Keller H.; Lam P.; Rolland Y.; Simmons S.F.

Publisher Serdi-Editions

Publication Type Article

Find Similar
Find Citing Articles
Get it UTL

Cite + My Projects + Annotate

28.

Abstract Reference
Complete Reference

Title **The Prevalence of Frailty by the FRAIL-NH Scale in Taiwan Nursing Home Residents.**

Source Journal of Nutrition, Health and Aging. 24 (5) (pp 507-511), 2020. Date of Publication: 01 May 2020.

Author Peng T.-C.; Chen W.-L.; Wu L.-W.; Chang Y.-W.; Kao T.-W.

Publisher Serdi-Editions

Publication Type Article

Find Similar
Find Citing Articles
Get it UTL

Cite + My Projects + Annotate

29.

Abstract Reference
Complete Reference

Title **Is the Effect of a High-Intensity Functional Exercise Program on Functional Balance Influenced by Applicability and Motivation among Older People with Dementia in Nursing Homes?.**

Source Journal of Nutrition, Health and Aging. 23 (10) (pp 1011-1020), 2019. Date of Publication: 01 Dec 2019.

Author Sondell A.; Littbrand H.; Holmberg H.; Lindelof N.; Rosendahl E.

Publisher Serdi-Editions

Publication Type Article

Find Similar
Find Citing Articles
Get it UTL

Cite + My Projects + Annotate

30.

**Title**	**The Effect of Exercise and Social Activity Interventions on Nutritional Status in Older Adults with Dementia Living in Nursing Homes: A Randomised Controlled Trial.**	Abstract Reference<br>Complete Reference<br>Find Similar<br>Find Citing Articles<br>Get it UTL
**Source**	Journal of Nutrition, Health and Aging. 22 (7) (pp 824-828), 2018. Date of Publication: 01 Jul 2018.	
**Author**	Maltais M.; Rolland Y.; Hay P.-E.; Armaingaud D.; Cestac P.; Rouch L.; de Souto Barreto P.	
**Publisher**	Springer-Verlag France	
**Publication Type**	Article	

Cite + My Projects + Annotate

31.

**Title**	**Status of Geriatrics in 22 Countries.**	Abstract Reference<br>Complete Reference<br>Find Similar<br>Find Citing Articles
**Source**	Journal of Nutrition, Health and Aging. 22 (5) (pp 627-631), 2018. Date of Publication: 01 May 2018.	
**Author**	Pitkala K.H.; Martin F.C.; Maggi S.; Jyvakorpi S.K.; Strandberg T.E.	
**Publisher**	Springer-Verlag France	
**Publication Type**	Article	

Cite + My Projects + Annotate

32.

**Title**	**Diagnosis of Sarcopenia in Long-Term Care Homes for the Elderly: The Sensitivity and Specificity of Two Simplified Algorithms with Respect to the EWGSOP Consensus.**	Abstract Reference<br>Complete Reference<br>Find Similar<br>Find Citing Articles<br>Get it UTL
**Source**	Journal of Nutrition, Health and Aging. 22 (7) (pp 796-801), 2018. Date of Publication: 01 Jul 2018.	
**Author**	Rodriguez-Rejon A.I.; Artacho R.; Puerta A.; Zuniga A.; Ruiz-Lopez M.D.	
**Publisher**	Springer-Verlag France	
**Publication Type**	Article	

Cite + My Projects + Annotate

33.

**Title**	**Frailty related factors as predictors of functional recovery in geriatric rehabilitation: The sarcopenia and function in aging rehabilitation (safari) multi-centric study.**	Abstract Reference<br>Complete Reference<br>Find Similar<br>Find Citing Articles<br>Get it UTL
**Source**	Journal of Nutrition, Health and Aging. 22 (9) (pp 1099-1106), 2018. Date of Publication: November 2018.	
**Author**	Calle A.; Onder G.; Morandi A.; Bellelli G.; Ortolani E.; Perez L.M.; Mesas M.; Sanniti A.; Mazzanti P.; Platto C.N.; Gentile S.; Martinez N.; Roque M.; Inzitari M.	
**Publisher**	Springer-Verlag France	

**Publication Type**	Article

Cite + My Projects + Annotate

34.

Abstract Reference
Complete Reference

Find Similar
Find Citing Articles

Get it UTL

**Title**	A new adaptive home-based exercise technology among older adults living in nursing home: A pilot study on feasibility, acceptability and physical performance.
**Source**	Journal of Nutrition, Health and Aging. 21 (7) (pp 819-824), 2017. Date of Publication: 01 Jul 2017.
**Author**	Valiani V.; Lauze M.; Martel D.; Pahor M.; Manini T.M.; Anton S.; Aubertin-Leheudre M.
**Publisher**	Springer-Verlag France
**Publication Type**	Article

Cite + My Projects + Annotate

35.

Abstract Reference
Complete Reference

Find Similar
Find Citing Articles

Get it UTL

**Title**	Person-centered feeding care: A protocol to re-introduce oral feeding for nursing home patients with tube feeding.
**Source**	Journal of Nutrition, Health and Aging. 20 (6) (pp 621-627), 2016. Date of Publication: 01 Jun 2016.
**Author**	Bell C.L.; Lopez R.P.; Mahendra N.; Tamai A.; Davis J.; Amella E.J.; Masaki K.
**Publisher**	Springer-Verlag France
**Publication Type**	Article

Cite + My Projects + Annotate

36.

Abstract Reference
Complete Reference

Find Similar
Find Citing Articles

Get it UTL

**Title**	Relearning of activities of daily living: A comparison of the effectiveness of three learning methods in patients with dementia of the Alzheimer type.
**Source**	Journal of Nutrition, Health and Aging. 20 (1) (pp 48-55), 2016. Date of Publication: 01 Jan 2016.
**Author**	Bourgeois J.; Laye M.; Lemaire J.; Leone E.; Deudon A.; Darmon N.; Giaume C.; Lafont V.; Brinck-Jensen S.; Dechamps A.; Konig A.; Robert P.
**Publisher**	Springer-Verlag France
**Publication Type**	Article

Cite + My Projects + Annotate

37.

Abstract Reference
Complete Reference

Title	**Association of grip strength, upper arm circumference, and waist circumference with dementia in older adults of the wise study: A cross-sectional analysis.**
Source	Journal of Nutrition, Health and Aging. 20 (10) (pp 996-1001), 2016. Date of Publication: 01 Dec 2016.
Author	Ong H.L.; Chang S.H.S.; Abdin E.; Vaingankar J.A.; Jeyagurunathan A.; Shafie S.; Magadi H.; Chong S.A.; Subramaniam M.
Publisher	Springer-Verlag France
Publication Type	Article

Find Similar
Find Citing Articles
Get it UTL

Cite + My Projects + Annotate

38.

Abstract Reference
Complete Reference

Title	**Three different outcomes in older community-dwelling patients receiving intermediate care in nursing home after acute hospitalization.**
Source	Journal of Nutrition, Health and Aging. 20 (4) (pp 446-452), 2016. Date of Publication: 01 Apr 2016.
Author	Abrahamsen J.F.; Haugland C.; Nilsen R.M.; Ranhoff A.H.
Publisher	Springer-Verlag France
Publication Type	Article

Find Similar
Find Citing Articles
Get it UTL

Cite + My Projects + Annotate

39.

Abstract Reference
Complete Reference

Title	**Defining rehabilitation success in older adults with dementia-results from an inpatient geriatric rehabilitation unit.**
Source	Journal of Nutrition, Health and Aging. 20 (4) (pp 439-445), 2016. Date of Publication: 01 Apr 2016.
Author	Muir-Hunter S.W.; Lim Fat G.; Mackenzie R.; Wells J.; Montero-Odasso M.
Publisher	Springer-Verlag France
Publication Type	Article

Find Similar
Find Citing Articles
Get it UTL

Cite + My Projects + Annotate

40.

Abstract Reference
Complete Reference

Title	**Compliance of nursing home residents with a nutrient- and energy-dense oral nutritional supplement determines effects on nutritional status.**
Source	Journal of Nutrition, Health and Aging. 19 (3) (pp 356-364), 2015. Date of Publication: 11 Mar 2015.
Author	Jobse I.; Liao Y.; Bartram M.; Delantonio K.; Uter W.; Stehle P.; Sieber C.C.; Volkert D.

Find Similar
Find Citing Articles
Get it UTL

**Publisher**	Springer-Verlag France
**Publication Type**	Article

Cite + My Projects + Annotate

41.

**Title**	**Nutrition and psychological well-being among long-term care residents with dementia.**
**Source**	Journal of Nutrition, Health and Aging. 19 (2) (pp 178-182), 2015. Date of Publication: 22 Feb 2015.
**Author**	Muurinen S.; Savikko N.; Soini H.; Suominen M.; Pitkala K.
**Publisher**	Springer-Verlag France
**Publication Type**	Article

Abstract Reference
Complete Reference
Find Similar
Find Citing Articles
Get it UTL

Cite + My Projects + Annotate

42.

**Title**	**Strategies to implement community guidelines on nutrition and their long-term clinical effects in nursing home residents.**
**Source**	Journal of Nutrition, Health and Aging. 19 (1) (pp 70-76), 2015. Date of Publication: January 2014.
**Author**	Torma J.; Winblad U.; Saletti A.; Cederholm T.
**Publisher**	Springer-Verlag France
**Publication Type**	Article

Abstract Reference
Complete Reference
Find Similar
Find Citing Articles
Get it UTL

Cite + My Projects + Annotate

43.

**Title**	**The Rapid Cognitive Screen (RCS): A point-of-care screening for dementia and mild cognitive impairment.**
**Source**	Journal of Nutrition, Health and Aging. 19 (7) (pp 741-744), 2015. Date of Publication: 28 Aug 2015.
**Author**	Malmstrom T.K.; Voss V.B.; Cruz-Oliver D.M.; Cummings-Vaughn L.A.; Tumosa N.; Grossberg G.T.; Morley J.E.
**Publisher**	Springer-Verlag France
**Publication Type**	Article

Abstract Reference
Complete Reference
Find Similar
Find Citing Articles
Get it UTL

Cite + My Projects + Annotate

44.

**Title**	**Wrist actigraphy: A simple way to record motor activity in elderly patients with dementia and apathy or aberrant motor**

Abstract Reference
Complete Reference

behavior.

Source: Journal of Nutrition, Health and Aging. 19 (7) (pp 759-764), 2015. Date of Publication: 28 Aug 2015.

Author: Valembois L.; Oasi C.; Pariel S.; Jarzebowski W.; Lafuente-Lafuente C.; Belmin J.

Publisher: Springer-Verlag France

Publication Type: Article

Find Similar
Find Citing Articles
Get it UTL

Cite + My Projects + Annotate

45.

Abstract Reference
Complete Reference

Title: **Multimorbidity type, hospitalizations and emergency department visits among nursing home residents: A preliminary study.**

Source: Journal of Nutrition, Health and Aging. 18 (7) (pp 705-709), 2014. Date of Publication: 28 Sep 2014.

Author: de Souto Barreto P.; Lapeyre-Mestre M.; Vellas B.; Rolland Y.

Publisher: Springer-Verlag France

Publication Type: Article

Find Similar
Find Citing Articles
Get it UTL

Cite + My Projects + Annotate

46.

Abstract Reference
Complete Reference

Title: **Patterns of chronic co-morbid medical conditions in older residents of U.S. nursing homes: Differences between the sexes and across the agespan.**

Source: Journal of Nutrition, Health and Aging. 18 (4) (pp 429-436), 2014. Date of Publication: April 2014.

Author: Moore K.L.; Boscardin W.J.; Steinman M.A.; Schwartz J.B.

Publisher: Springer-Verlag France

Publication Type: Article

Find Similar
Find Citing Articles
Get it UTL

Cite + My Projects + Annotate

47.

Abstract Reference
Complete Reference

Title: **Relation between caffeine and behavioral symptoms in elderly patients with dementia: An observational study.**

Source: Journal of Nutrition, Health and Aging. 18 (4) (pp 407-410), 2014. Date of Publication: April 2014.

Author: Kromhout M.A.; Jongerling J.; Achterberg W.P.

Publisher: Springer-Verlag France

Publication Type: Article

Find Similar
Find Citing Articles
Get it UTL

Cite + My Projects + Annotate

48.

Abstract Reference
Complete Reference

Find Similar
Find Citing Articles

Get it UTL

**Title**	**A multicentric individually-tailored controlled trial of education and professional support to nursing home staff: Research protocol and baseline data of the IQUARE study.**
**Source**	Journal of Nutrition, Health and Aging. 17 (2) (pp 173-178), 2013. Date of Publication: February 2013.
**Author**	De Souto Barreto P.; Lapeyre-Mestre M.; Mathieu C.; Piau C.; Bouget C.; Cayla F.; Vellas B.; Rolland Y.
**Publisher**	Springer-Verlag France
**Publication Type**	Article

Cite + My Projects + Annotate

49.

Abstract Reference
Complete Reference

Find Similar
Find Citing Articles

Get it UTL

**Title**	**The influence of nutritional supplement drinks on providing adequate calorie and protein intake in older adults with dementia.**
**Source**	Journal of Nutrition, Health and Aging. 17 (9) (pp 752-755), 2013. Date of Publication: November 2013.
**Author**	Allen V.; Methven L.; Gosney M.
**Publisher**	Springer-Verlag France
**Publication Type**	Article

Cite + My Projects + Annotate

50.

Abstract Reference
Complete Reference

Find Similar
Find Citing Articles

Get it UTL

**Title**	**Nutritional screening strategy in nonagenarians: The value of the MNA-SF (Mini Nutritional Assessment short form) in NutriAction.**
**Source**	Journal of Nutrition, Health and Aging. 17 (4) (pp 310-314), 2013. Date of Publication: April 2013.
**Author**	Vandewoude M.; Van Gossum A.
**Publisher**	Springer-Verlag France
**Publication Type**	Article

Cite + My Projects + Annotate

51.

Complete Reference

Find Similar
Find Citing Articles

**Title**	**The nursing home population: An opportunity to make advances on research on multimorbidity and polypharmacy.**
**Source**	Journal of Nutrition, Health and Aging. 17 (4) (pp 399-400), 2013. Date of Publication: April 2013.

**Author**	De Souto Barreto P.; Vellas B.; Morley J.E.; Rolland Y.	Get it UTL
**Publisher**	Springer-Verlag France	
**Publication Type**	Editorial	

Cite + My Projects + Annotate

52.

**Title**	**Screening for malnutrition in nursing home residents: Comparison of different risk markers and their association to functional impairment.**	Abstract Reference<br>Complete Reference<br>Find Similar<br>Find Citing Articles
**Source**	Journal of Nutrition, Health and Aging. 17 (4) (pp 357-363), 2013.<br>Date of Publication: April 2013.	
**Author**	Stange I.; Poeschl K.; Stehle P.; Sieber C.C.; Volkert D.	Get it UTL
**Publisher**	Springer-Verlag France	
**Publication Type**	Article	

Cite + My Projects + Annotate

53.

**Title**	**Institutional factors associated with the nutritional status of residents from 10 german nursing homes (ernstes study).**	Abstract Reference<br>Complete Reference<br>Find Similar<br>Find Citing Articles
**Source**	Journal of Nutrition, Health and Aging. 17 (3) (pp 271-276), 2013.<br>Date of Publication: March 2013.	
**Author**	Strathmann S.; Lesser S.; Bai-Habelski J.; Overzier S.; Paker-Eichelkraut H.S.; Stehle P.; Heseker H.	Get it UTL
**Publisher**	Springer-Verlag France	
**Publication Type**	Article	

Cite + My Projects + Annotate

54.

**Title**	**Multidisciplinary team meetings (MDTM) in detection of Alzheimer's disease: Data from the idem study.**	Abstract Reference<br>Complete Reference<br>Find Similar<br>Find Citing Articles
**Source**	Journal of Nutrition, Health and Aging. 17 (2) (pp 137-141), 2013.<br>Date of Publication: February 2013.	
**Author**	Rolland Y.; Tavassoli N.; Gillette-Guyonnet S.; Perrin A.; Hermabessiere S.; Ousset P.-J.; Nourhashemi F.; Cestac P.; Vellas B.	Get it UTL
**Publisher**	Springer-Verlag France	
**Publication Type**	Article	

Cite + My Projects + Annotate

55.

Title [Fewer referrals to Swedish emergency departments among nursing home patients with dementia, comprehensive cognitive decline and multicomorbidity.]

Source Journal of Nutrition, Health and Aging. 16 (10) (pp 891-897), 2012. Date of Publication: October 2012.

Author Mamhidir A.-G.; Wimo A.; Kihlgren A.

Publisher Springer-Verlag France

Publication Type Article

Abstract Reference
Complete Reference
Find Similar
Find Citing Articles
Get it UTL

Cite + My Projects + Annotate

56.

Title Water homeostasis, frailty and cognitive function in the nursing home.

Source Journal of Nutrition, Health and Aging. 16 (1) (pp 35-39), 2012. Date of Publication: January 2012.

Author Kehayias J.J.; Ribeiro S.M.L.; Skahan A.; Itzkowitz L.; Dallal G.; Rogers G.; Khodeir M.

Publisher Springer-Verlag France

Publication Type Article

Abstract Reference
Complete Reference
Find Similar
Find Citing Articles
Get it UTL

Cite + My Projects + Annotate

57.

Title The oldest old: Red blood cell and plasma folate in African American and white octogenarians and centenarians in Georgia.

Source Journal of Nutrition, Health and Aging. 15 (9) (pp 744-750), 2011. Date of Publication: November 2011.

Author Hausman D.B.; Johnson M.A.; Davey A.; Woodard J.L.; Poon L.W.; Allen R.H.; Stabler S.P.

Publisher Springer-Verlag France

Publication Type Article

Abstract Reference
Complete Reference
Find Similar
Find Citing Articles
Get it UTL

Cite + My Projects + Annotate

58.

Title Clinical practice in nursing homes as a key for progress.

Source Journal of Nutrition, Health and Aging. 14 (7) (pp 586-593), 2010. Date of Publication: July 2010.

Author Morley J.E.

Publisher Springer-Verlag France

Publication Review

Abstract Reference
Complete Reference
Find Similar
Find Citing Articles
Get it UTL

Type

Cite + My Projects + Annotate

59.

Abstract Reference
Complete Reference

Title	**Health care for older people in Italy: The U.L.I.S.S.E. project (Un link informatico sui servizi sanitari esistenti per l'anziano - A computerized network on health care services for older people).**
Source	Journal of Nutrition, Health and Aging. 14 (3) (pp 238-242), 2010. Date of Publication: March 2010.
Author	Lattanzio F.; Mussi C.; Scafato E.; Ruggiero C.; Dell'aquila G.; Pedone C.; Mammarella F.; Galluzzo L.; Salvioli G.; Senin U.; Carbonin P.U.; Bernabei R.; Cherubini A.
Publisher	Springer-Verlag France
Publication Type	Article

Find Similar
Find Citing Articles

Get it UTL

Cite + My Projects + Annotate

60.

Abstract Reference
Complete Reference

Title	**Sleep disturbances in nursing homes.**
Source	Journal of Nutrition, Health and Aging. 14 (3) (pp 207-211), 2010. Date of Publication: March 2010.
Author	Neikrug A.B.; Ancoli-Israel S.
Publisher	Springer-Verlag France
Publication Type	Review

Find Similar
Find Citing Articles

Get it UTL

Cite + My Projects + Annotate

61.

Abstract Reference
Complete Reference

Title	**Costs of dementia in Hungary.**
Source	Journal of Nutrition, Health and Aging. 14 (8) (pp 633-639), 2010. Date of Publication: August 2010.
Author	Ersek K.; Kovacs T.; Wimo A.; Karpati K.; Brodszky V.; Pentek M.; Jonsson L.; Gustavsson A.; McDaid D.; Kenigsberg P.A.; Valtonen H.; Gulacsi L.
Publisher	Springer-Verlag France
Publication Type	Article

Find Similar
Find Citing Articles

Get it UTL

Cite + My Projects + Annotate

62.

Abstract Reference
Complete Reference

Title **Comparison of two different approaches for the application of**

the mini nutritional assessment in nursing homes: Resident interviews versus assessment by nursing staff.

Find Similar
Find Citing Articles

Get it UTL

Source Journal of Nutrition, Health and Aging. 13 (10) (pp 863-869), 2009. Date of Publication: 2009.

Author Kaiser R.; Winning K.; Uter W.; Lesser S.; Stehle P.; Sieber C.C.; Bauer J.M.

Publisher Springer-Verlag France

Publication Type Article

Cite + My Projects + Annotate

63.

Abstract Reference
Complete Reference

Title Who will become malnourished? A prospective study of factors associated with malnutrition in older persons living at home.

Find Similar
Find Citing Articles

Source Journal of Nutrition, Health and Aging. 13 (10) (pp 855-861), 2009. Date of Publication: 2009.

Author Johansson L.; Sidenvall B.; Malmberg B.; Christensson L.

Get it UTL

Publisher Springer-Verlag France

Publication Type Article

Cite + My Projects + Annotate

64.

Abstract Reference
Complete Reference

Title Balneotherapy, prevention of cognitive decline and care the Alzheimer patient and his family: Outcome of a multidisciplinary workgroup.

Find Similar
Find Citing Articles

Source Journal of Nutrition, Health and Aging. 13 (9) (pp 797-806), 2009. Date of Publication: 2009.

Author Secher M.; Soto M.; Gillette S.; Andrieu S.; Villars H.; Vellas B.; Tabone C.; Chareyras J.-B.; Dubois O.; Roques C.-F.; Dubois B.

Get it UTL

Publisher Springer-Verlag France

Publication Type Review

Cite + My Projects + Annotate

65.

Complete Reference

Title Gerontology and Geriatrics, a worldwide expansion in 2009: Data from the IAGG world meeting Paris 2009, July 5-9th.

Find Similar
Find Citing Articles

Source Journal of Nutrition, Health and Aging. 13 (4) (pp 292), 2009. Date of Publication: April 2009.

Author Vellas B.; Forette B.

Get it UTL

Publisher Springer-Verlag Italia s.r.l.

Publication Type Editorial

Cite + My Projects + Annotate

66.

Abstract Reference
Complete Reference

Title **Methodological issues in the non pharmacological treatment of BPSD in nursing home - The TNM study.**

Source Journal of Nutrition, Health and Aging. 13 (3) (pp 260-263), 2009. Date of Publication: March 2009.

Author Leone E.; Deudon A.; Maubourguet N.; Gervais X.; Robert P.H.

Publisher Springer-Verlag France

Publication Type Article

Find Similar
Find Citing Articles

Get it UTL

Cite + My Projects + Annotate

67.

Complete Reference

Title **Nutrition and aging. The carla workshop.**

Source Journal of Nutrition, Health and Aging. 12 (6) (pp 335-364), 2008. Date of Publication: June 2008.

Author Van Kan G.A.; Gambassi G.; De Groot L.C.P.G.M.; Andrieu S.; Cederholm T.; Andre E.; Caubere J.-P.; Bonjour J.-P.; Ritz P.; Salva A.; Sinclair A.; Vellas B.; Dayde J.; Deregnaucourt J.; Latge C.; Salva T.

Publisher Springer-Verlag Italia s.r.l.

Publication Type Conference Paper

Find Similar
Find Citing Articles

Get it UTL

Cite + My Projects + Annotate

68.

Abstract Reference
Complete Reference

Title **Dementia severity, decline and improvement after a lower respiratory tract infection.**

Source Journal of Nutrition, Health and Aging. 11 (6) (pp 502-506), 2007. Date of Publication: November/December 2007.

Author Van Der Steen J.T.; Kruse R.L.; Mehr D.R.; Ribbe M.W.; Van Der Wal G.

Publisher Springer-Verlag Italia s.r.l.

Publication Type Article

Find Similar
Find Citing Articles

Get it UTL

Cite + My Projects + Annotate

69.

Abstract Reference
Complete Reference

Title **Silent and invisible; nursing home residents with advanced dementia.**

Source Journal of Nutrition, Health and Aging. 11 (6) (pp 484-488), 2007.

Find Similar
Find Citing Articles

Date of Publication: November/December 2007.

Author Simard J.

Publisher Springer-Verlag Italia s.r.l.

Publication Type Review

Get it UTL

Cite + My Projects + Annotate

70.

Title **The Mini Nutritional Assessment (MNA) review of the literature - What does it tell us?.**

Source Journal of Nutrition, Health and Aging. 10 (6) (pp 466-485), 2006. Date of Publication: November/December 2006.

Author Guigoz Y.

Publisher Springer-Verlag Italia s.r.l.

Publication Type Review

Abstract Reference
Complete Reference

Find Similar
Find Citing Articles

Get it UTL

Cite + My Projects + Annotate

71.

Title **Geriatric future history.**

Source Journal of Nutrition, Health and Aging. 10 (5) (pp 431), 2006. Date of Publication: September/October 2006.

Author Morley J.E.

Publisher Springer-Verlag France

Publication Type Note

Complete Reference

Find Similar
Find Citing Articles

Get it UTL

Cite + My Projects + Annotate

72.

Title **Survival after percutaneous endoscopic gastrostomy: The role of dementia.**

Source Journal of Nutrition, Health and Aging. 9 (4) (pp 255-259), 2005. Date of Publication: 2005.

Author Shah P.M.; Sen S.; Perlmuter L.C.; Feller A.

Publisher Springer-Verlag Italia s.r.l.

Publication Type Review

Abstract Reference
Complete Reference

Find Similar
Find Citing Articles

Get it UTL

Cite + My Projects + Annotate

73.

Title **Nutrient content of served food, nutrient intake and nutritional**

Abstract Reference
Complete Reference

status of residents with dementia in a finnish nursing home.

Source	Journal of Nutrition, Health and Aging. 8 (4) (pp 234-238), 2004. Date of Publication: 2004.	Find Similar<br>Find Citing Articles
Author	Suominen M.; Laine A.; Routasalo P.; Pitkala K.H.; Rasanen L.	Get it UTL
Publisher	Springer-Verlag France	
Publication Type	Article	

Cite + My Projects + Annotate

74.

Complete Reference

Title	**Developing specialist healthcare for older people: A challenge for the European Union.**	Find Similar<br>Find Citing Articles
Source	Journal of Nutrition, Health and Aging. 8 (2) (pp 109-112), 2004. Date of Publication: 2004.	
Author	O'Neill D.; Hastie I.; Williams B.	Get it UTL
Publisher	Springer-Verlag France	
Publication Type	Review	

Cite + My Projects + Annotate

75.

Abstract Reference
Complete Reference

Title	**Administering the "AHSP questionnaire" (appetite, hunger, sensory perception) in a geriatric rehabilitation care.**	Find Similar<br>Find Citing Articles
Source	Journal of Nutrition, Health and Aging. 7 (6) (pp 385-389), 2003. Date of Publication: 2003.	
Author	Savina C.; Donini L.M.; Anzivino R.; De Felice M.R.; De Bernardini L.; Cannella C.	Get it UTL
Publisher	Springer-Verlag Italia s.r.l.	
Publication Type	Article	

Cite + My Projects + Annotate

76.

Abstract Reference
Complete Reference

Title	**Dementia and nutrition. Intervention study in institutionalized patients with Alzheimer Disease.**	Find Similar<br>Find Citing Articles
Source	Journal of Nutrition, Health and Aging. 7 (5) (pp 304-308), 2003. Date of Publication: 2003.	
Author	Gil Gregorio P.; Ramirez Diaz S.P.; Ribera Casado J.M.; Tobaruela J.L.; Neira R.; Medina J.; Gonzalez P.; Navarro C.; Robledillo R.; Moreno J.; Teja J.	Get it UTL
Publisher	Springer-Verlag Italia s.r.l.	
Publication Type	Article	

Cite + My Projects + Annotate

77.

Abstract Reference
Complete Reference

Find Similar
Find Citing Articles

Get it UTL

**Title**	**Nutritional intake and daily functioning of psychogeriatric nursing home residents.**
**Source**	Journal of Nutrition, Health and Aging. 7 (4) (pp 242-246), 2003. Date of Publication: 2003.
**Author**	Deijen J.B.; Slump E.; Wouters-Wesseling W.; De Groot C.P.G.M.; Gallie E.; Pas H.
**Publisher**	Springer-Verlag Italia s.r.l.
**Publication Type**	Article

Cite + My Projects + Annotate

78.

Abstract Reference
Complete Reference

Find Similar
Find Citing Articles

Get it UTL

**Title**	**Correlates of cognitive impairment in elderly residents of long term care institutions in the Metropolitan area of Guadalajara, Mexico.**
**Source**	Journal of Nutrition, Health and Aging. 7 (2) (pp 97-101), 2003. Date of Publication: 2003.
**Author**	Arias-Merino E.D.; Orozco-Mares I.; Garabito-Esparza L.C.; Fernandez-Cruz L.; Arias-Merino M.J.; De La Rosa A.C.; Cabrera-Ivaral C.; Gonzalez-Perez G.J.
**Publisher**	Springer-Verlag Italia s.r.l.
**Publication Type**	Article

Cite + My Projects + Annotate

79.

Abstract Reference
Complete Reference

Find Similar
Find Citing Articles

Get it UTL

**Title**	**Enteral nutrition in French institutionalized patients: A multicentric study.**
**Source**	Journal of Nutrition, Health and Aging. 6 (5) (pp 301-305), 2002. Date of Publication: 2002.
**Author**	Pfitzenmeyer P.; Manckoundia P.; Mischis-Troussard C.; D'Athis P.H.; Michel M.; Lussier M.D.; Derycke B.; Hermet R.; Collart M.; Leurs P.
**Publisher**	Springer-Verlag Italia s.r.l.
**Publication Type**	Article

Cite + My Projects + Annotate

80.

Abstract Reference
Complete Reference

Find Similar
Find Citing Articles

**Title**	**Weight loss and metabolic changes in dementia.**
**Source**	Journal of Nutrition, Health and Aging. 6 (3) (pp 201-205), 2002. Date of Publication: 2002.

Author Wang S.Y.

Publisher Springer-Verlag Italia s.r.l.

Publication Type Article

Get it UTL

Cite + My Projects + Annotate

81.

Abstract Reference
Complete Reference

Title **Psychometric comparison of the scales: PAINAD and Abbey Pain Scale in long-Term care facilities in Barcelona.**

Original Title Comparacion psicometrica de las escalas: PAINAD y Abbey Pain Scale en centros sociosanitarios de Barcelona.

Source Medicina Paliativa. 28 (no pagination), 2021. Date of Publication: 2021.

Author Gonzalez-Vaca J.; Cobo C.M.S.; Azuela E.M.

Publisher Sociedad Espanola de Cuidados Paliativos

Publication Type Article

Find Similar
Find Citing Articles

Get it UTL

Cite + My Projects + Annotate

82.

Abstract Reference
Complete Reference

Title **Revisiting the Role of Physicians in Assisted Living and Residential Care Settings.**

Source Gerontology and Geriatric Medicine. 6 (no pagination), 2020. Date of Publication: 2020.

Author Dys S.; Smith L.; Tunalilar O.; Carder P.

Publisher SAGE Publications Inc.

Publication Type Article

Find Similar
Find Citing Articles

Get it UTL

Cite + My Projects + Annotate

83.

Abstract Reference
Complete Reference

Title **Analysis of C9orf72 Intermediate Alleles in a Retrospective Cohort of Neurological Patients: Risk Factors for Alzheimer's Disease?.**

Source Journal of Alzheimer's Disease. 81 (4) (pp 1445-1451), 2021. Date of Publication: 2021.

Author Serpente M.; Fenoglio C.; Arighi A.; Fumagalli G.G.; Arcaro M.; Sorrentino F.; Visconte C.; Scarpini E.; Galimberti D.

Publisher IOS Press BV

Publication Type Article

Find Similar
Find Citing Articles

Get it UTL

Cite + My Projects + Annotate

84.

Abstract Reference
Complete Reference

**Title**	Impact of Dementia-Related Behavioral Symptoms on Healthcare Resource Use and Caregiver Burden: Real-World Data from Europe and the United States.
**Source**	Journal of Alzheimer's Disease. 81 (4) (pp 1567-1578), 2021. Date of Publication: 2021.
**Author**	Chekani F.; Pike J.; Jones E.; Husbands J.; Khandker R.K.
**Publisher**	IOS Press BV
**Publication Type**	Article

Find Similar
Find Citing Articles

Get it UTL

Cite + My Projects + Annotate

85.

Abstract Reference
Complete Reference

**Title**	The effect of pre-operative high doses of methylprednisolone on pain management and convalescence after total hip replacement in elderly: a double-blind randomized study.
**Source**	International Orthopaedics. 45 (4) (pp 857-863), 2021. Date of Publication: April 2021.
**Author**	Gadek A.; Liszka H.; Zajac M.
**Publisher**	Springer Science and Business Media Deutschland GmbH
**Publication Type**	Article

Find Similar
Find Citing Articles

Full Text

Cite + My Projects + Annotate

86.

Abstract Reference
Complete Reference

**Title**	Cistanche deserticola polysaccharides alleviate cognitive decline in aging model mice by restoring the gut microbiota-brain axis.
**Source**	Aging. 13 (11) (pp 15320-15335), 2021. Date of Publication: 03 Jun 2021.
**Author**	Gao Y.; Li B.; Liu H.; Tian Y.; Gu C.; Du X.; Bu R.; Gao J.; Liu Y.; Li G.
**Publisher**	NLM (Medline)
**Publication Type**	Article

Find Similar
Find Citing Articles

Full Text

Cite + My Projects + Annotate

87.

Abstract Reference
Complete Reference

**Title**	Covid-19 mortality rates among nursing home residents declined from march to november 2020.
**Source**	Health Affairs. 40 (4) (pp 655-663), 2021. Date of Publication: April 2021.
**Author**	Kosar C.M.; White E.M.; Feifer R.A.; Blackman C.; Gravenstein S.; Panagiotou O.A.; McConeghy K.; Mor V.
**Publisher**	Project HOPE

Find Similar
Find Citing Articles

Get it UTL

Publication Type: Article

Cite + My Projects + Annotate

88.

Abstract Reference
Complete Reference
Find Similar
Find Citing Articles
Full Text

Title	**Namaste care delivered by caregivers of community-dwelling older adults with moderate to advanced dementia: A mixed methods study protocol.**
Source	Journal of advanced nursing. 77 (2) (pp 1027-1036), 2021. Date of Publication: 01 Feb 2021.
Author	Yous M.-L.; Ploeg J.; Kaasalainen S.; McAiney C.
Publisher	NLM (Medline)
Publication Type	Article

Cite + My Projects + Annotate

89.

Abstract Reference
Complete Reference
Find Similar
Find Citing Articles
Full Text

Title	**Drugs as a custodial measure in residential nursing homes? A critical analysis.**
Original Title	Medikamente als freiheitsentziehende Masnahme in stationaren Pflegeeinrichtungen? Eine kritische Analyse.
Source	Rechtsmedizin. 31 (2) (pp 101-109), 2021. Date of Publication: April 2021.
Author	Gleich S.; Kruger J.; Fels H.; Skopp G.; Musshoff F.; Roider G.; Schopfer J.; Graw M.; Wiedfeld C.
Publisher	Springer Medizin
Publication Type	Article

Cite + My Projects + Annotate

90.

Abstract Reference
Complete Reference
Find Similar
Find Citing Articles
Full Text

Title	**The use of physical restraints in Chinese long-term care facilities and its risk factors: An observational and cross-sectional study.**
Source	Journal of advanced nursing. 76 (10) (pp 2597-2609), 2020. Date of Publication: 01 Oct 2020.
Author	Wang J.; Liu W.; Peng D.; Xiao M.; Zhao Q.
Publisher	NLM (Medline)
Publication Type	Article

Cite + My Projects + Annotate

91.

**Title**	**The Impact of a Randomized Controlled Trial Testing the Implementation of Function-Focused Care in Assisted Living on Resident Falls, Hospitalizations, and Nursing Home Transfers.**	Abstract Reference<br>Complete Reference<br>Find Similar<br>Find Citing Articles<br>Get it UTL
**Source**	Journal of aging and physical activity. (pp 1-9), 2021. Date of Publication: 17 Jun 2021.	
**Author**	Resnick B.; Boltz M.; Galik E.; Zhu S.	
**Publisher**	NLM (Medline)	
**Publication Type**	Article	

Cite + My Projects + Annotate

92.

**Title**	**Stroke rehabilitation in nursing homes: How do we measure quality?.**	Abstract Reference<br>Complete Reference<br>Find Similar<br>Find Citing Articles<br>Get it UTL
**Source**	Clinics in Geriatric Medicine. 15 (4) (pp 869-884), 1999. Date of Publication: 1999.	
**Author**	Kramer A.M.; Coleman E.A.	
**Publisher**	W.B. Saunders	
**Publication Type**	Review	

Cite + My Projects + Annotate

93.

**Title**	**Successful Discharge to Community Gap of FFS Medicare Beneficiaries With and Without ADRD Narrowed.**	Abstract Reference<br>Complete Reference<br>Find Similar<br>Find Citing Articles<br>Full Text
**Source**	Journal of the American Geriatrics Society. 69 (4) (pp 972-978), 2021. Date of Publication: April 2021.	
**Author**	Bardenheier B.H.; Rahman M.; Kosar C.; Werner R.M.; Mor V.	
**Publisher**	Blackwell Publishing Inc.	
**Publication Type**	Article	

Cite + My Projects + Annotate

94.

**Title**	**Psychotropic drug use in residents with dementia living in small-scaled special care facilities; a longitudinal study.**	Abstract Reference<br>Complete Reference<br>Find Similar<br>Find Citing Articles<br>Get it UTL
**Source**	Aging & mental health. 24 (4) (pp 689-696), 2020. Date of Publication: 01 Apr 2020.	
**Author**	Kok J.S.; Oude Voshaar R.C.; Scherder E.J.A.	
**Publisher**	NLM (Medline)	
**Publication Type**	Article	

Cite + My Projects + Annotate

95.

Abstract Reference
Complete Reference

**Title**	**Nursing homes underreport antipsychotic prescribing.**
**Source**	Aging & mental health. 24 (4) (pp 668-672), 2020. Date of Publication: 01 Apr 2020.
**Author**	Briesacher B.A.; Mui B.; Devlin J.W.; Koethe B.
**Publisher**	NLM (Medline)
**Publication Type**	Article

Find Similar
Find Citing Articles
Get it UTL

Cite + My Projects + Annotate

96.

Abstract Reference
Complete Reference

**Title**	**Temporal trends in nutrition intake among older long-term care residents.**
**Source**	Clinical Nutrition. 40 (6) (pp 3793-3797), 2021. Date of Publication: June 2021.
**Author**	Salminen K.; Willman M.; Kautiainen H.; Pitkala K.; Roitto H.-M.; Suominen M.
**Publisher**	Churchill Livingstone
**Publication Type**	Article

Find Similar
Find Citing Articles
Get it UTL

Cite + My Projects + Annotate

97.

Abstract Reference
Complete Reference

**Title**	**Trajectories of functional performance recovery after inpatient geriatric rehabilitation: an observational study.**
**Source**	Medical Journal of Australia. (no pagination), 2021. Date of Publication: 2021.
**Author**	Soh C.H.; Reijnierse E.M.; Tuttle C.; Marston C.; Goonan R.; Lim W.K.; Maier A.B.
**Publisher**	John Wiley and Sons Inc
**Publication Type**	Article

Find Similar
Find Citing Articles
Get it UTL

Cite + My Projects + Annotate

98.

Abstract Reference
Complete Reference

**Title**	**A pharmacist-led pilot using a performance dashboard to improve psychotropic medication use in a skilled nursing facility.**
**Source**	BMJ open quality. 9 (3) (no pagination), 2020. Date of Publication: 01 Aug 2020.
**Author**	Bell K.; Hartmann C.; Baughman A.W.

Find Similar
Find Citing Articles
Get it UTL

Publisher NLM (Medline)

Publication Type Article

Cite + My Projects + Annotate

☐ 99.

Title What Determines Step-Rate at Work? An Investigation of Factors at the Shift, Worker, Ward, and Nursing Home Levels in Eldercare.

Source Annals of work exposures and health. (no pagination), 2021. Date of Publication: 17 Jun 2021.

Author Stevens M.L.; Karstad K.; Mathiassen S.E.; Januario L.B.; Holtermann A.; Hallman D.M.

Publisher NLM (Medline)

Publication Type Article

Abstract Reference
Complete Reference

Find Similar
Find Citing Articles

Get it UTL

Cite + My Projects + Annotate

☐ 100.

Title Impact of place of residence, frailty and other factors on rehabilitation outcomes post hip fracture.

Source Age and Ageing. 50 (2) (pp 423-430), 2021. Date of Publication: 01 Mar 2021.

Author Low S.; Wee E.; Dorevitch M.

Publisher Oxford University Press

Publication Type Article

Abstract Reference
Complete Reference

Find Similar
Find Citing Articles

Get it UTL

Cite + My Projects + Annotate

© 2021 Ovid Technologies, Inc. All rights reserved. OvidUI_04.14.00.086, SourceID 90004a473d2e16b9a99bd726b1500dc38f7baefd

▼ **Search History** (34)

View Saved

# ▲	Searches	Results	Type	Actions	Annotations
1	(Transition* adj3 (care* or unit* or bed* or program* or ward* or setting* or facilit* or service* or model* or centre* or center*)).tw.	9860	Advanced	Display Results | More	
2	(Intermediate adj3 (unit* or care or bed* or program* or ward* or setting* or facilit* or service* or model* or centre* or center*)).tw.	1067	Advanced	Display Results | More	
3	((Subacute or sub-acute) adj3 (unit* or care or bed* or program* or ward* or setting* or facilit* or service* or model* or centre* or center*)).tw.	295	Advanced	Display Results | More	
4	((Postacute or post-acute) adj3 (unit* or care or bed* or program* or ward* or setting* or facilit* or service* or model* or centre* or center*)).tw.	464	Advanced	Display Results | More	
5	(Post acute adj3 (unit* or care or bed* or program* or ward* or setting* or facilit* or service* or model* or centre* or center*)).tw.	308	Advanced	Display Results | More	
6	(Skilled Nursing adj3 (unit* or bed* or program* or ward* or setting* or facilit* or service* or model* or centre* or center*)).tw.	650	Advanced	Display Results | More	
7	(Restor* adj3 (unit* or care or bed* or program* or ward* or setting* or facilit* or service* or model* or centre* or center*)).tw.	1042	Advanced	Display Results | More	
8	(Convalesc* adj3 (unit* or care or bed* or program* or ward* or setting* or facilit* or service* or model* or centre* or center* or home* or hospital*)).tw.	184	Advanced	Display Results | More	
9	or/1-8	13339	Advanced	Display Results | More	
10	(Old* or aged or aging).tw.	703913	Advanced	Display Results | More	
11	(Centenarian* or nonagenarian* or octogenarian* or geriatr* or gerontol* or senescen* or septuagenarian* or pensioner* or senile).tw.	30323	Advanced	Display Results | More	
12	Senior*.tw.	29638	Advanced	Display Results | More	
13	Elder*.tw.	74632	Advanced	Display Results | More	
14	Geriatric patients/	13732	Advanced	Display Results | More	
15	or/10-14	763909	Advanced	Display Results | More	
16	(Cognit* adj3 impair*).tw.	50210	Advanced	Display Results | More	
17	Mild neurocognitive disorder*.tw.	154	Advanced	Display Results | More	
18	Cognitive Impairment/	39404	Advanced	Display Results | More	
19	Mild Cognitive Impairment/	7724	Advanced	Display Results | More	
20	Major neurocognitive disorder*.tw.	162	Advanced	Display Results | More	
21	Dement*.tw.	72747	Advanced	Display Results | More	
22	Dementia/	37016	Advanced	Display Results | More	
23	"Alzheimer's Disease"/	49326	Advanced	Display Results | More	

Contract

24	Alzheimer*.tw.	65308	Advanced	Display Results | More	
25	AIDS Dementia Complex/	154	Advanced	Display Results | More	
26	Dementia with Lewy Bodies/	2064	Advanced	Display Results | More	
27	Presenile Dementia/	287	Advanced	Display Results | More	
28	Senile Dementia/	1074	Advanced	Display Results | More	
29	Semantic Dementia/	2279	Advanced	Display Results | More	
30	Vascular Dementia/	2193	Advanced	Display Results | More	
31	Deliri*.tw.	7766	Advanced	Display Results | More	
32	Delirium/	3576	Advanced	Display Results | More	
33	or/16-32	155522	Advanced	Display Results | More	
34	9 and 15 and 33	334	Advanced	Display Results | More	

Save Remove Combine with: AND OR

Save All Edit Create RSS View Saved Email All Search History Copy Search History Link Copy Search History Details

Advanced Search | Basic Search | Find Citation | Search Tools | Search Fields | Multi-Field Search

1 Resource selected | Hide | Change

APA PsycInfo 1806 to July Week 1 2021

Enter keyword or phrase (* or $ for truncation)

Keyword Author Title Journal

Search

Limits *(close)* Include Multimedia Map Term to Subject Heading

Human English Language

Publication Year - -

Additional Limits Edit Limits

Options

To search Open Access content on Ovid, go to Basic Search.

Print Email Export + My Projects Keep Selected

All Range Clear 30 Per Page 1 Go Next ›

Search Information

You searched:

9 and 15 and 33

Search terms used:

aged
aging
aids
dementia
complex
alzheimer's
disease
alzheimer*
bed*
care
care*
centenarian*
center*
centre*

1.

Title **Uncertainty in transition of African American caregivers. [References].**

Year of Publication 2020

Author Unson, Christine; Flynn, Deborah; Chukwurah, Queendalene; Glendon, Mary Anne; Testut, Tammy.

Source Issues in Mental Health Nursing. Vol.41(5), 2020, pp. 445-454.

Publication Month/Season May

Publication Type Journal; Peer Reviewed Journal

Abstract Reference
Complete Reference

Find Similar
Find Citing Articles

Get it UTL

cognit*
cognitive
impairment
convalesc*
deliri*
delirium
dement*
with
lewy
bodies
elder*
facilit*
geriatr*
geriatric
patients
gerontol*
home*
hospital*
impair*
intermediate
major
neurocognitive
disorder*
mild
model*
nonagenarian*
octogenarian*
old*
pensioner*
post
acute
post-acute
postacute
presenile
program*
restor*
semantic
senescen*
senile
senior*
septuagenarian*
service*
setting*
skilled
nursing
sub-acute
subacute
transition*
unit*
vascular
ward*

Search Returned:
334 text results

Sort By:
-

Customize Display

Filter By

Add to Search History

Selected Only (0)

Years

Cite + My Projects + Annotate

2.

**Title**	**Analysis of discharge documentation for older adults living with dementia: A cohort study.**
**Year of Publication**	2021
**Author**	Parker, Kirsten J; Phillips, Jane L; Luckett, Tim; Agar, Meera; Ferguson, Caleb; Hickman, Louise D.
**Source**	Journal of Clinical Nursing. 2021, pp. No Pagination Specified.
**Publication Month/Season**	Jun
**Publication Type**	Journal; Peer Reviewed Journal

Abstract Reference
Complete Reference
Find Similar
Find Citing Articles
Get it UTL

Cite + My Projects + Annotate

3.

**Title**	**Ageing- and dementia-friendly design: Theory and evidence from cognitive psychology, neuropsychology and environmental psychology can contribute to design guidelines that minimise spatial disorientation. [References].**
**Year of Publication**	2021
**Author**	Wiener, Jan M; Pazzaglia, Francesca.
**Source**	Cognitive Processing. 2021, pp. No Pagination Specified.
**Publication Month/Season**	May
**Publication Type**	Journal; Peer Reviewed Journal

Abstract Reference
Complete Reference
Find Similar
Find Citing Articles
Get it UTL

Cite + My Projects + Annotate

4.

**Title**	**The potential role of protein kinase R as a regulator of age-related neurodegeneration. [References].**
**Year of Publication**	2021
**Author**	Martinez, Nicolas W; Gomez, Felipe E; Matus, Soledad.
**Source**	Frontiers in Aging Neuroscience. Vol.13 2021, ArtID 638208.
**Publication Month/Season**	Apr
**Publication Type**	Journal; Peer Reviewed Journal

Abstract Reference
Complete Reference
Find Similar
Find Citing Articles
Get it UTL

Cite + My Projects + Annotate

All Years
Current year
Past 3 years
Past 5 years
▶ Specific Year Range

▶ Subject
▶ Author
▶ Journal
▶ Book
▶ Publication Type

My Projects

+ New Project

No projects available.

5.

Title	**Availability of home palliative care services and dying at home in conditions needing palliative care: A population-based death certificate study. [References].**
Year of Publication	2020
Author	Nakanishi, Miharu; Ogawa, Asao; Nishida, Atsushi.
Source	Palliative Medicine. Vol.34(4), 2020, pp. 504-512.
Publication Month/Season	Apr
Publication Type	Journal; Peer Reviewed Journal

Abstract Reference
Complete Reference
Find Similar
Find Citing Articles
Get it UTL

Cite + My Projects + Annotate

6.

Title	**An exploration of dementia friendly communities from the perspective of persons living with dementia.**
Year of Publication	2021
Author	Hebert, Catherine Ann.
Source	Dissertation Abstracts International: Section B: The Sciences and Engineering. Vol.82(8-B),2021, pp. No Pagination Specified.
Publication Type	Dissertation Abstract

Abstract Reference
Complete Reference
Find Similar
Find Citing Articles
Get it UTL

Cite + My Projects + Annotate

7.

Title	**Cognitive screening tools for late career physicians: A critical review. [References].**
Year of Publication	2021
Author	Garrett, Kelly Davis; Perry, William; Williams, Betsy; Korinek, Lauri; Bazzo, David E. J.
Source	Journal of Geriatric Psychiatry and Neurology. Vol.34(3), 2021, pp. 171-180.
Publication Month/Season	May
Publication Type	Journal; Peer Reviewed Journal

Abstract Reference
Complete Reference
Find Similar
Find Citing Articles
Get it UTL

Cite + My Projects + Annotate

8.

Title	**Delirium screening and prevention in older adult post-acute care patients.**
Year of	2021

Abstract Reference
Complete Reference
Find Similar

Publication

Author: Spear, Rebecca Anne.

Source: Dissertation Abstracts International: Section B: The Sciences and Engineering. Vol.82(7-B),2021, pp. No Pagination Specified.

Publication Type: Dissertation Abstract

Find Citing Articles

Get it UTL

Cite + My Projects + Annotate

9.

Title: **Cerebrovascular damage after midlife transient hypertension in non-transgenic and Alzheimer's disease rats. [References].**

Year of Publication: 2021

Author: Lai, Aaron Y; Joo, Illsung L; Trivedi, Arunachala U; Dorr, Adrienne; Hill, Mary E; Stefanovic, Bojana; McLaurin, JoAnne.

Source: Brain Research. Vol.1758 2021, ArtID 147369.

Publication Month/Season: May

Publication Type: Journal; Peer Reviewed Journal

Abstract Reference
Complete Reference

Find Similar
Find Citing Articles

Get it UTL

Cite + My Projects + Annotate

10.

Title: **Perceptions of Missouri special education teachers concerning their role in writing and implementing transition plans.**

Year of Publication: 2021

Author: Stewart, Tana.

Source: Dissertation Abstracts International Section A: Humanities and Social Sciences. Vol.82(6-A),2021, pp. No Pagination Specified.

Publication Type: Dissertation Abstract

Abstract Reference
Complete Reference

Find Similar
Find Citing Articles

Get it UTL

Cite + My Projects + Annotate

11.

Title: **Chapter: "We have been robbed of the life we planned": Relational turbulence and experiences of Alzheimer's disease. [References].**

Year of Publication: 2019

Author: Catona, Danielle.

Source: Theiss, Jennifer A [Ed]; Greene, Kathryn [Ed]. (2019). Contemporary studies on relationships, health, and wellness. (pp. 139-159). xiv, 365 pp. New York, NY, US: Cambridge University Press; US.

Publication: Book; Edited Book

Abstract Reference
Complete Reference

Find Similar
Find Citing Articles

Get it UTL

Type

Cite + My Projects + Annotate

12.

Abstract Reference
Complete Reference

Find Similar
Find Citing Articles

Full Text

**Title**	**Association of positive delirium screening with incident dementia in skilled nursing facilities. [References].**
**Year of Publication**	2020
**Author**	Briesacher, Becky A; Koethe, Benjamin; Olivieri-Mui, Brianne; Saczynski, Jane S; Fick, Donna Marie; Devlin, John W; Marcantonio, Edward R.
**Source**	Journal of the American Geriatrics Society. Vol.68(12), 2020, pp. 2931-2936.
**Publication Month/Season**	Dec
**Publication Type**	Journal; Peer Reviewed Journal

Cite + My Projects + Annotate

13.

Abstract Reference
Complete Reference

Find Similar
Find Citing Articles

Get it UTL

**Title**	**Top ten tips palliative care clinicians should know about cognitive impairment and institutional care. [References].**
**Year of Publication**	2020
**Author**	Schlogl, Mathias; Riese, Florian; Little, Milta O; Blum, David; Jox, Ralf J; O'Neill, Lynn; Pautex, Sophie; Piers, Ruth; Way, Deborah; Jones, Christopher A.
**Source**	Journal of Palliative Medicine. Vol.23(11), 2020, pp. 1525-1531.
**Publication Month/Season**	Nov
**Publication Type**	Journal; Peer Reviewed Journal

Cite + My Projects + Annotate

14.

Abstract Reference
Complete Reference

Find Similar
Find Citing Articles

Get it UTL

**Title**	**The license plate test performance in Canadian adolescents with learning disabilities: A preliminary study. [References].**
**Year of Publication**	2020
**Author**	Harrison, Allyson G; Armstrong, Irene.
**Source**	Applied Neuropsychology: Child. Vol.9(4), 2020, pp. 360-366.
**Publication Month/Season**	Oct-Dec
**Publication Type**	Journal; Peer Reviewed Journal

Cite + My Projects + Annotate

15.

Title [Risk factors and outcomes of sepsis-associated delirium in intensive care unit patients: A secondary data analysis. [References].]

Year of Publication 2020

Author Kim, Yeunwoo; Jin, Yinji; Jin, Taixian; Lee, Sun-Mi.

Source Intensive and Critical Care Nursing. Vol.59 2020, ArtID 102844.

Publication Month/Season Aug

Publication Type Journal; Peer Reviewed Journal

Abstract Reference
Complete Reference
Find Similar
Find Citing Articles
Full Text

Cite + My Projects + Annotate

16.

Title Care home life and identity: A qualitative case study. [References].

Year of Publication 2019

Author Paddock, Katie; Wilson, Christine Brown; Walshe, Catherine; Todd, Chris.

Source The Gerontologist. Vol.59(4), 2019, pp. 655-664.

Publication Month/Season Aug

Publication Type Journal; Peer Reviewed Journal

Abstract Reference
Complete Reference
Find Similar
Find Citing Articles
Get it UTL

Cite + My Projects + Annotate

17.

Title Frailty phenotype and healthcare costs and utilization in older men. [References].

Year of Publication 2020

Author Ensrud, Kristine E; Kats, Allyson M; Schousboe, John T; Taylor, Brent C; Vo, Tien N; Cawthon, Peggy M; Hoffman, Andrew R; Langsetmo, Lisa.

Source Journal of the American Geriatrics Society. Vol.68(9), 2020, pp. 2034-2042.

Publication Month/Season Sep

Publication Type Journal; Peer Reviewed Journal

Abstract Reference
Complete Reference
Find Similar
Find Citing Articles
Full Text

Cite + My Projects + Annotate

18.

Abstract Reference
Complete Reference

Find Similar
Find Citing Articles

Get it UTL

**Title**	**Promoting physical activity in geriatric patients with cognitive impairment after discharge from ward-rehabilitation: A feasibility study. [References].**
**Year of Publication**	2020
**Author**	Eckert, Tobias; Bongartz, Martin; Ullrich, Phoebe; Abel, Bastian; Christian, Werner; Kiss, Rainer; Hauer, Klaus.
**Source**	European Journal of Ageing. Vol.17(3), 2020, pp. 309-320.
**Publication Month/Season**	Sep
**Publication Type**	Journal; Peer Reviewed Journal

Cite + My Projects + Annotate

19.

Abstract Reference
Complete Reference

Find Similar
Find Citing Articles

Full Text

**Title**	**The association of cognitive impairment as screened by the Mini-Cog with long term post-hospitalization outcomes. [References].**
**Year of Publication**	2019
**Author**	Shami, Ali; Brennan, Maura; Marie, Peter St; Lindenauer, Peter K; Stefan, Mihaela S.
**Source**	Archives of Gerontology and Geriatrics. Vol.85 2019, ArtID 103916.
**Publication Month/Season**	Nov-Dec
**Publication Type**	Journal; Peer Reviewed Journal

Cite + My Projects + Annotate

20.

Abstract Reference
Complete Reference

Find Similar
Find Citing Articles

Get it UTL

**Title**	**Chapter: Assessment of sexual consent capacity. [References].**
**Year of Publication**	2020
**Author**	Katz, Emma; Spalding, Rachael; Gallegos, Jarred V; Edelstein, Barry.
**Source**	Moye, Jennifer [Ed]. (2020). Assessing capacities of older adults: A casebook to guide difficult decisions. (pp. 209-241). xii, 337 pp. Washington, DC, US: American Psychological Association; US.
**Publication Type**	Book; Edited Book

Cite + My Projects + Annotate

21.

Title	Cognitive limitations in older adults receiving care reduces well-being among spouse caregivers. [References].	Abstract Reference<br>Complete Reference<br>Find Similar<br>Find Citing Articles<br>Full Text
Year of Publication	2020	
Author	Hawkley, Louise; Zheng, Boyan; Hedberg, E. C; Huisingh-Scheetz, Megan; Waite, Linda.	
Source	Psychology and Aging. Vol.35(1), 2020, pp. 28-40.	
Publication Month/Season	Feb	
Publication Type	Journal; Peer Reviewed Journal	

Cite + My Projects + Annotate

22.

Title	Coping with cognitive impairment and family caregiving: Introduction to the special section. [References].	Abstract Reference<br>Complete Reference<br>Find Similar<br>Find Citing Articles<br>Full Text
Year of Publication	2020	
Author	Cheng, Sheung-Tak; Haley, William E.	
Source	Psychology and Aging. Vol.35(1), 2020, pp. 1-7.	
Publication Month/Season	Feb	
Publication Type	Journal; Peer Reviewed Journal	

Cite + My Projects + Annotate

23.

Title	Vietnamese family caregivers' adjustment process to their caregiving roles for family members with dementia.	Abstract Reference<br>Complete Reference<br>Find Similar<br>Find Citing Articles<br>Get it UTL
Year of Publication	2020	
Author	Nguyen, Trang.	
Source	Dissertation Abstracts International Section A: Humanities and Social Sciences. Vol.81(2-A),2020, pp. No Pagination Specified.	
Publication Type	Dissertation Abstract	

Cite + My Projects + Annotate

24.

Title	An hourly rounding program to reduce falls and improve overall health of dementia residents 65 years and older.	Abstract Reference<br>Complete Reference<br>Find Similar<br>Find Citing Articles
Year of Publication	2020	

**Author**	Merritt, Cathy.	Get it UTL
**Source**	Dissertation Abstracts International: Section B: The Sciences and Engineering. Vol.81(12-B),2020, pp. No Pagination Specified.	
**Publication Type**	Dissertation Abstract	

Cite + My Projects + Annotate

25.

Abstract Reference
Complete Reference

Find Similar
Find Citing Articles

Get it UTL

**Title**	**Evaluating the sensitivity and specificity of the Adult Functional Adaptive Behavior Scale to cognitive impairment in the geriatric population.**
**Year of Publication**	2020
**Author**	Hughes, Taylor.
**Source**	Dissertation Abstracts International: Section B: The Sciences and Engineering. Vol.81(3-B),2020, pp. No Pagination Specified.
**Publication Type**	Dissertation Abstract

Cite + My Projects + Annotate

26.

Abstract Reference
Complete Reference

Find Similar
Find Citing Articles

Get it UTL

**Title**	**Feasibility of hair cortisol as a biomarker of well-being in older adults with dementia.**
**Year of Publication**	2020
**Author**	Kim, Eunsaem.
**Source**	Dissertation Abstracts International: Section B: The Sciences and Engineering. Vol.81(5-B),2020, pp. No Pagination Specified.
**Publication Type**	Dissertation Abstract

Cite + My Projects + Annotate

27.

Abstract Reference
Complete Reference

Find Similar
Find Citing Articles

Get it UTL

**Title**	**Care transitions in the psychiatric hospital: Focus on older adults. [References].**
**Year of Publication**	2020
**Author**	Conlon, Matthew; Tew, James; Solai, LalithKumer K; Gopalan, Priya; Azzam, Pierre; Karp, Jordan F.
**Source**	The American Journal of Geriatric Psychiatry. Vol.28(3), 2020, pp. 368-377.
**Publication Month/Season**	Mar
**Publication Type**	Journal; Peer Reviewed Journal

Cite + My Projects + Annotate

28.

Title	**Chapter: Cognitive behavior therapy with culturally diverse older adults. [References].**	Abstract Reference<br>Complete Reference<br>Find Similar<br>Find Citing Articles<br>Get it UTL
Year of Publication	2019	
Author	Lau, Angela W; Kinoshita, Lisa M.	
Source	Iwamasa, Gayle Y [Ed]; Hays, Pamela A [Ed]. (2019). Culturally responsive cognitive behavior therapy: Practice and supervision., 2nd ed. (pp. 231-256). xi, 348 pp. Washington, DC, US: American Psychological Association; US.	
Publication Type	Book; Edited Book	

Cite + My Projects + Annotate

29.

Title	**Psychosocial health interventions by social robots: Systematic review of randomized controlled trials. [References].**	Abstract Reference<br>Complete Reference<br>Find Similar<br>Find Citing Articles<br>Full Text
Year of Publication	2019	
Author	Robinson, Nicole Lee; Cottier, Timothy Vaughan; Kavanagh, David John.	
Source	Journal of Medical Internet Research. Vol.21(5), 2019, ArtID e13203.	
Publication Month/Season	May	
Publication Type	Journal; Peer Reviewed Journal	

Cite + My Projects + Annotate

30.

Title	**Distinguishing characteristics of delirium in a skilled nursing facility in Spain: Influence of baseline cognitive status. [References].**	Abstract Reference<br>Complete Reference<br>Find Similar<br>Find Citing Articles<br>Full Text
Year of Publication	2019	
Author	Franco, Jose G; Trzepacz, Paula T; Gaviria, Ana M; Sepulveda, Esteban; Vinuelas, Eva; Palma, Jose; Grau, Imma; Vilella, Elisabet.	
Source	International Journal of Geriatric Psychiatry. Vol.34(8), 2019, pp. 1217-1225.	
Publication Month/Season	Aug	
Publication Type	Journal; Peer Reviewed Journal	

Cite + My Projects + Annotate

© 2021 Ovid Technologies, Inc. All rights reserved. OvidUI_04.14.00.086, SourceID 90004a473d2e16b9a99bd726b1500dc38f7baefd

Cochrane
Library

Access provided by: **University of Toronto Library**

Advanced Search

Search manager

Save this search | View saved searches | ? Search help

Cochrane Search_TCP Systematic Review_July 15, 2021

Last saved on: 15/07/2021 18:46:23

✔ Search saved.

View fewer lines | Print

#	Search				Results
#1	(Transition* NEAR/3 (care* or unit* or bed* or program* or ward* or setting* or facilit* or service* or model* or centre* or center*)):ti,ab,kw	S	MeSH	Limits	1633
#2	[mh ^"Transitional care"]			Limits	60
#3	(Intermediate NEAR/3 (unit* or care or bed* or program* or ward* or setting* or facilit* or service* or model* or centre* or center*)):ti,ab,kw			Limits	420
#4	[mh ^"Intermediate care facilities"]			Limits	14
#5	((Subacute or sub-acute) NEAR/3 (unit* or care or bed* or program* or ward* or setting* or facilit* or service* or model* or centre* or center*)):ti,ab,kw			Limits	280
#6	[mh ^"Subacute care"]			Limits	19
#7	((Postacute or post-acute) NEAR/3 (unit* or care or bed* or program* or ward* or setting* or facilit* or service* or model* or centre* or center*)):ti,ab,kw			Limits	258
#8	((Post acute) NEAR/3 (unit* or care or bed* or program* or ward* or setting* or facilit* or service* or model* or centre* or center*)):ti,ab,kw			Limits	12292
#9	((Skilled Nursing) NEAR/3 (unit* or bed* or program* or ward* or setting* or facilit* or service* or model* or centre* or center*)):ti,ab,kw			Limits	3044
#10	[mh ^"Skilled nursing facilities"]			Limits	65

#11	(Restor* NEAR/3 (unit* or care or bed* or program* or ward* or setting* or facilit* or service* or model* or centre* or center*)):ti,ab,kw	Limits	392
#12	(Convalesc* NEAR/3 (unit* or care or bed* or program* or ward* or setting* or facilit* or service* or model* or centre* or center* or home* or hospital*)):ti,ab,kw	Limits	216
#13	[mh ^"Convalescence"]	Limits	150
#14	{or #1-#13}	Limits	17752
#15	(Old* or aged or aging):ti,ab,kw	Limits	586822
#16	(Centenarian* or nonagenarian* or octogenarian* or geriatr* or gerontol* or senescen* or septuagenarian* or pensioner* or senile):ti,ab,kw	Limits	11577
#17	Senior*:ti,ab,kw	Limits	4208
#18	Elder*:ti,ab,kw	Limits	52681
#19	[mh ^"Aged"]	Limits	212093
#20	[mh ^"Aged, 80 and over"]	Limits	54160
#21	{or #15-#20}	Limits	609295
#22	(Cognit* NEAR/3impair*):ti,ab,kw	Limits	82296
#23	(Mild neurocognitive disorder*):ti,ab,kw	Limits	206
#24	[mh ^"Cognitive dysfunction"]	Limits	1760
#25	(Major neurocognitive disorder*):ti,ab,kw	Limits	537
#26	Dement*:ti,ab,kw	Limits	14016
#27	[mh ^"Dementia"]	Limits	2498
#28	[mh ^"Alzheimer disease"]	Limits	3519
#29	Alzheimer*:ti,ab,kw	Limits	11682
#30	[mh ^"AIDS Dementia Complex"]	Limits	77
#31	[mh ^"Dementia, Vascular"]	Limits	293
#32	[mh ^"Dementia, Multi-Infarct"]	Limits	68
#33	[mh ^"Lewy body disease"]	Limits	93

#34	Deliri*:ti,ab,kw		Limits	3931
#35	[mh ^"Delirium"]		Limits	743
#36	{or #22-#35}		Limits	95436
#37	#14 and #21 and #36		Limits	1193
#38	Type a search term or use the S or MeSH buttons to	S MeSH	Limits	N/A

Clear all

Highlight orphan lines

Save this search | View saved searches | Search help

Cochrane Search_TCP Systematic Review_July 15, 2021

Last saved on: 15/07/2021 18:46:23

Search saved.

View fewer lines | Print

Filter your results

Cochrane Reviews	Cochrane Protocols	Trials	Editorials	Special Collections	Clinical Answers	More
20	0	1172	1	0	0	

20 Cochrane Reviews matching **"#37 - #14 and #21 and #36"**

Cochrane Database of Systematic Reviews
Issue 7 of 12, July 2021

Select all (20) | Export selected citation(s) | **Show all previews**

Order by Relevancy | Results per page 25

1

Special care units for dementia individuals with behavioural problems

Claudia KY Lai, Jonas HM Yeung, Vincent Mok, Iris Chi

Intervention Review 7 October 2009

Show PICOs BETA **Show preview**

2

Benzodiazepines for treatment of patients with delirium excluding those who are cared for in an intensive care unit

Yan Li, Jun Ma, Yinghui Jin, Nan Li, Rui Zheng, Wei Mu, Jiaying Wang, Jin Hua Si, Jing Chen, Hong Cai Shang

Intervention Review 28 February 2020 Free access

Show PICOs BETA **Show preview**

3

Interventions for preventing and reducing the use of physical restraints in long-term geriatric care

Ralph Möhler, Tanja Richter, Sascha Köpke, Gabriele Meyer

Intervention Review 16 February 2011

Show PICOs BETA **Show preview**

4

Oral care measures for preventing nursing home-acquired pneumonia

Chang Liu, Yubin Cao, Jie Lin, Linda Ng, Ian Needleman, Tanya Walsh, Chunjie Li

Intervention Review 27 September 2018 Free access

Show PICOs BETA **Show preview**

5

Withdrawal versus continuation of long-term antipsychotic drug use for behavioural and psychological symptoms in older people with dementia

Ellen Van Leeuwen, Mirko Petrovic, Mieke L van Driel, An IM De Sutter, Robert Vander Stichele, Tom Declercq, Thierry Christiaens

Intervention Review 30 March 2018 New search Free access

Show PICOs BETA **Show preview**

6

Dance therapy for schizophrenia

Juanjuan Ren, Jun Xia

Intervention Review 4 October 2013 New search Free access

Show PICOs BETA **Show preview**

7

Montreal Cognitive Assessment for the detection of dementia

Daniel HJ Davis, Samuel T Creavin, Jennifer LY Yip, Anna H Noel-Storr, Carol Brayne, Sarah Cullum

Diagnostic Review 13 July 2021

Show preview

8

Comprehensive geriatric assessment for older people admitted to a surgical service

Gilgamesh Eamer, Amir Taheri, Sidian S Chen, Quinn Daviduck, Thane Chambers, Xinzhe Shi, Rachel G Khadaroo

Intervention Review 31 January 2018 Free access

Show PICOs BETA Show preview

9

Habit retraining for the management of urinary incontinence in adults

Joan Ostaszkiewicz, Tracey Chestney, Brenda Roe

Intervention Review 19 April 2004 New search

Show preview

10

Interventions to increase patient and family involvement in escalation of care for acute life-threatening illness in community health and hospital settings

Nicola J Mackintosh, Rachel E Davis, Abigail Easter, Hannah Rayment-Jones, Nick Sevdalis, Sophie Wilson, Mary Adams, Jane Sandall

Intervention Review 8 December 2020

Show PICOs BETA Show preview

11

AD-8 for detection of dementia across a variety of healthcare settings

Kirsty Hendry, Claire Green, Rupert McShane, Anna H Noel-Storr, David J Stott, Sumayya Anwer, Alex J Sutton, Jennifer K Burton, Terry J Quinn

Diagnostic Review 4 March 2019 Free access

Show preview

12

Comprehensive geriatric assessment for older adults admitted to hospital

Graham Ellis, Mike Gardner, Apostolos Tsiachristas, Peter Langhorne, Orlaith Burke, Rowan H Harwood, Simon P Conroy, Tilo Kircher, Dominique Somme, Ingvild Saltvedt, Heidi Wald, Desmond O'Neill, David Robinson, Sasha Shepperd

Intervention Review 12 September 2017 New search Free access

Show PICOs BETA Show preview

13

Multi-disciplinary rehabilitation for acquired brain injury in adults of working age

Lynne Turner-Stokes, Anton Pick, Ajoy Nair, Peter B Disler, Derick T Wade

Intervention Review 22 December 2015 New search Conclusions changed Free access

Show PICOs BETA **Show preview**

14

'As required' medication regimens for seriously mentally ill people in hospital

Petrina Douglas-Hall, Emma V Whicher

Intervention Review 21 December 2015 New search Free access

Show PICOs BETA **Show preview**

15

Rehabilitation interventions for improving physical and psychosocial functioning after hip fracture in older people

Maria Crotty, Kathleen Unroe, Ian D Cameron, Michelle Miller, Gilbert Ramirez, Leah Couzner

Intervention Review 20 January 2010

Show preview

16

Informant Questionnaire on Cognitive Decline in the Elderly (IQCODE) for the diagnosis of dementia within a secondary care setting

Jennifer K Harrison, Patricia Fearon, Anna H Noel-Storr, Rupert McShane, David J Stott, Terry J Quinn

Diagnostic Review 10 March 2015 Free access

Show preview

17

Physical fitness training for stroke patients

David H Saunders, Mark Sanderson, Sara Hayes, Liam Johnson, Sharon Kramer, Daniel D Carter, Hannah Jarvis, Miriam Brazzelli, Gillian E Mead

Intervention Review 20 March 2020 New search Conclusions changed Free access

Show PICOs BETA **Show preview**

18

Reminiscence therapy for dementia

Bob Woods, Laura O'Philbin, Emma M Farrell, Aimee E Spector, Martin Orrell

Intervention Review 1 March 2018 New search Conclusions changed Free access

Show PICOs BETA **Show preview**

19

Psychological therapies for the treatment of mental disorders in low- and middle-income countries affected by humanitarian crises

Marianna Purgato, Chiara Gastaldon, Davide Papola, Mark van Ommeren, Corrado Barbui, Wietse A Tol

Intervention Review 5 July 2018 Free access

Show PICOs BETA **Show preview**

20

Cough augmentation techniques for extubation or weaning critically ill patients from mechanical ventilation

Louise Rose, Neill KJ Adhikari, David Leasa, Dean A Fergusson, Douglas McKim

Intervention Review 11 January 2017 Free access

Show PICOs BETA **Show preview**

Appendix A: OVID Medline Search Strategy

Ovid MEDLINE: Epub Ahead of Print, In-Process & Other Non-Indexed Citations, Ovid MEDLINE® Daily and Ovid MEDLINE® 1946-Present

Search done on: July 9, 2022

Search dates: Publication Year 2021-2022

1. (Transition* adj3 (care* or unit* or bed* or program* or ward* or setting* or facilit* or service* or model* or centre* or center*)).tw,kf.
2. Transitional care/
3. (Intermediate adj3 (unit* or care or bed* or program* or ward* or setting* or facilit* or service* or model* or centre* or center*)).tw,kf.
4. Intermediate care facilities/
5. ((Subacute or sub-acute) adj3 (unit* or care or bed* or program* or ward* or setting* or facilit* or service* or model* or centre* or center*)).tw,kf.
6. Subacute care/
7. ((Postacute or post-acute) adj3 (unit* or care or bed* or program* or ward* or setting* or facilit* or service* or model* or centre* or center*)).tw,kf.
8. ((Post acute) adj3 (unit* or care or bed* or program* or ward* or setting* or facilit* or service* or model* or centre* or center*)).tw,kf.
9. ((Skilled Nursing) adj3 (unit* or bed* or program* or ward* or setting* or facilit* or service* or model* or centre* or center*)).tw,kf.
10. Skilled nursing facilities/
11. (Restor* adj3 (unit* or care or bed* or program* or ward* or setting* or facilit* or service* or model* or centre* or center*)).tw,kf.
12. (Convalesc* adj3 (unit* or care or bed* or program* or ward* or setting* or facilit* or service* or model* or centre* or center* or home* or hospital*)).tw,kf.
13. Convalescence/
14. or/1-13
15. (Old* or aged or aging).tw,kf.
16. (Centenarian* or nonagenarian* or octogenarian* or geriatr* or gerontol* or senescen* or septuagenarian* or pensioner* or senile).tw,kf.
17. Senior*.tw,kf.
18. Elder*.tw,kf.
19. Aged/
20. "Aged, 80 and over"/
21. or/15-20
22. (Cognit* adj3 impair*).tw,kf.
23. (Mild neurocognitive disorder*).tw,kf.
24. Cognitive dysfunction/
25. (Major neurocognitive disorder*).tw,kf.
26. Dement*.tw,kf.

27. Dementia/
28. Alzheimer disease/
29. Alzheimer*.tw,kf.
30. AIDS Dementia Complex/
31. Dementia, Vascular/
32. Dementia, Multi-Infarct/
33. Lewy body disease/
34. Deliri*.tw,kf.
35. Delirium/
36. or/22-35
37. 14 and 21 and 36
38. limit 37 to yr="2021 - 2022"
    Result: 174 articles

Appendix B: OVID Embase Search Strategy

Embase Classic+Embase 1947 to 2022 July 08

Search done on: July 9, 2022

1. (Transition* adj3 (care* or unit* or bed* or program* or ward* or setting* or facilit* or service* or model* or centre* or center*)).tw,kw.
2. Transitional care/
3. (Intermediate adj3 (unit* or care or bed* or program* or ward* or setting* or facilit* or service* or model* or centre* or center*)).tw,kw.
4. Nursing home/
5. ((Subacute or sub-acute) adj3 (unit* or care or bed* or program* or ward* or setting* or facilit* or service* or model* or centre* or center*)).tw,kw.
6. Subacute care/
7. ((Postacute or post-acute) adj3 (unit* or care or bed* or program* or ward* or setting* or facilit* or service* or model* or centre* or center*)).tw,kw.
8. ((Post acute) adj3 (unit* or care or bed* or program* or ward* or setting* or facilit* or service* or model* or centre* or center*)).tw,kw.
9. ((Skilled Nursing) adj3 (unit* or bed* or program* or ward* or setting* or facilit* or service* or model* or centre* or center*)).tw,kw.
10. (Restor* adj3 (unit* or care or bed* or program* or ward* or setting* or facilit* or service* or model* or centre* or center*)).tw,kw.
11. (Convalesc* adj3 (unit* or care or bed* or program* or ward* or setting* or facilit* or service* or model* or centre* or center* or home* or hospital*)).tw,kw.
12. Convalescence/
13. or/1-12
14. (Old* or aged or aging).tw,kw.
15. (Centenarian* or nonagenarian* or octogenarian* or geriatr* or gerontol* or senescen* or septuagenarian* or pensioner* or senile).tw,kw.
16. Senior*.tw,kw.
17. Elder*.tw,kw.
18. Aged/
19. Very Elderly/
20. or/14-19
21. (Cognit* adj3 impair*).tw,kw.
22. (Mild neurocognitive disorder*).tw,kw.
23. Cognitive deficit/
24. (Major neurocognitive disorder*).tw,kw.
25. Dement*.tw,kw.
26. Dementia/
27. Alzheimer disease/
28. Alzheimer*.tw,kw.
29. Frontotemporal dementia/

30. Frontal variant frontotemporal dementia/
31. HIV associated dementia/
32. "Mixed depression and dementia"/
33. Multiinfarct dementia/
34. Pick presenile dementia/
35. Presenile dementia/
36. Senile dementia/
37. Diffuse Lewy body disease/
38. Deliri*.tw,kw.
39. Delirium/
40. or/21-39
41. 13 and 20 and 40
42. limit 41 to yr="2021 - 2022"
    Result: 1064 articles

Appendix C: OVID APA PsycInfo Search Strategy

APA PsycInfo 1806 to July Week 1 2022

Search done on: July 9, 2022

1. (Transition* adj3 (care* or unit* or bed* or program* or ward* or setting* or facilit* or service* or model* or centre* or center*)).tw
2. (Intermediate adj3 (unit* or care or bed* or program* or ward* or setting* or facilit* or service* or model* or centre* or center*)).tw
3. ((Subacute or sub-acute) adj3 (unit* or care or bed* or program* or ward* or setting* or facilit* or service* or model* or centre* or center*)).tw
4. ((Postacute or post-acute) adj3 (unit* or care or bed* or program* or ward* or setting* or facilit* or service* or model* or centre* or center*)).tw
5. ((Post acute) adj3 (unit* or care or bed* or program* or ward* or setting* or facilit* or service* or model* or centre* or center*)).tw
6. ((Skilled Nursing) adj3 (unit* or bed* or program* or ward* or setting* or facilit* or service* or model* or centre* or center*)).tw
7. (Restor* adj3 (unit* or care or bed* or program* or ward* or setting* or facilit* or service* or model* or centre* or center*)).tw
8. (Convalesc* adj3 (unit* or care or bed* or program* or ward* or setting* or facilit* or service* or model* or centre* or center* or home* or hospital*)).tw
9. or/1-8
10. (Old* or aged or aging).tw
11. (Centenarian* or nonagenarian* or octogenarian* or geriatr* or gerontol* or senescen* or septuagenarian* or pensioner* or senile).tw
12. Senior*.tw
13. Elder*.tw
14. Geriatric patients/
15. or/10-14
16. (Cognit* adj3 impair*).tw
17. (Mild neurocognitive disorder*).tw
18. Cognitive Impairment/
19. Mild Cognitive Impairment/
20. (Major neurocognitive disorder*).tw
21. Dement*.tw
22. Dementia/
23. "Alzheimer's Disease"/
24. Alzheimer*.tw
25. AIDS Dementia Complex/
26. Dementia with Lewy Bodies/
27. Presenile Dementia/
28. Senile Dementia/
29. Semantic Dementia/

30. Vascular Dementia/
31. Deliri*.tw
32. Delirium/
33. or/16-32
34. 9 and 15 and 33
35. limit 34 to yr="2021 - 2022"
    Result: 25 articles

Appendix D: CINAHL Plus Search Strategy

CINAHL Plus with Full Text

Search done on: July 9, 2022

Search dates: 20210701-20220731

TI or AB:

1. (Transition* N3 (care* or unit* or bed* or program* or ward* or setting* or facilit* or service* or model* or centre* or center*))
2. MH Transitional Care
3. (Intermediate N3 (unit* or care or bed* or program* or ward* or setting* or facilit* or service* or model* or centre* or center*))
4. ((Subacute or sub-acute) N3 (unit* or care or bed* or program* or ward* or setting* or facilit* or service* or model* or centre* or center*))
5. MH Subacute care
6. ((Postacute or post-acute) N3 (unit* or care or bed* or program* or ward* or setting* or facilit* or service* or model* or centre* or center*))
7. ((Post acute) N3 (unit* or care or bed* or program* or ward* or setting* or facilit* or service* or model* or centre* or center*))
8. ((Skilled Nursing) N3 (unit* or bed* or program* or ward* or setting* or facilit* or service* or model* or centre* or center*))
9. MH Skilled Nursing Facilities
10. (Restor* N3 (unit* or care or bed* or program* or ward* or setting* or facilit* or service* or model* or centre* or center*))
11. (Convalesc* N3 (unit* or care or bed* or program* or ward* or setting* or facilit* or service* or model* or centre* or center* or home* or hospital*))
12. S1 OR S2 OR S3 OR S4 OR S5 OR S6 OR S7 OR S8 OR S9 OR S10 OR S11
13. (Old* or aged or aging)
14. (Centenarian* or nonagenarian* or octogenarian* or geriatr* or gerontol* or senescen* or septuagenarian* or pensioner* or senile)
15. Senior*
16. Elder*
17. MH Aged
18. MH "Aged, 80 and over"
19. S13 OR S14 OR S15 OR S16 OR S17 OR S18
20. (Cognit* N3 impair*)
21. (Mild neurocognitive disorder*)
22. MH "Mild Cognitive Impairment"
23. (Major neurocognitive disorder*)
24. Dement*
25. MH Dementia
26. MH "Alzheimer’s disease"

27. Alzheimer*
28. MH "AIDS Dementia Complex"
29. MH "Dementia, Vascular"
30. MH "Dementia, Multi-Infarct"
31. MH "Lewy Body Disease"
32. MH "Dementia, Presenile"
33. MH "Dementia, Senile"
34. Deliri*
35. MH Delirium
36. S20 OR S21 OR S22 OR S23 OR S24 OR S25 OR S26 OR S27 OR S28 OR S29 OR S30 OR S31 OR S32 OR S33 OR S34 OR S35
37. S12 AND S19 AND S36
    Result: 925 articles
38. Limiters for search 37: Published Date: 20210701-20220731
    Result: 83 articles

Appendix E: Cochrane Search Strategy

Cochrane Central Register of Controlled Trials

Issue 7 of 12, June 2022

Search done on: July 9, 2022

Search dates: with Cochrane Library publication date from Jul 2021 to Jul 2022

1. (Transition* NEAR/3 (care* or unit* or bed* or program* or ward* or setting* or facilit* or service* or model* or centre* or center*)):ti,ab,kw
2. [mh ^"Transitional care"]
3. (Intermediate NEAR/3 (unit* or care or bed* or program* or ward* or setting* or facilit* or service* or model* or centre* or center*)):ti,ab,kw
4. [mh ^"Intermediate care facilities"]
5. ((Subacute or sub-acute) NEAR/3 (unit* or care or bed* or program* or ward* or setting* or facilit* or service* or model* or centre* or center*)):ti,ab,kw
6. [mh ^"Subacute care"]
7. ((Postacute or post-acute) NEAR/3 (unit* or care or bed* or program* or ward* or setting* or facilit* or service* or model* or centre* or center*)):ti,ab,kw
8. ((Post acute) NEAR/3 (unit* or care or bed* or program* or ward* or setting* or facilit* or service* or model* or centre* or center*)):ti,ab,kw
9. ((Skilled Nursing) NEAR/3 (unit* or bed* or program* or ward* or setting* or facilit* or service* or model* or centre* or center*)):ti,ab,kw
10. [mh ^"Skilled nursing facilities"]
11. (Restor* NEAR/3 (unit* or care or bed* or program* or ward* or setting* or facilit* or service* or model* or centre* or center*)):ti,ab,kw
12. (Convalesc* NEAR/3 (unit* or care or bed* or program* or ward* or setting* or facilit* or service* or model* or centre* or center* or home* or hospital*)):ti,ab,kw
13. [mh ^"Convalescence"]
14. {or #1-#13}
15. (Old* or aged or aging):ti,ab,kw
16. (Centenarian* or nonagenarian* or octogenarian* or geriatr* or gerontol* or senescen* or septuagenarian* or pensioner* or senile):ti,ab,kw
17. Senior*:ti,ab,kw
18. Elder*:ti,ab,kw
19. [mh ^"Aged"]
20. [mh ^"Aged, 80 and over"]
21. {or #15-#20}
22. (Cognit* NEAR/3impair*):ti,ab,kw
23. (Mild neurocognitive disorder*):ti,ab,kw
24. [mh ^"Cognitive dysfunction"]
25. (Major neurocognitive disorder*):ti,ab,kw
26. Dement*:ti,ab,kw

27. [mh ^"Dementia"]
28. [mh ^"Alzheimer disease"]
29. Alzheimer*:ti,ab,kw
30. [mh ^"AIDS Dementia Complex"]
31. [mh ^"Dementia, Vascular"]
32. [mh ^"Dementia, Multi-Infarct"]
33. [mh ^"Lewy body disease"]
34. Deliri*:ti,ab,kw
35. [mh ^"Delirium"]
36. {or #22-#35}
37. #14 and #21 and #36
    Result: 1322 articles
38. #14 and #21 and #36 with Cochrane Library publication date from Jul 2021 to Jul 2022
    Result: 121 articles

Search History saved as "OVID MEDLINE TCP Systematic Review July 15 2021_Updated on July 9 2022"

▼ Search History (38)

View Saved

# ▲	Searches	Results	Type	Actions	Annotations
1	(Transition* adj3 (care* or unit* or bed* or program* or ward* or setting* or facilit* or service* or model* or centre* or center*)).tw,kf.	23451	Advanced	Display Results | More	
2	Transitional care/	1151	Advanced	Display Results | More	
3	(Intermediate adj3 (unit* or care or bed* or program* or ward* or setting* or facilit* or service* or model* or centre* or center*)).tw,kf.	5240	Advanced	Display Results | More	
4	Intermediate care facilities/	716	Advanced	Display Results | More	
5	((Subacute or sub-acute) adj3 (unit* or care or bed* or program* or ward* or setting* or facilit* or service* or model* or centre* or center*)).tw,kf.	1738	Advanced	Display Results | More	
6	Subacute care/	1345	Advanced	Display Results | More	
7	((Postacute or post-acute) adj3 (unit* or care or bed* or program* or ward* or setting* or facilit* or service* or model* or centre* or center*)).tw,kf.	2780	Advanced	Display Results | More	
8	(Post acute adj3 (unit* or care or bed* or program* or ward* or setting* or facilit* or service* or model* or centre* or center*)).tw,kf.	1816	Advanced	Display Results | More	
9	(Skilled Nursing adj3 (unit* or bed* or program* or ward* or setting* or facilit* or service* or model* or centre* or center*)).tw,kf.	3573	Advanced	Display Results | More	
10	Skilled nursing facilities/	5032	Advanced	Display Results | More	
11	(Restor* adj3 (unit* or care or bed* or program* or ward* or setting* or facilit* or service* or model* or centre* or center*)).tw,kf.	5275	Advanced	Display Results | More	
12	(Convalesc* adj3 (unit* or care or bed* or program* or ward* or setting* or facilit* or service* or model* or centre* or center* or home* or hospital*)).tw,kf.	1275	Advanced	Display Results | More	
13	Convalescence/	3921	Advanced	Display Results | More	
14	or/1-13	49762	Advanced	Display Results | More	
15	(Old* or aged or aging).tw,kf.	2296417	Advanced	Display Results | More	
16	(Centenarian* or nonagenarian* or octogenarian* or geriatr* or gerontol* or senescen* or septuagenarian* or pensioner* or senile).tw,kf.	153432	Advanced	Display Results | More	
17	Senior*.tw,kf.	48034	Advanced	Display Results | More	
18	Elder*.tw,kf.	293714	Advanced	Display Results | More	
19	Aged/	3357394	Advanced	Display Results | More	
20	"Aged, 80 and over"/	1007454	Advanced	Display Results | More	
21	or/15-20	5219333	Advanced	Display Results | More	
22	(Cognit* adj3 impair*).tw,kf.	97256	Advanced	Display Results | More	
23	Mild neurocognitive disorder*.tw,kf.	223	Advanced	Display Results | More	
24	Cognitive dysfunction/	30811	Advanced	Display Results | More	
25	Major neurocognitive disorder*.tw,kf.	262	Advanced	Display Results | More	
26	Dement*.tw,kf.	136770	Advanced	Display Results | More	
27	Dementia/	58693	Advanced	Display Results | More	
28	Alzheimer disease/	110857	Advanced	Display Results | More	
29	Alzheimer*.tw,kf.	174315	Advanced	Display Results | More	
30	AIDS Dementia Complex/	3907	Advanced	Display Results | More	
31	Dementia, Vascular/	5410	Advanced	Display Results | More	
32	Dementia, Multi-Infarct/	1090	Advanced	Display Results | More	
33	Lewy body disease/	4024	Advanced	Display Results | More	
34	Deliri*.tw,kf.	19570	Advanced	Display Results | More	
35	Delirium/	11366	Advanced	Display Results | More	
36	or/22-35	359590	Advanced	Display Results | More	
37	14 and 21 and 36	1204	Advanced	Display Results | More	
38	limit 37 to yr="2021 - 2022"	174	Advanced	Display Results | More	

Contract

Save | Remove | Combine with: AND | OR

Save All | Edit | Create RSS | Create Auto-Alert | View Saved

Email All Search History | Copy Search History Link | Copy Search History Details

Advanced Search | Basic Search | Find Citation | Search Tools | Search Fields | Multi-Field Search

1 Resource selected | Hide | Change

Ovid MEDLINE: Epub Ahead of Print, In-Process & Other Non-Indexed Citations, Ovid MEDLINE® Daily and Ovid MEDLINE® 1946-Present

Enter keyword or phrase (* or $ for truncation) ◉ **Keyword** ○ Author ○ Title ○ Journal

Search | Expand Term Finder

▾ **Limits** *(close)* ☐ Include Multimedia ☑ Map Term to Subject Heading

☐ English Language ☐ Humans

Publication Year - | -

Age Groups
- -
- All Infant (birth to 23 months)
- All Child (0 to 18 years)
- All Adult (19 plus years)
- Newborn Infant (birth to 1 month)
- Infant (1 to 23 months)

Publication Types
- -
- Adaptive Clinical Trial
- Address
- Autobiography
- Bibliography
- Biography

Clinical Queries
- -
- Reviews (maximizes sensitivity)
- Reviews (maximizes specificity)
- Reviews (best balance of sensitivity and specificity)
- Therapy (maximizes sensitivity)
- Therapy (maximizes specificity)

Additional Limits | Edit Limits

View By
Text (174 Results)
Multimedia (0 Results)

Search Information

You searched:
limit 37 to yr="2021 - 2022"

Search terms used:
80
aged
aged,
and
over
aging
aids
dementia
complex
alzheimer
disease
alzheimer*
bed*
care
care*
centenarian*
center*
centre*
cognit*
cognitive
dysfunction
convalesc*
convalescence
deliri*
delirium
dement*
dementia,
multi-infarct
vascular
elder*
facilit*
geriatr*
gerontol*
home*
hospital*
impair*
intermediate
facilities
lewy
body
major
neurocognitive
disorder*
mild
model*
nonagenarian*
octogenarian*
old*
pensioner*
post
acute
post-acute
postacute
program*
restor*
senescen*
senile
senior*
septuagenarian*
service*
setting*
skilled
nursing
sub-acute
subacute
transition*
transitional
unit*
ward*

Search Returned:
174 text results

Sort By:
-

Customize Display

Filter By
Add to Search History
Selected Only (0)
Years
All Years
Current year
Past 3 years
Past 5 years
Specific Year Range
Subject
Author
Journal
Book
Publication Type

My Projects
+ New Project

101.

Unique Identifier	34047895
Title	Ageing- and dementia-friendly design: theory and evidence from cognitive psychology, neuropsychology and environmental psychology can contribute to design guidelines that minimise spatial disorientation.
Source	Cognitive Processing. 22(4):715-730, 2021 Nov.
Authors	Wiener JM; Pazzaglia F
Authors Full Name	Wiener, Jan M; Pazzaglia, Francesca.
Publication Type	Journal Article.

Abstract Reference | Complete Reference | Find Similar | Find Citing Articles | Get it UTL

Cite + My Projects + Annotate

102.

Unique Identifier	33769114
Title	Challenges to Admitting Residents: Perspectives from Rural Nursing Home Administrators and Staff.
Source	Inquiry. 58:469580211005191, 2021 Jan-Dec.
Authors	Henning-Smith C; Cross D; Rahman A
Authors Full Name	Henning-Smith, Carrie; Cross, Dori; Rahman, Adrita.
Publication Type	Journal Article. Research Support, Non-U.S. Gov't. Research Support, U.S. Gov't, P.H.S..

Abstract Reference | Complete Reference | Find Similar | Find Citing Articles | Get it UTL

Cite + My Projects + Annotate

103.

Unique Identifier	34663289
Title	Performance of quantitative measures of multimorbidity: a population-based retrospective analysis.
Source	BMC Public Health. 21(1):1881, 2021 10 18.
Authors	Vela E; Cleries M; Monterde D; Carot-Sans G; Coca M; Valero-Bover D; Piera-Jimenez J; Garcia Eroles L; Perez Sust P
Authors Full Name	Vela, Emili; Cleries, Montse; Monterde, David; Carot-Sans, Gerard; Coca, Marc; Valero-Bover, Damia; Piera-Jimenez, Jordi; Garcia Eroles, Luis; Perez Sust, Pol.
Publication Type	Journal Article.

Abstract Reference | Complete Reference | Find Similar | Find Citing Articles | Full Text

Cite + My Projects + Annotate

104.

Unique Identifier	34639409
Title	Eating Difficulties among Older Adults with Dementia in Long-Term Care Facilities: A Scoping Review. [Review]
Source	International Journal of Environmental Research & Public Health [Electronic Resource]. 18(19), 2021 09 26.
Authors	Jung D; Lee K; De Gagne JC; Lee M; Lee H; Yoo L; Won S; Choi E
Authors Full Name	Jung, Dukyoo; Lee, Kyuri; De Gagne, Jennie C; Lee, Minkyung; Lee, Hyesoon; Yoo, Leeho; Won, Sarah; Choi, Eunju.
Publication Type	Journal Article. Research Support, Non-U.S. Gov't. Review.

Abstract Reference | Complete Reference | Find Similar | Find Citing Articles | Get it UTL

Cite + My Projects + Annotate

105.

Unique Identifier	34578901
Title	Does a 12-Month Transitional Care Model Intervention by Geriatric-Experienced Care Professionals Improve Nutritional Status of Older Patients after Hospital Discharge? A Randomized Controlled Trial.
Source	Nutrients. 13(9), 2021 Aug 29.
Authors	Chareh N; Rappl A; Rimmele M; Wingenfeld K; Freiberger E; Sieber CC; Volkert D
Authors Full Name	Chareh, Neshat; Rappl, Anja; Rimmele, Martina; Wingenfeld, Klaus; Freiberger, Ellen; Sieber, Cornel C; Volkert, Dorothee.
Publication Type	Journal Article. Randomized Controlled Trial.

Abstract Reference | Complete Reference | Find Similar | Find Citing Articles | Get it UTL

Cite + My Projects + Annotate

106.

Unique Identifier	34551841
Title	Delirium occurrence and association with outcomes in hospitalized COVID-19 patients.
Source	International Psychogeriatrics. 33(10):1105-1109, 2021 10.
Authors	Pagali S; Fu S; Lindroth H; Sohn S; Burton MC; Lapid M
Authors Full Name	Pagali, Sandeep; Fu, Sunyang; Lindroth, Heidi; Sohn, Sunghwan; Burton, M Caroline; Lapid, Maria.
Publication Type	Journal Article.

Abstract Reference | Complete Reference | Find Similar | Find Citing Articles | Get it UTL

Cite + My Projects + Annotate

107.

Unique Identifier	34493523
Title	Protocol for conducting scoping reviews to map implementation strategies in different care settings: focusing on evidence-based interventions for preselected phenomena in people with dementia.
Source	BMJ Open. 11(9):e051611, 2021 09 07.
Authors	Manietta C; Quasdorf T; Rommerskirch-Manietta M; Braunwarth JI; Purwins D; Roes M
Authors Full Name	Manietta, Christina; Quasdorf, Tina; Rommerskirch-Manietta, Mike; Braunwarth, Jana Isabelle; Purwins, Daniel; Roes, Martina.
Publication Type	Journal Article. Research Support, Non-U.S. Gov't.

Abstract Reference | Complete Reference | Find Similar | Find Citing Articles | Full Text

Cite + My Projects + Annotate

JBI EBP Tools

SUMARI

108.

Unique Identifier	34468334	Abstract Reference<br>Complete Reference
Title	Use of psychotropic drugs and drugs with anticholinergic properties among residents with dementia in intermediate care facilities for older adults in Japan: a cohort study.	Find Similar<br>Find Citing Articles
Source	BMJ Open. 11(4):e045787, 2021 04 08.	
Authors	Hamada S; Kojima T; Hattori Y; Maruoka H; Ishii S; Okochi J; Akishita M	Full Text
Authors Full Name	Hamada, Shota; Kojima, Taro; Hattori, Yukari; Maruoka, Hiroshi; Ishii, Shinya; Okochi, Jiro; Akishita, Masahiro.	
Publication Type	Journal Article. Research Support, Non-U.S. Gov't.	

Cite + My Projects + Annotate

109.

Unique Identifier	34419032	Abstract Reference<br>Complete Reference
Title	CO-FLOW: COvid-19 Follow-up care paths and Long-term Outcomes Within the Dutch health care system: study protocol of a multicenter prospective cohort study following patients 2 years after hospital discharge.	Find Similar<br>Find Citing Articles
Source	BMC Health Services Research. 21(1):847, 2021 Aug 21.	
Authors	Bek LM; Berentschot JC; Hellemons ME; Huijts SM; Aerts JGJV; van Bommel J; van Genderen ME; Gommers DAMPJ; Ribbers GM; Heijenbrok-Kal MH; van den Berg-Emons RJG; CO-FLOW Collaboration Group	Full Text
Authors Full Name	Bek, L Martine; Berentschot, Julia C; Hellemons, Merel E; Huijts, Susanne M; Aerts, Joachim G J V; van Bommel, Jasper; van Genderen, Michel E; Gommers, Diederik A M P J; Ribbers, Gerard M; Heijenbrok-Kal, Majanka H; van den Berg-Emons, Rita J G; CO-FLOW Collaboration Group.	
Publication Type	Journal Article.	

Cite + My Projects + Annotate

110.

Unique Identifier	34418841	Abstract Reference<br>Complete Reference
Title	Entry and Passage Variables Associated with Nursing Home Adjustment in Older Adults with Dementia.	Find Similar<br>Find Citing Articles
Source	Geriatric Nursing. 42(5):1084-1092, 2021 Sep-Oct.	
Authors	Wareing S; Sethares KA; Chin E; Ayotte B	
Authors Full Name	Wareing, Susan; Sethares, Kristen A; Chin, Elizabeth; Ayotte, Brian.	Get it UTL
Publication Type	Journal Article.	

Cite + My Projects + Annotate

111.

Unique Identifier	34379638	Abstract Reference<br>Complete Reference
Title	A large-scale brain network mechanism for increased seizure propensity in Alzheimer's disease.	Find Similar<br>Find Citing Articles
Source	PLoS Computational Biology. 17(8):e1009252, 2021 08.	
Authors	Tait L; Lopes MA; Stothart G; Baker J; Kazanina N; Zhang J; Goodfellow M	
Authors Full Name	Tait, Luke; Lopes, Marinho A; Stothart, George; Baker, John; Kazanina, Nina; Zhang, Jiaxiang; Goodfellow, Marc.	Full Text
Publication Type	Journal Article. Research Support, Non-U.S. Gov't.	

Cite + My Projects + Annotate

112.

Unique Identifier	34352202	Abstract Reference<br>Complete Reference
Title	Association Between Dementia Care Programs in Assisted Living Facilities and Transitions to Nursing Homes in Ontario, Canada: A Population-Based Cohort Study.	Find Similar<br>Find Citing Articles
Source	Journal of the American Medical Directors Association. 22(10):2115-2120.e6, 2021 10.	
Authors	Manis DR; Rahim A; Poss JW; Bielska IA; Bronskill SE; Tarride JE; Abelson J; Costa AP	Get it UTL
Authors Full Name	Manis, Derek R; Rahim, Ahmad; Poss, Jeffrey W; Bielska, Iwona A; Bronskill, Susan E; Tarride, Jean-Eric; Abelson, Julia; Costa, Andrew P.	
Publication Type	Journal Article. Research Support, Non-U.S. Gov't.	

Cite + My Projects + Annotate

113.

Unique Identifier	34307282	Abstract Reference<br>Complete Reference
Title	Identifying the Association Between Older Adults' Characteristics and Their Health-Related Outcomes in a Transition Care Setting: A Retrospective Audit.	Find Similar<br>Find Citing Articles
Source	Frontiers in Public Health. 9:688640, 2021.	
Authors	Hang JA; Francis-Coad J; Naseri C; Jacques A; Waldron N; Purslowe K; Hill AM	Get it UTL
Authors Full Name	Hang, Jo-Aine; Francis-Coad, Jacqueline; Naseri, Chiara; Jacques, Angela; Waldron, Nicholas; Purslowe, Kate; Hill, Anne-Marie.	
Publication Type	Journal Article. Research Support, Non-U.S. Gov't.	

Cite + My Projects + Annotate

114.

Unique Identifier	34212470	Abstract Reference<br>Complete Reference
Title	Toward the development of a vibrant, super-aged society: The future of medicine and society in Japan.	Find Similar<br>Find Citing Articles
Source	Geriatrics & gerontology international. 21(8):601-613, 2021 Aug.	
Authors	Iijima K; Arai H; Akishita M; Endo T; Ogasawara K; Kashihara N; Hayashi YK; Yumura W; Yokode M; Ouchi Y	
Authors Full Name	Iijima, Katsuya; Arai, Hidenori; Akishita, Masahiro; Endo, Tamao; Ogasawara, Kouetsu; Kashihara, Naoki; Hayashi, Yukiko K; Yumura, Wako; Yokode, Masayuki; Ouchi, Yasuyoshi.	Full Text
Publication Type	Journal Article.	

Cite + My Projects + Annotate

115.

Abstract Reference
Complete Reference
Find Similar
Find Citing Articles
Full Text

Unique Identifier	34209673
Title	*C9orf72* Intermediate Repeats Confer Genetic Risk for Severe COVID-19 Pneumonia Independently of Age.
Source	International Journal of Molecular Sciences. 22(13), 2021 06 29.
Authors	Zanella I; Zacchi E; Piva S; Filosto M; Beligni G; Alaverdian D; Amitrano S; Fava F; Baldassarri M; Frullanti E; Meloni I; Renieri A; GEN-COVID Multicenter Study; GEVACOBA Study Group; Castelli F; Quiros-Roldan E
Authors Full Name	Zanella, Isabella; Zacchi, Eliana; Piva, Simone; Filosto, Massimiliano; Beligni, Giada; Alaverdian, Diana; Amitrano, Sara; Fava, Francesca; Baldassarri, Margherita; Frullanti, Elisa; Meloni, Ilaria; Renieri, Alessandra; GEN-COVID Multicenter Study; GEVACOBA Study Group; Castelli, Francesco; Quiros-Roldan, Eugenia.
Publication Type	Journal Article.

Cite + My Projects + Annotate

116.

Abstract Reference
Complete Reference
Find Similar
Find Citing Articles
Full Text

Unique Identifier	34187380
Title	Exploring life-space in the nursing home. An observational longitudinal study.
Source	BMC Geriatrics. 21(1):396, 2021 06 29.
Authors	Sverdrup K; Bergh S; Selbaek G; Benth JS; Husebo B; Roen IM; Thingstad P; Tangen GG
Authors Full Name	Sverdrup, Karen; Bergh, Sverre; Selbaek, Geir; Benth, Jurate Saltyte; Husebo, Bettina; Roen, Irene Mari; Thingstad, Pernille; Tangen, Gro Gujord.
Publication Type	Journal Article. Observational Study. Research Support, Non-U.S. Gov't.

Cite + My Projects + Annotate

117.

Abstract Reference
Complete Reference
Find Similar
Find Citing Articles
Full Text

Unique Identifier	34173231
Title	Outcomes of post-acute care in skilled nursing facilities in Medicare beneficiaries with and without a diagnosis of dementia.
Source	Journal of the American Geriatrics Society. 69(10):2899-2907, 2021 10.
Authors	Burke RE; Xu Y; Ritter AZ
Authors Full Name	Burke, Robert E; Xu, Yao; Ritter, Ashley Z.
Publication Type	Journal Article. Research Support, Non-U.S. Gov't. Research Support, U.S. Gov't, Non-P.H.S..

Cite + My Projects + Annotate

118.

Abstract Reference
Complete Reference
Find Similar
Find Citing Articles
Full Text

Unique Identifier	34137032
Title	Trajectories of functional performance recovery after inpatient geriatric rehabilitation: an observational study.
Title Comment	Comment in: Med J Aust. 2021 Aug 16;215(4):169-170<br>PMID: 34287924 [https://www-ncbi-nlm-nih-gov.myaccess.library.utoronto.ca/pub...]
Source	Medical Journal of Australia. 215(4):173-179, 2021 08 16.
Authors	Soh CH; Reijnierse EM; Tuttle C; Marston C; Goonan R; Lim WK; Maier AB
Authors Full Name	Soh, Cheng Hwee; Reijnierse, Esmee M; Tuttle, Camilla; Marston, Celia; Goonan, Rose; Lim, Wen Kwang; Maier, Andrea B.
Publication Type	Journal Article. Observational Study.

Cite + My Projects + Annotate

119.

Ovid Full Text
Abstract Reference
Complete Reference
Find Similar
Find Citing Articles

Unique Identifier	34131093
Title	Executive Function Moderates Functional Outcomes of Engagement Strategies During Rehabilitation in Older Adults.
Source	American Journal of Physical Medicine & Rehabilitation. 100(7):635-642, 2021 07 01.
Authors	Ercal B; Rodebaugh TL; Bland MD; Barco P; Lenard E; Lang CE; Miller JP; Yingling M; Lenze EJ
Authors Full Name	Ercal, Baris; Rodebaugh, Thomas L; Bland, Marghuretta D; Barco, Peggy; Lenard, Emily; Lang, Catherine E; Miller, J Philip; Yingling, Michael; Lenze, Eric J.
Publication Type	Journal Article. Randomized Controlled Trial. Research Support, N.I.H., Extramural.

Article as PDF (230KB) Cite + My Projects + Annotate

120.

Abstract Reference
Complete Reference
Find Similar
Find Citing Articles
Full Text

Unique Identifier	34109693
Title	Analysis of discharge documentation for older adults living with dementia: A cohort study.
Source	Journal of Clinical Nursing. 30(23-24):3634-3643, 2021 Dec.
Authors	Parker KJ; Phillips JL; Luckett T; Agar M; Ferguson C; Hickman LD
Authors Full Name	Parker, Kirsten J; Phillips, Jane L; Luckett, Tim; Agar, Meera; Ferguson, Caleb; Hickman, Louise D.
Publication Type	Journal Article.

Cite + My Projects + Annotate

121.

Ovid Full Text
Abstract Reference
Complete Reference
Find Similar
Find Citing Articles

Unique Identifier	34108416
Title	High occurrence of postintensive care syndrome identified in surgical ICU survivors after implementation of a multidisciplinary clinic.
Source	The Journal of Trauma and Acute Care Surgery. 91(2):406-412, 2021 08 01.
Authors	Bottom-Tanzer SF; Poyant JO; Louzada MT; Ahmed SE; Boudouvas A; Poon E; Hojman HM; Bugaev N; Johnson BP; Van Kirk AL; Daniel E; Emoff C; Mahoney EJ
Authors Full Name	Bottom-Tanzer, Samantha F; Poyant, Janelle O; Louzada, Maria T; Ahmed, Sana E; Boudouvas, Abbey; Poon, Eileen; Hojman, Horacio M; Bugaev, Nikolay; Johnson, Benjamin P; Van Kirk, Anna Lisa; Daniel, Eryn; Emoff, Caroline; Mahoney, Eric J.
Publication Type	Journal Article. Observational Study. Research Support, N.I.H., Extramural. Research Support, Non-U.S. Gov't.

Article as PDF (1273KB) Cite + My Projects + Annotate

☐ 122.

Unique Identifier	34081627
Title	*Cistanche deserticola* polysaccharides alleviate cognitive decline in aging model mice by restoring the gut microbiota-brain axis.
Source	Aging. 13(11):15320-15335, 2021 06 03.
Authors	Gao Y; Li B; Liu H; Tian Y; Gu C; Du X; Bu R; Gao J; Liu Y; Li G
Authors Full Name	Gao, Yuan; Li, Bing; Liu, Hong; Tian, Yajuan; Gu, Chao; Du, Xiaoli; Bu, Ren; Gao, Jie; Liu, Yang; Li, Gang.
Publication Type	Journal Article. Research Support, Non-U.S. Gov't.

Abstract Reference
Complete Reference
Find Similar
Find Citing Articles
Full Text

Cite + My Projects + Annotate

☐ 123.

Unique Identifier	34079240
Title	Dementia Enlightened?! A Systematic Literature Review of the Influence of Indoor Environmental Light on the Health of Older Persons with Dementia in Long-Term Care Facilities.
Source	Clinical Interventions In Aging. 16:909-937, 2021.
Authors	Goudriaan I; van Boekel LC; Verbiest MEA; van Hoof J; Luijkx KG
Authors Full Name	Goudriaan, Ingrid; van Boekel, Leonieke C; Verbiest, Marjolein E A; van Hoof, Joost; Luijkx, Katrien G.
Publication Type	Journal Article. Systematic Review.

Abstract Reference
Complete Reference
Find Similar
Find Citing Articles
Get it UTL

Cite + My Projects + Annotate

☐ 124.

Unique Identifier	34059366
Title	Effect of Dementia on Outcomes After Surgically Treated Hip Fracture in Older Adults.
Source	Journal of Arthroplasty. 36(9):3181-3186.e4, 2021 09.
Authors	Jorissen RN; Inacio MC; Cations M; Lang C; Caughey GE; Crotty M
Authors Full Name	Jorissen, Robert N; Inacio, Maria C; Cations, Monica; Lang, Catherine; Caughey, Gillian E; Crotty, Maria.
Publication Type	Journal Article. Research Support, Non-U.S. Gov't.

Abstract Reference
Complete Reference
Find Similar
Find Citing Articles
Get it UTL

Cite + My Projects + Annotate

☐ 125.

Unique Identifier	34030672
Title	Facility and resident characteristics associated with variation in nursing home transfers: evidence from the OPTIMISTIC demonstration project.
Source	BMC Health Services Research. 21(1):492, 2021 May 24.
Authors	Blackburn J; Balio CP; Carnahan JL; Fowler NR; Hickman SE; Sachs GA; Tu W; Unroe KT
Authors Full Name	Blackburn, Justin; Balio, Casey P; Carnahan, Jennifer L; Fowler, Nicole R; Hickman, Susan E; Sachs, Greg A; Tu, Wanzhu; Unroe, Kathleen T.
Publication Type	Journal Article.

Abstract Reference
Complete Reference
Find Similar
Find Citing Articles
Full Text

Cite + My Projects + Annotate

☐ 126.

Unique Identifier	33951153
Title	Follow-up services for delirium after COVID-19-where now?.
Source	Age & Ageing. 50(3):601-604, 2021 05 05.
Authors	Rahman S; Byatt K
Authors Full Name	Rahman, Shibley; Byatt, Kit.
Publication Type	Journal Article.

Abstract Reference
Complete Reference
Find Similar
Find Citing Articles
Get it UTL

Cite + My Projects + Annotate

☐ 127.

Unique Identifier	33935096
Title	Analysis of C9orf72 Intermediate Alleles in a Retrospective Cohort of Neurological Patients: Risk Factors for Alzheimer's Disease?.
Source	Journal of Alzheimer's Disease. 81(4):1445-1451, 2021.
Authors	Serpente M; Fenoglio C; Arighi A; Fumagalli GG; Arcaro M; Sorrentino F; Visconte C; Scarpini E; Galimberti D
Authors Full Name	Serpente, Maria; Fenoglio, Chiara; Arighi, Andrea; Fumagalli, Giorgio G; Arcaro, Marina; Sorrentino, Federica; Visconte, Caterina; Scarpini, Elio; Galimberti, Daniela.
Publication Type	Journal Article. Research Support, Non-U.S. Gov't.

Abstract Reference
Complete Reference
Find Similar
Find Citing Articles
Get it UTL

Cite + My Projects + Annotate

☐ 128.

Unique Identifier	33814428
Title	The Characteristics of Social Network Structure in Later Life in Relation to Incidence of Mild Cognitive Impairment and Conversion to Probable Dementia.
Source	Journal of Alzheimer's Disease. 81(2):699-710, 2021.
Authors	Zhang Y; Natale G; Clouston S
Authors Full Name	Zhang, Yun; Natale, Ginny; Clouston, Sean.
Publication Type	Journal Article. Research Support, N.I.H., Extramural. Research Support, Non-U.S. Gov't.

Abstract Reference
Complete Reference
Find Similar
Find Citing Articles
Get it UTL

Cite + My Projects + Annotate

☐ 129.

Abstract Reference

**Unique Identifier**	33813146	Complete Reference
**Title**	Validation of the **Delirium** Diagnostic Tool-Provisional (DDT-Pro) in a **skilled nursing facility** and comparison to the 4 'A's test (4AT).	
**Source**	General Hospital Psychiatry. 70:116-123, 2021 May-Jun.	Find Similar<br>Find Citing Articles
**Authors**	Sepulveda E; Bermudez E; Gonzalez D; Cotino P; Vinuelas E; Palma J; Ciutat M; Grau I; Vilella E; Trzepacz PT; Franco JG	
**Authors Full Name**	Sepulveda, Esteban; Bermudez, Ester; Gonzalez, Dulce; Cotino, Paula; Vinuelas, Eva; Palma, Jose; Ciutat, Marta; Grau, Imma; Vilella, Elisabet; Trzepacz, Paula T; Franco, Jose G.	Full Text
**Publication Type**	Journal Article. Research Support, Non-U.S. Gov't.	

Cite + My Projects + Annotate

130.

		Abstract Reference<br>Complete Reference
**Unique Identifier**	33781222	
**Title**	Understanding **transitional care programs** for **older** adults who experience delayed discharge: a scoping review. [Review]	Find Similar<br>Find Citing Articles
**Source**	BMC Geriatrics. 21(1):210, 2021 03 29.	
**Authors**	McGilton KS; Vellani S; Krassikova A; Robertson S; Irwin C; Cumal A; Bethell J; Burr E; Keatings M; McKay S; Nichol K; Puts M; Singh A; Sidani S	
**Authors Full Name**	McGilton, Katherine S; Vellani, Shirin; Krassikova, Alexandra; Robertson, Sheryl; Irwin, Constance; Cumal, Alexia; Bethell, Jennifer; Burr, Elaine; Keatings, Margaret; McKay, Sandra; Nichol, Kathryn; Puts, Martine; Singh, Anita; Sidani, Souraya.	Full Text
**Publication Type**	Journal Article. Research Support, Non-U.S. Gov't. Review.	
**Annotation(s)**	our review<br>***Modified:*** *2021-Aug-08 10:21:25 PM*	

Cite + My Projects – Annotate

131.

		Ovid Full Text<br>Abstract Reference<br>Complete Reference
**Unique Identifier**	33740785	
**Title**	Redefining **geriatric** trauma: 55 is the new 65.	
**Source**	The Journal of Trauma and Acute Care Surgery. 90(4):738-743, 2021 04 01.	Find Similar<br>Find Citing Articles
**Authors**	Fakhry SM; Morse JL; Garland JM; Wilson NY; Shen Y; Wyse RJ; Watts DD	
**Authors Full Name**	Fakhry, Samir M; Morse, Jennifer L; Garland, Jeneva M; Wilson, Nina Y; Shen, Yan; Wyse, Ransom J; Watts, Dorraine D.	
**Publication Type**	Journal Article. Multicenter Study. Research Support, Non-U.S. Gov't.	

Article as PDF (149KB) Cite + My Projects + Annotate

132.

		Abstract Reference<br>Complete Reference
**Unique Identifier**	33739444	
**Title**	Management and outcomes of a COVID-19 outbreak in a **nursing home** with predominantly Black residents.	
**Source**	Journal of the American Geriatrics Society. 69(5):1155-1165, 2021 05.	Find Similar<br>Find Citing Articles
**Authors**	Beiting KJ; Huisingh-Scheetz M; Walker J; Graupner J; Martinchek M; Thompson K; Levine S; Gleason LJ	
**Authors Full Name**	Beiting, Kimberly J; Huisingh-Scheetz, Megan; Walker, Jacob; Graupner, Jeffrey; Martinchek, Michelle; Thompson, Katherine; Levine, Stacie; Gleason, Lauren J.	Full Text
**Publication Type**	Journal Article. Observational Study. Research Support, N.I.H., Extramural. Research Support, U.S. Gov't, P.H.S..	

Cite + My Projects + Annotate

133.

		Abstract Reference<br>Complete Reference
**Unique Identifier**	33724390	
**Title**	**Delirium** and Functional Recovery in Patients Discharged to **Skilled Nursing Facilities** After **Hospitalization** for Heart Failure.	
**Source**	JAMA Network Open. 4(3):e2037968, 2021 03 01.	Find Similar<br>Find Citing Articles
**Authors**	Madrigal C; Kim J; Jiang L; Lafo J; Bozzay M; Primack J; Correia S; Erqou S; Wu WC; Rudolph JL	
**Authors Full Name**	Madrigal, Caroline; Kim, Jenny; Jiang, Lan; Lafo, Jacob; Bozzay, Melanie; Primack, Jennifer; Correia, Stephen; Erqou, Sebhat; Wu, Wen-Chih; Rudolph, James L.	Get it UTL
**Publication Type**	Journal Article. Research Support, Non-U.S. Gov't. Research Support, U.S. Gov't, Non-P.H.S..	

Cite + My Projects + Annotate

134.

		Abstract Reference<br>Complete Reference
**Unique Identifier**	33722204	
**Title**	Genetic testing and serological screening for SARS-CoV-2 infection in a COVID-19 outbreak in a **nursing facility** in Japan.	
**Source**	BMC Infectious Diseases. 21(1):263, 2021 Mar 15.	Find Similar<br>Find Citing Articles
**Authors**	Chong Y; Tani N; Ikematsu H; Terazawa N; Nakashima H; Shimono N; Akashi K; Tanaka Y	
**Authors Full Name**	Chong, Yong; Tani, Naoki; Ikematsu, Hideyuki; Terazawa, Nobuto; Nakashima, Hitoshi; Shimono, Nobuyuki; Akashi, Koichi; Tanaka, Yosuke.	Full Text
**Publication Type**	Journal Article.	

Cite + My Projects + Annotate

135.

		Abstract Reference<br>Complete Reference
**Unique Identifier**	33579284	
**Title**	Characteristics and mortality rates among patients requiring **intermediate care**: a national cohort study using linked databases.	
**Source**	BMC Medicine. 19(1):48, 2021 02 12.	Find Similar<br>Find Citing Articles
**Authors**	Evans CJ; Potts L; Dalrymple U; Pring A; Verne J; Higginson IJ; Gao W; SPACE	
**Authors Full Name**	Evans, Catherine J; Potts, Laura; Dalrymple, Ursula; Pring, Andrew; Verne, Julia; Higginson, Irene J; Gao, Wei; SPACE.	Full Text
**Publication Type**	Journal Article. Research Support, Non-U.S. Gov't.	

Cite + My Projects + Annotate

136.

Abstract Reference
Complete Reference

**Unique Identifier**	33543243
**Title**	**Age and frailty are independently associated with increased COVID-19 mortality and increased care needs in survivors: results of an international multi-centre study.**
**Source**	Age & Ageing. 50(3):617-630, 2021 05 05.
**Authors**	Geriatric Medicine Research Collaborative; Covid Collaborative; Welch C
**Authors Full Name**	Geriatric Medicine Research Collaborative; Covid Collaborative; Welch, Carly.
**Publication Type**	Journal Article. Multicenter Study. Research Support, Non-U.S. Gov't.

Find Similar
Find Citing Articles
Get it UTL

Cite + My Projects + Annotate

137.

Abstract Reference
Complete Reference

**Unique Identifier**	33188132
**Title**	**Cumulative health deficits, *APOE* genotype, and risk for later-life mild cognitive impairment and dementia.**
**Source**	Journal of Neurology, Neurosurgery & Psychiatry. 92(2):136-142, 2021 02.
**Authors**	Ward DD; Wallace LMK; Rockwood K
**Authors Full Name**	Ward, David D; Wallace, Lindsay M K; Rockwood, Kenneth.
**Publication Type**	Journal Article. Research Support, N.I.H., Extramural. Research Support, Non-U.S. Gov't.

Find Similar
Find Citing Articles
Get it UTL

Cite + My Projects + Annotate

138.

Ovid Full Text
Abstract Reference
Complete Reference

**Unique Identifier**	33196587
**Title**	**What Factors Predict Adverse Discharge Disposition in Patients Older Than 60 Years Undergoing Lower-extremity Surgery? The Adverse Discharge in Older Patients after Lower-extremity Surgery (ADELES) Risk Score.**
**Title Comment**	Comment in: Clin Orthop Relat Res. 2021 Mar 1;479(3):558-560<br>PMID: 33201023 [https://www-ncbi-nlm-nih-gov.myaccess.library.utoronto.ca/pub...]
**Source**	Clinical Orthopaedics & Related Research. 479(3):546-547, 2021 Mar 01.
**Authors**	Schaefer MS; Hammer M; Platzbecker K; Santer P; Grabitz SD; Murugappan KR; Houle T; Barnett S; Rodriguez EK; Eikermann M
**Authors Full Name**	Schaefer, Maximilian S; Hammer, Maximilian; Platzbecker, Katharina; Santer, Peter; Grabitz, Stephanie D; Murugappan, Kadhiresan R; Houle, Tim; Barnett, Sheila; Rodriguez, Edward K; Eikermann, Matthias.
**Publication Type**	Journal Article. Multicenter Study.

Find Similar
Find Citing Articles
Full Text

Cite + My Projects + Annotate

139.

Abstract Reference
Complete Reference

**Unique Identifier**	33121870
**Title**	**Receipt of Timely Primary Care Services Following Post-Acute Skilled Nursing Facility Care.**
**Source**	Journal of the American Medical Directors Association. 22(3):701-705.e1, 2021 03.
**Authors**	Simning A; Orth J; Caprio TV; Li Y; Wang J; Temkin-Greener H
**Authors Full Name**	Simning, Adam; Orth, Jessica; Caprio, Thomas V; Li, Yue; Wang, Jinjiao; Temkin-Greener, Helena.
**Publication Type**	Journal Article. Research Support, N.I.H., Extramural. Research Support, Non-U.S. Gov't.

Find Similar
Find Citing Articles
Get it UTL

Cite + My Projects + Annotate

140.

Ovid Full Text
Abstract Reference
Complete Reference

**Unique Identifier**	33433117
**Title**	**Pharmacologic Management of Intensive Care Unit Delirium: Clinical Prescribing Practices and Outcomes in More Than 8500 Patient Encounters.**
**Source**	Anesthesia & Analgesia. 133(3):713-722, 2021 09 01.
**Authors**	Boncyk CS; Farrin E; Stollings JL; Rumbaugh K; Wilson JE; Marshall M; Feng X; Shotwell MS; Pandharipande PP; Hughes CG
**Authors Full Name**	Boncyk, Christina S; Farrin, Emily; Stollings, Joanna L; Rumbaugh, Kelli; Wilson, Jo Ellen; Marshall, Matt; Feng, Xiaoke; Shotwell, Matthew S; Pandharipande, Pratik P; Hughes, Christopher G.
**Publication Type**	Journal Article. Observational Study. Research Support, N.I.H., Extramural.

Find Similar
Find Citing Articles

Article as PDF (494KB) Cite + My Projects + Annotate

141.

Ovid Full Text
Abstract Reference
Complete Reference

**Unique Identifier**	33528173
**Title**	**Successful Community Discharge Among Older Adults With Traumatic Brain Injury in Skilled Nursing Facilities.**
**Source**	Journal of Head Trauma Rehabilitation. 36(3):E186-E198, 2021 May-Jun 01.
**Authors**	Evans E; Gutman R; Resnik L; Zonfrillo MR; Lueckel SN; Kumar RG; DeVone F; Dams-O'Connor K; Thomas KS
**Authors Full Name**	Evans, Emily; Gutman, Roee; Resnik, Linda; Zonfrillo, Mark R; Lueckel, Stephanie N; Kumar, Raj G; DeVone, Frank; Dams-O'Connor, Kristen; Thomas, Kali S.
**Publication Type**	Journal Article. Research Support, N.I.H., Extramural. Research Support, Non-U.S. Gov't. Research Support, U.S. Gov't, Non-P.H.S..

Find Similar
Find Citing Articles

Article as PDF (473KB) Cite + My Projects + Annotate

142.

Abstract Reference
Complete Reference

**Unique Identifier**	33320184
**Title**	**Individual Heterogeneity in the Probability of Hospitalization, Skilled Nursing Facility Admission, and Mortality.**
**Source**	Journals of Gerontology Series A-Biological Sciences & Medical Sciences. 76(9):1668-1677, 2021 08 13.
**Authors**	McAvay GJ; Vander Wyk B; Allore H
**Authors Full Name**	McAvay, Gail J; Vander Wyk, Brent; Allore, Heather.
**Publication Type**	Journal Article. Research Support, N.I.H., Extramural.

Find Similar
Find Citing Articles
Get it UTL

Cite + My Projects + Annotate

143.

**Unique Identifier**	33535968
**Title**	**The Frailty In Residential Sector over Time (FIRST) study: methods and baseline cohort description.**
**Source**	BMC Geriatrics. 21(1):99, 2021 02 03.
**Authors**	Jadczak AD; Robson L; Cooper T; Bell JS; Visvanathan R; FIRST Study Collaborators
**Authors Full Name**	Jadczak, Agathe Daria; Robson, Leonie; Cooper, Tina; Bell, J Simon; Visvanathan, Renuka; FIRST Study Collaborators.
**Publication Type**	Journal Article. Research Support, Non-U.S. Gov't.

Abstract Reference
Complete Reference
Find Similar
Find Citing Articles
Full Text

Cite + My Projects + Annotate

144.

**Unique Identifier**	33524341
**Title**	**Two European Examples of Acute Geriatric Units Located Outside of a General Hospital for Older Adults With Exacerbated Chronic Conditions.**
**Source**	Journal of the American Medical Directors Association. 22(6):1228-1234, 2021 06.
**Authors**	Ribbink ME; Gual N; MacNeil-Vroomen JL; Ars Ricart J; Buurman BM; Inzitari M; AGCH-Study Group
**Authors Full Name**	Ribbink, Marthe E; Gual, Neus; MacNeil-Vroomen, Janet L; Ars Ricart, Joan; Buurman, Bianca M; Inzitari, Marco; AGCH-Study Group.
**Publication Type**	Journal Article. Research Support, Non-U.S. Gov't.

Abstract Reference
Complete Reference
Find Similar
Find Citing Articles
Get it UTL

Cite + My Projects + Annotate

145.

**Unique Identifier**	33516078
**Title**	**Risk factors of readmission after geriatric hospital care: An interRAI-based cohort study in Finland.**
**Source**	Archives of Gerontology & Geriatrics. 94:104350, 2021 May-Jun.
**Authors**	Kerminen HM; Jantti PO; Valvanne JNA; Huhtala HSA; Jamsen ERK
**Authors Full Name**	Kerminen, Hanna M; Jantti, Pirkko O; Valvanne, Jaakko N A; Huhtala, Heini S A; Jamsen, Esa R K.
**Publication Type**	Journal Article.

Abstract Reference
Complete Reference
Find Similar
Find Citing Articles
Full Text

Cite + My Projects + Annotate

146.

**Unique Identifier**	33504362
**Title**	**Severe heat stroke complicated by multiple cerebral infarctions: a case report.**
**Source**	Journal of Medical Case Reports [Electronic Resource]. 15(1):24, 2021 Jan 28.
**Authors**	Kamidani R; Okada H; Kitagawa Y; Kusuzawa K; Ichihashi M; Kakino Y; Oiwa H; Yasuda R; Fukuta T; Yoshiyama N; Miyake T; Okamoto H; Suzuki K; Yamada N; Doi T; Yoshida T; Ushikoshi H; Kumada K; Yoshida S; Ogura S
**Authors Full Name**	Kamidani, Ryo; Okada, Hideshi; Kitagawa, Yuichiro; Kusuzawa, Keigo; Ichihashi, Masahiro; Kakino, Yoshinori; Oiwa, Hideaki; Yasuda, Ryu; Fukuta, Tetsuya; Yoshiyama, Naomasa; Miyake, Takahito; Okamoto, Haruka; Suzuki, Kodai; Yamada, Noriaki; Doi, Tomoaki; Yoshida, Takahiro; Ushikoshi, Hiroaki; Kumada, Keisuke; Yoshida, Shozo; Ogura, Shinji.
**Publication Type**	Case Reports. Journal Article.

Abstract Reference
Complete Reference
Find Similar
Find Citing Articles
Full Text

Cite + My Projects + Annotate

147.

**Unique Identifier**	33476569
**Title**	**Mobility and Self-Care are Associated With Discharge to Community After Home Health for People With Dementia.**
**Source**	Journal of the American Medical Directors Association. 22(7):1493-1499.e1, 2021 07.
**Authors**	Knox S; Downer B; Haas A; Ottenbacher KJ
**Authors Full Name**	Knox, Sara; Downer, Brian; Haas, Allen; Ottenbacher, Kenneth J.
**Publication Type**	Journal Article. Research Support, N.I.H., Extramural.

Abstract Reference
Complete Reference
Find Similar
Find Citing Articles
Get it UTL

Cite + My Projects + Annotate

148.

**Unique Identifier**	33440229
**Title**	**Evidence-Based Management of Acute Heart Failure. [Review]**
**Source**	Canadian Journal of Cardiology. 37(4):621-631, 2021 04.
**Authors**	Gupta AK; Tomasoni D; Sidhu K; Metra M; Ezekowitz JA
**Authors Full Name**	Gupta, Arjun K; Tomasoni, Daniela; Sidhu, Kiran; Metra, Marco; Ezekowitz, Justin A.
**Publication Type**	Journal Article. Review.

Abstract Reference
Complete Reference
Find Similar
Find Citing Articles
Get it UTL

Cite + My Projects + Annotate

149.

**Unique Identifier**	33413822
**Title**	**Polypharmacy Management in Older Patients. [Review]**
**Source**	Mayo Clinic Proceedings. 96(1):242-256, 2021 01.
**Authors**	Hoel RW; Giddings Connolly RM; Takahashi PY
**Authors Full Name**	Hoel, Robert William; Giddings Connolly, Ryan M; Takahashi, Paul Y.
**Publication Type**	Journal Article. Review. Video-Audio Media.

Abstract Reference
Complete Reference
Find Similar
Find Citing Articles
Get it UTL

Cite + My Projects + Annotate

150.

**Unique Identifier**	33370820

Abstract Reference
Complete Reference

**Title**	Length of Stay Beyond Medical Readiness in a Neurosurgical Patient Population and Associated Healthcare Costs.	Find Similar<br>Find Citing Articles<br>Get it UTL
**Title Comment**	Comment in: Neurosurgery. 2021 Feb 16;88(3):E265-E266<br>PMID: 33370838 [https://www-ncbi-nlm-nih-gov.myaccess.library.utoronto.ca/pub...]<br><br>Comment in: Neurosurgery. 2021 Aug 16;89(3):E168-E169<br>PMID: 34089328 [https://www-ncbi-nlm-nih-gov.myaccess.library.utoronto.ca/pub...]	
**Source**	Neurosurgery. 88(3):E259-E264, 2021 02 16.	
**Authors**	Linzey JR; Foshee R; Moriguchi F; Adapa AR; Koduri S; Kahn EN; Williamson CA; Sheehan K; Rajajee V; Thompson BG; Muraszko KM; Pandey AS	
**Authors Full Name**	Linzey, Joseph R; Foshee, Rachel; Moriguchi, Francine; Adapa, Arjun R; Koduri, Sravanthi; Kahn, Elyne N; Williamson, Craig A; Sheehan, Kyle; Rajajee, Venkatakrishna; Thompson, B Gregory; Muraszko, Karin M; Pandey, Aditya S.	
**Publication Type**	Journal Article.	

Cite + My Projects + Annotate

151.

		Abstract Reference<br>Complete Reference
**Unique Identifier**	33308926	
**Title**	Association of Therapy Time and Cognitive Recovery in Stroke Patients in Post-Acute Rehabilitation.	Find Similar<br>Find Citing Articles<br>Get it UTL
**Title Comment**	Comment in: J Am Med Dir Assoc. 2021 Jun;22(6):1327-1328<br>PMID: 33640313 [https://www-ncbi-nlm-nih-gov.myaccess.library.utoronto.ca/pub...]<br><br>Comment in: J Am Med Dir Assoc. 2021 Jun;22(6):1328-1329<br>PMID: 33785311 [https://www-ncbi-nlm-nih-gov.myaccess.library.utoronto.ca/pub...]	
**Source**	Journal of the American Medical Directors Association. 22(2):453-458.e3, 2021 02.	
**Authors**	Cogan AM; Weaver JA; Davidson LF; Khromouchkine N; Mallinson T	
**Authors Full Name**	Cogan, Alison M; Weaver, Jennifer A; Davidson, Leslie F; Khromouchkine, Nikolai; Mallinson, Trudy.	
**Publication Type**	Journal Article. Observational Study. Research Support, U.S. Gov't, Non-P.H.S..	

Cite + My Projects + Annotate

152.

		Abstract Reference<br>Complete Reference
**Unique Identifier**	33302219	
**Title**	Association of Geriatric Events With Perioperative Outcomes After Elective Inpatient Surgery.	Find Similar<br>Find Citing Articles<br>Get it UTL
**Source**	Journal of Surgical Research. 259:192-199, 2021 03.	
**Authors**	Dworsky JQ; Shellito AD; Childers CP; Copeland TP; Maggard-Gibbons M; Tan HJ; Saliba D; Russell MM	
**Authors Full Name**	Dworsky, Jill Q; Shellito, Adam D; Childers, Christopher P; Copeland, Timothy P; Maggard-Gibbons, Melinda; Tan, Hung-Jui; Saliba, Debra; Russell, Marcia M.	
**Publication Type**	Journal Article. Research Support, Non-U.S. Gov't. Research Support, U.S. Gov't, P.H.S..	

Cite + My Projects + Annotate

153.

		Abstract Reference<br>Complete Reference
**Unique Identifier**	33300605	
**Title**	Successful Discharge to Community Gap of FFS Medicare Beneficiaries With and Without ADRD Narrowed.	Find Similar<br>Find Citing Articles
**Source**	Journal of the American Geriatrics Society. 69(4):972-978, 2021 04.	
**Authors**	Bardenheier BH; Rahman M; Kosar C; Werner RM; Mor V	
**Authors Full Name**	Bardenheier, Barbara H; Rahman, Momotazur; Kosar, Cyrus; Werner, Rachel M; Mor, Vincent.	Full Text
**Publication Type**	Journal Article. Research Support, N.I.H., Extramural.	

Cite + My Projects + Annotate

154.

		Abstract Reference<br>Complete Reference
**Unique Identifier**	33290190	
**Title**	Skeletal Muscle Index's Impact on Discharge Disposition After Head and Neck Cancer Free Flap Reconstruction.	Find Similar<br>Find Citing Articles
**Source**	Otolaryngology - Head & Neck Surgery. 165(1):59-68, 2021 07.	
**Authors**	Jones AJ; Campiti VJ; Alwani M; Novinger LJ; Bonetto A; Sim MW; Yesensky JA; Moore MG; Mantravadi AV	
**Authors Full Name**	Jones, Alexander Joseph; Campiti, Vincent Joseph; Alwani, Mohamedkazim; Novinger, Leah J; Bonetto, Andrea; Sim, Michael W; Yesensky, Jessica A; Moore, Michael G; Mantravadi, Avinash V.	Get it UTL
**Publication Type**	Journal Article. Research Support, Non-U.S. Gov't.	

Cite + My Projects + Annotate

155.

		Abstract Reference<br>Complete Reference
**Unique Identifier**	33271123	
**Title**	Cross-Cultural Adaptation and Validation of the Italian Version of the Observational Scale of Level of Arousal.	Find Similar<br>Find Citing Articles
**Source**	Journal of the American Medical Directors Association. 22(8):1615-1620.e4, 2021 08.	
**Authors**	Martella LA; Carmisciano L; Giannotti C; Signori A; Pontremoli R; Giusti M; Gualco E; Beccati V; Marengoni A; Nencioni A; Monacelli F	
**Authors Full Name**	Martella, Lucia Anna; Carmisciano, Luca; Giannotti, Chiara; Signori, Alessio; Pontremoli, Roberto; Giusti, Massimo; Gualco, Elisa; Beccati, Valentina; Marengoni, Alessandra; Nencioni, Alessio; Monacelli, Fiammetta.	Get it UTL
**Publication Type**	Journal Article.	

Cite + My Projects + Annotate

156.

		Abstract Reference<br>Complete Reference
**Unique Identifier**	33270824	
**Title**	The impact of dementia on aged care service transitions in the last five years of life.	Find Similar<br>Find Citing Articles
**Source**	Age & Ageing. 50(4):1159-1165, 2021 06 28.	
**Authors**	Welberry HJ; Jorm LR; Barbieri S; Hsu B; Brodaty H	
**Authors Full Name**	Welberry, Heidi J; Jorm, Louisa R; Barbieri, Sebastiano; Hsu, Benjumin; Brodaty, Henry.	Get it UTL
**Publication Type**	Journal Article. Research Support, Non-U.S. Gov't.	

Cite + My Projects + Annotate

157.

Abstract Reference
Complete Reference

**Unique Identifier**	33253424
**Title**	An MDS 3.0 Distressed Behavior in **Dementia** Indicator (DBDI): A Clinical Tool to Capture Change.
**Source**	Journal of the American Geriatrics Society. 69(3):785-791, 2021 03.
**Authors**	Curyto KJ; Jedele JM; Mohr DC; Eaker A; Intrator O; Karel M
**Authors Full Name**	Curyto, Kimberly J; Jedele, Jenefer M; Mohr, David C; Eaker, April; Intrator, Orna; Karel, Michele.
**Publication Type**	Evaluation Study. Journal Article.

Find Similar
Find Citing Articles
Full Text

Cite + My Projects + Annotate

158.

Abstract Reference
Complete Reference

**Unique Identifier**	33249493
**Title**	How **Transitions** in **Dementia Care** Trajectories Affect Health Problems in Partners: A Longitudinal Analysis With Linked Health and Administrative Data.
**Source**	Journals of Gerontology Series B-Psychological Sciences & Social Sciences. 76(6):1186-1194, 2021 06 14.
**Authors**	van der Heide I; Heins MJ; van Hout HPJ; Verheij RA; Francke AL; Joling KJ
**Authors Full Name**	van der Heide, Iris; Heins, Marianne J; van Hout, Hein P J; Verheij, Robert A; Francke, Anneke L; Joling, Karlijn J.
**Publication Type**	Journal Article. Research Support, Non-U.S. Gov't.

Find Similar
Find Citing Articles
Get it UTL

Cite + My Projects + Annotate

159.

Abstract Reference
Complete Reference

**Unique Identifier**	33246842
**Title**	Health Profiles, Health **Services** Use, and **Transition** to **Dementia** in Inpatients With Late-Life Depression and Other Mental Illnesses.
**Source**	Journal of the American Medical Directors Association. 22(7):1465-1470, 2021 07.
**Authors**	Reppermund S; Heintze T; Srasuebkul P; Trollor JN
**Authors Full Name**	Reppermund, Simone; Heintze, Theresa; Srasuebkul, Preeyaporn; Trollor, Julian N.
**Publication Type**	Journal Article. Research Support, Non-U.S. Gov't.

Find Similar
Find Citing Articles
Get it UTL

Cite + My Projects + Annotate

160.

Abstract Reference
Complete Reference

**Unique Identifier**	33223395
**Title**	Trajectories of pro re nata (PRN) medication prescribing and administration in long-term **care facilities**.
**Source**	Research In Social & Administrative Pharmacy. 17(8):1463-1468, 2021 08.
**Authors**	Sharma M; Wong XY; Bell JS; Corlis M; Hogan M; Sluggett JK
**Authors Full Name**	Sharma, Monica; Wong, Xin Yee; Bell, J Simon; Corlis, Megan; Hogan, Michelle; Sluggett, Janet K.
**Publication Type**	Journal Article. Research Support, Non-U.S. Gov't.

Find Similar
Find Citing Articles
Get it UTL

Cite + My Projects + Annotate

161.

Abstract Reference
Complete Reference

**Unique Identifier**	33216954
**Title**	**Nursing Home** Transfers for Behavioral Concerns: Findings from the OPTIMISTIC Demonstration Project.
**Source**	Journal of the American Geriatrics Society. 69(2):415-423, 2021 02.
**Authors**	Hathaway EE; Carnahan JL; Unroe KT; Stump TE; O'Kelly Phillips E; Hickman SE; Fowler NR; Sachs GA; Bateman DR
**Authors Full Name**	Hathaway, Elizabeth E; Carnahan, Jennifer L; Unroe, Kathleen T; Stump, Timothy E; O'Kelly Phillips, Erin; Hickman, Susan E; Fowler, Nicole R; Sachs, Greg A; Bateman, Daniel R.
**Publication Type**	Journal Article. Research Support, N.I.H., Extramural. Research Support, U.S. Gov't, Non-P.H.S..

Find Similar
Find Citing Articles
Full Text

Cite + My Projects + Annotate

162.

Abstract Reference
Complete Reference

**Unique Identifier**	33159697
**Title**	Impact of an Advance **Care** Planning Video Intervention on **Care** of Short-Stay **Nursing Home** Patients.
**Source**	Journal of the American Geriatrics Society. 69(3):735-743, 2021 03.
**Authors**	Loomer L; Ogarek JA; Mitchell SL; Volandes AE; Gutman R; Gozalo PL; McCreedy EM; Mor V
**Authors Full Name**	Loomer, Lacey; Ogarek, Jessica A; Mitchell, Susan L; Volandes, Angelo E; Gutman, Roee; Gozalo, Pedro L; McCreedy, Ellen M; Mor, Vincent.
**Publication Type**	Journal Article. Pragmatic Clinical Trial. Research Support, N.I.H., Extramural.

Find Similar
Find Citing Articles
Full Text

Cite + My Projects + Annotate

163.

Abstract Reference
Complete Reference

**Unique Identifier**	33082097
**Title**	Using Standardized Tools to Characterize Adult Day **Program** Populations: Implications for Future Research and Clinical Practice.
**Source**	Journal of the American Medical Directors Association. 22(5):1096-1100.e1, 2021 05.
**Authors**	Dharmakulaseelan L; Berall A; Santiago AT; Gardner S; Aleong R; Edelstein B; Karuza J; Blake C; Crawford S; Naglie G
**Authors Full Name**	Dharmakulaseelan, Laavanya; Berall, Anna; Santiago, Anna Theresa; Gardner, Sandra; Aleong, Rosanne; Edelstein, Beatrise; Karuza, Jurgis; Blake, Catherine; Crawford, Steve; Naglie, Gary.
**Publication Type**	Journal Article. Research Support, Non-U.S. Gov't.

Find Similar
Find Citing Articles
Get it UTL

Cite + My Projects + Annotate

164.

Unique Identifier	33071094
Title	Predictors of return visits to the emergency department among different age groups of older adults.
Source	American Journal of Emergency Medicine. 46:241-246, 2021 08.
Authors	Oliveira J E Silva L; Jeffery MM; Campbell RL; Mullan AF; Takahashi PY; Bellolio F
Authors Full Name	Oliveira J E Silva, Lucas; Jeffery, Molly M; Campbell, Ronna L; Mullan, Aidan F; Takahashi, Paul Y; Bellolio, Fernanda.
Publication Type	Journal Article. Research Support, Non-U.S. Gov't.

Abstract Reference
Complete Reference
Find Similar
Find Citing Articles
Full Text

Cite + My Projects + Annotate

165.

Unique Identifier	33044304
Title	A Model to Assess the Outcomes Associated With Dementia With Lewy Bodies.
Source	Alzheimer Disease & Associated Disorders. 35(1):68-74, 2021 Jan-Mar 01.
Authors	Espinosa R; Davis M; Johnson S; Cline S; Weintraub D
Authors Full Name	Espinosa, Robert; Davis, Matthew; Johnson, Scott; Cline, Stephanie; Weintraub, Daniel.
Publication Type	Journal Article.

Ovid Full Text
Abstract Reference
Complete Reference
Find Similar
Find Citing Articles

Cite + My Projects + Annotate

166.

Unique Identifier	32990494
Title	Transitioning to Long-Term Care: Family Caregiver Experiences of Dementia, Communities, and Counseling.
Title Comment	Erratum in: J Aging Health. 2021 Jun-Jul;33(5-6):NP1<br>PMID: 33983060 [https://www-ncbi-nlm-nih-gov.myaccess.library.utoronto.ca/pub...]
Source	Journal of Aging & Health. 33(1-2):133-146, 2021 01.
Authors	Zmora R; Statz TL; Birkeland RW; McCarron HR; Finlay JM; Rosebush CE; Gaugler JE
Authors Full Name	Zmora, Rachel; Statz, Tamara L; Birkeland, Robyn W; McCarron, Hayley R; Finlay, Jessica M; Rosebush, Christina E; Gaugler, Joseph E.
Publication Type	Journal Article. Randomized Controlled Trial. Research Support, N.I.H., Extramural.

Abstract Reference
Complete Reference
Find Similar
Find Citing Articles
Get it UTL

Cite + My Projects + Annotate

167.

Unique Identifier	32962491
Title	Change in Social Engagement among Incident Caregivers and Controls: Findings from the Caregiving Transitions Study.
Source	Journal of Aging & Health. 33(1-2):114-124, 2021 01.
Authors	Liu C; Fabius CD; Howard VJ; Haley WE; Roth DL
Authors Full Name	Liu, Chelsea; Fabius, Chanee D; Howard, Virginia J; Haley, William E; Roth, David L.
Publication Type	Journal Article. Research Support, N.I.H., Extramural.

Abstract Reference
Complete Reference
Find Similar
Find Citing Articles
Get it UTL

Cite + My Projects + Annotate

168.

Unique Identifier	32948474
Title	Initiation of Psycholeptic Medication During Hospitalization With Recommendation for Discontinuation After Discharge.
Source	Journal of the American Medical Directors Association. 22(1):96-100.e5, 2021 01.
Authors	Conti F; Consonni D; Damanti S; Nobili A; Pasina L; Mannucci PM; Cesari M; Rossi PD
Authors Full Name	Conti, Federica; Consonni, Dario; Damanti, Sarah; Nobili, Alessandro; Pasina, Luca; Mannucci, Pier Mannuccio; Cesari, Matteo; Rossi, Paolo Dionigi.
Publication Type	Journal Article.

Abstract Reference
Complete Reference
Find Similar
Find Citing Articles
Get it UTL

Cite + My Projects + Annotate

169.

Unique Identifier	32943341
Title	Care Transitions to the Community from Veterans Affairs Nursing Homes: Experiences of Social Connection and Disconnection.
Source	Journal of the American Medical Directors Association. 22(3):682-688, 2021 03.
Authors	Simons KV; Bower ES; Gillespie SM; Mills WL
Authors Full Name	Simons, Kelsey V; Bower, Emily S; Gillespie, Suzanne M; Mills, Whitney L.
Publication Type	Journal Article. Research Support, U.S. Gov't, Non-P.H.S..

Abstract Reference
Complete Reference
Find Similar
Find Citing Articles
Get it UTL

Cite + My Projects + Annotate

170.

Unique Identifier	32940751
Title	The effect of pre-operative high doses of methylprednisolone on pain management and convalescence after total hip replacement in elderly: a double-blind randomized study.
Source	International Orthopaedics. 45(4):857-863, 2021 04.
Authors	Gadek A; Liszka H; Zajac M
Authors Full Name	Gadek, Artur; Liszka, Henryk; Zajac, Malgorzata.
Publication Type	Journal Article. Randomized Controlled Trial.

Find Similar
Find Citing Articles
Full Text

Cite + My Projects + Annotate

171.

Unique Identifier	32816014

Abstract Reference
Complete Reference

**Title**	Stress, Burden, and Well-Being in Dementia and Nondementia Caregivers: Insights From the Caregiving Transitions Study.	Find Similar<br>Find Citing Articles
**Title Comment**	Erratum in: Gerontologist. 2021 Jul 13;61(5):804<br>PMID: 33415329 [https://www-ncbi-nlm-nih-gov.myaccess.library.utoronto.ca/pub...]	
**Source**	Gerontologist. 61(5):670-679, 2021 07 13.	Get it UTL
**Authors**	Sheehan OC; Haley WE; Howard VJ; Huang J; Rhodes JD; Roth DL	
**Authors Full Name**	Sheehan, Orla C; Haley, William E; Howard, Virginia J; Huang, Jin; Rhodes, J David; Roth, David L.	
**Publication Type**	Journal Article. Research Support, N.I.H., Extramural.	

Cite + My Projects + Annotate

172.

Abstract Reference
Complete Reference

**Unique Identifier**	32776123	
**Title**	Participation in a Substance Misuse Intervention in Post-acute Care Is Associated With More Optimal Rehabilitation Outcomes.	Find Similar<br>Find Citing Articles
**Source**	Gerontologist. 61(5):787-796, 2021 07 13.	
**Authors**	Cimarolli VR; Burack O; Minahan J; Hennessa A; Stone R; Shi X	
**Authors Full Name**	Cimarolli, Verena R; Burack, Orah; Minahan, Jillian; Hennessa, Alexandra; Stone, Robyn; Shi, Xiaomei.	Get it UTL
**Publication Type**	Journal Article. Research Support, Non-U.S. Gov't.	

Cite + My Projects + Annotate

173.

Abstract Reference
Complete Reference

**Unique Identifier**	32594461	
**Title**	Cognitive status as a robust predictor of repeat falls in older Veterans in post-acute care.	Find Similar<br>Find Citing Articles
**Source**	Aging-Clinical & Experimental Research. 33(6):1677-1682, 2021 Jun.	
**Authors**	Rochette AD; Alexander NB; Cigolle CT; Hogikyan R; Phillips K; Khan FA; Stelmokas J	
**Authors Full Name**	Rochette, Amber D; Alexander, Neil B; Cigolle, Christine T; Hogikyan, Robert; Phillips, Kristin; Khan, Fareeha A; Stelmokas, Julija.	Get it UTL
**Publication Type**	Journal Article.	

Cite + My Projects + Annotate

174.

Ovid Full Text
Abstract Reference
Complete Reference

**Unique Identifier**	32366793	
**Title**	Factors Associated With Discharge Destination in Community-Dwelling Adults Admitted to Acute General Medical Units.	
**Source**	Journal of Geriatric Physical Therapy. 44(2):94-100, 2021 Apr-Jun 01.	Find Similar<br>Find Citing Articles
**Authors**	D'Souza AN; Granger CL; Patrick CJ; Kay JE; Said CM	
**Authors Full Name**	D'Souza, Aruska N; Granger, Catherine L; Patrick, Cameron J; Kay, Jacqueline E; Said, Catherine M.	
**Publication Type**	Journal Article. Observational Study. Research Support, Non-U.S. Gov't.	

Article as PDF (446KB) Cite + My Projects + Annotate

© 2022 Ovid Technologies, Inc. All rights reserved. OvidUI_04.20.00.020, SourceID 14e5c95b5e0c0a81fe93edcfe511aa92a1a5a35a

Search History saved as "OVID EMBASE TCP Systematic Review July 15_ 2021 Updated on July 9 2022"

▼ **Search History** (42)

View Saved

# ▲	Searches	Results	Type	Actions	Annotations
1	(Transition* adj3 (care* or unit* or bed* or program* or ward* or setting* or facilit* or service* or model* or centre* or center*)).tw,kw.	32226	Advanced	Display Results | More	
2	Transitional care/	4451	Advanced	Display Results | More	
3	(Intermediate adj3 (unit* or care or bed* or program* or ward* or setting* or facilit* or service* or model* or centre* or center*)).tw,kw.	7011	Advanced	Display Results | More	
4	Nursing home/	61244	Advanced	Display Results | More	
5	((Subacute or sub-acute) adj3 (unit* or care or bed* or program* or ward* or setting* or facilit* or service* or model* or centre* or center*)).tw,kw.	2609	Advanced	Display Results | More	
6	Subacute care/	1473	Advanced	Display Results | More	
7	((Postacute or post-acute) adj3 (unit* or care or bed* or program* or ward* or setting* or facilit* or service* or model* or centre* or center*)).tw,kw.	3907	Advanced	Display Results | More	
8	(Post acute adj3 (unit* or care or bed* or program* or ward* or setting* or facilit* or service* or model* or centre* or center*)).tw,kw.	2856	Advanced	Display Results | More	
9	(Skilled Nursing adj3 (unit* or bed* or program* or ward* or setting* or facilit* or service* or model* or centre* or center*)).tw,kw.	5889	Advanced	Display Results | More	
10	(Restor* adj3 (unit* or care or bed* or program* or ward* or setting* or facilit* or service* or model* or centre* or center*)).tw,kw.	6430	Advanced	Display Results | More	
11	(Convalesc* adj3 (unit* or care or bed* or program* or ward* or setting* or facilit* or service* or model*	1824	Advanced	Display Results | More	

Contract

		or centre* or center* or home* or hospital*)).tw,kw.			
☐	12	Convalescence/	59768	Advanced	Display Results | More
☐	13	or/1-12	173998	Advanced	Display Results | More
☐	14	(Old* or aged or aging).tw,kw.	3359548	Advanced	Display Results | More
☐	15	(Centenarian* or nonagenarian* or octogenarian* or geriatr* or gerontol* or senescen* or septuagenarian* or pensioner* or senile).tw,kw.	209676	Advanced	Display Results | More
☐	16	Senior*.tw,kw.	68298	Advanced	Display Results | More
☐	17	Elder*.tw,kw.	429443	Advanced	Display Results | More
☐	18	Aged/	3563945	Advanced	Display Results | More
☐	19	Very Elderly/	265709	Advanced	Display Results | More
☐	20	or/14-19	6259499	Advanced	Display Results | More
☐	21	(Cognit* adj3 impair*).tw,kw.	144264	Advanced	Display Results | More
☐	22	Mild neurocognitive disorder*.tw,kw.	383	Advanced	Display Results | More
☐	23	Cognitive deficit/	195244	Advanced	Display Results | More
☐	24	Major neurocognitive disorder*.tw,kw.	404	Advanced	Display Results | More
☐	25	Dement*.tw,kw.	200350	Advanced	Display Results | More
☐	26	Dementia/	136948	Advanced	Display Results | More
☐	27	Alzheimer disease/	228254	Advanced	Display Results | More
☐	28	Alzheimer*.tw,kw.	239923	Advanced	Display Results | More
☐	29	Frontotemporal dementia/	16170	Advanced	Display Results | More
☐	30	Frontal variant frontotemporal dementia/	2816	Advanced	Display Results | More
☐	31	HIV associated dementia/	3185	Advanced	Display Results | More
☐	32	"Mixed depression and dementia"/	146	Advanced	Display Results | More
☐	33	Multiinfarct dementia/	13614	Advanced	Display Results | More
☐	34	Pick presenile dementia/	1537	Advanced	Display Results | More
☐	35	Presenile dementia/	885	Advanced	Display Results | More
☐	36	Senile dementia/	3368	Advanced	Display Results | More
☐	37	Diffuse Lewy body disease/	10477	Advanced	Display Results | More
☐	38	Deliri*.tw,kw.	31865	Advanced	Display Results | More
☐	39	Delirium/	33641	Advanced	Display Results | More
☐	40	or/21-39	627273	Advanced	Display Results | More
☐	41	13 and 20 and 40	10320	Advanced	Display Results | More
☐	42	limit 41 to yr="2021 - 2022"	1064	Advanced	Display Results | More

Save Remove Combine with: AND OR

Save All | Edit | Create RSS | Create Auto-Alert | View Saved

Email All Search History | Copy Search History Link | Copy Search History Details

Advanced Search | Basic Search | Find Citation | Search Tools | Search Fields | Multi-Field Search

1 Resource selected | Hide | Change

Embase Classic+Embase 1947 to 2022 July 08

(•) **Keyword** ( ) Author ( ) Title ( ) Journal

Enter keyword or phrase
(* or $ for truncation)

Search

[ ] Include Multimedia
[x] Map Term to Subject Heading

Limits *(close)*

[ ] Human
[ ] English Language

Publication Year
- | -

Clinical Trials
-
Clinical Trial
Randomized Controlled Trial
Controlled Clinical Trial
Multicenter Study
Phase 1 Clinical Trial

Clinical Queries
-
Reviews (maximizes sensitivity)
Reviews (maximizes specificity)
Reviews (best balance of sensitivity and specificity)
Therapy (maximizes sensitivity)
Therapy (maximizes specificity)

Publication Types
-
Article
Article in Press
Books
Chapter
Conference Abstract

Human Age Groups
-
Embryo <first trimester>
Infant <to one year>
Child <unspecified age>
Preschool Child <1 to 6 years>
School Child <7 to 12 years>

Routes of Drug Administration
-
Buccal
Epidural
Inhalational
Intraarterial
Intraarticular

Additional Limits | Edit Limits

To search Open Access content on Ovid, go to Basic Search.

Options

View By

Text (1064 Results)
Multimedia (0 Results)

Search Information

You searched:

limit 41 to yr="2021 - 2022"

Search terms used:

aged
aging
alzheimer
disease
alzheimer*
bed*

care
care*
centenarian*
center*
centre*
cognit*
cognitive
deficit
convalesc*
convalescence
deliri*
delirium
dement*
dementia
diffuse
lewy
body
elder*
facilit*
frontal
variant
frontotemporal
geriatr*
gerontol*
hiv
associated
home*
hospital*
impair*
intermediate
major
neurocognitive
disorder*
mild
mixed
depression
and
model*
multiinfarct
nonagenarian*
nursing
home
octogenarian*
old*
pensioner*
pick
presenile
post
acute
post-acute
postacute
program*
restor*
senescen*
senile
senior*
septuagenarian*
service*
setting*
skilled
sub-acute
subacute
transition*
transitional
unit*
very
elderly
ward*

Search Returned:
1064 text results

Sort By:
-

Customize Display

Filter By

Add to Search History

Selected Only (0)

▼ Years

All Years

Current year

Past 3 years

Past 5 years

▶ Specific Year Range

▶ Subject
▶ Author
▶ Journal
▶ Publication Type

My Projects

+ New Project

Articles fr Handsearching

August 7, 2021 for TCP

HSS Aug 7, 2021

JBI EBP Tools

SUMARI

Print Email Export + My Projects Keep Selected

All Range

Clear

100 Per Page 1 Go

Next ›

1.

Abstract Reference
Complete Reference

Title	**Risk factors for developing symptomatic COVID-19 in older residents of nursing homes: A hypothesis-generating observational study.**
Source	medRxiv. (no pagination), 2022. Date of Publication: 24 Jan 2022.
Author	Escriba-Salvans A.; Rierola-Fochs S.; Farres-Godayol P.; Molas-Tuneu M.; de Souza D.L.B.; Skelton D.A.; Goutan-Roura E.; Masmitja D.A.; Minobes-Molina E.; Jerez-Roig J.
Publisher	medRxiv
Publication Type	Preprint

Find Similar
Find Citing Articles

Get it UTL

Cite
+ My Projects
+ Annotate

2.

Abstract Reference
Complete Reference

Title	**Clozapine as an Effective Alternative for Dementia- Related Psychosis Refractory to Pimavanserin.**
Source	Movement Disorders Clinical Practice. Conference: Pan American Parkinson's Disease and Movement Disorders Congress, PAS 2022. Miami, FL United States. 9(SUPPL 1) (pp S59), 2022. Date of Publication: May 2022.
Author	Thames B.; Ondo W.
Publisher	Wiley-Blackwell
Publication Type	Conference Abstract

Find Similar
Find Citing Articles

Full Text

Cite
+ My Projects
+ Annotate

3.

Abstract Reference
Complete Reference

Title	**The Impact of the Fascia Iliaca Block Beyond Perioperative Pain Control in**

Hip Fractures: A Retrospective Review.

**Source**	Geriatric Orthopaedic Surgery and Rehabilitation. 13 (no pagination), 2022. Date of Publication: April 2022.
**Author**	Houserman D.J.; Raszewski J.A.; Palmer B.; Chavan B.; Sferrella A.; Campbell M.; Santanello S.
**Publisher**	SAGE Publications Inc.
**Publication Type**	Article

Find Similar
Find Citing Articles

Get it UTL

Cite
+ My Projects
+ Annotate

4.

Abstract Reference
Complete Reference

**Title**	**Remodeling of T Cell Dynamics During Long COVID Is Dependent on Severity of SARS-CoV-2 Infection.**
**Source**	Frontiers in Immunology. 13 (no pagination), 2022. Article Number: 886431. Date of Publication: 10 Jun 2022.
**Author**	Wiech M.; Chroscicki P.; Swatler J.; Stepnik D.; De Biasi S.; Hampel M.; Brewinska-Olchowik M.; Maliszewska A.; Sklinda K.; Durlik M.; Wierzba W.; Cossarizza A.; Piwocka K.
**Publisher**	Frontiers Media S.A.
**Publication Type**	Article

Find Similar
Find Citing Articles

Get it UTL

Cite
+ My Projects
+ Annotate

5.

Abstract Reference
Complete Reference

**Title**	**Post-acute sequelae of COVID-19: Evidence of mood & cognitive impairment.**
**Source**	Brain, Behavior, and Immunity - Health. 17 (no pagination), 2021. Article Number: 100347. Date of Publication: November 2021.
**Author**	Lamontagne S.J.; Winters M.F.; Pizzagalli D.A.; Olmstead M.C.
**Publisher**	Elsevier Inc.
**Publication Type**	Article

Find Similar
Find Citing Articles

Get it UTL

Cite
+ My Projects
+ Annotate

6.

Abstract Reference
Complete Reference

**Title**	**Can we change our minds? Dementia, feeding, and advance directives in long-term care.**
**Source**	Canadian Family Physician. 68(6) (pp 405-407), 2022. Date of Publication: 01 Jun 2022.
**Author**	Wohlgemut J.
**Publisher**	College of Family Physicians of Canada
**Publication Type**	Article

Find Similar
Find Citing Articles

Full Text

Cite
+ My Projects
+ Annotate

7.

Abstract Reference
Complete Reference

**Title**	**New Onset (Incidence) of Epilepsy and Seizures in Nursing Home Residents.**
**Source**	Journal of the American Medical Directors Association. (no pagination), 2022. Date of Publication: 30 Jun 2022.
**Author**	Leppik I.E.; Birnbaum A.K.; Svensden K.; Eberly L.E.

Find Similar
Find Citing Articles

Get it UTL

Publisher NLM (Medline)

Publication Type Article

Cite
+ My Projects
+ Annotate

8.

Abstract Reference
Complete Reference

Title **Identifying factors associated with acute hospital discharge dispositions in patients with moderate-to-severe traumatic brain injury.**

Find Similar
Find Citing Articles

Source Brain Injury. 36(3) (pp 383-392), 2022. Date of Publication: 2022.

Author Lu J.; Gormley M.; Donaldson A.; Agyemang A.; Karmarkar A.; Seel R.T.

Get it UTL

Publisher Taylor and Francis Ltd.

Publication Type Article

Cite
+ My Projects
+ Annotate

9.

Abstract Reference
Complete Reference

Title **Spread of COVID-19 Infection in Long-Term Care Facilities of Trieste (Italy) during the Pre-Vaccination Era. Integrating Findings of 41 Forensic Autopsies with Geriatric Comorbidity Index as a Valid Option for the Assessment of Strength of Causation.**

Find Similar
Find Citing Articles

Source Vaccines. 10(5) (no pagination), 2022. Article Number: 774. Date of Publication: May 2022.

Full Text

Author Zanon M.; Peruch M.; Concato M.; Moreschi C.; Pizzolitto S.; Radaelli D.; D'errico S.

Publisher MDPI

Publication Type Article

Cite
+ My Projects
+ Annotate

10.

Abstract Reference
Complete Reference

Title **Nonpharmacological Interventions for Management of Behavioral and Psychological Symptoms of Dementia in Long-Term Care Facilities by Direct Caregivers: A Systematic Review.**

Find Similar
Find Citing Articles

Source Journal of gerontological nursing. 48(7) (pp 18-23), 2022. Date of Publication: 01 Jul 2022.

Get it UTL

Author Lyons T.L.; Champion J.D.

Publisher NLM (Medline)

Publication Type Article

Cite
+ My Projects
+ Annotate

11.

Abstract Reference
Complete Reference

Title **From psychiatric hospitals to residential facilities: Characteristics of patients who benefited from an institutional partnership.**

Find Similar
Find Citing Articles

Source International journal of geriatric psychiatry. 37(8) (no pagination), 2022. Date of Publication: 01 Aug 2022.

Author Stojanov T.; Perquier F.; Boiteux C.; Soudani M.L.N.; Chateau N.; Perozziello A.; Gallarda T.

Full Text

Publisher NLM (Medline)

Publication Type Article

Cite
+ My Projects
+ Annotate

12.

Abstract Reference
Complete Reference

Title Using health information technology in residential aged care homes: An integrative review to identify service and quality outcomes.

Source International Journal of Medical Informatics. 165 (no pagination), 2022. Article Number: 104824. Date of Publication: September 2022.

Author Bail K.; Gibson D.; Acharya P.; Blackburn J.; Kaak V.; Kozlovskaia M.; Turner M.; Redley B.

Publisher Elsevier Ireland Ltd

Publication Type Review

Find Similar
Find Citing Articles
Get it UTL

Cite
+ My Projects
+ Annotate

13.

Abstract Reference
Complete Reference

Title Beyond garden design: A review of outdoor occupation in hospital and residential care settings for people with dementia.

Source Australian occupational therapy journal. (no pagination), 2022. Date of Publication: 30 Jun 2022.

Author Ng L.; Oliver E.; Laver K.

Publisher NLM (Medline)

Publication Type Review

Find Similar
Find Citing Articles
Full Text

Cite
+ My Projects
+ Annotate

14.

Abstract Reference
Complete Reference

Title Advanced cancer and concomitant dementia: access to specialized palliative care, emergency room, hospital care, and place of death.

Source Acta Oncologica. 61(7) (pp 874-880), 2022. Date of Publication: 2022.

Author Furst P.; Strang P.; Hedman C.; Schultz T.

Publisher Taylor and Francis Ltd.

Publication Type Article

Find Similar
Find Citing Articles
Get it UTL

Cite
+ My Projects
+ Annotate

15.

Ovid Full Text
Abstract Reference
Complete Reference

Title Pharmacologic Management of Intensive Care Unit Delirium: Clinical Prescribing Practices and Outcomes in More Than 8500 Patient Encounters.

Source Anesthesia and Analgesia. 133(3) (pp 713-722), 2021. Date of Publication: 01 Sep 2021.

Author Boncyk C.S.; Farrin E.; Stollings J.L.; Rumbaugh K.; Wilson J.E.; Marshall M.; Feng X.; Shotwell M.S.; Pandharipande P.P.; Hughes C.G.

Publisher Lippincott Williams and Wilkins

Publication Type Article

Find Similar
Find Citing Articles

Article as PDF (494KB)
Cite
+ My Projects
+ Annotate

16.

Title Psychotropic Drug Prescription in Nursing Homes During the COVID-19 Pandemic.

Source Drugs and Aging. 39(6) (pp 467-475), 2022. Date of Publication: June 2022.

Author Ferro Uriguen A.; Laso Lucas E.; Sannino Menicucci C.; Iturrioz Arrechea I.; Alaba Trueba J.; Echevarria Orella E.; Gil Goikouria J.; Beobide Telleria I.

Publisher Adis

Publication Type Article

Abstract Reference
Complete Reference

Find Similar
Find Citing Articles

Get it UTL

Cite
+ My Projects
+ Annotate

17.

Title Perceptions and Knowledge Regarding Medical Situations at the End of Life among Older Adults in Switzerland.

Source Journal of palliative medicine. (no pagination), 2022. Date of Publication: 29 Jun 2022.

Author Meier C.; Vilpert S.; Borasio G.D.; Maurer J.; Jox R.J.

Publisher NLM (Medline)

Publication Type Article

Abstract Reference
Complete Reference

Find Similar
Find Citing Articles

Get it UTL

Cite
+ My Projects
+ Annotate

18.

Title Highlighting hybridization: a case report of virtual reality-augmented interventions to improve chronic post-stroke recovery.

Source Medicine. 101(25) (pp e29357), 2022. Date of Publication: 24 Jun 2022.

Author Bailey R.B.

Publisher NLM (Medline)

Publication Type Article

Abstract Reference
Complete Reference

Find Similar
Find Citing Articles

Full Text

Cite
+ My Projects
+ Annotate

19.

Title Residents' perspectives of mobile X-ray services in support of healthcare-in-place in residential aged care facilities: a qualitative study.

Source BMC geriatrics. 22(1) (pp 525), 2022. Date of Publication: 25 Jun 2022.

Author Dollard J.; Edwards J.; Yadav L.; Gaget V.; Tivey D.; Inacio M.; Maddern G.; Visvanathan R.

Publisher NLM (Medline)

Publication Type Article

Abstract Reference
Complete Reference

Find Similar
Find Citing Articles

Full Text

Cite
+ My Projects
+ Annotate

20.

Title Within moments of becoming-everyday citizenship in nursing homes.

Source Scandinavian journal of occupational therapy. (pp 1-12), 2022. Date of Publication: 25 Jun 2022.

Author Sund M.; Jaeger Fjetland K.; Hanisch H.

Abstract Reference
Complete Reference

Find Similar
Find Citing Articles

Get it UTL

Publisher NLM (Medline)

Publication Type Article

Cite
+ My Projects
+ Annotate

21.

Abstract Reference
Complete Reference

Title **Comprehensive geriatric assessment in older people: An umbrella review of health outcomes.**

Find Similar
Find Citing Articles

Source Age and Ageing. 51(5) (no pagination), 2022. Article Number: afac104. Date of Publication: 01 May 2022.

Author Veronese N.; Custodero C.; Demurtas J.; Smith L.; Barbagallo M.; Maggi S.; Cella A.; Vanacore N.; Aprile P.L.; Ferrucci L.; Pilotto A.; Polidori M.C.; Petra B.; Nicolas B.; Julie B.; Irwin C.; Ben C.; Annette C.; Maria C.L.A.; Rodeles C.; Alfonso C.-J.; Vito C.; Libuse D.; Franco D.; Aafke D.G.; Cathrine D.G.; Jan D.L.; Benjamin D.V.; Anne-Marie D.; Michael D.; Ayse D.; Simone D.; Amaury D.; Ami F.; Marilia F.; Nicola F.; Bahaa F.; Laura F.; Ellen F.; Rose G.; Blanca G.; Sophie G.; Javier G.P.; Goudzwaard J.A.; Antonio G.; Heidi G.; Bernd G.; Lisa H.; Vered H.; Jan-Kees H.B.; Ilaria I.; Julia I.; Javier J.; Hanna K.; Ni L.A.; Sandra L.; Isabel L.; Teresa M.S.A.; Arduino M.; Da Silva Pedro M.; Patricia M.; Hana M.-K.; Francesco M.R.; Simone M.; Andrea M.; Clarissa M.; Kiruba N.; Uomo N.; Margaret O.; Fatma O.K.K.; Marc P.; Anil P.; Tajana P.; Raymond P.N.; Izabela P.; Harmke P.; Gabriel P.; Ragnhild R.; Lisa R.; Krzysztof R.; Carlos R.; Regina R.-W.; Juhani R.; Giovanni R.; Georg R.; Dan R.; Carlo S.; Elisabet S.; Sumru S.; Veronika S.; Kaisa S.; Monica S.; Orla S.; George S.; Maria T.; Natasia T.; Ulrich T.; Eva T.; Jorien T.; Michiel V.B.; Lars V.H.; Bob V.; Heleen V.; Hana V.; Rafaela V.; Merel V.; Calin V.; Paul W.; Michael W.; Anna-Karin W.; Berenice W.; Ozlem Y.; Zaidi S.M.; Mihaela Z.; Ilo Z.; Jen Z.; Mariana A.; Suna A.; Gulistan B.-O.; Cafer B.; Charlotte B.; Olivier B.; Antonio C.; Mariana D.C.A.; Joseph F.; Sabine G.; Mehmet H.; Christopher H.; Wolfgang K.; Eva K.; Marina K.; Ai K.; Medea L.; Alessandra M.; Nida; Mahwish; Alexandru O.F.; Shane O.; Andrew O.; Claudio P.; Mirko P.; Damiano P.; Konstantinos P.; Hanna R.; Dolores S.R.; Daniel S.; Lukas S.; Susan S.; Marco S.; Pinar S.; Brendon S.; Trevor T.; Gabriel T.; Brigid U.

Get it UTL

Publisher Oxford University Press

Publication Type Review

Cite
+ My Projects
+ Annotate

22.

Abstract Reference
Complete Reference

Title **Cognitive function is associated with home discharge in subacute stroke patients: a retrospective cohort study.**

Find Similar
Find Citing Articles

Source BMC Neurology. 22(1) (no pagination), 2022. Article Number: 219. Date of Publication: December 2022.

Author Ito D.; Kawakami M.; Ishii R.; Tsujikawa M.; Honaga K.; Kondo K.; Tsuji T.

Full Text

Publisher BioMed Central Ltd

Publication Type Article

Cite
+ My Projects
+ Annotate

23.

Abstract Reference
Complete Reference

Title **Backward relative to forward walking speed and falls in older adults with dementia.**

Find Similar
Find Citing Articles

Source Gait and Posture. 96 (pp 60-66), 2022. Date of Publication: July 2022.

Author Toots A.; Domellof M.E.; Lundin-Olsson L.; Gustafson Y.; Rosendahl E.

Full Text

Publisher Elsevier B.V.

Publication Type Article

Cite
+ My Projects
+ Annotate

+ Annotate

24.

Abstract Reference
Complete Reference

Title National Multicentre Cross-Sectional Study to Identify Medical, Social and Ethical Problems of the Elderly Population in Georgia.

Source Palliative Medicine. Conference: 12th World Research Congress of the European Association for Palliative Care, EAPC 2022. Online. 36(1 SUPPL) (pp 50-51), 2022. Date of Publication: July 2022.

Author Janberidze E.; Kordzaia D.; Bakuradze N.; Gugunishvili M.; Jojua R.; Eremashvili M.; Tsikoridze N.; Dalakhishvili S.

Publisher SAGE Publications Ltd

Publication Type Conference Abstract

Find Similar
Find Citing Articles

Get it UTL

Cite
+ My Projects
+ Annotate

25.

Abstract Reference
Complete Reference

Title End-of-Life Communication in Nursing Home.

Source Palliative Medicine. Conference: 12th World Research Congress of the European Association for Palliative Care, EAPC 2022. Online. 36(1 SUPPL) (pp 14), 2022. Date of Publication: July 2022.

Author Gonella S.

Publisher SAGE Publications Ltd

Publication Type Conference Abstract

Find Similar
Find Citing Articles

Get it UTL

Cite
+ My Projects
+ Annotate

26.

Abstract Reference
Complete Reference

Title Research on Advance Care Planning in Nursing Homes During the COVID-19 Pandemic.

Source Palliative Medicine. Conference: 12th World Research Congress of the European Association for Palliative Care, EAPC 2022. Online. 36(1 SUPPL) (pp 12), 2022. Date of Publication: July 2022.

Author Preston N.

Publisher SAGE Publications Ltd

Publication Type Conference Abstract

Find Similar
Find Citing Articles

Get it UTL

Cite
+ My Projects
+ Annotate

27.

Abstract Reference
Complete Reference

Title Predictors of Adverse Outcomes and High Resource Use in Elders Hospitalized for Isolated Orthopedic Injury.

Source Canadian Journal of Anesthesia. Conference: Critical Care Canada Forum 2021. Virtual. 69(1 SUPPL) (pp S103), 2022. Date of Publication: May 2022.

Author Isaac C.J.; Moore L.; Berube M.; Belcaid A.; Dionne C.E.

Publisher Springer

Publication Type Conference Abstract

Find Similar
Find Citing Articles

Get it UTL

Cite
+ My Projects
+ Annotate

28.

Abstract Reference
[illegible]

Complete Reference

Title	**FACTORS CONTRIBUTING TO BED BLOCK AND EMERGENCY DEPARTMENT RAMPING: A PROSPECTIVE AUDIT ON PROPORTION OF MEDICAL INPATIENTS AT SIR CHARLES GAIRDNER HOSPITAL NOT REQUIRING TERTIARY CARE.**	Find Similar<br>Find Citing Articles
Source	Internal Medicine Journal. Conference: RACP Congress 2022, A Climate for Change. Online. 52(SUPPL 1) (pp 11-12), 2022. Date of Publication: May 2022.	Full Text
Author	Badeshae B.; Stoinis N.; Wei S.	
Publisher	Blackwell Publishing	
Publication Type	Conference Abstract	

Cite
+ My Projects
+ Annotate

29.

Abstract Reference
Complete Reference

Title	**NEW BIOMARKERS FOR ALZHEIMER DIAGNOSIS AND EARLY DETECTION OF MILD COGNITIVE IMPAIRMENT.**	Find Similar<br>Find Citing Articles
Source	Journal of Nutrition, Health and Aging. Conference: 2nd Euro Geroscience Conference. Toulouse France. 26(4) (pp 438), 2022. Date of Publication: April 2022.	Get it UTL
Author	Roman-Dominguez A.; Dromant M.; Borras C.	
Publisher	Springer Science+Business Media B.V.	
Publication Type	Conference Abstract	

Cite
+ My Projects
+ Annotate

30.

Abstract Reference
Complete Reference

Title	**The Association Between Race and Place of Death Among Persons With Dementia.**	Find Similar<br>Find Citing Articles
Source	Journal of Pain and Symptom Management. (no pagination), 2022. Date of Publication: 2022.	Full Text
Author	Mayan I.; Yaffe K.; James J.; Hunt L.J.	
Publisher	Elsevier Inc.	
Publication Type	Article	

Cite
+ My Projects
+ Annotate

31.

Abstract Reference
Complete Reference

Title	**The delivery of intergenerational programmes in the nursing home setting and impact on adolescents and older adults: A mixed studies systematic review.**	Find Similar<br>Find Citing Articles
Source	International journal of nursing studies. 133 (pp 104281), 2022. Date of Publication: 20 May 2022.	Full Text
Author	Laging B.; Slocombe G.; Liu P.; Radford K.; Gorelik A.	
Publisher	NLM (Medline)	
Publication Type	Review	

Cite
+ My Projects
+ Annotate

32.

Abstract Reference
Complete Reference

Title	**Functional Constipation in Older Adults: Prevalence, Clinical Symptoms and Subtypes, Association with Frailty, and Impact on Quality of Life.**	Find Similar<br>Find Citing Articles
Source	Gerontology. 68(4) (pp 397-406), 2022. Date of Publication: 01 May 2022.	

**Author**	Arco S.; Saldana E.; Serra-Prat M.; Palomera E.; Ribas Y.; Font S.; Clave P.; Mundet L.
**Publisher**	S. Karger AG
**Publication Type**	Article

Get it UTL

Cite
+ My Projects
+ Annotate

33.

Abstract Reference
Complete Reference

**Title**	The economic burden of **dementia** in low- and middle-income countries (LMICs): a systematic review.
**Source**	BMJ Global Health. 7(4) (no pagination), 2022. Article Number: e007409. Date of Publication: 04 Apr 2022.
**Author**	Mattap S.M.; Mohan D.; McGrattan A.M.; Allotey P.; Stephan B.C.M.; Reidpath D.D.; Siervo M.; Robinson L.; Chaiyakunapruk N.
**Publisher**	BMJ Publishing Group
**Publication Type**	Review

Find Similar
Find Citing Articles

Full Text

Cite
+ My Projects
+ Annotate

34.

Abstract Reference
Complete Reference

**Title**	Trends in places and causes of death among **centenarians** in Japan from 2006 to 2016.
**Source**	Geriatrics and Gerontology International. (no pagination), 2022. Date of Publication: 2022.
**Author**	Koyama T.; Higashionna T.; Maruo A.; Ushio S.; Zamami Y.; Harada K.; Hagiya H.
**Publisher**	John Wiley and Sons Inc
**Publication Type**	Article

Find Similar
Find Citing Articles

Full Text

Cite
+ My Projects
+ Annotate

35.

Complete Reference

**Title**	Coronavirus Disease-2019 in **Older** People with **Cognitive Impairment**.
**Source**	Clinics in Geriatric Medicine. (no pagination), 2022. Date of Publication: 2022.
**Author**	Rolland Y.; Baziard M.; De Mauleon A.; Dubus E.; Saidlitz P.; Soto M.E.
**Publisher**	W.B. Saunders
**Publication Type**	Review

Find Similar
Find Citing Articles

Get it UTL

Cite
+ My Projects
+ Annotate

36.

Abstract Reference
Complete Reference

**Title**	Telehealth Use and **Hospital** Readmission Rates in Long-term **Care Facilities** in Southeastern Minnesota During the COVID-19 Pandemic.
**Source**	Mayo Clinic Proceedings: Innovations, Quality and Outcomes. 6(3) (pp 186-192), 2022. Date of Publication: June 2022.
**Author**	Bogin M.H.; Chandra A.; Manggaard J.; Thorsteinsdottir B.; Hanson G.J.; Takahashi P.Y.
**Publisher**	Elsevier B.V.
**Publication Type**	Article

Find Similar
Find Citing Articles

Full Text

Cite
+ My Projects

+ My Projects
+ Annotate

37.

Abstract Reference
Complete Reference

**Title**	**Feasibility of Hair Cortisol as a Biomarker of Chronic Stress in People With Dementia.**
**Source**	Biological research for nursing. 24(3) (pp 388-399), 2022. Date of Publication: 01 Jul 2022.
**Author**	Kim E.; Bolkan C.; Crespi E.; Madigan J.
**Publisher**	NLM (Medline)
**Publication Type**	Article

Find Similar
Find Citing Articles
Get it UTL

Cite
+ My Projects
+ Annotate

38.

Abstract Reference
Complete Reference

**Title**	**Incidence and Predictive Factors of Functional Decline in Older People Living in Nursing Homes: A Systematic Review.**
**Source**	Journal of the American Medical Directors Association. (no pagination), 2022. Date of Publication: 2022.
**Author**	Moreno-Martin P.; Jerez-Roig J.; Rierola-Fochs S.; Oliveira V.R.; Farres-Godayol P.; Bezerra de Souza D.L.; Gine-Garriga M.; Booth J.; Skelton D.A.; Minobes-Molina E.
**Publisher**	Elsevier Inc.
**Publication Type**	Review

Find Similar
Find Citing Articles
Get it UTL

Cite
+ My Projects
+ Annotate

39.

Abstract Reference
Complete Reference

**Title**	**Malnutrition among patients admitted to the subacute geriatric ward during the COVID-19 pandemic era: A cross-sectional study in a tertiary hospital in Malaysia.**
**Source**	Medical Journal of Malaysia. 77(3) (pp 313-319), 2022. Date of Publication: May 2022.
**Author**	Thiam C.N.; Mathavan S.; Abdullah A.; Chong E.G.M.
**Publisher**	Malaysian Medical Association
**Publication Type**	Article

Find Similar
Find Citing Articles
Get it UTL

Cite
+ My Projects
+ Annotate

40.

Abstract Reference
Complete Reference

**Title**	**Predictors of Acute Care Transfers From Skilled Nursing Facilities: Recommendations for Preventing Unnecessary Hospitalization.**
**Source**	Research in gerontological nursing. (pp 1-7), 2022. Date of Publication: 17 Jun 2022.
**Author**	Antony A.R.; Champion J.D.
**Publisher**	NLM (Medline)
**Publication Type**	Article

Find Similar
Find Citing Articles
Get it UTL

Cite
+ My Projects
+ Annotate

41.

Abstract Reference

41.

Complete Reference

**Title**	The ethics of euthanasia in **dementia**: A qualitative content analysis of case summaries (2012-2020).
**Source**	Journal of the American Geriatrics Society. 70(6) (pp 1704-1716), 2022. Date of Publication: June 2022.
**Author**	Groenewoud A.S.; Leijten E.; van den Oever S.; van Sommeren J.; Boer T.A.
**Publisher**	John Wiley and Sons Inc
**Publication Type**	Article

Find Similar
Find Citing Articles

Full Text

Cite
+ My Projects
+ Annotate

42.

Abstract Reference
Complete Reference

**Title**	**Geriatric** patients with **dementia** show increased mortality and lack of functional recovery after hip fracture treated with hemiprosthesis.
**Source**	European journal of trauma and emergency surgery : official publication of the European Trauma Society. 48(3) (pp 1827-1833), 2022. Date of Publication: 01 Jun 2022.
**Author**	Schuetze K.; Eickhoff A.; Rutetzki K.-S.; Richter P.H.; Gebhard F.; Ehrnthaller C.
**Publisher**	NLM (Medline)
**Publication Type**	Article

Find Similar
Find Citing Articles

Get it UTL

Cite
+ My Projects
+ Annotate

43.

Abstract Reference
Complete Reference

**Title**	"It's Huge, in a Way." Conflicting Stakeholder Priorities for Managing Hearing **Impairment** for People Living with **Dementia** in Residential **Aged Care Facilities**.
**Source**	Clinical gerontologist. 45(4) (pp 844-858), 2022. Date of Publication: 01 Jul 2022.
**Author**	Bott A.; Meyer C.; Hickson L.; Pachana N.A.
**Publisher**	NLM (Medline)
**Publication Type**	Article

Find Similar
Find Citing Articles

Get it UTL

Cite
+ My Projects
+ Annotate

44.

Complete Reference

**Title**	Decision-making experiences of family **carers** of **older** people with moderate **dementia** towards community and residential **care home services**: a grounded theory inquiry (abridged secondary publication).
**Source**	Hong Kong medical journal = Xianggang yi xue za zhi. 28(3 Supplement 3) (pp 24-27), 2022. Date of Publication: 01 Jun 2022.
**Author**	Low L.P.L.; Lee D.T.F.; Lam L.W.
**Publisher**	NLM (Medline)
**Publication Type**	Article

Find Similar
Find Citing Articles

Get it UTL

Cite
+ My Projects
+ Annotate

45.

Abstract Reference
Complete Reference

**Title**	**Aged care** staff perceptions of an online training **program** for responsive behaviours of residents with **dementia**.
**Source**	Australasian journal on ageing. 41(2) (pp e112-e121), 2022. Date of Publication: 01 Jun 2022.

Find Similar
Find Citing Articles

**Author**	Almutairi H.; Stafford A.; Etherton-Beer C.; Flicker L.; Saunders R.	Full Text
**Publisher**	NLM (Medline)	
**Publication Type**	Article	

Cite
+ My Projects
+ Annotate

46.

Abstract Reference
Complete Reference

**Title**	**Prevalence and treatment of neuropathic pain diagnoses among U.S. nursing home residents.**	Find Similar<br>Find Citing Articles
**Source**	Pain. 163(7) (pp 1370-1377), 2022. Date of Publication: 01 Jul 2022.	
**Author**	Mbrah A.K.; Nunes A.P.; Hume A.L.; Zhao D.; Jesdale B.M.; Bova C.; Lapane K.L.	Full Text
**Publisher**	NLM (Medline)	
**Publication Type**	Article	

Cite
+ My Projects
+ Annotate

47.

Abstract Reference
Complete Reference

**Title**	**Falls and Preventive Practices among Institutionalized Older People.**	
**Source**	International Journal of Environmental Research and Public Health. 19(13) (no pagination), 2022. Article Number: 7577. Date of Publication: July-1 2022.	Find Similar<br>Find Citing Articles
**Author**	Baixinho C.L.; Madeira C.; Alves S.; Henriques M.A.; Dixe M.A.	Get it UTL
**Publisher**	MDPI	
**Publication Type**	Article	

Cite
+ My Projects
+ Annotate

48.

Abstract Reference
Complete Reference

**Title**	**CONSERVATIVE MANAGEMENT IN HOSPITALIZED PATIENTS WITH ADVANCED CHRONIC KIDNEY DISEASE-A SINGLE CENTRE 4-YEARS' EXPERIENCE.**	Find Similar<br>Find Citing Articles
**Source**	Nephrology Dialysis Transplantation. Conference: 59th ERA Congress. Virtual. 37(SUPPL 3) (pp i343), 2022. Date of Publication: May 2022.	Full Text
**Author**	Silva R.; Figueiredo C.; Romaozinho C.; Sa H.; Alves R.	
**Publisher**	Oxford University Press	
**Publication Type**	Conference Abstract	

Cite
+ My Projects
+ Annotate

49.

Abstract Reference
Complete Reference

**Title**	**The Utilization and National Variation of Plain X-Ray Services by Australian Residents of Long-Term Care Facilities.**	Find Similar<br>Find Citing Articles
**Source**	Journal of the American Medical Directors Association. (no pagination), 2022. Date of Publication: 2022.	
**Author**	Inacio M.C.; Jorissen R.N.; Gaget V.; Tivey D.R.; Visvanathan R.; Maddern G.J.	Get it UTL
**Publisher**	Elsevier Inc.	
**Publication Type**	Article	

Cite
+ My Projects
+ Annotate

50.

Abstract Reference
Complete Reference

Title Three-Minute Diagnostic Assessment for Delirium using the Confusion Assessment Method (3D-CAM): French translation and cultural adaptation.

Original Title Evaluation diagnostique de l'etat confusionnel de l'instrument 3D-CAM: traduction francaise et adaptation culturelle.

Source Canadian Journal of Anesthesia. 69(6) (pp 726-735), 2022. Date of Publication: June 2022.

Author Gaudet J.G.; Kull C.; Eskenazi M.L.; Diaper J.; Maillard J.; Mollard F.; Marti C.; Marcantonio E.R.; Courvoisier D.S.; Walder B.

Publisher Springer

Publication Type Article

Find Similar
Find Citing Articles

Get it UTL

Cite
+ My Projects
+ Annotate

51.

Abstract Reference
Complete Reference

Title Assessing quality of older persons' emergency transitions between long-term and acute care settings: A proof-of-concept study.

Source BMJ Open Quality. 11(1) (no pagination), 2022. Article Number: e001639. Date of Publication: 09 Mar 2022.

Author Tate K.; Mclane P.; Reid C.; Rowe B.H.; Estabrooks C.A.; Cummings G.

Publisher BMJ Publishing Group

Publication Type Article

Find Similar
Find Citing Articles

Full Text

Cite
+ My Projects
+ Annotate

52.

Abstract Reference
Complete Reference

Title Analysis of the Cost and Case-mix of Post-acute Stroke Patients in China Using Quantile Regression and the Decision-tree Models.

Source Risk Management and Healthcare Policy. 15 (pp 1113-1127), 2022. Date of Publication: 2022.

Author Zhi M.; Hu L.; Geng F.; Shao N.; Liu Y.

Publisher Dove Medical Press Ltd

Publication Type Article

Find Similar
Find Citing Articles

Get it UTL

Cite
+ My Projects
+ Annotate

53.

Abstract Reference
Complete Reference

Title Tick-borne encephalitis among US travellers, 2010-20.

Source Journal of Travel Medicine. 29(2) (no pagination), 2022. Article Number: taab167. Date of Publication: 01 Mar 2022.

Author Hills S.L.; Broussard K.R.; Broyhill J.C.; Shastry L.G.; Cossaboom C.M.; White J.L.; Machesky K.D.; Kosoy O.; Girone K.; Klena J.D.; Backenson B.P.; Gould C.V.; Lind L.; Hieronimus A.; Gaines D.N.; Wong S.J.; Choi M.J.; Laven J.J.; Staples J.E.; Fischer M.

Publisher Oxford University Press

Publication Type Article

Find Similar
Find Citing Articles

Get it UTL

Cite
+ My Projects

+ Annotate

54.

Abstract Reference
Complete Reference

Title	**Disease Burden of Huntington's Disease (HD) on People Living with HD and Care Partners in Canada.**
Source	Journal of Huntington's Disease. 11(2) (pp 179-193), 2022. Date of Publication: 2022.
Author	Shaw E.; Mayer M.; Ekwaru P.; McMullen S.; Graves E.; Wu J.W.; Budd N.; Maturi B.; Cowling T.; Mestre T.A.
Publisher	IOS Press BV
Publication Type	Article

Find Similar
Find Citing Articles

Get it UTL

Cite
+ My Projects
+ Annotate

55.

Abstract Reference
Complete Reference

Title	**Use of Intravenous Paracetamol Preoperatively Favors Lower Risk of Delirium and Functional Recovery in Elderly Patients with Hip Fracture: A Propensity Score-Matched Analysis.**
Source	Pain Research and Management. 2022 (no pagination), 2022. Article Number: 1582727. Date of Publication: 2022.
Author	Guo J.; Wang T.; Zheng X.; Long Y.; Wang X.; Zhang Q.; Liu J.; Zhang G.; Zha J.; Hou Z.; Zhang Y.
Publisher	Hindawi Limited
Publication Type	Article

Find Similar
Find Citing Articles

Get it UTL

Cite
+ My Projects
+ Annotate

56.

Abstract Reference
Complete Reference

Title	**A bridge to cross: Tube feeding and the barriers to implementation of palliative care for the advanced dementia patient.**
Source	Journal of clinical nursing. 31(13-14) (pp 1826-1834), 2022. Date of Publication: 01 Jul 2022.
Author	Matarasso Greenfeld S.; Gil E.; Agmon M.
Publisher	NLM (Medline)
Publication Type	Article

Find Similar
Find Citing Articles

Full Text

Cite
+ My Projects
+ Annotate

57.

Abstract Reference
Complete Reference

Title	**Spiritual Interventions Delivered by Nurses to Address Patients' Needs in Hospitals or Long-Term Care Facilities: A Systematic Review.**
Source	Journal of Palliative Medicine. 25(4) (pp 662-677), 2022. Date of Publication: April 2022.
Author	Dos Santos F.C.; Macieira T.G.R.; Yao Y.; Hunter S.; Madandola O.O.; Cho H.; Bjarnadottir R.I.; Dunn Lopez K.; Wilkie D.J.; Keenan G.M.
Publisher	Mary Ann Liebert Inc.
Publication Type	Review

Find Similar
Find Citing Articles

Get it UTL

Cite
+ My Projects
+ Annotate

58.

Abstract Reference
Complete Reference

Title	**Reopening the doors of Dutch nursing homes during the COVID-19 crisis: Results of an in-depth monitoring.**

Find Similar

Find Similar
Find Citing Articles

Source International Psychogeriatrics. 34(4) (pp 391-398), 2022. Date of Publication: 05 Apr 2022.

Author Koopmans R.T.C.M.; Verbeek H.; Bielderman A.; Janssen M.M.; Persoon A.; Lesman-Leegte I.; Sizoo E.M.; Hamers J.P.H.; Gerritsen D.L.

Get it UTL

Publisher Cambridge University Press

Publication Type Article

Cite
+ My Projects
+ Annotate

59.

Abstract Reference
Complete Reference

Title **Greater Opioid Use Among Nursing Home Residents in Ontario, Canada During the First 2 Waves of the COVID-19 Pandemic.**

Find Similar
Find Citing Articles

Source Journal of the American Medical Directors Association. 23(6) (pp 936-941), 2022. Date of Publication: June 2022.

Author Maxwell C.J.; Campitelli M.A.; Cotton C.A.; Hogan D.B.; Iaboni A.; Gruneir A.; Evans C.; Bronskill S.E.

Get it UTL

Publisher Elsevier Inc.

Publication Type Article

Cite
+ My Projects
+ Annotate

60.

Abstract Reference
Complete Reference

Title **Functional and cognitive outcomes after suprapubic catheter placement in nursing home residents: A national cohort study.**

Find Similar
Find Citing Articles

Source Journal of the American Geriatrics Society. (no pagination), 2022. Date of Publication: 2022.

Author Chou W.H.; Covinsky K.; Zhao S.; Boscardin W.J.; Finlayson E.; Suskind A.M.

Full Text

Publisher John Wiley and Sons Inc

Publication Type Article

Cite
+ My Projects
+ Annotate

61.

Abstract Reference
Complete Reference

Title **Higher Activity and Quality of Life Correlates with Swallowing Function in Older Adults with Low Activities of Daily Living.**

Find Similar
Find Citing Articles

Source Gerontology. 68(6) (pp 673-681), 2022. Date of Publication: 01 Jun 2022.

Author Ishii M.; Nakagawa K.; Yoshimi K.; Okumura T.; Hasegawa S.; Yamaguchi K.; Nakane A.; Tamai T.; Nagasawa Y.; Yoshizawa A.; Tohara H.

Get it UTL

Publisher S. Karger AG

Publication Type Article

Cite
+ My Projects
+ Annotate

62.

Abstract Reference
Complete Reference

Title **Longitudinal analysis of aging in place at TigerPlace: Resident function and well-being.**

Find Similar
Find Citing Articles

Source Geriatric nursing (New York, N.Y.). 45 (pp 47-54), 2022. Date of Publication: 01 May 2022.

Author Popejoy L.; Zaniletti I.; Lane K.; Anderson L.; Miller S.; Rantz M.

Get it UTL

Publisher NLM (Medline)

**Publication Type**	Article

Cite
+ My Projects
+ Annotate

63.

Abstract Reference
Complete Reference

**Title**	**Malnutrition, poor function and comorbidities predict mortality up to one year after hip fracture: a cohort study of 2800 patients.**
**Source**	European Geriatric Medicine. 13(2) (pp 433-443), 2022. Date of Publication: April 2022.
**Author**	Frandsen C.F.; Glassou E.N.; Stilling M.; Hansen T.B.
**Publisher**	Springer Science and Business Media Deutschland GmbH
**Publication Type**	Article

Find Similar
Find Citing Articles
Get it UTL

Cite
+ My Projects
+ Annotate

64.

Abstract Reference
Complete Reference

**Title**	**Psychological Effects of Social Isolation during the COVID-19 Pandemic 2020.**
**Source**	GeroPsych: The Journal of Gerontopsychology and Geriatric Psychiatry. 35(1) (pp 17-29), 2022. Date of Publication: March 2022.
**Author**	Plangger B.; Unterrainer C.; Kreh A.; Gatterer G.; Juen B.
**Publisher**	Hogrefe Publishing GmbH
**Publication Type**	Article

Find Similar
Find Citing Articles
Get it UTL

Cite
+ My Projects
+ Annotate

65.

Abstract Reference
Complete Reference

**Title**	**Potential Unintended Consequences of Antipsychotic Reduction in Ontario Nursing Homes.**
**Source**	Journal of the American Medical Directors Association. 23(6) (pp 1066-1072.e7), 2022. Date of Publication: June 2022.
**Author**	Harris D.A.; Maclagan L.C.; Iaboni A.; Austin P.C.; Rosella L.C.; Maxwell C.J.; Campitelli M.; Pequeno P.; Guan J.; Kirkham J.; Bronskill S.E.
**Publisher**	Elsevier Inc.
**Publication Type**	Article

Find Similar
Find Citing Articles
Get it UTL

Cite
+ My Projects
+ Annotate

66.

Abstract Reference
Complete Reference

**Title**	**"Well in in this neighborhood I have walked, not at all": Stroke survivors lived experience in the outdoor environment.**
**Source**	Social Science and Medicine. 305 (no pagination), 2022. Article Number: 115107. Date of Publication: July 2022.
**Author**	Twardzik E.; Colabianchi N.; Duncan L.; Lisabeth L.D.; Brown S.H.; Clarke P.J.
**Publisher**	Elsevier Ltd
**Publication Type**	Article

Find Similar
Find Citing Articles
Get it UTL

Cite
+ My Projects
+ Annotate

67.

Abstract Reference
Complete Reference

**Title**	**Effectiveness and Acceptability of e- and m-Health Interventions to Promote Physical Activity and Prevent Falls in Nursing Homes-A Systematic Review.**
**Source**	Frontiers in Physiology. 13 (no pagination), 2022. Article Number: 894397. Date of Publication: 20 May 2022.
**Author**	Diener J.; Rayling S.; Bezold J.; Krell-Roesch J.; Woll A.; Wunsch K.
**Publisher**	Frontiers Media S.A.
**Publication Type**	Review

Find Similar
Find Citing Articles

Get it UTL

Cite
+ My Projects
+ Annotate

68.

Abstract Reference
Complete Reference

**Title**	**Characteristics of centenarians in the Irish hip fracture database.**
**Source**	Age and Ageing. 114(7) (no pagination), 2021. Article Number: P401. Date of Publication: July-August 2021.
**Author**	Hogan P.C.P.; Ferris H.; Brent L.; McElwaine P.; Coughlan T.
**Publisher**	Oxford University Press
**Publication Type**	Article

Find Similar
Find Citing Articles

Get it UTL

Cite
+ My Projects
+ Annotate

69.

Complete Reference

**Title**	**An elderly woman with scars on her shins.**
**Source**	Diabetic Medicine. 39(5) (no pagination), 2022. Article Number: e14818. Date of Publication: May 2022.
**Author**	Crockett E.; Bain S.C.
**Publisher**	John Wiley and Sons Inc
**Publication Type**	Article

Find Similar
Find Citing Articles

Full Text

Cite
+ My Projects
+ Annotate

70.

Abstract Reference
Complete Reference

**Title**	**Pulmonary Artery Vasoconstriction Due to Bronchial Obstruction.**
**Source**	Journal of the Belgian Society of Radiology. 106(1) (no pagination), 2022. Article Number: 27. Date of Publication: 2022.
**Author**	Saliba T.; Salame H.; Tack D.
**Publisher**	Ubiquity Press
**Publication Type**	Article

Find Similar
Find Citing Articles

Full Text

Cite
+ My Projects
+ Annotate

71.

Abstract Reference
Complete Reference

**Title**	**Observational Variables for Considering a Switch from a Normal to a Dysphagia Diet among Older Adults Requiring Long-Term Care: A One-Year Multicenter Longitudinal Study.**
**Source**	International Journal of Environmental Research and Public Health. 19(11) (no pagination), 2022. Article Number: 6586. Date of Publication: June-1 2022.
**Author**	Takeda M.; Matsushita T.; Taira K.; Miura K.; Ohara Y.; Iwasaki M.; Ito K.;

Find Similar
Find Citing Articles

Full Text

Author [illegible]
Nakajima J.; Iwasa Y.; Itoda M.; Nishi Y.; Furuya J.; Watanabe Y.; Umemoto G.; Kishima M.; Hirano H.; Sato Y.; Yoshida M.; Yamazaki Y.

Publisher MDPI

Publication Type Article

Cite
+ My Projects
+ Annotate

72.

Abstract Reference
Complete Reference

Title **Development of a tool to evaluate medication management guidance provided to carers of people living with dementia at hospital discharge: A mixed methods study.**

Find Similar
Find Citing Articles

Source BMJ Open. 12(5) (no pagination), 2022. Article Number: e058237. Date of Publication: 02 May 2022.

Full Text

Author Sawan M.J.; Gench M.; Bond C.; Jeon Y.-H.; Hilmer S.N.; Chen T.F.; Gnjidic D.

Publisher BMJ Publishing Group

Publication Type Article

Cite
+ My Projects
+ Annotate

73.

Abstract Reference
Complete Reference

Title **Postoperative outcomes in older surgical patients with preoperative cognitive impairment: A systematic review and meta-analysis.**

Find Similar
Find Citing Articles

Source Journal of Clinical Anesthesia. 80 (no pagination), 2022. Article Number: 110883. Date of Publication: September 2022.

Author Chen L.; Au E.; Saripella A.; Kapoor P.; Yan E.; Wong J.; Tang-Wai D.F.; Gold D.; Riazi S.; Suen C.; He D.; Englesakis M.; Nagappa M.; Chung F.

Get it UTL

Publisher Elsevier Inc.

Publication Type Review

Cite
+ My Projects
+ Annotate

74.

Ovid Full Text
Abstract Reference
Complete Reference

Title **An Implementation Assessment of the Virtual Acute Care for Elders Program From the Perspective of Key Stakeholders.**

Find Similar
Find Citing Articles

Source Annals of Surgery. 275(6) (pp E752-E758), 2022. Date of Publication: 01 Jun 2022.

Author Balentine C.J.; Morris M.; Knight S.J.; Turan J.M.; Flood K.L.; Gutierrez-Meza D.; Macdonald C.L.; Bhatia S.; Brown C.J.

Publisher Lippincott Williams and Wilkins

Publication Type Article

Article as PDF (831KB)
Cite
+ My Projects
+ Annotate

75.

Abstract Reference
Complete Reference

Title **Clinical evaluation and management of a 45-year-old man with confusion, psychosis, agitation, stereotyped behavior, and impaired speech.**

Find Similar
Find Citing Articles

Source Case Reports in Psychiatry. 2022 (no pagination), 2022. Article Number: 8162871. Date of Publication: 2022.

Get it UTL

Author Deng X.; Negro P.J.; Jung P.L.; Marano C.M.; Knight S.; Doddi S.R.; Nimo N.Y.A.; Lemalefant R.M.; Myers D.A.; Haake A.K.; Chandler R.

**Publisher**	Hindawi Limited
**Publication Type**	Article

Cite
+ My Projects
+ Annotate

76.

Abstract Reference
Complete Reference

**Title**	**Hospital at Home: another piece of the armoury against COVID-19.**
**Source**	Future Healthcare Journal. 9(1) (no pagination), 2022. Date of Publication: March 2022.
**Author**	Schiff R.; Oyston M.; Quinn M.; Walters S.; McEnhill P.; Collins M.
**Publisher**	Royal College of Physicians
**Publication Type**	Article

Find Similar
Find Citing Articles
Get it UTL

Cite
+ My Projects
+ Annotate

77.

Abstract Reference
Complete Reference

**Title**	**Social Isolation and Nursing Leadership in Long-Term Care: Moving Forward After COVID-19.**
**Source**	The Nursing clinics of North America. 57(2) (pp 273-286), 2022. Date of Publication: 01 Jun 2022.
**Author**	Woods D.L.; Navarro A.E.; LaBorde P.; Dawson M.; Shipway S.
**Publisher**	NLM (Medline)
**Publication Type**	Review

Find Similar
Find Citing Articles
Get it UTL

Cite
+ My Projects
+ Annotate

78.

Abstract Reference
Complete Reference

**Title**	**Overview of hospital care of elderly patients with coronavirus infection in COVID unit in Slovenia.**
**Source**	European Respiratory Journal. Conference: International Congress of the European Respiratory Society, ERS 2021. Virtual. 58(SUPPL 65) (no pagination), 2021. Date of Publication: September 2021.
**Author**	Regvat J.; Osrajnik I.; Fosnaric I.
**Publisher**	European Respiratory Society
**Publication Type**	Conference Abstract

Find Similar
Find Citing Articles
Get it UTL

Cite
+ My Projects
+ Annotate

79.

Abstract Reference
Complete Reference

**Title**	**Respiratory tract infection in centenarians.**
**Source**	European Respiratory Journal. Conference: International Congress of the European Respiratory Society, ERS 2021. Virtual. 58(SUPPL 65) (no pagination), 2021. Date of Publication: September 2021.
**Author**	Yu C.; Aung A.; Foo C.
**Publisher**	European Respiratory Society
**Publication Type**	Conference Abstract

Find Similar
Find Citing Articles
Get it UTL

Cite
+ My Projects
+ Annotate

80.

Title Transition to Comfort Care Only and End-of-Life Trajectories in an Acute Geriatric Unit: A Secondary Analysis of the DAMAGE Cohort.

Source Journal of the American Medical Directors Association. (no pagination), 2022. Date of Publication: 2022.

Author Deschasse G.; Charpentier A.; Prod'homme C.; Genin M.; Delecluse C.; Gaxatte C.; Gerard C.; Bukor Z.; Devulder P.; Couvreur L.-A.; Bloch F.; Puisieux F.; Visade F.; Beuscart J.-B.

Publisher Elsevier Inc.

Publication Type Article

Abstract Reference
Complete Reference

Find Similar
Find Citing Articles

Get it UTL

Cite
+ My Projects
+ Annotate

81.

Title Comparing Behavioral and Psychological Symptoms of Dementia and Caregiver Distress Caused Between Older Adults With Dementia Living in the Community and in Nursing Homes.

Source Frontiers in Psychiatry. 13 (no pagination), 2022. Article Number: 881215. Date of Publication: 16 May 2022.

Author Lu X.; Ye R.; Wu J.; Rao D.; Liao X.

Publisher Frontiers Media S.A.

Publication Type Article

Abstract Reference
Complete Reference

Find Similar
Find Citing Articles

Get it UTL

Cite
+ My Projects
+ Annotate

82.

Title Life-Space Mobility in Aged Care Residents: Frailty in Residential Sector Over Time (FIRST) Study Findings.

Source Journal of the American Medical Directors Association. (no pagination), 2022. Date of Publication: 2022.

Author Jadczak A.D.; Edwards S.; Visvanathan R.

Publisher Elsevier Inc.

Publication Type Article

Abstract Reference
Complete Reference

Find Similar
Find Citing Articles

Get it UTL

Cite
+ My Projects
+ Annotate

83.

Title The National Partnership to Improve Dementia Care and Hospital Readmission Among Skilled Nursing Facility Residents.

Source Journal of Applied Gerontology. (no pagination), 2022. Date of Publication: 2022.

Author Wang S.; Temkin-Greener H.; Conwell Y.; Cai S.

Publisher SAGE Publications Inc.

Publication Type Article

Abstract Reference
Complete Reference

Find Similar
Find Citing Articles

Get it UTL

Cite
+ My Projects
+ Annotate

84.

Title Estimating the cost of an individualised music intervention for aged care residents with dementia

Abstract Reference
Complete Reference

residents with dementia.

Source Nursing older people. (no pagination), 2022. Date of Publication: 08 Jun 2022.

Author Gaviola M.A.; Searles A.; Dilworth S.; Higgins I.; Holliday E.; Inder K.J.

Publisher NLM (Medline)

Publication Type Article

Find Similar
Find Citing Articles

Get it UTL

Cite
+ My Projects
+ Annotate

85.

Abstract Reference
Complete Reference

Title **Factors Affecting Hospital Length of Stay in Geriatric Patients With a Surgically Treated Fragility Ankle Fracture.**

Source Journal of Foot and Ankle Surgery. 61(3) (pp 490-496), 2022. Date of Publication: 01 May 2022.

Author Dang Q.; Leijdesdorff H.A.; Hoogendoorn J.M.; Emmink B.L.

Publisher Academic Press Inc.

Publication Type Article

Find Similar
Find Citing Articles

Get it UTL

Cite
+ My Projects
+ Annotate

86.

Abstract Reference
Complete Reference

Title **Electronic Health Records to Rapidly Assess Biosimilar Uptake: An Example Using Insulin Glargine in a Large U.S. Nursing Home Cohort.**

Source Frontiers in Pharmacology. 13 (no pagination), 2022. Article Number: 855598. Date of Publication: 04 May 2022.

Author Hayes K.N.; Mor V.; Zullo A.R.

Publisher Frontiers Media S.A.

Publication Type Article

Find Similar
Find Citing Articles

Get it UTL

Cite
+ My Projects
+ Annotate

87.

Abstract Reference
Complete Reference

Title **Using Journey Mapping to support staff, family members and allies of people with dementia to think and act differently during a care transition: The benefits and limits of care imagination.**

Source Dementia (London, England). (pp 14713012221097237), 2022. Date of Publication: 07 Jun 2022.

Author Smith L.; Phillipson L.

Publisher NLM (Medline)

Publication Type Article

Find Similar
Find Citing Articles

Get it UTL

Cite
+ My Projects
+ Annotate

88.

Abstract Reference
Complete Reference

Title **From fact to meaning: Care practitioners' hermeneutic competence development in residential care for persons with dementia.**

Source Journal of aging studies. 61 (pp 101003), 2022. Date of Publication: 01 Jun 2022.

Author Vrerink A.; Jacobs G.; Mulder A.; Ten Kate L.

Publisher NLM (Medline)

Publication Type Article

Find Similar
Find Citing Articles

Full Text

Cite
+ My Projects
+ Annotate

89.

Abstract Reference
Complete Reference

Title CHARACTERISTICS AND TREATMENT PATTERNS OF PATIENTS WITH PARKINSON'S DISEASE PSYCHOSIS TREATED WITH PIMAVANSERIN OR ATYPICAL ANTIPSYCHOTICS.

Find Similar
Find Citing Articles

Source Journal of Managed Care and Specialty Pharmacy. Conference: Academy of Managed Care Pharmacy Nexus 2020 Conference, AMCP 2021. Denver, CO United States. 27(10-B SUPPL) (pp S66), 2021. Date of Publication: October 2021.

Get it UTL

Author Davis T.; Rui P.; Rashid N.; Doshi D.; Ruetsch C.

Publisher Academy of Managed Care Pharmacy (AMCP)

Publication Type Conference Abstract

Cite
+ My Projects
+ Annotate

90.

Abstract Reference
Complete Reference

Title ANTIPSYCHOTIC DRUG USE AND HEALTHCARE RESOURCE UTILIZATION AMONG ALZHEIMER'S DEMENTIA PATIENTS WITH AGITATION IDENTIFIED FROM A NOVEL CLAIMS-BASED ALGORITHM.

Find Similar
Find Citing Articles

Source Journal of Managed Care and Specialty Pharmacy. Conference: Academy of Managed Care Pharmacy Nexus 2020 Conference, AMCP 2021. Denver, CO United States. 27(10-B SUPPL) (pp S52), 2021. Date of Publication: October 2021.

Get it UTL

Author Teigland C.; Pulungan Z.; Bruhn D.; Hwang S.

Publisher Academy of Managed Care Pharmacy (AMCP)

Publication Type Conference Abstract

Cite
+ My Projects
+ Annotate

91.

Abstract Reference
Complete Reference

Title Behavioral and Psychological Symptoms of Dementia in Different Dementia Disorders: A Large-Scale Study of 10,000 Individuals.

Find Similar
Find Citing Articles

Source Journal of Alzheimer's Disease. 87(3) (pp 1307-1318), 2022. Date of Publication: 2022.

Author Schwertner E.; Pereira J.B.; Xu H.; Secnik J.; Winblad B.; Eriksdotter M.; Nagga K.; Religa D.

Get it UTL

Publisher IOS Press BV

Publication Type Article

Cite
+ My Projects
+ Annotate

92.

Abstract Reference
Complete Reference

Title Elderspeak communication and pain severity as modifiable factors to rejection of care in hospital dementia care.

Find Similar
Find Citing Articles

Source Journal of the American Geriatrics Society. (no pagination), 2022. Date of Publication: 2022.

Author Shaw C.A.; Ward C.; Gordon J.; Williams K.N.; Herr K.

Full Text

Publisher John Wiley and Sons Inc

Publication Type Article

Cite

+ My Projects
+ Annotate

93.

Abstract Reference
Complete Reference

**Title**	**Exploring changes to resident thriving and associated factors in Swedish nursing homes: A repeated cross-sectional study.**
**Source**	International Journal of Geriatric Psychiatry. 37(6) (no pagination), 2022. Article Number: GPS5731. Date of Publication: June 2022.
**Author**	Baxter R.; Lovheim H.; Bjork S.; Skoldunger A.; Edvardsson D.
**Publisher**	John Wiley and Sons Ltd
**Publication Type**	Article

Find Similar
Find Citing Articles

Full Text

Cite
+ My Projects
+ Annotate

94.

Abstract Reference
Complete Reference

**Title**	**BURDEN OF ILLNESS AMONG U.S. MEDICARE BENEFICIARIES WITH LATE-ONSET HUNTINGTON'S DISEASE.**
**Source**	Journal of Managed Care and Specialty Pharmacy. Conference: Annual Meeting of the Academy of Managed Care and Specialty Pharmacy, AMCP 2021. Virtual. 27(4-A SUPPL) (pp S66-S67), 2021. Date of Publication: April 2021.
**Author**	Ta J.; Reiss Reddy S.; Chang E.; Exuzides A.; Gandhy R.; Yohrling G.
**Publisher**	Academy of Managed Care Pharmacy (AMCP)
**Publication Type**	Conference Abstract

Find Similar
Find Citing Articles

Get it UTL

Cite
+ My Projects
+ Annotate

95.

Abstract Reference
Complete Reference

**Title**	**High-dose versus standard-dose vitamin D supplementation in older adults with COVID-19 (COVIT-TRIAL): A multicenter, open-label, randomized controlled superiority trial.**
**Source**	PLoS Medicine. 19(5) (no pagination), 2022. Article Number: e1003999. Date of Publication: May 2022.
**Author**	Annweiler C.; Beaudenon M.; Gautier J.; Gonsard J.; Boucher S.; Chapelet G.; Darsonval A.; Fougere B.; Guerin O.; Houvet M.; Menager P.; Roubaud-Baudron C.; Tchalla A.; Souberbielle J.-C.; Riou J.; Parot-Schinkel E.; Celarier T.
**Publisher**	Public Library of Science
**Publication Type**	Article

Find Similar
Find Citing Articles

Full Text

Cite
+ My Projects
+ Annotate

96.

Abstract Reference
Complete Reference

**Title**	**Psychometric comparison of the scales: PAINAD and Abbey Pain Scale in long-Term care facilities in Barcelona.**
**Original Title**	Comparacion psicometrica de las escalas: PAINAD y Abbey Pain Scale en centros sociosanitarios de Barcelona.
**Source**	Medicina Paliativa. 28(2) (no pagination), 2021. Date of Publication: 2021.
**Author**	Gonzalez-Vaca J.; Cobo C.M.S.; Azuela E.M.
**Publisher**	Sociedad Espanola de Cuidados Paliativos
**Publication Type**	Article

Find Similar
Find Citing Articles

Get it UTL

Cite
+ My Projects
+ Annotate

97.

Title: **Neighborhood Socioeconomic Disadvantage and Disability after Critical Illness*.**

Source: Critical Care Medicine. 50(5) (pp 733-741), 2022. Date of Publication: 01 May 2022.

Author: Falvey J.R.; Murphy T.E.; Leo-Summers L.; Gill T.M.; Ferrante L.E.

Publisher: Lippincott Williams and Wilkins

Publication Type: Article

Ovid Full Text
Abstract Reference
Complete Reference

Find Similar
Find Citing Articles

Article as PDF (860KB)
Cite
+ My Projects
+ Annotate

98.

Title: **Predictors of nursing home placement at 2 years in Alzheimer's disease: A follow-up survey from the THERAD study.**

Source: International Journal of Geriatric Psychiatry. 37(6) (no pagination), 2022. Article Number: GPS5724. Date of Publication: June 2022.

Author: Villars H.; Gardette V.; Frayssignes P.; Deperetti E.; Perrin A.; Cantet C.; Soto-Martin M.

Publisher: John Wiley and Sons Ltd

Publication Type: Article

Abstract Reference
Complete Reference

Find Similar
Find Citing Articles

Full Text

Cite
+ My Projects
+ Annotate

99.

Title: **Potentially Inappropriate Medications and Potential Prescribing Omissions in Elderly Patients Receiving Post-Acute and Long-Term Care: Application of Screening Tool of Older People's Prescriptions/Screening Tool to Alert to Right Treatment Criteria.**

Source: Frontiers in Pharmacology. 12 (no pagination), 2021. Article Number: 747523. Date of Publication: 19 Oct 2021.

Author: Candeias C.; Gama J.; Rodrigues M.; Falcao A.; Alves G.

Publisher: Frontiers Media S.A.

Publication Type: Article

Abstract Reference
Complete Reference

Find Similar
Find Citing Articles

Get it UTL

Cite
+ My Projects
+ Annotate

100.

Title: **Medicare Beneficiary Factors Associated with Skilled Nursing Facility Lengths of Stay.**

Source: Journal of Applied Gerontology. 41(5) (pp 1365-1375), 2022. Date of Publication: May 2022.

Author: Kummet C.; Schneider K.; Wang C.; Longinaker N.; Joseph-King M.; Anderson K.; Brecher A.; Vitolo S.

Publisher: SAGE Publications Inc.

Publication Type: Article

Abstract Reference
Complete Reference

Find Similar
Find Citing Articles

Get it UTL

Cite
+ My Projects

All Range

Clear

100 Per Page 1 Go

© 2022 Ovid Technologies, Inc. All rights reserved. OvidUI_04.20.00.020, SourceID 14e5c95b5e0c0a81fe93edcfe511aa92a1a5a35a

Search History saved as "OVID Psychinfo TCP Systematic Review July 15 2021_Updated on July 9 2022"

▼ Search History (35) View Saved

# ▲	Searches	Results	Type	Actions	Annotations
1	(Transition* adj3 (care* or unit* or bed* or program* or ward* or setting* or facilit* or service* or model* or centre* or center*)).tw.	10600	Advanced	Display Results | More	
2	(Intermediate adj3 (unit* or care or bed* or program* or ward* or setting* or facilit* or service* or model* or centre* or center*)).tw.	1097	Advanced	Display Results | More	
3	((Subacute or sub-acute) adj3 (unit* or care or bed* or program* or ward* or setting* or facilit* or service* or model* or centre* or center*)).tw.	320	Advanced	Display Results | More	
4	((Postacute or post-acute) adj3 (unit* or care or bed* or program* or ward* or setting* or facilit* or service* or model* or centre* or center*)).tw.	512	Advanced	Display Results | More	
5	(Post acute adj3 (unit* or care or bed* or program* or ward* or setting* or facilit* or service* or model* or centre* or center*)).tw.	345	Advanced	Display Results | More	
6	(Skilled Nursing adj3 (unit* or bed* or program* or ward* or setting* or facilit* or service* or model* or centre* or center*)).tw.	697	Advanced	Display Results | More	
7	(Restor* adj3 (unit* or care or bed* or program* or ward* or setting* or facilit* or service* or model* or centre* or center*)).tw.	1123	Advanced	Display Results | More	
8	(Convalesc* adj3 (unit* or care or bed* or program* or ward* or setting* or facilit* or service* or model* or centre* or center* or home* or hospital*)).tw.	187	Advanced	Display Results | More	
9	or/1-8	14279	Advanced	Display Results | More	

Contract

☐	10	(Old* or aged or aging).tw.	728272	Advanced	Display Results | More	
☐	11	(Centenarian* or nonagenarian* or octogenarian* or geriatr* or gerontol* or senescen* or septuagenarian* or pensioner* or senile).tw.	31536	Advanced	Display Results | More	
☐	12	Senior*.tw.	30772	Advanced	Display Results | More	
☐	13	Elder*.tw.	76816	Advanced	Display Results | More	
☐	14	Geriatric patients/	13991	Advanced	Display Results | More	
☐	15	or/10-14	790197	Advanced	Display Results | More	
☐	16	(Cognit* adj3 impair*).tw.	54047	Advanced	Display Results | More	
☐	17	Mild neurocognitive disorder*.tw.	175	Advanced	Display Results | More	
☐	18	Cognitive Impairment/	41798	Advanced	Display Results | More	
☐	19	Mild Cognitive Impairment/	8679	Advanced	Display Results | More	
☐	20	Major neurocognitive disorder*.tw.	199	Advanced	Display Results | More	
☐	21	Dement*.tw.	77138	Advanced	Display Results | More	
☐	22	Dementia/	39465	Advanced	Display Results | More	
☐	23	"Alzheimer's Disease"/	52561	Advanced	Display Results | More	
☐	24	Alzheimer*.tw.	69361	Advanced	Display Results | More	
☐	25	AIDS Dementia Complex/	155	Advanced	Display Results | More	
☐	26	Dementia with Lewy Bodies/	2237	Advanced	Display Results | More	
☐	27	Presenile Dementia/	290	Advanced	Display Results | More	
☐	28	Senile Dementia/	1078	Advanced	Display Results | More	
☐	29	Semantic Dementia/	2516	Advanced	Display Results | More	
☐	30	Vascular Dementia/	2288	Advanced	Display Results | More	
☐	31	Deliri*.tw.	8114	Advanced	Display Results | More	
☐	32	Delirium/	3802	Advanced	Display Results | More	
☐	33	or/16-32	164817	Advanced	Display Results | More	
☐	34	9 and 15 and 33	361	Advanced	Display Results | More	
☐	35	limit 34 to yr="2021 - 2022"	25	Advanced	Display Results | More	

Save Remove Combine with: AND OR

Save All | Edit | Create RSS | Create Auto-Alert | View Saved

Email All Search History | Copy Search History Link | Copy Search History Details

Advanced Search | Basic Search | Find Citation | Search Tools | Search Fields | Multi-Field Search

1 Resource selected | Hide | Change

APA PsycInfo 1806 to July Week 1 2022

◉ **Keyword** ○ Author ○ Title ○ Journal

Enter keyword or phrase
(* or $ for truncation)

Expand Term Finder ▸

Search

Search

- [ ] Include Multimedia
- [x] Map Term to Subject Heading

▾ **Limits** *(close)*

- [ ] Human
- [ ] English Language

Publication Year

- | -

Additional Limits | Edit Limits

To search Open Access content on Ovid, go to Basic Search.

Options ◀

View By

Text (25 Results)

Multimedia (0 Results)

Search Information

You searched:

limit 34 to yr="2021 - 2022"

Search terms used:

aged
aging
aids
dementia
complex
alzheimer's
disease
alzheimer*
bed*
care
care*
centenarian*
center*
centre*
cognit*
cognitive
impairment
convalesc*
deliri*
delirium
dement*
with
lewy
bodies
elder*
facilit*
geriatr*
geriatric
patients
gerontol*
home*
hospital*
impair*
intermediate
major
neurocognitive
disorder*
mild
model*
nonagenarian*
octogenarian*
old*
pensioner*
post
acute
post-acute
postacute
presenile
program*
restor*

semantic
senescen*
senile
senior*
septuagenarian*
service*
setting*
skilled
nursing
sub-acute
subacute
transition*
unit*
vascular
ward*

Search Returned:
25 text results

Sort By:
-

Customize Display

Filter By

Add to Search History

Selected Only (0)

▼ **Years**

All Years
Current year
Past 3 years
Past 5 years
▶ **Specific Year Range**

▶ **Subject**
▶ **Author**
▶ **Journal**
▶ **Publication Type**

My Projects

+ New Project

Articles fr Handsearching
August 7, 2021 for TCP
HSS Aug 7, 2021

JBI EBP Tools

SUMARI

Print Email Export + My Projects Keep Selected

All Range
Clear
100 Per Page

1.

Abstract Reference
Complete Reference

Find Similar
Find Citing Articles

Get it UTL

**Title**	**Care work in the woodwork: Medicaid home care and family caregivers' health.**
**Year of Publication**	2022
**Author**	Unger, Emily S.
**Source**	Dissertation Abstracts International: Section B: The Sciences and Engineering. Vol.83(6-B),2022, pp. No Pagination Specified.
**Publication Type**	Dissertation Abstract

Cite
+ My Projects
+ Annotate

2.

Ovid Full Text
Abstract Reference
Complete Reference

Find Similar
Find Citing Articles

**Title**	**Successful community discharge among older adults with traumatic brain injury in skilled nursing facilities. [References].**
**Year of Publication**	2021
**Author**	Evans, Emily; Gutman, Roee; Resnik, Linda; Zonfrillo, Mark R; Lueckel, Stephanie N; Kumar, Raj G; DeVone, Frank; Dams-O'Connor, Kristen; Thomas, Kali S.
**Source**	The Journal of Head Trauma Rehabilitation. Vol.36(3), 2021, pp. E186-E198.
**Publication Month/Season**	May-Jun
**Publication Type**	Journal; Peer Reviewed Journal

Article as PDF (473KB)
Cite
+ My Projects
+ Annotate

3.

Abstract Reference
Complete Reference

Find Similar
Find Citing Articles

Get it UTL

**Title**	**Communication challenges in social isolation, subjective cognitive decline, and mental health status in older adults: A scoping review (2019-2021).**
**Year of Publication**	2022
**Author**	Koszalinski, Rebecca S; Olmos, Brenda.
**Source**	Perspectives in Psychiatric Care. 2022, pp. No Pagination Specified.
**Publication Month/Season**	May
**Publication Type**	Journal; Peer Reviewed Journal

Cite
+ My Projects
+ Annotate

4.

Abstract Reference
Complete Reference

Find Similar
Find Citing Articles

Get it UTL

**Title**	**Development and evaluation of a nurse practitioner-directed doll therapy program for adults with dementia in a long-term care facility.**
**Year of Publication**	2022
**Author**	Babenko, Svetlana.
**Source**	Dissertation Abstracts International: Section B: The Sciences and Engineering. Vol.83(7-B),2022, pp. No Pagination Specified.
**Publication Type**	Dissertation Abstract

Cite
+ My Projects
+ Annotate

5.

Abstract Reference
Complete Reference

Find Similar
Find Citing Articles

**Title**	**Validation of the Delirium Diagnostic Tool-Provisional (DDT-Pro) in a skilled nursing facility and comparison to the 4 'A's test (4AT). [References].**
**Year of Publication**	2021
**Author**	Sepulveda, Esteban; Bermudez, Ester; Gonzalez, Dulce; Cotino, Paula; Vinuelas, Eva; Palma, Jose; Ciutat, Marta; Grau, Imma; Vilella, Elisabet; Trzepacz, Paula T; Franco, Jose G.

**Source**	General Hospital Psychiatry. Vol.70 2021, pp. 116-123.
**Publication Month/Season**	May-Jun
**Publication Type**	Journal; Peer Reviewed Journal

Cite
+ My Projects
+ Annotate

6.

Abstract Reference
Complete Reference

**Title**	**Using meta-ethnography to understand the care transition experience of people with dementia and their caregivers. [References].**
**Year of Publication**	2022
**Author**	Saragosa, Marianne; Jeffs, Lianne; Okrainec, Karen; Kuluski, Kerry.
**Source**	Dementia: The International Journal of Social Research and Practice. Vol.21(1), 2022, pp. 153-180.
**Publication Month/Season**	Jan
**Publication Type**	Journal; Peer Reviewed Journal

Find Similar
Find Citing Articles
Get it UTL

Cite
+ My Projects
+ Annotate

7.

Abstract Reference
Complete Reference

**Title**	**The characteristics of social network structure in later life in relation to incidence of mild cognitive impairment and conversion to probable dementia. [References].**
**Year of Publication**	2021
**Author**	Zhang, Yun; Natale, Ginny; Clouston, Sean.
**Source**	Journal of Alzheimer's Disease. Vol.81(2), 2021, pp. 699-710.
**Publication Type**	Journal; Peer Reviewed Journal

Find Similar
Find Citing Articles
Get it UTL

Cite
+ My Projects
+ Annotate

8.

Abstract Reference
Complete Reference

**Title**	**Pain treatment and functional improvement in home health care: Relationship with dementia. [References].**
**Year of Publication**	2021
**Author**	Wang, Jinjiao; Cato, Kenrick; Conwell, Yeates; Yu, Fang; Heffner, Kathi; Caprio, Thomas V; Nathan, Kobi; Monroe, Todd B; Muench, Ulrike; Li, Yue.
**Source**	Journal of the American Geriatrics Society. Vol.69(12), 2021, pp. 3545-3556.
**Publication Month/Season**	Dec
**Publication Type**	Journal; Peer Reviewed Journal

Find Similar
Find Citing Articles
Full Text

Cite
+ My Projects
+ Annotate

9.

Abstract Reference
Complete Reference

**Title**	**Patterns of incident dementia codes during the COVID-19 pandemic at an integrated healthcare system. [References].**
**Year of**	2021

Find Similar
Find Citing Articles

Publication

Author Borson, Soo; Chen, Aiyu; Wang, Susan E; Nguyen, Huong Q.

Source Journal of the American Geriatrics Society. Vol.69(12), 2021, pp. 3389-3396.

Publication Month/Season Dec

Publication Type Journal; Peer Reviewed Journal

Full Text

Cite
+ My Projects
+ Annotate

10.

Abstract Reference
Complete Reference

Title **Towards patient-centred communication in the management of older patients' medications across transitions of care: A focused ethnographic study.**

Year of Publication 2021

Author Ozavci, Guncag; Bucknall, Tracey; Woodward-Kron, Robyn; Hughes, Carmel; Jorm, Christine; Manias, Elizabeth.

Source Journal of Clinical Nursing. 2021, pp. No Pagination Specified.

Publication Month/Season Dec

Publication Type Journal; Peer Reviewed Journal

Find Similar
Find Citing Articles

Get it UTL

Cite
+ My Projects
+ Annotate

11.

Abstract Reference
Complete Reference

Title **Analysis of discharge documentation for older adults living with dementia: A cohort study. [References].**

Year of Publication 2021

Author Parker, Kirsten J; Phillips, Jane L; Luckett, Tim; Agar, Meera; Ferguson, Caleb; Hickman, Louise D.

Source Journal of Clinical Nursing. Vol.30(23-24), 2021, pp. 3634-3643.

Publication Month/Season Dec

Publication Type Journal; Peer Reviewed Journal

Find Similar
Find Citing Articles

Full Text

Cite
+ My Projects
+ Annotate

12.

Abstract Reference
Complete Reference

Title **Ageing- and dementia-friendly design: Theory and evidence from cognitive psychology, neuropsychology and environmental psychology can contribute to design guidelines that minimise spatial disorientation. [References].**

Year of Publication 2021

Author Wiener, Jan M; Pazzaglia, Francesca.

Source Cognitive Processing. Vol.22(4), 2021, pp. 715-730.

Publication Month/Season Nov

Publication Type Journal; Peer Reviewed Journal

Find Similar
Find Citing Articles

Get it UTL

Cite
+ My Projects
+ Annotate

13.

Title: **Cumulative health deficits, APOE genotype, and risk for later-life mild cognitive impairment and dementia. [References].**

Year of Publication: 2021

Author: Ward, David D; Wallace, Lindsay M. K; Rockwood, Kenneth.

Source: Journal of Neurology, Neurosurgery & Psychiatry. Vol.92(2), 2021, pp. 136-142.

Publication Month/Season: Feb

Publication Type: Journal; Peer Reviewed Journal

Abstract Reference
Complete Reference
Find Similar
Find Citing Articles
Get it UTL

Cite
+ My Projects
+ Annotate

14.

Title: **Delirium occurrence and association with outcomes in hospitalized covid-19 patients.**

Year of Publication: 2021

Author: Pagali, Sandeep; Fu, Sunyang; Lindroth, Heidi; Sohn, Sunghwan; Burton, M. Caroline; Lapid, Maria.

Source: International Psychogeriatrics. 2021, pp. No Pagination Specified.

Publication Month/Season: Sep

Publication Type: Journal; Peer Reviewed Journal

Abstract Reference
Complete Reference
Find Similar
Find Citing Articles
Get it UTL

Cite
+ My Projects
+ Annotate

15.

Title: **Participation of persons with dementia and their caregivers in research. [References].**

Year of Publication: 2021

Author: Frank, Lori; Jennings, Lee A; Petersen, Ronald C; Majid, Tabassum; Gilmore-Bykovskyi, Andrea; Schicker, Lonni; Karlawish, Jason.

Source: Journal of the American Geriatrics Society. Vol.69(7), 2021, pp. 1784-1792.

Publication Month/Season: Jul

Publication Type: Journal; Peer Reviewed Journal

Abstract Reference
Complete Reference
Find Similar
Find Citing Articles
Full Text

Cite
+ My Projects
+ Annotate

16.

Title: **Management and outcomes of a COVID-19 outbreak in a nursing home with predominantly black residents. [References].**

Year of Publication: 2021

Author: Beiting, Kimberly J; Huisingh-Scheetz, Megan; Walker, Jacob; Graupner, Jeffrey; Martinchek, Michelle; Thompson, Katherine; Levine, Stacie; Gleason, Lauren J.

Source: Journal of the American Geriatrics Society. Vol.69(5), 2021, pp. 1155-1165.

Publication Month/Season: May

Publication Type: Journal; Peer Reviewed Journal

Abstract Reference
Complete Reference
Find Similar
Find Citing Articles
Full Text

Cite
+ My Projects
+ Annotate

17.

Abstract Reference
Complete Reference

Title	**Successful discharge to community gap of FFS Medicare beneficiaries with and without ADRD narrowed. [References].**
Year of Publication	2021
Author	Bardenheier, Barbara H; Rahman, Momotazur; Kosar, Cyrus; Werner, Rachel M; Mor, Vincent.
Source	Journal of the American Geriatrics Society. Vol.69(4), 2021, pp. 972-978.
Publication Month/Season	Apr
Publication Type	Journal; Peer Reviewed Journal

Find Similar
Find Citing Articles

Full Text

Cite
+ My Projects
+ Annotate

18.

Abstract Reference
Complete Reference

Title	**Skilled nursing facility-to-home trajectories for older adults with mental illness or dementia.**
Year of Publication	2021
Author	Simning, Adam; Orth, Jessica; Temkin-Greener, Helena; Li, Yue; Simons, Kelsey V; Conwell, Yeates.
Source	The American Journal of Geriatric Psychiatry. 2021, pp. No Pagination Specified.
Publication Month/Season	Jun
Publication Type	Journal; Peer Reviewed Journal

Find Similar
Find Citing Articles

Get it UTL

Cite
+ My Projects
+ Annotate

19.

Abstract Reference
Complete Reference

Title	**"it's not the same without you:" exploring the experience and perception of transition for people with intellectual disabilities and dementia.**
Year of Publication	2021
Author	Sheth, Alisa Jordan; Kramer, Jessica M; Magasi, Susan; Heller, Tamar; Nishida, Akemi; Hammel, Joy.
Source	British Journal of Learning Disabilities. 2021, pp. No Pagination Specified.
Publication Month/Season	Jul
Publication Type	Journal; Peer Reviewed Journal

Find Similar
Find Citing Articles

Full Text

Cite
+ My Projects
+ Annotate

20.

Abstract Reference
Complete Reference

Title	**The potential role of protein kinase R as a regulator of age-related neurodegeneration. [References].**
Year of Publication	2021
Author	Martinez, Nicolas W; Gomez, Felipe E; Matus, Soledad.
Source	Frontiers in Aging Neuroscience. Vol.13 2021, ArtID 638208.

Find Similar
Find Citing Articles

Get it UTL

Publication

Publication Month/Season Apr

Publication Type Journal; Peer Reviewed Journal

Cite
+ My Projects
+ Annotate

21.

Abstract Reference
Complete Reference

Title **Cognitive screening tools for late career physicians: A critical review. [References].**

Find Similar
Find Citing Articles

Year of Publication 2021

Author Garrett, Kelly Davis; Perry, William; Williams, Betsy; Korinek, Lauri; Bazzo, David E. J.

Get it UTL

Source Journal of Geriatric Psychiatry and Neurology. Vol.34(3), 2021, pp. 171-180.

Publication Month/Season May

Publication Type Journal; Peer Reviewed Journal

Cite
+ My Projects
+ Annotate

22.

Abstract Reference
Complete Reference

Title **Delirium screening and prevention in older adult post-acute care patients.**

Find Similar
Find Citing Articles

Year of Publication 2021

Author Spear, Rebecca Anne.

Get it UTL

Source Dissertation Abstracts International: Section B: The Sciences and Engineering. Vol.82(7-B),2021, pp. No Pagination Specified.

Publication Type Dissertation Abstract

Cite
+ My Projects
+ Annotate

23.

Abstract Reference
Complete Reference

Title **An exploration of dementia friendly communities from the perspective of persons living with dementia.**

Find Similar
Find Citing Articles

Year of Publication 2021

Author Hebert, Catherine Ann.

Get it UTL

Source Dissertation Abstracts International: Section B: The Sciences and Engineering. Vol.82(8-B),2021, pp. No Pagination Specified.

Publication Type Dissertation Abstract

Cite
+ My Projects
+ Annotate

24.

Abstract Reference
Complete Reference

Title **Cerebrovascular damage after midlife transient hypertension in non-transgenic and Alzheimer's disease rats. [References].**

Find Similar
Find Citing Articles

Year of Publication 2021

Author Lai, Aaron Y; Joo, Illsung L; Trivedi, Arunachala U; Dorr, Adrienne; Hill, Mary E; Stefanovic, Bojana; McLaurin, JoAnne.

Full Text

Source Brain Research. Vol.1758 2021, ArtID 147369.

Publication

Publication Month/Season May

Publication Type Journal; Peer Reviewed Journal

Cite
+ My Projects
+ Annotate

25.

Abstract Reference
Complete Reference

Title Perceptions of Missouri special education teachers concerning their role in writing and implementing transition plans.

Find Similar
Find Citing Articles

Year of Publication 2021

Author Stewart, Tana.

Get it UTL

Source Dissertation Abstracts International Section A: Humanities and Social Sciences. Vol.82(6-A),2021, pp. No Pagination Specified.

Publication Type Dissertation Abstract

Cite
+ My Projects
+ Annotate

All Range

Clear

100 Per Page

Print Email Export + My Projects Keep Selected

English Français Italiano Deutsch 日本語 繁體中文 Español 简体中文 한국어

About Us Contact Us Privacy Policy Terms of Use

© 2022 Ovid Technologies, Inc. All rights reserved. OvidUI_04.20.00.020, SourceID 14e5c95b5e0c0a81fe93edcfe511aa92a1a5a35a

New search

Search History/Alerts

Print Search History Retrieve Searches Retrieve Alerts Save Searches / Alerts

Select / deselect all | Search with AND | Search with OR | Delete Searches | Refresh Search Results

Search ID#	Search Terms	Search Options	Actions
S38	S12 AND S19 AND S36	**Limiters -** Published Date: 20210701-20220731 **Search modes** - Boolean/Phrase	View Results (83) View Details Edit
S37	S12 AND S19 AND S36	**Search modes** - Boolean/Phrase	View Results (925) View Details Edit
S36	S20 OR S21 OR S22 OR S23 OR S24 OR S25 OR S26 OR S27 OR S28 OR S29 OR S30 OR S31 OR S32 OR S33 OR S34 OR S35	**Search modes** - Boolean/Phrase	View Results (136,029) View Details Edit
S35	MH Delirium	**Search modes** - Boolean/Phrase	View Results (7,829) View Details

				Edit
☐	S34	TI Deliri* OR AB Deliri*	**Search modes** - Boolean/Phrase	View Results (9,881) View Details Edit
☐	S33	MH "Dementia, Senile"	**Search modes** - Boolean/Phrase	View Results (1,839) View Details Edit
☐	S32	MH "Dementia, Presenile"	**Search modes** - Boolean/Phrase	View Results (115) View Details Edit
☐	S31	MH "Lewy Body Disease"	**Search modes** - Boolean/Phrase	View Results (1,341) View Details Edit
☐	S30	MH "Dementia, Multi-Infarct"	**Search modes** - Boolean/Phrase	View Results (309) View Details Edit
☐	S29	MH "Dementia, Vascular"	**Search modes** - Boolean/Phrase	View Results (1,320) View Details Edit

☐	S28	MH "AIDS Dementia Complex"	**Search modes** - Boolean/Phrase	View Results (559) View Details Edit
☐	S27	TI Alzheimer* OR AB Alzheimer*	**Search modes** - Boolean/Phrase	View Results (41,020) View Details Edit
☐	S26	MH "Alzheimer’s disease"	**Search modes** - Boolean/Phrase	View Results (36,564) View Details Edit
☐	S25	MH Dementia	**Search modes** - Boolean/Phrase	View Results (45,102) View Details Edit
☐	S24	TI Dement* OR AB Dement*	**Search modes** - Boolean/Phrase	View Results (63,373) View Details Edit
☐	S23	TI (Major neurocognitive disorder*) OR AB (Major neurocognitive disorder*)	**Search modes** - Boolean/Phrase	View Results (120) View Details Edit
☐	S22	MH "Mild Cognitive Impairment"		

			**Search modes** - Boolean/Phrase	View Results (484) View Details Edit
☐	S21	TI (Mild neurocognitive disorder*) OR AB (Mild neurocognitive disorder*)	**Search modes** - Boolean/Phrase	View Results (77) View Details Edit
☐	S20	TI (Cognit* N3 impair*) OR AB (Cognit* N3 impair*)	**Search modes** - Boolean/Phrase	View Results (35,294) View Details Edit
☐	S19	S13 OR S14 OR S15 OR S16 OR S17 OR S18	**Search modes** - Boolean/Phrase	View Results (1,298,956) View Details Edit
☐	S18	MH "Aged, 80 and over"	**Search modes** - Boolean/Phrase	View Results (325,295) View Details Edit
☐	S17	MH Aged	**Search modes** - Boolean/Phrase	View Results (898,109) View Details Edit
☐	S16	TI Elder* OR AB Elder*	**Search modes**	

			- Boolean/Phrase	View Results (112,983) View Details Edit
☐	S15	TI Senior* OR AB Senior*	**Search modes** - Boolean/Phrase	View Results (28,670) View Details Edit
☐	S14	TI ((Centenarian* or nonagenarian* or octogenarian* or geriatr* or gerontol* or senescen* or septuagenarian* or pensioner* or senile)) OR AB ((Centenarian* or nonagenarian* or octogenarian* or geriatr* or gerontol* or senescen* or septuagenarian* or pensioner* or senile))	**Search modes** - Boolean/Phrase	View Results (44,557) View Details Edit
☐	S13	TI ((Old* or aged or aging)) OR AB ((Old* or aged or aging))	**Search modes** - Boolean/Phrase	View Results (538,272) View Details Edit
☐	S12	S1 OR S2 OR S3 OR S4 OR S5 OR S6 OR S7 OR S8 OR S9 OR S10 OR S11	**Search modes** - Boolean/Phrase	View Results (29,456) View Details Edit
☐	S11	TI ((Convalesc* N3 (unit* or care or bed* or program* or ward* or setting* or facilit* or service* or model* or centre* or center* or home* or hospital*))) OR AB ((Convalesc* N3 (unit* or care or bed* or program* or ward* or setting* or facilit* or service* or model* or centre* or center* or home* or hospital*)))	**Search modes** - Boolean/Phrase	View Results (381) View Details Edit
☐	S10		**Search modes**	

		TI ((Restor* N3 (unit* or care or bed* or program* or ward* or setting* or facilit* or service* or model* or centre* or center*))) OR AB ((Restor* N3 (unit* or care or bed* or program* or ward* or setting* or facilit* or service* or model* or centre* or center*)))	- Boolean/Phrase	View Results (1,922) View Details Edit
☐	S9	MH Skilled Nursing Facilities	**Search modes** - Boolean/Phrase	View Results (4,692) View Details Edit
☐	S8	TI (((Skilled Nursing) N3 (unit* or bed* or program* or ward* or setting* or facilit* or service* or model* or centre* or center*))) OR AB (((Skilled Nursing) N3 (unit* or bed* or program* or ward* or setting* or facilit* or service* or model* or centre* or center*)))	**Search modes** - Boolean/Phrase	View Results (3,057) View Details Edit
☐	S7	TI (((Post acute) N3 (unit* or care or bed* or program* or ward* or setting* or facilit* or service* or model* or centre* or center*))) OR AB (((Post acute) N3 (unit* or care or bed* or program* or ward* or setting* or facilit* or service* or model* or centre* or center*)))	**Search modes** - Boolean/Phrase	View Results (1,780) View Details Edit
☐	S6	TI (((Postacute or post-acute) N3 (unit* or care or bed* or program* or ward* or setting* or facilit* or service* or model* or centre* or center*))) OR AB (((Postacute or post-acute) N3 (unit* or care or bed* or program* or ward* or setting* or facilit* or service* or model* or centre* or center*)))	**Search modes** - Boolean/Phrase	View Results (2,628) View Details Edit
☐	S5	MH Subacute care	**Search modes** - Boolean/Phrase	View Results (1,907) View Details Edit
☐	S4		**Search modes**	

		TI (((Subacute or sub-acute) N3 (unit* or care or bed* or program* or ward* or setting* or facilit* or service* or model* or centre* or center*))) OR AB (((Subacute or sub-acute) N3 (unit* or care or bed* or program* or ward* or setting* or facilit* or service* or model* or centre* or center*)))	- Boolean/Phrase	View Results (1,157) View Details Edit
☐	S3	TI ((Intermediate N3 (unit* or care or bed* or program* or ward* or setting* or facilit* or service* or model* or centre* or center*))) OR AB ((Intermediate N3 (unit* or care or bed* or program* or ward* or setting* or facilit* or service* or model* or centre* or center*)))	**Search modes** - Boolean/Phrase	View Results (1,947) View Details Edit
☐	S2	MH Transitional Care	**Search modes** - Boolean/Phrase	View Results (3,093) View Details Edit
☐	S1	TI ((Transition* N3 (care* or unit* or bed* or program* or ward* or setting* or facilit* or service* or model* or centre* or center*))) OR AB ((Transition* N3 (care* or unit* or bed* or program* or ward* or setting* or facilit* or service* or model* or centre* or center*)))	**Search modes** - Boolean/Phrase	View Results (14,787) View Details Edit

1. Using documentary films to teach nurses about gender and the vulnerabilities facing older men with advanced dementia.

(includes abstract) Bartlett, Ruth; Jøranson, Nina; Breievne, Grete Journal of Clinical Nursing (John Wiley & Sons, Inc.), Jul2022; 31(13/14): 1817-1825. 9p. (Article - review, tables/charts) ISSN: 0962-1067

Subjects: Motion Pictures; Vulnerability Education; Gender Bias Education; Dementia, Senile Nursing; Men's Health

2. Study on the Correlation between Chinese Medicine Syndrome and Cognitive Dysfunction in Mild Cognitive Impairment.

(includes abstract) Lin, Zhiying; Huang, Tianwen; Zheng, Guanyi; Chen, Runqiong; Yao, Minglong; Liu, Wenhong; Li, Shujie
Evidence-based Complementary & Alternative Medicine (eCAM), 6/24/2022; 1-6. 6p. (Article - research, tables/charts) ISSN: 1741-427X

Subjects: Mild Cognitive Impairment; Medicine, Chinese Traditional; Cognition Disorders

Show all 4 Images

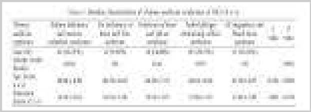
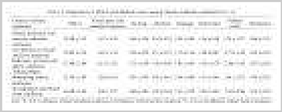
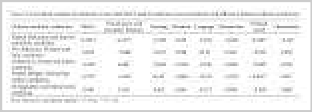

HTML Full Text **PDF Full Text**

3. Probiotic supplementation demonstrates therapeutic potential in treating gut dysbiosis and improving neurocognitive function in age-related dementia.

(includes abstract) Meng, Henry Yue Hong; Mak, Christopher Chi Hang; Mak, Wing Yan; Zuo, Tao; Ko, Ho; Chan, Francis Ka Leung
European Journal of Nutrition, Jun2022; 61(4): 1701-1734. 34p. (Article - meta analysis, pictorial, research, systematic review, tables/charts) ISSN: 1436-6207

Subjects: Probiotics Therapeutic Use; Dietary Supplementation; Therapeutics; Gut Microbiota Pathology; Cognition Disorders Diet Therapy; Dementia, Senile Diet Therapy; Pathologic Processes Diet Therapy; Treatment Outcomes

4. Dementia screening in rural-dwelling Chinese older adults: The utility of a smell test and the self-rated AD8.

(includes abstract) Dong, Yi; Wang, Yongxiang; Liu, Keke; Hou, Tingting; Han, Xiaolei; Cong, Lin; Ren, Yifei; Zhang, Qinghua; Tang, Shi; Ekström, Ingrid; Laukka, Erika J.; Du, Yifeng; Qiu, Chengxuan Journal of the American Geriatrics Society, Apr2022; 70(4): 1106-1116. 11p. (Article - equations & formulas, research, tables/charts) ISSN: 0002-8614

Subjects: Dementia, Senile Diagnosis; Health Screening Methods; Rural Population In Old Age; Chinese Psychosocial Factors; Smell Evaluation; Cognition Evaluation; Alzheimer's Disease Diagnosis; Dementia, Vascular Diagnosis; Clinical Assessment Tools; Sensitivity and Specificity; Aged: 65+ years; Female; Male

5. 老年痴呆病人激越行为的研究进展.

李翠翠; 祝筠 Chinese Evidence-based Nursing, Mar2022; 8(6): 754-762. 9p. (Article) ISSN: 2095-8668

Subjects: Dementia, Senile; Dementia Patients

6. What's good for the heart is good for the mind. . .

(includes abstract) Quinn, Terence J Age & Ageing, Mar2022; 51(3): 1-2. 2p. (Editorial - commentary, editorial) ISSN: 0002-0729

Subjects: Atrial Fibrillation Drug Therapy; Antiarrhythmia Agents Therapeutic Use; Atrial Fibrillation Therapy; Catheter Ablation; Dementia, Senile Prevention and Control; Aged: 65+ years

7. Effects of diabetes and obesity on cognitive impairment and mortality in older mexicans.

(includes abstract) Milani, Sadaf Arefi; Lopez, David S.; Downer, Brian; Samper-Ternent, Rafael; Wong, Rebeca Archives of Gerontology & Geriatrics, Mar2022; 99 N.PAG-N.PAG. 1p. (Article - research) ISSN: 0167-4943

Subjects: Diabetes Mellitus Complications; Diabetes Mellitus Complications; Obesity Complications; Obesity Complications; Dementia, Senile Risk Factors; Diabetes Mellitus Mortality; Obesity Mortality; Comorbidity; Middle Aged: 45-64 years; Aged: 65+ years

8. Evolución de la capacidad funcional en personas mayores en programa de atención domiciliaria y evaluación de factores predictivos de mortalidad.

(includes abstract) Roure-Murillo, Rosa; Nuin Orrio, Carmen; Rodriguez Cala, Ana; Ángel Escobar-Bravo, Miguel Gerokomos, Mar2022; 33(1): 21-26. 6p. (Article - research, tables/charts) ISSN: 1134-928X

Subjects: Home Health Care In Old Age; Functional Status In Old Age; Mortality Risk Factors; Risk Assessment; Community Programs Spain; Aged: 65+ years

9. Undiagnosed Dementia a Risk for Older Patients.

(includes abstract) Hospital Case Management, Mar2022; 30(3): 1-2. 2p. (Article) ISSN: 1087-0652

Subjects: Undiagnosed Diseases; Dementia; Dementia, Senile Risk Factors; Case Management

Cited References: (1)

10. Physical Activity and its Influencing Factors in Community-Dwelling Older Adults With Dementia: A Path Analysis.

(includes abstract) Li, Bei; Huang, Xiuxiu; Meng, Chenchen; Wan, Qiaoqin; Sun, Yongan Clinical Nursing Research, Feb2022; 31(2): 301-309. 9p. (Article - research, tables/charts) ISSN: 1054-7738

Subjects: Physical Activity; Community Living In Old Age; Dementia In Old Age; Activities of Daily Living; Caregivers; Fear; Accidental Falls; Aged: 65+ years; Middle Aged: 45-64 years; Aged, 80 & over; Male; Female

11. An update on cognitive frailty: Its definition, impact, associated factors and underlying mechanisms, and interventions.

(includes abstract) Sugimoto, Taiki; Arai, Hidenori; Sakurai, Takashi Geriatrics & Gerontology International, Feb2022; 22(2): 99-109. 11p. (Article - review, tables/charts) ISSN: 1444-1586

Subjects: Cognition Disorders; Frailty Syndrome

12. The Effect of Food Odor Exposure on Appetite and Nutritional Intake of Older Adults with Dementia.

(includes abstract) Verwijs, M. H.; van de Rest, O.; van der Putten, G.-J.; de Groot, L. C. P. G. M.; Boesveldt, Sanne Journal of Nutrition, Health & Aging, Feb2022; 26(2): 112-118. 7p. (Article - research, tables/charts) ISSN: 1279-7707

Subjects: Dementia In Old Age; Appetite; Nutrition; Food Intake In Old Age; Odors; Body Weight; Nursing Home Patients Netherlands; Aged: 65+ years

13. Au et al. Respond to "Body Mass Index and Risk of Dementia".

(includes abstract) Au, Rhoda; Li, Jinlei; Liu, Chunyu American Journal of Epidemiology, Dec2021; 190(12): 2515-2516. 2p. (Article - response) ISSN: 0002-9262

Subjects: Body Mass Index; Obesity In Adulthood; Obesity In Middle Age; Dementia Risk Factors; Dementia, Senile Risk Factors; Adult: 19-44 years; Middle Aged: 45-64 years

14. Erratum to: Diagnostic accuracy of dementia screening tools in the Chinese population: a systematic review and meta-analysis of 167 diagnostic studies...Huo Z, Lin J, Bat BKK et al. Diagnostic accuracy of dementia screening tools in the Chinese population: a systematic review and meta-analysis of 167 diagnostic studies. AGE AGEING. 2021;50(4):1093-1101.

(includes abstract) Huo, Zhaohua; Lin, Jiaer; Bat, Baker K K; Chan, Joyce Y C; Tsoi, Kelvin K F; Yip, Benjamin H K *In:* Age & Ageing; Nov2021; v.50. n.6, e16-e16. 1p. (Correction Notice - corrected article) ISSN: 0002-0729

Subjects: Psychological Tests; Dementia, Senile Diagnosis; Chinese

15. Creutzfeldt-Jakob Disease in Nonagenarian: A Rare Presentation from India.

(includes abstract) Khichar, Shubhakaran P.; Sutariya, Nirav L.; Bhargava, Amita N.; Pradhan, Sangeeta R. *In:* Annals of Indian Academy of Neurology; Nov/Dec2021; v.24. n.6, 1012-1014. 3p. (Case Study - case study, diagnostic images, letter, tables/charts, tracings) ISSN: 0972-2327

Subjects: Creutzfeldt-Jakob Syndrome Diagnosis; Creutzfeldt-Jakob Syndrome Symptoms; Nonagenarians India; Aged, 80 & over; Male

16. Psychosocial and Physical Contextual Factors in Cognitive Aging in Older Blacks.

(includes abstract) Sol, Ketlyne; Hill-Jarrett, Tanisha G. Annual Review of Gerontology & Geriatrics, 2021; 41(1): 327-362. 36p. (Article - review, tables/charts) ISSN: 0198-8794

Subjects: Cognitive Aging; Black Persons Psychosocial Factors; Environment; Neighborhood Characteristics; Aged: 65+ years

17. Normative tensions in filial caring for a mother with dementia: A narrative perspective.

(includes abstract) Luichies, Ina; Goossensen, Anne; van der Meide, Hanneke Dementia (14713012), Nov2021; 20(8): 2766-2778. 13p. (Article - research, tables/charts) ISSN: 1471-3012

Subjects: Stress, Psychological; Caregiver Support; Mothers; Dementia, Senile

18. Playing the long game: Exploring the phenomenon of dementia-friendly golf.

(includes abstract) Norval, Robbie S; Henderson, Fiona; Whittam, Geoff Dementia (14713012), Nov2021; 20(8): 2867-2875. 9p. (Article - research) ISSN: 1471-3012

Subjects: Dementia, Senile; Golf; Aged: 65+ years

19. Nutrition, Physical Activity, and Other Lifestyle Factors in the Prevention of Cognitive Decline and Dementia.

(includes abstract) Dominguez, Ligia J.; Veronese, Nicola; Vernuccio, Laura; Catanese, Giuseppina; Inzerillo, Flora; Salemi, Giuseppe; Barbagallo, Mario Nutrients, Nov2021; 13(11): 4080. 1p. (Article - review, tables/charts) ISSN: 2072-6643

Subjects: Nutrition; Physical Activity; Life Style; Cognition Disorders Prevention and Control; Dementia, Senile Prevention and Control; Food Habits; Dietary Supplements Therapeutic Use; Sleep; Social Participation; Aged: 65+ years

20. Clinical Observation on the Effect of Systematic Nursing Intervention on Cognitive Function, Life Activity Ability, and Quality of Life of Senile Dementia Patients.

(includes abstract) Zheng, Yuan; Xu, Xiaoyan; Zheng, Birong Evidence-based Complementary & Alternative Medicine (eCAM), 10/12/2021; 1-7. 7p. (Article - research, tables/charts) ISSN: 1741-427X

Subjects: Nursing Interventions; Cognition Evaluation; Activities of Daily Living Evaluation; Quality of Life Evaluation; Dementia, Senile; Dementia Patients

Show all 9 Images

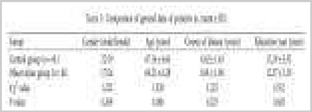
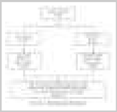
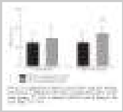
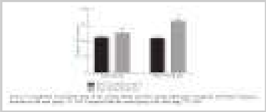

PDF Full Text

21. Admission to long-stay residential care and mortality among people with and without dementia living at home but on the boundary of residential care: a competing risks survival analysis.

(includes abstract) Carter, L.; O'Neill, S.; Austin, Peter C.; Keogh, F.; Pierce, M.; O'Shea, E. Aging & Mental Health, Oct2021; 25(10): 1869-1876. 8p. (Article - research, tables/charts) ISSN: 1360-7863

Subjects: Residential Care; Psychiatric Home Care; Risk Assessment; Dementia, Senile Mortality; Survival Analysis

22. Challenges in the provision of digital technologies to elderly with dementia to support ageing in place: a case study of a Swedish municipality.

(includes abstract) Tsertsidis, Antonios Disability & Rehabilitation: Assistive Technology, Oct2021; 16(7): 758-768. 11p. (Article - research, tables/charts) ISSN: 1748-3107

Subjects: Digital Technology In Old Age; Dementia, Senile; Local Government Sweden; Assistive Technology Devices Statistics and Numerical Data; Aged: 65+ years

23. Improved quality of care and reduced healthcare costs at the end-of-life among older people with dementia who received palliative home care: A nationwide propensity score-matched decedent cohort study.

(includes abstract) Miranda, Rose; Smets, Tinne; De Schreye, Robrecht; Faes, Kristof; Van Den Noortgate, Nele; Cohen, Joachim; Van den Block, Lieve Palliative Medicine, Oct2021; 35(9): 1701-1712. 12p. (Article - research, tables/charts) ISSN: 0269-2163

Subjects: Quality of Health Care Evaluation; Health Care Costs; Palliative Care Economics; Home Health Care Economics; Dementia, Senile Therapy; Aged: 65+ years; Female; Male

24. Effects of olfactory stimulation on cognitive function and behavior problems in older adults with dementia: A systematic literature review.

(includes abstract) Cha, Hyegyeong; Kim, Sisook; Seo, Min-sook; Kim, Han-song Geriatric Nursing, Sep2021; 42(5): 1210-1217. 8p. (Article - research, systematic review) ISSN: 0197-4572

Subjects: Cognition; Dementia, Senile; Neurodegenerative Diseases; Smell; Aromatherapy; Sensory Stimulation

25. Association of verbal fluency test with progression to dementia in non-demented older adults.

(includes abstract) Umegaki, Hiroyuki; Suzuki, Yusuke Geriatrics & Gerontology International, Sep2021; 21(9): 868-869. 2p. (Article - letter, research, tables/charts) ISSN: 1444-1586

Subjects: Dementia, Senile; Verbal Behavior; Neuropsychological Tests Japan; Mild Cognitive Impairment; Language Tests Japan

26. Function-Focused Goal Attainment and Discharge Outcomes in Hospitalized Persons With Dementia.

(includes abstract) Boltz, Marie; Monturo, Cheryl; Brockway, Cindy; Kuzmik, Ashley; Jones, Joanne Roman; Resnick, Barbara Journal of Gerontological Nursing, Sep2021; 47(9): 13-20. 8p. (Article - research, tables/charts) ISSN: 0098-9134

Subjects: Dementia Patients; Goal Attainment; Hospitalization of Older Persons; Delirium; Severity of Illness; Patient Discharge; Functional Status; Aged: 65+ years; Aged, 80 & over; Male; Female

Linked Full Text

27. Barriers to telehealth access among homebound older adults.

(includes abstract) Kalicki, Alexander V.; Moody, Kate A.; Franzosa, Emily; Gliatto, Peter M.; Ornstein, Katherine A. Journal of the American Geriatrics Society, Sep2021; 69(9): 2404-2411. 8p. (Article - research, tables/charts) ISSN: 0002-8614

Subjects: Telehealth Utilization; Health Services Accessibility; Homebound Persons In Old Age; Aged: 65+ years; Aged, 80 & over

28. Mediterranean Diet Protects Against Memory Loss and Dementia.

(includes abstract) Life Extension, Sep2021; 27(9): 22-22. 1p. (Article - pictorial) ISSN: 1524-198X

Subjects: Consumer Health Information; Mediterranean Diet; Memory Disorders Prevention and Control; Dementia, Senile Prevention and Control; Biological Markers Cerebrospinal Fluid; Cognition Evaluation

29. Care Square: Enhancing care of older adults with cognitive impairments.

(includes abstract) WILLIAMS, RHONDA; JACQUES, KATELYN KEITH; MIRZA, SEVDA; MATTERS, LORETTA; GUERRIER, LILLIAN DENISE Nursing, Sep2021; 51(9): 66-70. 5p. (Article - tables/charts) ISSN: 0360-4039

Subjects: Gerontologic Care; Cognition Disorders In Old Age; Program Implementation; Program Development; Aged: 65+ years

30. Multidisciplinary pharmacotherapy collaboration for home-based older adults with dementia: a study focusing on physicians, pharmacists, and nursing professionals.

(includes abstract) Suwa, Sayuri; Tsujimura, Mayuko; Yumoto, Akiyo; Iwata, Naoko; Shimamura, Atsuko Psychogeriatrics, Sep2021; 21(5): 749-762. 14p. (Article - research, tables/charts) ISSN: 1346-3500

Subjects: Multidisciplinary Care Team; Collaboration; Dementia, Senile Drug Therapy; Home Health Care; Physician Attitudes; Pharmacist Attitudes; Nurse Attitudes; Aged: 65+ years

31. Long COVID and beyond: post-traumatic stress disorder and delayed-onset post-traumatic stress disorder.

(includes abstract) Dening, Karen Harrison British Journal of Neuroscience Nursing, Aug/Sep2021; 17(4): 130-131. 2p. (Editorial - editorial) ISSN: 1747-0307

Subjects: COVID-19 Complications; Stress Disorders, Post-Traumatic Risk Factors; Delayed Onset; Post-Acute COVID-19 Syndrome

32. 失智老年人家庭照顾者家庭弹性研究进展.

(includes abstract) 乔巨波; 唐庆蓉 Chinese Nursing Research, Aug2021; 35(15): 2718-2723. 6p. (Article - review) ISSN: 1009-6493

Subjects: Dementia; Dementia, Senile; Caregivers

33. Low or Declining Gait Speed is Associated With Risk of Developing Dementia Over 5 Years Among People Aged 85 Years and Over.

(includes abstract) Öhlin, Jerry; Gustafson, Yngve; Littbrand, Håkan; Olofsson, Birgitta; Toots, Annika Journal of Aging & Physical Activity, Aug2021; 29(4): 678-685. 8p. (Article - research, tables/charts) ISSN: 1063-8652

Subjects: Walking Speed; Dementia, Senile Risk Factors; Cognition Disorders; Risk Assessment; Aged, 80 & over

34. Diagnostic accuracy of dementia screening tools in the Chinese population: a systematic review and meta-analysis of 167 diagnostic studies.

(includes abstract) Huo, Zhaohua; Lin, Jiaer; Bat, Baker K K; Chan, Joyce Y C; Tsoi, Kelvin K F; Yip, Benjamin H K Age & Ageing, Jul2021; 50(4): 1093-1101. 9p. (Article - meta analysis, research, systematic review, tables/charts) ISSN: 0002-0729

Subjects: Psychological Tests; Dementia, Senile Diagnosis; Chinese

35. ## Factors influencing length of hospital stay in an acute psychogeriatric unit.

(includes abstract) Koskas, Pierre; Bourdonnec, Lauriane; Kohler, Samuel; Romdhani, Mouna; Drunat, Olivier Psychogeriatrics, Jul2021; 21(4): 478-482. 5p. (Article - research, tables/charts) ISSN: 1346-3500

Subjects: Length of Stay Psychosocial Factors; Acute Care; Geriatric Psychiatry; Dementia, Senile; Socioeconomic Factors; Cognition Evaluation; Body Weight; Aged: 65+ years; Aged, 80 & over; Female; Male

36. ## The provision of person-centred dementia care in the context of mental health co-morbidities: 'It can be upsetting and distressing and it's incredibly sad'.

(includes abstract) McKenzie, Ellen L.; Brown, Patricia M. Australasian Journal on Ageing, Jun2021; 40(2): e133-e142. 10p. (Article - research, tables/charts) ISSN: 1440-6381

Subjects: Patient Centered Care; Gerontologic Care; Dementia, Senile; Attitude of Health Personnel; Mental Health; Comorbidity; Adult: 19-44 years; Middle Aged: 45-64 years; Male; Female

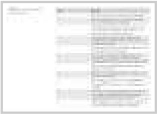
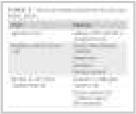

PDF Full Text

37. Antipsychotic Initiation Among Older Dementia Patients Using Cholinesterase Inhibitors: A National Retrospective Cohort Study.

(includes abstract) Rege, Sanika; Carnahan, Ryan M.; Johnson, Michael L.; Chen, Hua; Holmes, Holly M.; Aparasu, Rajender R. Drugs & Aging, Jun2021; 38(6): 493-502. 10p. (Article - research, tables/charts) ISSN: 1170-229X

Subjects: Risk Assessment; Antipsychotic Agents Therapeutic Use; Behavioral Symptoms Drug Therapy; Dementia, Senile Drug Therapy; Cholinesterase Inhibitors Therapeutic Use; Donepezil Therapeutic Use; Rivastigmine Therapeutic Use; Galanthamine Therapeutic Use; Aged: 65+ years; Aged, 80 & over

38. The extra costs associated with a cognitive impairment: Estimates from 15 OECD countries.

(includes abstract) Morris, Zachary A; Zaidi, Asghar; McGarity, Stephen European Journal of Public Health, Jun2021; 31(3): 647-652. 6p. (Article - research, tables/charts) ISSN: 1101-1262

Subjects: Cognition Disorders Economics; Dementia, Senile Economics; Economic Aspects of Illness; Organisation for Economic Co-Operation and Development; Aged: 65+ years

HTML Full Text **PDF Full Text**

39. Community participation in activities and places among older adults with and without dementia.

(includes abstract) Chaudhury, Habib; Mahal, Tanveer; Seetharaman, Kishore; Nygaard, Haakon B. Dementia (14713012), May2021; 20(4): 1213-1233. 21p. (Article - research, tables/charts) ISSN: 1471-3012

Subjects: Dementia, Senile; Consumer Participation; Aged: 65+ years

40. Reversion to the original concept of clinical practice for patients with dementia: towards an appropriate intervention for a favourable prognosis.

(includes abstract) Meguro, Kenichi Psychogeriatrics, May2021; 21(3): 447-448. 2p. (Article - letter, tables/charts) ISSN: 1346-3500

Subjects: Alzheimer's Disease Therapy; Psychosocial Intervention; Alzheimer's Disease Drug Therapy; Combined Modality Therapy; Alzheimer's Disease Prognosis

41. Hearing Loss Linked With Increased Risk for Dementia.

(includes abstract) ASHA Leader, Apr2021; 26(3): 14-14. 1p. (Article - pictorial) ISSN: 1085-9586

Subjects: Hearing Disorders Complications; Dementia, Senile Risk Factors; Mild Cognitive Impairment Risk Factors; Neuropsychological Tests; Risk Assessment; Aged: 65+ years

PDF Full Text

42. Dual Trajectories of Dementia and Social Support in the Mexican-Origin Population.

(includes abstract) Rote, Sunshine M; Angel, Jacqueline L; Kim, Jiwon; Markides, Kyriakos S Gerontologist, Apr2021; 61(3): 374-382. 9p. (Article - research, tables/charts) ISSN: 0016-9013

Subjects: Support, Psychosocial; Activities of Daily Living; Dementia, Senile Risk Factors; Hispanic Americans Psychosocial Factors; Risk Assessment; Aged: 65+ years; Aged, 80 & over; Female; Male

43. Rehabilitation of persons with dementia: using technology to improve participation.

(includes abstract) Zarit, Steven H.; Chiusano, Carrie; Harrison, Aime S.; Sewell, Lynne; Krause, Christopher; Liu, Yin Aging & Mental Health, Mar2021; 25(3): 543-550. 8p. (Article - clinical trial, research, tables/charts) ISSN: 1360-7863

Subjects: Dementia, Senile Rehabilitation; Rehabilitation, Geriatric Methods; Therapy, Computer Assisted; Treatment Outcomes Evaluation; Aged: 65+ years; Aged, 80 & over; Female; Male

Show all 6 Images

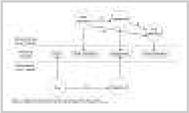
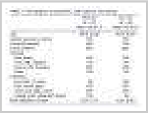
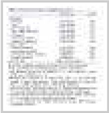
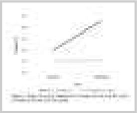

HTML Full Text **PDF Full Text**

44. 智慧医学在老年痴呆病人护理中的研究 进展.

(includes abstract) 燕杏钰; 刘艳丽; 王沙沙; 郭 蔚; 卢 霞 Chinese Nursing Research, 2021; 35(3): 454-457. 4p. (Article - review) ISSN: 1009-6493

Subjects: Dementia, Senile Nursing; Dementia Patients; Nursing Care; Telemedicine Utilization

45. High-intensity functional exercise in older adults with dementia: A systematic review and meta-analysis.

(includes abstract) Yeh, Shu-Wei; Lin, Li-Fong; Chen, Hung-Chou; Huang, Li-Kai; Hu, Chaur-Jong; Tam, Ka-Wai; Kuan, Yi-Chun; Hong, Chien-Hsiung Clinical Rehabilitation, Feb2021; 35(2): 169-181. 13p. (Article - meta analysis, research, systematic review, tables/charts) ISSN: 0269-2155

Subjects: High-Intensity Interval Training In Old Age; Exercise Intensity In Old Age; Dementia, Senile Rehabilitation; Therapeutic Exercise; Aged: 65+ years

46. The effect of PARO robotic seals for hospitalized patients with dementia: A feasibility study.

(includes abstract) Kelly, Patricia A.; Cox, L. Annette; Petersen, Sandra F.; Gilder, Richard E.; Blann, Amy; Autrey, Ashley E; MacDonell, Kathryn Geriatric Nursing, Jan2021; 42(1): 37-45. 9p. (Article - clinical trial, research, tables/charts) ISSN: 0197-4572

Subjects: Robotics; Dementia, Senile Therapy; Hospitalization; Treatment Outcomes; Interpersonal Relations; Physiological Processes; Aged: 65+ years; Aged, 80 & over; Male; Female

47. THE LATEST IN... NUTRITION. Soy Products Linked to Reduced Dementia Risk.

(includes abstract) Good Medicine, Winter2021; 30(1): 5-5. 1/3p. (Article - brief item, pictorial) ISSN: 1072-8503

48. Caffeine Consumption and Behavioral Symptoms in Nursing Home Residents: A Cross-Sectional Analysis.

(includes abstract) Kromhout (Michelle), M. A.; Rius Ottenheim, N.; Putter, H.; Numans, M. E.; Achterberg, W. P. Journal of Nutrition, Health & Aging, Jan2021; 25(1): 100-107. 8p. (Article - research, tables/charts) ISSN: 1279-7707

Subjects: Nursing Home Patients; Caffeine; Behavioral Symptoms In Old Age; Dementia, Senile; Fluid Intake; Aged: 65+ years

49. Do Caregiving Factors Affect Hospitalization Risk Among Disabled Older Adults?

(includes abstract) Amjad, Halima; Mulcahy, John; Kasper, Judith D.; Burgdorf, Julia; Roth, David L.; Covinsky, Ken; Wolff, Jennifer L. Journal of the American Geriatrics Society, Jan2021; 69(1): 129-139. 11p. (Article - research, tables/charts) ISSN: 0002-8614

Subjects: Caregiver Support; Risk Assessment; Hospitalization; Persons with Disabilities In Old Age; Dementia, Senile; Aged: 65+ years; Male; Female

50. Dementia and Falls Management in Underserved Populations: The Cognition and Mobility Care Management Program.

(includes abstract) Tan, Zaldy S.; Hamade, Wael; Menkin, Josephine; Pacheco, Renee Guillen; Gans, Daphna; Weintraub, Nancy; Garcia, Maribel; Guerrero, Lourdes R.; Reuben, David B. Journal of the American Geriatrics Society, Jan2021; 69(1): 210-215. 6p. (Article - algorithm, research, tables/charts) ISSN: 0002-8614

Subjects: Dementia, Senile Therapy; Accidental Falls Risk Factors; Cognition Disorders; Medically Underserved; Program Implementation; Aged: 65+ years; Aged, 80 & over; Male; Female

EBSCO Connect | Privacy Policy | A/B Testing | Terms of Use | Copyright | Cookie Policy

powered by EBSCO*host*

© 2022 EBSCO Industries, Inc. All rights reserved.

Cochrane Library

Access provided by: **University of Toronto Library**

Advanced Search

Search manager

Save this search | View/Share saved searches | ? Search help

Cochrane Search_TCP Systematic Review_July 15, 2021_Updated on July 9, 2022

Last saved on: 09/07/2022 17:54:54

✔ Search saved.

View fewer lines | Print search history

#	Search		Results
#1	(Transition* NEAR/3 (care* or unit* or bed* or program* or ward* or setting* or facilit* or service* or model* or centre* or center*)):ti,ab,kw	Limits	1835
#2	[mh ^"Transitional care"]	Limits	83
#3	(Intermediate NEAR/3 (unit* or care or bed* or program* or ward* or setting* or facilit* or service* or model* or centre* or center*)):ti,ab,kw	Limits	453
#4	[mh ^"Intermediate care facilities"]	Limits	14
#5	((Subacute or sub-acute) NEAR/3 (unit* or care or bed* or program* or ward* or setting* or facilit* or service* or model* or centre* or [illegible]	Limits	301
#6	[mh ^"Subacute care"]	Limits	22
#7	((Postacute or post-acute) NEAR/3 (unit* or care or bed* or program* or ward* or setting* or facilit* or service* or model* or centre* or center*)):ti,ab,kw	Limits	292
#8	((Post acute) NEAR/3 (unit* or care or bed* or program* or ward* or setting* or facilit* or service* or model* or centre* or center*)):ti,ab,kw	Limits	13424
#9	((Skilled Nursing) NEAR/3 (unit* or bed* or program* or ward* or setting* or facilit* or service* or model* or centre* or center*)):ti,ab,kw	Limits	3312
#10	[mh ^"Skilled nursing facilities"]	Limits	82
#11	(Restor* NEAR/3 (unit* or care or bed* or program* or ward* or setting* or facilit* or service* or model* or centre* or center*)):ti,ab,kw	Limits	441
#12	(Convalesc* NEAR/3 (unit* or care or bed* or program* or ward* or setting* or facilit* or service* or model* or centre* or center* or home* or hospital*)):ti,ab,kw	Limits	227
#13	[mh ^"Convalescence"]	Limits	158
#14	{or #1-#13}	Limits	19396
#15	(Old* or aged or aging):ti,ab,kw	Limits	619418
#16	(Centenarian* or nonagenarian* or octogenarian* or geriatr* or gerontol* or senescen* or septuagenarian* or pensioner* or senile):ti,ab,kw	Limits	12180
#17	Senior*:ti,ab,kw	Limits	4502
#18	Elder*:ti,ab,kw	Limits	55749
#19	[mh ^"Aged"]	Limits	220092
#20	[mh ^"Aged, 80 and over"]	Limits	56060
#21	{or #15-#20}	Limits	643072
#22	(Cognit* NEAR/3impair*):ti,ab,kw	Limits	89168
#23	(Mild neurocognitive disorder*):ti,ab,kw	Limits	230

#24	[mh ^"Cognitive dysfunction"]	Limits	2220
#25	(Major neurocognitive disorder*):ti,ab,kw	Limits	604
#26	Dement*:ti,ab,kw	Limits	15164
#27	[mh ^"Dementia"]	Limits	2778
#28	[mh ^"Alzheimer disease"]	Limits	3767
#29	Alzheimer*:ti,ab,kw	Limits	12454
#30	[mh ^"AIDS Dementia Complex"]	Limits	79
#31	[mh ^"Dementia, Vascular"]	Limits	302
#32	[mh ^"Dementia, Multi-Infarct"]	Limits	68
#33	[mh ^"Lewy body disease"]	Limits	106
#34	Deliri*:ti,ab,kw	Limits	4448
#35	[mh ^"Delirium"]	Limits	873
#36	{or #22-#35}	Limits	103267
#37	#14 and #21 and #36	Limits	1322
#38	#14 and #21 and #36<br>with Cochrane Library publication date from Jul 2021 to Jul 2022	Limits	121
#39	Type a search term or use the S or MeSH buttons to compose	S MeSH Limits	N/A

Clear all

Highlight orphan lines

Save this search | View/Share saved searches | ? Search help

Cochrane Search_TCP Systematic Review_July 15, 2021_Updated on July 9, 2022

Last saved on: 09/07/2022 17:54:54

✔ Search saved.

View fewer lines | Print search history

Filter your results

Cochrane Reviews	Cochrane Protocols	Trials	Editorials	Special Collections	Clinical Answers	More
4	0	117	0	0	0	

4 Cochrane Reviews matching **"#38 - #14 and #21 and #36" with Cochrane Library publication date Between Jul 2021 and Jul 2022**

Did you mean: *Rand* | *band* | *hand*

Cochrane Database of Systematic Reviews

Issue 7 of 12, July 2022

Select all (4) Export selected citation(s) Show all previews

Order by Relevancy Results per page 25

1

Non-pharmacological measures implemented in the setting of long-term care facilities to prevent SARS-CoV-2 infections and their consequences: a rapid review

Jan M Stratil, Renke L Biallas, Jacob Burns, Laura Arnold, Karin Geffert, Angela M Kunzler, Ina Monsef, Julia Stadelmaier, Katharina Wabnitz, Tim Litwin, Clemens Kreutz, Anna Helen Boger, Saskia Lindner, Ben Verboom, Stephan Voss, Ani Movsisyan

Rapid Review 15 September 2021 Free access

Show PICOs ▾ Show preview ▾

2 ☐

Montreal Cognitive Assessment for the detection of dementia

Daniel HJ Davis, Samuel T Creavin, Jennifer LY Yip, Anna H Noel-Storr, Carol Brayne, Sarah Cullum

Diagnostic Review 13 July 2021

Show preview ▾

3 ☐

Informant Questionnaire on Cognitive Decline in the Elderly (IQCODE) for the detection of dementia within a secondary care setting

Jennifer K Burton, Patricia Fearon, Anna H Noel-Storr, Rupert McShane, David J Stott, Terry J Quinn

Diagnostic Review 19 July 2021

Show preview ▾

4 ☐

Music therapy for autistic people

Monika Geretsegger, Laura Fusar-Poli, Cochavit Elefant, Karin A Mössler, Giovanni Vitale, Christian Gold

Intervention Review 9 May 2022 New search Conclusions changed

Show PICOs ▾ Show preview ▾
