# Supplementary material for: The impact of facility-based transitional care programs on function and discharge destination for older adults with cognitive impairment: a systematic review
Source: BMC Geriatr. 2022 Nov 14;22:854. doi: 10.1186/s12877-022-03537-y (PMC9661763; doi:10.1186/s12877-022-03537-y)
Supplement: Supplementary file 3 — Additional file 3: Table S1. Risk of Bias Assessment for Cohort Studies; Table S2. Risk of Bias Assessment for Cross-Sectional Studies; Table S3. Characteristics of Included Studies; Table S4. Patient Outcomes; Table S5. Health Services Outcomes. [file 12877_2022_3537_MOESM3_ESM.docx]

**Additional file 3**

**Table S1.** Risk of Bias Assessment for Cohort Studies

| **Author Year** | **Checklist Question Number** | | | | | | | | | | | **Score (%)** | **Study Quality** |
| --- | --- | --- | --- | --- | --- | --- | --- | --- | --- | --- | --- | --- | --- |
|  | **1** | **2** | **3** | **4** | **5** | **6** | **7** | **8** | **9** | **10** | **11** |  |  |
| Abrahamsen 2016 | NA | NA | Y | Y | Y | Y | Y | Y | U | N | Y | 78 | Moderate |
| Burke 2021 | Y | Y | Y | Y | Y | Y | Y | Y | Y | N | Y | 91 | Good |
| Cations 2020 | NA | NA | Y | Y | Y | Y | Y | Y | Y | N | Y | 89 | Good |
| Chong 2012 | NA | NA | Y | N | N | Y | Y | Y | Y | Y | U | 67 | Moderate |
| Downer 2022 | Y | Y | Y | Y | Y | Y | Y | Y | Y | N | Y | 91 | Good |
| Kosar 2017 | NA | NA | Y | Y | Y | Y | Y | Y | Y | Y | Y | 100 | Good |
| Lee 2008 | NA | NA | Y | Y | Y | Y | Y | Y | Y | N | Y | 89 | Good |
| Lee 2011 | NA | NA | Y | N | N | Y | Y | Y | N | N | N | 50 | Fair |
| Lei 2022 | Y | Y | Y | Y | Y | Y | Y | Y | U | N | Y | 80 | Good |
| Loomer 2019 | NA | NA | Y | Y | Y | Y | Y | Y | N | N | Y | 78 | Moderate |
| Lueckel 2018 | NA | NA | Y | Y | Y | Y | Y | Y | U | N | Y | 78 | Moderate |
| Madrigal 2021 | Y | Y | Y | Y | Y | Y | Y | Y | Y | N | Y | 82 | Good |
| Marcantonio 2003 | NA | NA | Y | Y | Y | Y | Y | Y | N | N | Y | 78 | Moderate |
| Marcantonio 2005 | NA | NA | Y | Y | Y | Y | Y | Y | N | N | Y | 78 | Moderate |
| Mazzola 2022 | NA | NA | Y | N | N | Y | Y | Y | N | N | Y | 60 | Moderate |
| Miu 2016 | NA | NA | Y | Y | Y | Y | Y | Y | U | N | Y | 78 | Moderate |
| Simning 2022 | Y | Y | Y | Y | Y | Y | Y | Y | Y | N | Y | 91 | Good |
| Wysocki 2015 | NA | NA | Y | Y | Y | Y | Y | Y | Y | N | Y | 89 | Good |

Y=yes; N=no; NA=not applicable; U=unclear.

Column numbers correspond to the following checklist questions from the JBI Risk of Bias Assessment for Cohort Studies: 1=Were the two groups similar and recruited from the same population?; 2= Were the exposures measured similarly to assign people to both exposed and unexposed groups? 3= Was the exposure measured in a valid and reliable way?; 4= Were confounding factors identified?; 5= Were strategies to deal with confounding factors stated?; 6= Were the groups/participants free of the outcome at the start of the study (or at the moment of exposure)?; 7= Were the outcomes measured in a valid and reliable way?; 8=Was the follow up time reported and sufficient to be long enough for outcomes to occur?; 9= Was follow up complete, and if not, were the reasons to loss to follow up described and explored?; 10= Were strategies to address incomplete follow up utilized?; 11= Was appropriate statistical analysis used?

Study Quality: Fair <50% of checklist items given a rating of yes; moderate 51-80% of items given a rating of yes; good >80% of items given a rating of yes (Benenson et al., 2021). Items with a rating of NA were excluded from the score calculation.

**Table S2.** Risk of Bias Assessment for Cross-Sectional Studies

| **Author Year** | **Checklist Question Number** | | | | | | | | **Score (%)** | **Study Quality** |
| --- | --- | --- | --- | --- | --- | --- | --- | --- | --- | --- |
|  | **1** | **2** | **3** | **4** | **5** | **6** | **7** | **8** |  |  |
| Bardenheier 2021 | Y | Y | Y | Y | Y | Y | Y | Y | 100 | Good |
| Hang 2021 | Y | Y | Y | Y | NA | NA | Y | Y | 100 | Good |
| Intrator 2021 | Y | Y | Y | Y | Y | Y | Y | Y | 100 | Good |
| Nakanishi 2016 | Y | Y | Y | Y | Y | Y | Y | Y | 100 | Good |

Y=yes; N=no.

Column numbers correspond to the following checklist questions from the JBI Risk of Bias Assessment for Cross-Sectional Studies. : 1= Were the criteria for inclusion in the sample clearly defined?; 2= Were the study subjects and the setting described in detail?; 3= Was the exposure measured in a valid and reliable way?; 4= Were objective, standard criteria used for measurement of the condition?; 5= Were confounding factors identified?; 6= Were strategies to deal with confounding factors stated?; 7= Were the outcomes measured in a valid and reliable way?; 8= Was appropriate statistical analysis used?

Study Quality: Fair <50% of checklist items given a rating of yes; moderate 51-80% of items given a rating of yes; good >80% of items given a rating of yes (Benenson et al., 2021). Items with a rating of NA were excluded from the score calculation.

**Table S3.** Characteristics of Included Studies

| **Author Year Setting**  **Country** | **Study Design** | **Study Purpose** | **Sample Size** | **Staff Complement** | **Description of TCP Services** | **Inclusion Criteria** | **Exclusion Criteria** |
| --- | --- | --- | --- | --- | --- | --- | --- |
| Abrahamsen 2016  Post-acute intermediate care unit in a nursing home  Norway | Prospective, observational cohort | To investigate the role and predictive value of 1) different admission diagnoses and 2) the degree of functional loss, assessed by BI, on the recovery potential of older patients after acute hospitalization | Participants with CI: n=206 (29% of total in study) | Increased multidisciplinary staffing (2 fulltime physicians, one of whom is a geriatrician, 15 nurses, 1.2 positions for physiotherapists, and 0.8 positions for an occupational therapist. | 19-bed unit. Provides treatment and rehabilitation for elderly people within a few days after acute hospitalization | 1) > 70 years, home dwelling in the municipality of Bergen and considered to be respiratory and circulatory stable; 2) hospital doctor expected return home within 2 weeks of treatment in unit; 3) no major cognitive impairment or delirium (based on judgement of hospital doctor) | NR |
| Bardenheier 2021  SNFs  USA | Retrospective cross-sectional trend study | To compare  the post-acute and long-term care experience of Medicare  beneficiaries with and without Alzheimer Disease and  Related Dementias (ADRD), and whether differences changed from January 1, 2007 to September 30, 2015. | Total number of persons with ADRD:  n=2134798 | NR | NR | 1) Fee-for-service Medicare beneficiaries  aged ≥66 years; 2) discharged alive from the hospital; 3) admitted to a SNF within 1 day of hospital discharge. | 1) History of being in a nursing home within the 12 months (using the MDS) before hospital admission because they were likely to be sicker and/or more severely demented. |
| Burke 2021  SNFs  USA | Retrospective observational study | To assess both ostensible potential “benefits”  (improvement in physical function, ability to successfully  return home) and “risks” (hospital readmission and mortality  during SNF stay) of post-acute care in SNFs for  Medicare beneficiaries with a diagnosis of dementia, and compare these to similar Medicare beneficiaries without a diagnosis of dementia | Total number with dementia: 830,524 (34.3%) | NR | SNF post acute care is paid by Medicare for skilled care (including nursing and therapy services) and is intended for recuperation, rehabilitation, and promoting the ability of older adults to return home | 1) Identified Medicare beneficiaries hospitalized  between January 1, 2015, and December 31, 2016, with a  SNF stay starting within 3 days of hospital discharge; 2) only  the first hospitalization followed by SNF stay for each  beneficiary during the study period | 1) Medicare beneficiaries who were younger than age 65; 2) admitted to the hospital from a nursing home, or discharged to a swing bed or hospice; 3) Patients who were diagnosed with dementia after their index hospitalization and SNF care episode |
| Cations 2020  Transition care program in nursing home  Australia | Descriptive cohort study | To examine TCP users  to (a) describe the common outcomes of receiving TCP,  and (b) identify factors associated with TCP ‘success’, defined here as improved functional independence from admission  to discharge, discharge to home, and continued  residence at home at 6-months post-discharge | Individuals in residential care settings with dementia: n=10701 (25.4%) | May include physiotherapy, occupational therapy, social work, nursing, case management | A plan for services is developed by the TCP provider in collaboration with the participant and is regularly reviewed. Services delivered are flexible and customised to the individual, but typically include low-intensity therapies to improve physical, cognitive, and psychosocial functioning. | a) Assessed by an Aged Care Assessment Program assessor; b) an inpatient in a public or private Australian hospital, or receiving  sub-acute care; c) medically stable and ready for discharge from acute or sub-acute care; d) otherwise eligible for residential aged care, and e) have the capacity to benefit from low-intensity therapies over a maximum of 18 weeks, according to clinical judgement. Participants were included in this study if they were a) non-Indigenous; b) aged 65 years or older at TCP entry; and c) used TCP for the first time between January 1, 2007 to December 31, 2015 | a) Aboriginal and Torres Strait Islander older people were not available in the data capture. |
| Chong 2012  Subacute ward in a community hospital  Singapore | Retrospective cohort study | To examine the impact of subacute model of care on the overall length of hospital stay, transitions at the nexus between the hospital and community sectors, patients’ discharge destination and functional performance.  We also looked at the secondary effects of this model on the cost of care (refl ected by the bill size) for inpatients of the department of geriatric medicine in the acute hospital setting prior to and after implementation | Persons with dementia and behavioural disturbances:  n=31 (16.9% of total in study) | The ward is staffed with geriatricians, geriatric trained nurses, allied health specialists, care coordinators and medical social workers to actively address the needs of the frail elderly patients. | The unit is an open concept ward and environment that is more conducive for patients with behavioural disturbances. A non-pharmacologic and pharmacological approach was provided to patients with dementia with challenging neuropsychiatric symptoms (e.g., agitated depression, delusions, agitation, nighttime behaviours, aberrant motor behaviour), with available consultation for medical titration from a psychogeriatrician. | 1) Medically stable, no longer required acute hospital care but still needed continued medical management of their illness; 2) Deemed to benefit from short course of functional rehabilitation (1 to 2 weeks) to address deconditioning following their medical illness rather than in a community hospital setting; 3) medically stable and awaiting a bed  at the community hospital facility;  4) Admitted for behavioural problems  or had concomitant behavioural problems with their medical illness requiring monitoring and medication titration for manageable behavioural control; 5) needed comprehensive  management to establish discharge care plans via  multidisciplinary meetings | 1) Dangerously ill; 2) on intranasal oxygen (except when patient was on long term therapy and the oxygen requirement had remained stable for the last 48 hours; 3) with unstable parameters; 4) awaiting urgent surgical procedures |
| Downer 2022  SNFs  USA | Retrospective Cohort Study | To leverage newly added information on MDS assessment for prior functioning and investigate if residents’ level of independence in self-care before being admitted to an SNF is associated with their self-care function at SNF admission, discharge, and the change in self-care during an SNF stay | Mild CI: n=120830  Moderate to severe CI: n=74183 | NR | Skilled nursing facilities (SNFs) provide short-term nursing care and rehabilitation services to patients who have been discharged from the hospital | 1) Medicare beneficiaries who were discharged from an SNF between October 1, 2018, and December 31, 2019 | 1) Beneficiaries who were not admitted to an SNF within 3-days of hospital discharge; 2) younger than 66 at the time of SNF discharge; 3) did not have continuous fee-for-service coverage or were enrolled in Medicare Advantage in the year before SNF discharge; 4) Died during the SNF stay; 5) Did not have information for prior functioning in self-care; 6) Did not have complete information for self-care at admission or discharge; 7) Residents missing information for selected characteristics in the MDS |
| Hang 2021  Facility-based transition care program  Australia | Cross-sectional design | To audit a transition care service to identify the association between the characteristics of older adults undertaking a facility-based TCP and (i) discharge destination and (ii) functional improvement. | With CI: n=87 | The facility health professional staffing included a manager,  Registered nurse,  physiotherapist,  social worker,  and an  occupational  therapist.  A general  practitioner,  speech pathologist, nutritionist, and podiatrist visited as required; care assistant staff | 47 bed transition care facility; Care assistant staff also assisted clients with daily personal care. The TCP included physiotherapy for functional and mobility  training, occupational therapy for cognitive activities and home visits, and social work for discharge planning and care support at home. | 1) Older adults aged 60 years and above; 2) admitted to the facility to undertake a TCP during the study period | 1) older adult was admitted for palliative care, or for <2 weeks duration. |
| Intrator 2021  Post acute care in CLCs (VA Nursing homes)  USA | Cross-sectional study | To compare the VA Community Living Centers (CLCs) to nursing homes in the community (NHs) in terms of characteristics of their post acute populations and performance on 3 claims-based (“short-stay”) quality measures. | With dementia: n=1091  No/mild CI: n=9808  Moderate/high CI: n=4979 | Average Nurse Staffing Hours per Bed Day in Fiscal Year 2016  Mean Hours per Bed Day (SD)  RN: 2.45 (7.99)  LPN/LVN: 1.63 (7.19)  Aide: 2.80 (8.52) | NR | 1) CLC and nursing home resident admissions directly from any VA hospital or VA-paid hospital for acute care (not rehabilitation or long-term care hospitals), between July 1, 2015, and June 30, 2016 | 1) Residents identified in the MDS as comatose; 2) stays that overlapped hospice care |
| Kosar 2017  Post acute care in the nursing home setting  USA | Retrospective cohort study | To identify the rate of delirium present during admission to postacute care (PAC) in the nursing home setting and to determine whether patients with delirium had higher risk for adverse outcomes | Participants with delirium:  n=242121 | NR | NR | I) Admitted to US nursing homes for PAC; 2) aged ≥65 years; 3) without prior history of nursing home residence | 1) Having any prior MDS admission assessment |
| Lee 2008  SNFs  USA (Midwestern region) | Retrospective correlational design | To examine the relationship between  health-related admission factors and post-hospital physical  function at 3, 6, 9 and 12 months in older adult nursing  facility residents | Total sample size 38591  Number of participants with CI not reported | NR | NR | 1) ≥65 years; 2) Admitted to shorty-stay skilled nursing units from acute care hospitals as indicated by MDS information, admission assessment and Medicare 5day assessment. | NA |
| Lee 2011  Community hospital-based PAC unit  Taiwan | Prospective Cohort Study | To quantitatively evaluate functional gain among elderly patients receiving community hospital-based PAC services in Taiwan so as to provide a baseline profile to support subsequent research in evaluating the impact of short-term functional gain on long-term clinical outcomes | Participants with dementia: n=139 | Case manager (a senior nurse), one PT/OT per four patients, one dietician and social worker for all patients, one nursing staff per 10 patients. | Physical Reablement program:  Case manager (a senior nurse) completed a comprehensive geriatric assessment (CGA) within 72 hours; exercise (with PT) and assistance for ADL training (with OT) once daily, 5 days a week; nutrition consultation, pharmacist (medication reconciliation) on admission, revisit per 2 weeks and PRN visit; social worker visit on admission and PRN;  nursing staff provided essential (function) nursing assessments, setting nursing goals and nursing care plans with functional goals at the individual and team level.  Dose/frequency/duration of services provided: 45-60 minutes physiotherapy sessions, once daily, 5 days a week for 4 weeks. 45-60 minutes OT sessions, once daily, 5 days a week for 4 weeks. The treatment course could be extended to a maximum of 12 weeks, if the interdisciplinary team agreed. Patients requiring more than 12 weeks of treatment would be transferred to LTC facilities for continuing care. | 1) Aged >65 years; 2) medically stable, requiring no intensive medical, laboratory, or oxygen support; 3) presence of acute functional decline during hospitalizations. | 1) Admission for elective procedures; 2) acute conditions related to terminal illness; 3) malignancy; 4) patients who were considered to have a low potential for functional recovery |
| Lei 2022  PAC  USA | Retrospective cohort study | To estimate the impact of  continuity of care (COC) on successful community discharge after hospitalization | 8317 veterans with dementia who were first discharged to a PAC  facility and then to the community | NR | NR | 1) Veterans with dementia; 2)  ≥66 years old; 3) had any acute inpatient medical or surgical hospitalization from the community between October  2014 to August 2015 with only the first hospitalization  in this period included | 1) Veterans not enrolled in Medicare or who were enrolled in Medicare Advantage due to  incomplete data; 2) veterans continually in nursing home for >90 days in FY 2014 (considered to not  be community dwelling); with <3 selected outpatient visits because the COC measure was less reliable with few visits; 3) veterans who died in hospital or who  died after discharge to post-acute care (PAC) facilities  (nursing homes and rehabilitation centers; 4) veterans discharged to community with outpatient  hospice; 5) veterans discharged to nursing homes for longterm  care; 6) veterans  residing in Puerto-Rico and the Virgin-Islands |
| Loomer 2019  SNFs  USA | Retrospective analysis | To examine the association between residents’ cognitive status on admission and change in self-care and mobility during a Medicare-covered SNF stay | Total number of participants with CI: n=78160 | NR | NR | 1. Medicare Part A beneficiaries who had a PPS stay between January 1, 2017, and June 30, 2017, but had not been in a NH in 2016 | 1) Had incomplete information on any independent variables; 2) had different Medicare stay and facility entry dates; 3) were in the NH less than 3 days; 4) were younger than 21 years; 5) did not receive any physical or occupational therapy; 6) were comatose; 7) were discharged to hospice or died; 8) had an unplanned discharge |
| Lueckel 2018  SNFs  USA | Retrospective cohort study | To describe the outcomes of  patients with traumatic brain injury (TBI) who are admitted to SNFs for postacute care  by key demographic and clinical characteristics | Total number of participants with CI: n=47469 | NR | NR | 1) Hospitalized Medicare beneficiaries with an active diagnosis of TBI who were discharged directly to an SNF between January 1, 2011 and December 31, 2014 | 1) < 65 years of age; 2) had prior postacute SNF use within 1 year of the index hospitalization |
| Madrigal 2021  Community-based SNF  USA | Retrospective Cohort Study | To assess the association of delirium with 30-day functional improvement in patients discharged to SNFs after heart failure hospitalization | Participants with delirium: n=882 | NR | NR | 1) Veterans from 129 VA medical centers hospitalized with a primary diagnosis of heart failure; 2) discharged to a community based SNF outside the VA Health System | 1) Received palliative or hospice care services before, during, or at the hospital discharge; 2) if the only follow-up minimum data set (MDS) 3.0 assessment occurred after the 90-day assessment interval because the scope of the study focused on short-term functional recovery; 3) if there was incomplete data (e.g. 2 or more MDS assessments) |
| Marcantonio 2003  SNFs  USA | Prospective cohort study | To determine the prevalence of delirium  symptoms at the time of admission to post-acute facilities, the persistence of delirium symptoms in this setting, and the association of delirium symptoms with functional recovery | Participants with delirium symptoms:  n=126 | NR | NR | 1) Aged ≥65 years; 2) newly admitted to participating post-acute care facilities from acute care hospitals | 1) Aged <65 years |
| Marcantonio 2005  Seven SNFs that specialize in postacute care  USA | Observational cohort study | to examine 30-day postacute facility outcomes and 6-month mortality in a nested cohort of subjects from the above study9 who met the criteria for delirium, subsyndromal delirium, or no delirium at the time of admission to postacute care | Total number of persons with CI: n=434 | NR | NR | 1) With delirium and with available medical records; 2) aged ≥65 years; 3) admitted directly from an acute medical-surgical hospitalization | 1) Inability to speak English, 2) hearing impairment  that precluded interview; 3) severe communication or functional impairment before acute illness; 4) admission for terminal care (life expectancy of 6 months); 5) residence outside a 25 mile radius from the research site |
| Mazzola 2022  Subacute Care Units  Italy | Retrospective observational cohort study | report the clinical characteristics  and outcomes of a cohort of patients residents in  Lombardy region admitted to an SCU during a 3-year  period. Since returning home after discharge is the principal  objective of the SCU model of care, we also aim to  identify the factors potentially associated with the lack of  returning to home of these patients | With dementia: n=98  With delirium: n=58 | 1 physician acting as a director; 2 geriatricians; 1 resident in geriatric medicine; 7 nurses; other auxiliary personnel | A 10-bed unit service managed by a team. Overall, nursing staff was assigned to work in such a way that each patient was allotted at least 180 min of direct nursing care per day. | 1) Should neither require intensive cardio-respiratory monitoring nor have drugs administered in continuous infusion; 2) Should not have “life-threatening” arrhythmias; 3) Should have independent breathing for at least 48h (even if with oxygen support). The presence of a tracheotomy is not a contraindication; 4) Should neither have sepsis nor acute single or multiorgan  failure; 5) Should not require a long-term use of nasogastric tube  or percutaneous endoscopic gastrostomy; 6) Should not be on a waiting list for planned surgery; 7) Should give informed consent for admission to the SCU | NA |
| Miu 2016  Post-acute convalescence units  Hong Kong | Prospective cohort study | To investigate the prevalence  and the characteristic associated factors in elderly patients admitted to post-acute care facilities. The secondary objective was to investigate the effect of delirium persistence on mobility status and placement | Total number of participants with CI: n=109 | Staff mix per ward with 43 beds:  Morning shift: 5 nurses, 3.7 PCWs; fternoon shift: 4 nurses, 3 PCWs; Night shift: 2 nurses, 1 PCW.  1 OT; 1 PT. | Provides extended care and rehabilitation service to patients transferred from a regional acute hospital in Hong Kong: 2 hours daily, 5 days per week of mobility and ADL training. | 1) Aged >65 years; 2) no serious hearing impairment; 3) were communicable before acute illness; 4) not admitted for terminal care | 1) Admitted for terminal care |
| Nakanishi  2016  Geriatric ICFs  Japan | Retrospective study design for data from a nationally representative cross-sectional survey | To investigate the association between dementia and the discharge destination of patients in geriatric intermediate care facilities under the public LTCI program in Japan | Participants who had dementia:  n=2483 | At each geriatric ICF: at least 1 full-time physician, 9 nurses, 1 rehabilitation staff (PT, OT, or speech therapist) per 100 residents | Provided intermediate care for older adult patients who require rehabilitation, nursing, and are aimed at discharge to home within 3 months. | 1) Patients who were discharged from facilities selected for the SIEL survey | 1) Patients were excluded from the SIEL patient survey when they were covered by respite care under the LTCI home care services |
| Simning 2022  SNFs  USA | Retrospective  cohort study based on secondary data analyses | To examine how mental illness (MI) and Alzheimer’s disease and  related dementias (ADRD) were associated with whether skilled nursing facility  (SNF) residents returned to and remained in the community and if receipt of home  health services was associated with post-SNF home time | With dementia: n=10426 | NR | For post-acute care and rehabilitation. | 1) New York State (NYS) fee for-  service Medicare beneficiaries aged 65 years and  older; 2) wih an index NYS SNF admission in 2014 following  a hospitalization of three or more days | NR |
| Wysocki 2015  Post-acute care in nursing homes  USA | Retrospective analysis | To examine the completeness of the activities of daily living (ADL) items on admission and discharge assessments and the improvement in ADL performance among short-stay residents in the newly adopted MDS 3.0 | Participants with CI (moderately impaired, severely impaired, dementia, and any signs of delirium) total: n = 397,284 | NR | For persons who require nursing and rehabilitation services after a hospitalization. Stays are short as persons recuperate and prepare to go back to their primary residence. | 1) Residents with admission and discharge assessments between July 1, 2011 and June 30, 2021 who were not new admissions from an acute hospital; 2) Length of stay 100 days or less. | 1) Individuals who were comatose or receiving hospice at admission; 2) Individuals who died in the nursing home within the 100-day time period were therefore excluded because they did not have a discharge assessment |

CI=cognitive impairment; SNFs=Skilled nursing facilities; NH=Nursing home; ADL=Activities of daily living; MDS=Minimum Data Set; PAC= Post acute care; MDS-PAC=Minimum Data Set for Post Acute Care; IADL=Instrumental activities of daily living; NDI=National Death Index; mBI=modified Barthel Index; ADRD=Alzheimer's Disease and related dementias; MDS=Minimum Data Set; OT=Occupational therapist; PT=Physiotherapist; ADL=Activities of daily living; SIEL= Survey of Institutions and Establishments for Long-Term Care; LTCI=Long-Term Care Insurance program; PRN=pro re nata (as needed); PCW=Personal care worker; LTC=Long Term Care; PPS=Prospective Payment System; NH=Nursing home; ICFs=Intermediate care facilities; VA=Veterans Affairs; TBI=traumatic brain injury; PAC=post acute care; SCU=subacute care units; RN=Registered Nurse; LPN=Licensed Practical Nurse; LVN=Licensed Vocational Nurse; CLC=Community living centers; VA=Veterans Affairs; SD=standard deviation.

**Table S4.** Patient Outcomes

| **Author Year** | **Outcome Measure** | **Score** | | | | | | |
| --- | --- | --- | --- | --- | --- | --- | --- | --- |
|  |  | **Baseline** | **1 or 2 Weeks** | **4 Weeks /**  **1 Month** | **At Discharge** | **3 Months** | **6 Months** | **1 Year** |
| Bardenheier 2021 | Death within 90 days after discharge from the SNF | NA | NA | NA | NA | Patients with ADRD:  2007: 8.9%  2008: 8.3%  2009: 8.2%  2010: 8.4%  2011: 8.4%  2012: 8.6%  2013: 8.4%  2014: 8.6%  2015: 8.2%  AAPC (95% confidence interval) −0.2 (−1.0, 0.7)  Patients with no ADRD:  2007: 6.3%  2008: 6.2%  2009: 6.1%  2010: 6.0%  2011: 5.9%  2012: 5.9%  2013: 5.7%  2014: 5.9%  2015: 5.7%  AAPC (95% confidence interval)  -1.1 (-1.5, -0.6) | NA | NA |
| Burke 2021 | Mortality | NA | NA | NA | During SNF stay:  N(%)  25,066 (4.9%) | NA | NA | NA |
| Kosar 2017 | Mortality | NA | NA | NA | NA | 33.7% of patients with delirium but no dementia compared to 12.8% of patients with no delirium and no dementia died within 90 days RR 2.15 (2.13, 2.18)  26.2% of person with delirium and dementia compared to 16.9% of persons with dementia but no delirium died at 90 days RR 1.57 (1.55, 1.59) | NA | 49.1% of persons with delirium but no dementia compared to 24.4% of persons with no delirium and no dementia died at 1 year RR 1.71 (1.69,1.72)  46.6% of persons with delirium and dementia compared to 34.8% of persons with dementia but no delirium died at 1 year RR 1.35 (1.34 1.36) |
| Lee 2011 | MMSE | Participants with dementia: 5.9±3.7  No dementia:  13.4 ± 8.1 | NA | Participants with dementia: 8.5±5.2 p<0.001  No dementia:  15.9±8.2  p<0.001 | NA | NA | NA | NA |
|  | GDS | Participants with dementia: 0.7±1.3 p=0.007  No dementia:  1.2 ± 2.1 p<0.001 | NA | Participants with dementia:0.6±1.1 p=0.007  No dementia  0.8 ±1.3  p<0.001 | NA | NA | NA | NA |
|  | MNA | Participants with dementia:  12.8±3.3  No dementia:  16.0 ± 3.9 | NA | Participants with dementia:  16.5±3.6 p<0.001  No dementia:  17.8±3.5  p<0.001 | NA | NA | NA | NA |
| Lei 2022 | Mortality | NA | NA | NA | Within 30 days of discharge: 3% | NA | NA | NA |
| Lueckel 2018 | Mortality | NA |  | Among residents with  cognitive impairment, 11.1% died within 30 days of admission to an SNF compared with 3.2% of those without impairment (RR = 2.55, 99% confidence interval = 2.32, 2.80). | NA | Among residents with cognitive impairment, 21.1% died within 90 days compared to 7.8% without CI (RR 2.16 (2.03, 2.29)) | NA | Among residents with cognitive impairment, 38.8% died within 1 year (RR 1.74 compared to 19.2% for those without CI 99.2% confidence interval (1.68, 1.80). |
| Marcantonio 2005 | 6-month mortality  (Six-month mortality rate across the  three groups was assessed using a Cox proportional hazards  regression model. Kaplan-Meier survival curves were generated) | NA | NA | NA | NA | NA | Mortality rate: Participants with delirium:  25%  Participants with subsyndromal delirium:  18.2%  Participants with no delirium: 5.7%  With participants with no delirium as the reference and adjusting for age, pre-existing dementia, and medical comorbidity, the hazard ratio for 6-month mortality of those with delirium was 5.2 (95% confidence interval (CI) 1.8–14.5) and for subsyndromal delirium was 3.4 (95%  confidence interval)  (1.2–9.5) |  |
| Miu 2016 | Live with family on discharge:  Participants with delirium (n=89) 27.85%  Participants with no delirium (n=172)  72.05%  p<0.001 | NR | NR | Living with family at 1 month:  Participants with Delirium (n=89) 21.05%  Participants with no delirium (n=172) 47.56%  P <0.001 | NR | Living with family at 3 months:  Participants with Delirium (n=89) 24.49%  Participants with no delirium (n=172) 67.92%  p <0.001 | NR | NR |
|  | Presence of Delirium (measured by CAM-CR) | Delirium was present in 89 participants on admission (34.1%) | NR | NR | A total of 70 participants (78.7%) continued to be delirious on discharge. | NR | NR | NR |
|  | Persistence of Delirium (measured by DOS Scale) | NA | NR | 76 patients (85.4%) had persistence of delirium at 1 month | NR | 49 patients (55.1%) continued to be delirious at 3 months. | NR | NR |
|  | Mortality | NR | NR | NR | NR | 28 participants died (10.7%) | NR | NR |

AAPC=Average annual percentage change; CI=Confidence interval; GDS=Geriatric depression scale, MMSE= Mini-Mental State Examination; MNA=Mini Nutritional Assessment; ADRD=Alzheimer’s disease and related dementias; CAM-CR=Chinese version of CAM; DOS=Delirium observation screening scale

**Table S5.** Health Services Outcomes

| **Author Year** | **Outcome** | **Score/Value** | | | | |
| --- | --- | --- | --- | --- | --- | --- |
|  |  | **Baseline** | **At discharge** | **At 30 days** | **At 3 months** | **At 6 months** |
| Bardenheier 2021 | Rehospitalization within 30 days of hospital discharge  (by year) | NA | NA | Patients with ADRD:  Year, %  2007, 16.0%  2008, 16.4%  2009, 16.1%  2010, 15.8%  2011, 15.3%  2012, 14.5%  2013, 13.8%  2014, 13.5%  2015, 13.4%  AAPC (95% CI) −2.8 (−3.6, −2.0)  Patients with no ADRD:  Year, %  2007, 16.5%  2008, 16.8%  2009, 16.4%  2010, 16.1%  2011, 15.5%  2012, 14.7%  2013, 14.0%  2014, 13.8%  2015, 13.9%  AAPC (95% CI) −2.9 (−3.6, −2.1) | NA | NA |
|  | Re-hospitalization within 90 days of hospital discharge (by year) | NA | NA | NA | Patients with ADRD:  Year, %  2007, 27.4%  2008, 27.8%  2009, 27.4%  2010, 27.3%  2011, 26.6%  2012, 25.9%  2013, 24.8%  2014, 24.8%  2015, 24.6%  AAPC (95% CI) −1.7 (−2.3, −1.2)  Patients with no ADRD:  Year, %  2007, 26.8%  2008, 27.2%  2009, 26.7%  2010, 26.3%  2011, 25.5%  2012, 24.6%  2013, 23.6%  2014, 23.5%  2015, 23.6%  AAPC (95% CI) −2.1 (−2.7, −1.5) |  |
| Burke 2021 | Hospital Readmission | NA | During SNF stay:  82,259 (16.0%) | NA | NA | NA |
| Cations 2020 | Adjusted subdistribution hazard ratio to identify factors associated with entry to nursing homes within six months of TCP discharge, accounting for the competing risk of death | NA | NA | NA | NA | Dementia was a factor associated with earlier entry to nursing home, adjusted subdistribution hazard ratio: 1.37 95% confidence interval (1.35-1.40) |
| Kosar 2017 | 30-day Readmission | NA | NA | 26.4% of patients with delirium but no dementia were readmitted, compared to 15.3% of patients with no delirium and no dementia (RR 1.70 (1.68, 1.72)  17.2% of patients with delirium and dementia were readmitted, compared to 14% of patients with dementia but no delirium (RR 1.29 (1.28,1.31)) | NA | NA |
| Lee 2011 | Mean length TCP of stay | NA | For participants with dementia: 28.6 days  For all participants: 32.3+/-14.3 days | NA | NA | NA |
| Lei 2022 | Unplanned rehospitalization | NA | NA | 24.9% | NA | NA |
|  | Any primary care visits within 14 days after community discharge | NA | NA | 12.8% | NA | NA |
| Lueckel 2018 | Duration of SNF stay | NA | Those with cognitive impairment had a mean duration  of stay of 31.7 days compared with 27.5 days among those  without cognitive impairment (mean difference  4.2 days, 99.2% confidence interval [3.1, 5.2]. | NA | NA | NA |
| Miu 2016 | 3-month unplanned hospital readmission | NA | NA | NA | Community-dwelling participants with cognitive impairment:  Unplanned hospital admission: yes: 23 (34.3%) no: 44 (65.6%)  Community-dwelling participants with dementia:  Unplanned hospital admission: yes: 12 (38.7%) no: 16 (61.3%)  Community-dwelling participants with no CI:  Unplanned hospital admission: yes: 23 (22.3%) no: 80 (77.7%)  Unplanned 3-month hospital admission was significantly  higher among the delirium group (42.9% vs 24.1%, p < 0.001). |  |
|  | Mean TCP Length of Stay | NA | Community-dwelling participants with cognitive impairment:  33.5±25.0 days  Community-dwelling participants with dementia:  37.2±26.6  Community-dwelling participants with no CI:  31.7±21.4 | NA | NA | NA |
| Simning 2022 | Home time (time spent at home post TCP discharge) Zero-Inflated Negative Binomial Regression Analyses | NA | NA | NA | For persons with ADRD: -1.22 | NA |

NA=Not applicable; TCP=Transitional care program; SNF=Skilled Nursing Facility; ADRD=Alzheimer’s disease and related dementias; AAPC=average annual percentage change.
